# Qualitative Risk Assessment of Infectious Agents Associated with Canine Importation into Canada, 2023–2024

**DOI:** 10.3201/eid3208.251602

**Published:** 2026-08

**Authors:** Vanessa May Leung, Jada Chiasson, Victoria Patterson, Anjali Narasimhan, Maureen E.C. Anderson, Tasha Epp, Christopher Fernandez-Prada, Emilia Wong Gordon, Jean-Philippe Rocheleau, Jason W. Stull, Margo Vachon, Scott Weese, Katie M. Clow

**Affiliations:** University of Guelph, Guelph, Ontario, Canada (V.M. Leung, J. Chiasson, V. Patterson, A. Narasimhan, S. Weese, K.M. Clow); Ontario Ministry of Agriculture, Food and Agribusiness, Guelph (M.E.C. Anderson); University of Saskatchewan, Saskatoon, Saskatchewan, Canada (T. Epp); University of Montreal, Saint-Hyacinthe, Quebec, Canada (C. Fernandez-Prada, J.-P. Rocheleau); Haven Veterinary Services, Vancouver, British Columbia, Canada (E. Wong Gordon); University of Prince Edward Island, Charlottetown, Prince Edward Island, Canada (J.W. Stull); Canadian Food Inspection Agency, Ottawa, Ontario, Canada (M. Vachon)

**Keywords:** canine importation, infectious agents, zoonoses, disease emergence, risk assessment, Canada

## Abstract

Dog importation into Canada has grown substantially since the early 2010s, and concerns have been raised regarding the potential introduction of infectious agents. We conducted a qualitative risk assessment of infectious agents associated with canine importation into Canada. We adapted international risk assessment methodologies coupled with an expert consultation. We estimated likelihood of entry, likelihood of canine exposure, and magnitude of impact of exposure to individual canines and the canine population for all hazards (n = 53). We also completed estimates for likelihood of human exposure and magnitude of impact of exposure on individual humans and the human population for zoonotic hazards (n = 33). Hazards were commonly ranked moderate or high for likelihood of entry and exposure estimates. Magnitude of impact of exposure estimates were assessed at much lower levels, indicating more restricted health impacts. Our study provides a foundation for future risk mitigation, including caregiver education, veterinary assessments, and importation regulations.

The importation of dogs into Canada has grown substantially since the early 2010s (*1*,*2*); an increase of >400% occurred from 2013 to 2019. In 2019, it was estimated that >37,000 dogs entered Canada from a vast array of countries (*2*). Numerous factors have likely contributed to the trend. Rescue organizations appear to be one of the primary sources of imported dogs (*3*); those organizations are often motivated by philanthropic goals (*4*). Rescue organizations transport dogs that might be unhoused, kept in poor conditions, destined for the food chain (i.e., at risk for being slaughtered for meat), or displaced from natural disasters (*1*,*5*). Another primary source of imported dogs is sales from intensive breeding facilities (i.e., puppy mills), particularly in Slovakia, Hungary, Ukraine, and Poland (*2*,*6*). Growing societal demand for specific breeds of dogs has fueled unethical breeding practices and the transportation of large numbers of puppies into many high-income nations, including Canada (*1*,*5*). Those populations of dogs are likely at high risk for exposure to and infection with infectious agents, given they might live in high dog-density situations; have unrestricted access to other animal populations (e.g., wildlife, livestock) and contaminated environments (e.g., waste facilities, polluted waterways); lack access to basic preventive care; or might be immunosuppressed from stress, malnutrition, and other concurrent health issues (*1*,*7*). Moreover, those infectious agents could be foreign to Canada or occur at a much higher prevalence than within Canada.

Unsurprisingly, public and animal health concerns have been raised regarding the potential introduction of infectious agents that are considered novel or well-controlled in Canada through imported dogs and the subsequent effects on dogs and humans in Canada (*5*,*8*). For example, in 2017, canine influenza virus was introduced through imported dogs from Asia, which led to multiple outbreak clusters in a naive dog population in Canada (*9*). In 2018, a woman from British Columbia tested positive for *Brucella canis* infection after handling an infected dog imported from Mexico (*10*). Two separate introductions of canine-variant rabies virus occurred in 2021 in dogs imported from Iran, which resulted in massive animal and public health responses (*11*,*12*). Those documented cases likely represent the tip of the iceberg, because no formalized mandatory systems exist for monitoring canine importation or reporting of cases in Canada, aside for protocols for a select number of immediately notifiable or reportable diseases (*13*,*14*).

In Canada, the Canadian Food Inspection Agency governs the importation of animals from other countries. Dogs must be identified as either personal or commercial imports and meet the corresponding importation requirements (*15*). Commercial importation includes almost all purposes of entry, including dogs for sale or adoption. Personal importation is restricted to dogs belonging to individual persons, and those dogs typically (but not strictly) travel with their owners; that type of importation is associated with the lowest entry requirements. However, the personal importation route has also been used, albeit not as intended, by rescue organizations. For example, some rescues organizations request flight parents, whereby persons traveling abroad volunteer to temporarily claim a dog from a rescue organization as their own to enable ease of entry into Canada and subsequently return the dog to the rescue organization for adoption (*16*,*17*). Currently, importation regulations set by the Canadian Food Inspection Agency predominantly focus on vaccination of imported dogs against rabies. The most stringent requirements exist for commercial dogs <8 months of age, and any commercial dog from a country deemed high risk for canine rabies is currently banned from entry (*18*). Minimal requirements exist for most other dogs, and no requirements for specific pathogen testing are in place (*15*).

Given the substantial gaps in knowledge regarding canine importation in Canada, an evidence-based approach is critically needed to inform risk estimates and prioritize subsequent risk-reduction measures. To fill those gaps, we conducted a comprehensive qualitative risk assessment of infectious agents associated with canine importation into Canada. A risk assessment using a formalized methodology is an internationally recognized tool that has been widely used for importation of animals and animal products to systematically estimate the likelihood of entry of specific hazards and the magnitude of their impact (*19*,*20*).

## Methods

### Hazard Identification

**Figure 1:**
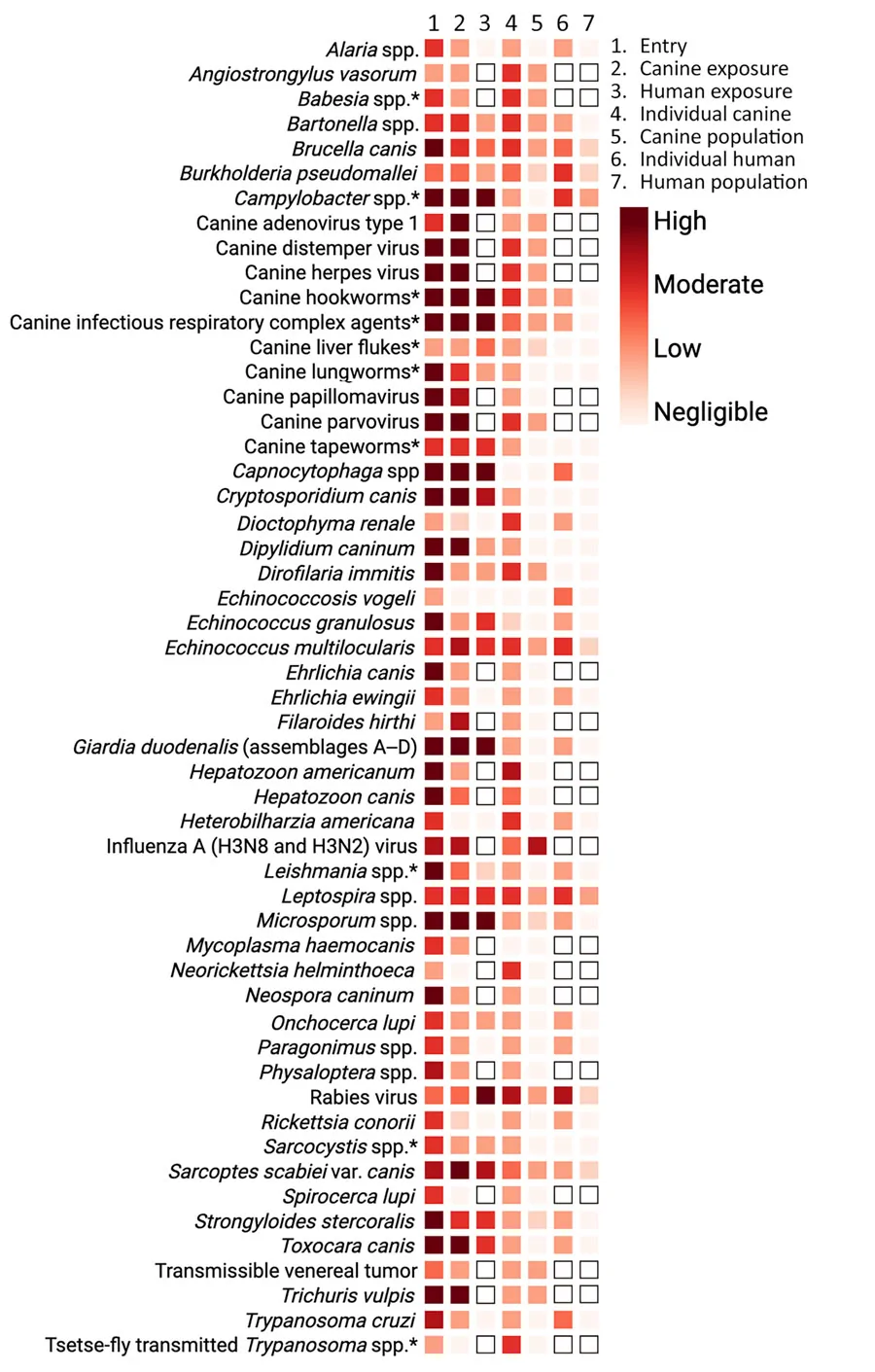
Heat map of qualitative estimates for all 53 hazards (arranged alphabetically) potentially associated with canine importation into Canada as determined through a qualitative risk assessment with expert consultation, 2023–2024. Estimates from left to right are likelihood of entry, likelihood of canine exposure, likelihood of human exposure, magnitude of impact of exposure for an individual canine, magnitude of impact of exposure for the canine population, magnitude of impact of exposure for an individual human, and magnitude of impact of exposure for the human population. For nonzoonotic hazards, estimates were not provided for human categories and are indicated by blank squares outlined in black. Hazards marked with an asterisk are groupings of agents. Full descriptions of those groups are available in Appendix 1 Table 3.

We scanned multiple primary, secondary, and tertiary data sources (*21*,*22*) to generate a comprehensive list of canine-associated infectious agents on the basis of inclusion and exclusion criteria established a priori (Table 1; Appendix 1 Figure 1). Any infectious agent that is foreign, rare, or endemic to Canada; that can cause disease in dogs; and for which dogs play a primary role in transmission was eligible for inclusion.

**Table 1:** Inclusion and exclusion criteria for hazard identification used in qualitative risk assessment of infectious agents associated with canine importation into Canada, 2023–2024

Inclusion criteria	Exclusion criteria
An infectious agent that can cause disease in dogs and/or another host for which dogs are a reservoir/primary host.	An infectious agent that can cause disease in dogs and/or another host for which dogs are dead-end or incidental hosts.
Might or might not be present in Canada.	External parasites, regardless of whether they are vectors for the identified hazards.

### Risk Assessment Methodology Development

We adapted the methodology for risk assessment from the World Organization for Animal Health Handbook on Risk Analysis for Animals and Animal Products and the Tripartite Joint Risk Assessment Operational Tool (*19*,*20*). For this risk assessment, we considered domestic canids and humans. We limited effects to health and did not expand into other areas such as economics and international trade. On the basis of that scope, we deemed 3 likelihood estimates and 4 magnitude estimates to be relevant (Table 2; Appendix 1 Figure 1).

**Table 2:** Guiding questions, key factors for consideration and underlying assumptions for each assessment completed in a qualitative risk assessment of infectious agents associated with canine importation into Canada, 2023–2024

Assessment	Guiding question	Key factors for considerations	Assumptions
Likelihood of entry	What is the likelihood of entry of hazard x through an infected dog imported into Canada in the next 12 mo?	Biologic factors including age and breed of dog as well as testing, treatment, and vaccination requirements for dog; exporting country factors including the prevalence of the hazard in country; importation factors including the quantity of dogs from specific countries	The ban on commercial dog importation from countries considered high-risk for canine rabies does not affect the major and minor countries of importation or the volume of dogs entering Canada but does increase the proportion of dogs entering through the personal route, especially from countries impacted by the ban. Testing, treatment, and preventive healthcare will vary widely by route (i.e., commercial has higher requirements for dogs <8 mo of age), country of origin, and importer. Documentation accompanying dogs may be falsified. Therefore, no measures will be considered when conducting the entry assessment. Despite the import requirement that dogs need to appear healthy on arrival, dogs will enter Canada displaying clinical signs of disease and not be prevented entry. Imported dogs will generally be young, have come from high-density housing situations (e.g., breeding facility, shelter) or higher risk populations (e.g., street dogs) and be immunosuppressed because of the stress of travel (among other reasons).
Likelihood of exposure	Canine: What is the likelihood of exposure to hazard x of >1 canine through an infected dog imported into Canada in the next 12 mo? Human: What is the likelihood of exposure to hazard x of >1 human through an infected dog imported into Canada in the next 12 mo?	Biologic factors including incubation period, transmission period, transmission route(s) and amount of pathogen required (e.g., infectious dose); importing country factors including the presence of vector and/or intermediate host and suitability of environmental conditions	Imported dogs are predominately intended to become household pets with high levels of contact with people and other domestic dogs; if a dog is showing clinical signs of illness, it does not mean that an animal will be appropriately handled (e.g., undergo a period of isolation, access veterinary care); not all dogs will receive veterinary care within a reasonable time period (e.g., 1 to 2 weeks) after arrival in Canada.
Magnitude of individual health consequences	Canine: What is the magnitude of the impact of exposure to hazard x on an individual dog from dogs imported into Canada in the next 12 mo? Human: What is the magnitude of the impact of exposure to hazard x on an individual human from dogs imported into Canada in the next 12 mo?	Impact of exposure to a hazard to an individual can vary considerably within a population. To conceptualize this question, consider a group of 100 individuals and envision the likely distribution of individual health consequences.	Dogs will have access to veterinary care and their caregivers will have sufficient funds to cover diagnostic testing; persons will have access to healthcare and have sufficient funds for diagnostic testing (if required).
Magnitude of population health consequences	Canine: What is the magnitude of the impact of exposure to hazard x on the dog population overall in Canada from dogs imported into Canada in the next 12 mo? Human: What is the magnitude of the impact of exposure to hazard x on the human population overall in Canada from dogs imported into Canada in the next 12 mo?	Impact of exposure to a hazard at the population level focuses on the degree of canine-to-canine or human-to-human spread (household, regional, national) and the capacity of health systems to respond. The current status of the pathogen in Canada is considered the baseline level and the assessment is for the contribution of canine importation to the level of the pathogen above that baseline.	Companion animal surveillance capacity is not considered as there are minimal formalized structures in Canada aside from professional networks; appropriate animal health or public health resources will be allocated if issues arise.

For each estimate, we developed a guiding question with a set of parameters that defined the scale of the estimate (i.e., number of dogs infected/exposed) and time frame of occurrence. To ensure that rare but potentially important events were captured, the scale was set at the lowest threshold of 1 infected or exposed dog. To reflect the current context (e.g., agent prevalence, importation trends, regulations), we set the time frame to cover the next 12 months (Table 2). We outlined a list of key factors to consider for each question, as well as underlying assumptions (Table 2). We chose qualitative estimate levels of negligible, low, moderate, and high and outlined interpretations of each level and example scenarios (Appendix 1 Tables 1, 2).

### Literature Review

We used peer-reviewed primary and secondary data sources to compile information on each hazard (Appendix 1 Figure 1; Appendix 2 Table; Appendix 3 Table). For likelihood of entry and exposure estimates, we compiled up-to-date information on geographic distribution, status in major and minor exporting countries, and agent dynamics (e.g., modes of transmission, vector or host species involved, incubation period, transmission period). On the basis of commercial importation records from the Canada Border Services Agency, we considered the United States, Mexico, Slovakia, Hungary, South Korea, Poland, Russia, Australia, and Taiwan the major countries of export and Colombia, China, Dominican Republic, United Kingdom, South Africa, Greece, Egypt, Türkiye, and Thailand minor countries of export (*2*). For magnitude of impact of exposure estimates, we gathered up-to-date information on potential spread scenarios, disease manifestation in canine and human hosts, and disease prevention and management options. For each hazard, we assigned an uncertainty estimate on the basis of the available literature (Table 3; Appendix 2 Table; Appendix 3 Table).

**Table 3:** Criteria for assigning an uncertainty estimate for available data for a qualitative risk assessment of infectious agents associated with canine importation into Canada, 2023–2024

Uncertainty estimate	Interpretation
Low	Reliable data available from ongoing national or sporadic research surveillance; strong evidence provided in multiple data sources and similar conclusions reported.
Moderate	Some gaps in availability or reliability of data available (e.g., only case reports and gray literature); lack of surveillance data; evidence provided in multiple references and findings reported vary.
High	Limited or lack of data or reliable information; evidence provided in veterinarian reports or subjective assessments; results based on limited consensus.

### Expert Consultation

We invited persons with expertise as measured by demonstrated research productivity or organizational role in canine and human infectious disease, shelter and community medicine, epidemiology, or regulatory medicine in Canada to participate in the expert consultation. Initially, we extended 12 invitations for participation; all persons accepted. However, because of competing demands, including the late stages of the COVID-19 pandemic and the onset of the highly pathogenic avian influenza pandemic, 4 persons resigned during the early stages of the project. The remaining 8 participants made substantial contributions to the risk assessment and are acknowledged as coauthors. We sought no additional infectious agent–specific expertise.

We used a modified version of the Delphi method to elicit expert opinion (*23*–*25*) and shared the compiled list of hazards in advance to ensure comprehensiveness. We flagged any potentially missing hazards and further evaluated them on the basis of the established inclusion and exclusion criteria (Table 1). We conducted a pilot round with 3 hazards to ensure clarity of wording and consistency of interpretation of methodology and to determine whether the provided information generated through the literature review was sufficient. We made minor modifications to the methodology on the basis of expert feedback.

**Figure 2:**
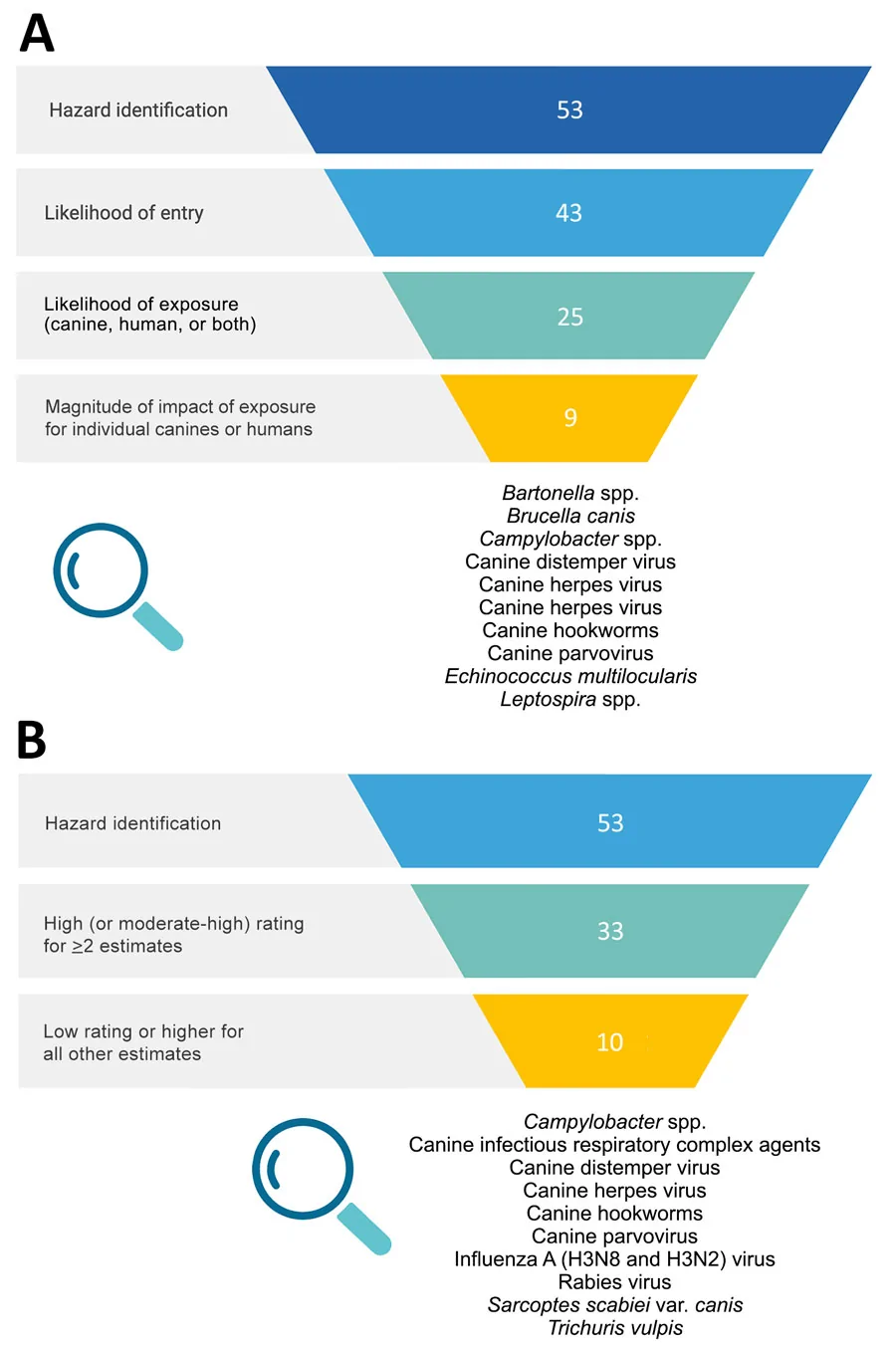
Approaches used to prioritize hazards in qualitative risk assessment of infectious agents associated with canine importation into Canada, 2023–2024. A) For the first approach, of the 53 hazards potentially associated with canine importation into Canada, 43 were ranked moderate or high likelihood of entry, 25 were ranked moderate or high likelihood of canine and/or human exposure, and 9 had a ranking of moderate or high for >1 of the 4 magnitudes of impact of exposure estimates. B) For the second approach, of the 53 hazards potentially associated with canine importation into Canada, 33 received a ranking of high (or moderate-high) for >2 estimates, and 10 had a minimum ranking of low or higher for all other estimates. Zoonotic hazards were only required to have a minimum ranking of low or higher for estimates related to 1 population (dogs or humans).

For each round of assessments, we provided experts with a summary data sheet for 8–10 hazards (Appendix 2 Table; Appendix 3 Table). We also provided pathway diagrams to assist with methodological interpretation (Appendix 1 Figures 2–7). We presented risk assessment questions for each hazard through an online questionnaire (Qualtrics, https://www.qualtrics.com), and experts submitted their individual estimates, along with their justification (optional) and level of uncertainty. A completion level of 50% plus 1 person was required before advancing to a discussion.

After each questionnaire, we held a virtual meeting to discuss areas of limited consensus. For meetings to occur, an attendance level of 50% plus 1 person was required. Limited consensus was defined for each assessment by 2 criteria: >30% of respondent experts differed in their ranking but were within 1 level of each other or 2 experts differed in their ranking by >1 level (Appendix 1 Figure 1).

Upon completion of all assessments, we shared estimates for all hazards. We invited experts to identify any areas of inconsistency for discussion and potential reevaluation.

### Synthesis

We calculated descriptive statistics for each estimate according to ranking levels. We compiled all estimates for all hazards into a heat map to illustrate rankings (negligible to high) across risk areas (entry to population level) for each of the hazards (BioRender, https://www.biorender.com).

We completed a prioritization activity once all hazards were assessed using 2 approaches agreed upon by the expert panel (Appendix 1 Figure 1). For the first approach, hazards were considered priorities if they were ranked moderate or high for likelihood of entry, followed by >1 likelihood of exposure estimate, and then moderate or high for >1 magnitude of impact of exposure estimate. For the second approach, hazards were considered priorities if they were ranked high (including moderate-high) for >2 estimates. They also needed to receive a minimum ranking of low for all other estimates; an exception was made for zoonotic agents, in which they could still be classified as high priorities as long as they received a ranking of low or above for 1 population of interest. This process enabled us to identify potentially high priority hazards for canines even if they are low priority for humans and vice versa.

## Results

### Hazard Identification

The hazard identification process yielded 74 infectious agents that are potentially associated with canine importation into Canada (Appendix 1 Table 3). They consisted of 14 bacteria, 9 viruses, 50 parasites, and 1 transmissible neoplasm. To increase efficiency, we assessed a subset of agents from the same genus or group (e.g., hookworms) together if there was considerable overlap in the factors considered for entry, exposure, and magnitude of impact. In total, we made estimates for 53 agents or agent groups (each herein referred to as a hazard). Of those 53 hazards, 33 were considered zoonotic. We determined estimates related to exposure and impact for humans only for the zoonotic subset.

### Expert Consultation

In the 14-month period during July 2023–August 2024, we distributed 6 questionnaires. We held eight 90-minute online meetings across that timeframe to discuss areas of limited consensus.

A final consensus could not be reached for numerous estimates despite discussion (i.e., the number of responses in adjacent categories was equal). We made an a posteriori decision to set the estimate between the 2 categories, which created 3 additional rankings: negligible-low, low-moderate, and moderate-high.

### Final Assessments

#### Likelihood Estimates

We classified >80% (n = 43/53) of hazards assessed as moderate or high likelihood of entry through >1 infected imported canine over the next 12 months (Figure 1; Appendix 1 Figure 8, panel A; Appendix 4 Table 1). Likelihood of exposure to >1 canine through an infected imported dog over the next 12 months was ranked moderate or high for approximately half (49%) of the hazards (n = 26/53) (Figure 1; Appendix 1 Figure 8, panel B; Appendix 4 Table 1). Of the zoonotic hazards, relatively equal numbers of hazards were ranked in each category (Figure 1; Appendix 1 Figure 8, panel B; Appendix 4 Table 1).

#### Magnitude of Impact of Exposure

For magnitude of impact of exposure for an individual canine, the highest ranking was moderate-high for 2 hazards (rabies virus and *Hepatozoon americanum* protozoa). Most hazards (79%, n = 42/53) ranked low (n = 27) or moderate (n = 15) (Figure 1; Appendix 1 Figure 8, panel C; Appendix 4 Table 1). At the canine population level, all hazards were ranked negligible or low for magnitude of impact of exposure, except canine influenza virus, which was ranked moderate-high (Figure 1; Appendix 1 Figure 8, panel C; Appendix 4 Table 1).

Similarly, for magnitude of impact of exposure for individual humans, the highest ranking was moderate-high for 1 hazard (rabies virus), followed by moderate for 4 hazards (*Echinococcus multilocularis* tapeworm and 3 bacteria, *Campylobacter* spp., *Leptospira* spp., and *Burkholderia pseudomallei*) (Figure 1; Appendix 1 Figure 8, panel D; Appendix 4 Table 1). Of the 33 zoonotic hazards, half of the hazards (n = 17/33) were ranked low. At the human population level, 79% (n = 26/33) of the zoonotic hazards were ranked negligible for magnitude of impact of exposure; the remaining were ranked negligible-low (n = 5) or low (n = 2) (Figure 1; Appendix 1 Figure 8, panel D; Appendix 4 Table 1).

#### Uncertainty

Uncertainty estimates were variable across experts. High uncertainty was consistently reported by experts for the likelihood of entry for *Echinococcus vogeli* tapeworms and the magnitude of impact of exposure at the canine population level for canine hookworms (Appendix 4 Table 2).

#### Priority Hazards

Using the first approach, 43 hazards ranked moderate or high for likelihood of entry, and 25 also ranked moderate or high for likelihood of exposure to canines, humans, or both. Of those 25, the following 9 ranked moderate or high for magnitude of impact of exposure for individual canines or humans: *Bartonella* spp., *B. canis*, *Campylobacter* spp., canine distemper virus, canine herpes virus, canine hookworms, canine parvovirus, *E. multilocularis* tapeworm, and *Leptospira* spp. (Figure 2, panel A). Using the second approach, 17 hazards ranked moderate-high or high in >2 categories. Of those 17 hazards, the following 10 met the criteria of having a minimum ranking of at least low for all other estimates: *Campylobacter* spp., canine infectious respiratory disease complex agents, canine distemper virus, canine herpes virus, canine hookworms, canine parvovirus, influenza A virus (H3N8, H3N2), rabies virus, *Sarcoptes scabiei* var. *canis* mites, and *Trichuris vulpis* nematodes (Figure 2, panel B).

## Discussion

We identified 74 infectious agents potentially associated with canine importation into Canada and systematically assessed their risks using an evidence-based methodology. We propose that, although entry of and exposure to these infectious agents is likely occurring frequently, the overall health impacts are likely low.

A substantial proportion of identified hazards received a ranking of moderate or high for entry. That finding is perhaps unsurprising given the large number of dogs that are estimated to enter Canada every year (*2*) and the lack of specific regulatory controls. As a result, even if a pathogen has a low prevalence in the imported dog population, the odds of >1 infected dog entering Canada are increased, on the basis of the volume of canine importation. The moderate or high likelihood of exposure for more than one third of hazards strongly suggests that those agents could also spread within the domestic canine population. Indeed, previous events within Canada, such as the canine influenza outbreak, provide evidence for those estimates (*9*). The caregivers of those dogs might be at risk for exposure for a subset of those hazards as well.

Although numerous hazards were deemed as moderate or high likelihood for entry and exposure, we found the magnitude of impact of exposure to be much more restricted across all hazards. This positive result of our risk assessment indicates that, although entry and exposure of many agents associated with canine importation occurs readily, the overall effects at the individual and population levels for both canines and humans likely present low to negligible risk.

Overall, this study provides strong justification for improved risk mitigation measures that generally target infectious agents, including improved health screening of dogs before or at entry (e.g., no overt clinical signs), as well as restricted contact of imported dogs with other dogs and humans upon arrival. A comprehensive veterinary assessment with the dog’s primary care veterinarian after arrival is also indicated. Given the vast array of agents that could enter, regardless of exposure potential, this assessment is necessary to support the health of imported dogs and ensure those dogs receive the necessary preventive and specialized supportive medical care (*3*). Developing targeted educational materials will be a key step in ensuring uptake of those measures by rescue organizations, prospective adopters, and animal health professionals.

To further refine the outcomes of the risk assessment, we undertook 2 approaches for hazard prioritization, both of which generated different lists. The first approach (Figure 2, panel A) was stepwise from entry through to magnitude of individual impact and only included those hazards that were ranked moderate or high at each estimate. The generated list included hazards that are of high prevalence in many importing countries, are readily transmissible within Canada, and cause disease in canines (or humans). The priority list is likely of greatest value to practicing veterinarians seeing imported dogs or domestic dogs in close contact with imported dogs who can provide direction on diagnosis and treatment. However, the list likely misses other nuances in risk, because risks of potentially higher magnitude of impact but slightly lower likelihood of entry or exposure would be excluded. The second approach (Figure 2, panel B) captures that nuance and is likely of most value to regulators in identifying key targets for in-depth hazard-specific risk assessments, refined importation regulation (e.g., screening, preventive measures), and surveillance, especially in the context of limited veterinary and public health resources.

The methodology used in this study provides a valuable framework that can be adapted for future risk assessments evaluating agents associated with canine and other animal importation. The methodology is reproducible; thus, the risk assessment could be updated in future to reflect global context, research findings, and surveillance data. Questions, metrics, considerations, and assumptions can also all be modified for contexts outside of Canada. We strongly recommend the integration of expert consultation into future studies; this method proved effective at filling in knowledge gaps and gathering lived expert experience, which is not adequately documented in peer-reviewed literature. The modified version of the Delphi method, which has been widely used in research to generate agreement and identify priorities (*25*–*27*), was invaluable. Although initial surveys tended to result in limited agreement among experts, subsequent meetings produced effective collaboration and were successful in achieving consensus. We also recommend consistently revisiting the methodology and established criteria for assessment. Considering the broad scope, this step was necessary to maintain clarity and ensure all experts were approaching their estimates consistently.

The first limitation of this study is that, given the sheer number of canine-associated infectious agents, some might have been missed during our literature scan, particularly for newly emerging agents, where the epidemiologic role of dogs has not yet been fully established. Moreover, we excluded external parasites to maintain scope. Given the growing threat of foreign tick introduction through dogs (*28*,*29*), the inclusion of external parasites would have brought greater value. We do, however, believe that our risk assessment is a strong starting point given the comprehensive nature of the hazards included. Furthermore, the thresholds used in this risk assessment were low, which might not have appropriately accounted for the variation between infectious agents. Specifically, although a single infected canine might pose minimal risk for 1 agent, for others, a single infection could have major implications for animal and human health. Future risk assessments should consider collecting and subsequently incorporating quantitative data to reflect the frequency of introduction and provide further refined risk estimates. Several aspects of our methodology were based on the work of Blackmore et al. (*2*), which included data up to 2019. Spatial and temporal trends related to canine importation might have changed since that time; we were unable to adjust our assessments because up-to-date data were not readily available. In addition, reliable data on the prevalence and transmission of many agents were lacking, both from foreign surveillance and within Canada, which was likely the greatest limitation. The inclusion of uncertainty estimates was critical to identify those gaps. Enhanced, coordinated international surveillance for specific hazards would assist in reducing those knowledge gaps.

In conclusion, our qualitative risk assessment indicates that, although imported dogs frequently introduce various infectious agents into Canada, the effects on public and animal health for most of those agents remain low overall. Targeted measures focusing on the few high-risk agents could substantially reduce overall risk. Our findings provide evidence to support enhanced health screening measures at the time of importation, greater coordinated surveillance, and improved public education, benefiting both canine and human populations. Moreover, they provide a starting point for more in-depth, hazard-specific risk assessments.

Appendix 1 Additional methodological information about qualitative risk assessment of infectious agents associated with canine importation into Canada, 2023–2024

Appendix 2 Additional information used for likelihood estimates for a qualitative risk assessment of infectious agents associated with canine importation into Canada, 2023–2024

Appendix 3 Additional information used for magnitude of impact estimates for a qualitative risk assessment of infectious agents associated with canine importation into Canada, 2023–2024

Appendix 4 Additional data generated from qualitative risk assessment of infectious agents associated with canine importation into Canada, 2023–2024
