# Supplementary material for: Qualitative Risk Assessment of Infectious Agents Associated with Canine Importation into Canada, 2023–2024
Source: Emerg Infect Dis. 2026 Aug;32(8):1231–40. doi: 10.3201/eid3208.251602 (PMC13426852; doi:10.3201/eid3208.251602)
Supplement: Appendix 1 — Additional methodological information about qualitative risk assessment of infectious agents associated with canine importation into Canada, 2023–2024 [file 25-1602-Techapp-s1.pdf]

Article DOI: https://doi.org/10.3201/eid3208.251602

EID cannot ensure accessibility for supplementary materials supplied by authors. Readers who have difficulty accessing supplementary content should contact the authors for assistance.

Qualitative Risk Assessment of Infectious Agents Associated with Canine Importation into Canada, 2023–2024

Appendix 1

Appendix 1 Table 1. The likelihood estimate categories and accompanying interpretation for the entry and exposure assessment of hazards associated with canine importation for a qualitative risk assessment of infectious agents associated with canine importation into Canada, 2023–2024*

Likelihood estimate	Interpretation	Example Scenarios	
		Entry	Exposure
Negligible	The likelihood of the circumstances described in the assessment question occurring is assessed as virtually zero, would occur only under exceptional circumstances.	The disease is rare in all countries.	Transmission is not possible from the dog due to absent vector species or intermediate host.
Low	The likelihood of the circumstances described in the assessment question occurring is unlikely.	The disease is endemic but uncommon or restricted in minor importing countries.	The incubation period for the disease is short (i.e., days) with a short period of infectivity.<br>A vector or intermediate host is required for completion of the transmission cycle, which might be geographically or temporally separate from the dog.<br>Transmission from the infected canine occurs in a minority of cases.
Moderate	The likelihood of the circumstances described in the assessment question occurring is likely.	The disease is endemic but uncommon or restricted in major importing countries.<br>The disease is endemic and common in minor importing countries.	A subset of dogs are subclinical carriers.<br>A vector or intermediate host is required for completion of the transmission cycle, which is common in Canada.<br>Transmission occurs frequently from the canine host.
High	The likelihood of the circumstances described in the assessment question occurring is very likely.	The disease is endemic and common in major importing countries.	Chronic infection common with ongoing transmission possible<br>Highly contagious

*Examples are provided to illustrate scenarios for each category but are not exhaustive.

Appendix 1 Table 2. The impact estimate categories and accompanying interpretation for the magnitude of impact of exposure assessments of hazards associated with canine importation at the individual and population levels for both dogs and humans. Examples are provided to illustrate scenarios for each category but are not exhaustive.

Impact estimate	Individual			Population		
	Interpretation	Canine	Human	Interpretation	Canine	Human
Negligible	The effects are likely indiscernible to an infected canine.	Exposure rarely leads to infection; clinical signs associated with infection are infrequently recognized, and if they occur are mild; no treatment is required.	Exposure rarely leads to infection; clinical signs associated with infection are infrequently recognized, and if they occur are mild; no treatment is required.	The effects are likely indiscernible at any level within Canada.	No additional cases of disease in dogs are reported.	No human cases of disease are reported.
Low	The effects are likely to be minimal for an affected canine.	Most dogs exposed become infected and show mild clinical signs that are self-limiting; most dogs exposed become infected but most do not show clinical signs; if clinical signs are evident, they are moderate in severity.	Most people exposed become infected and show mild clinical signs that are self-limiting. Most people exposed become infected but most do not show clinical signs. If clinical signs are evident, they are moderate in severity.	Effects are likely limited to the household.	Restricted to the household and/or immediate contacts. No interventions are required at the population level. Professional knowledge strong for timely diagnosis and rapid containment. Sufficient laboratory capacity exists to detect disease.	Further spread infrequently recognized. Morbidity is low, no mortality. No interventions are required at the population level. Professional knowledge strong for diagnosis and interventions can be quickly and easily implemented. Sufficient laboratory capacity exists to detect disease. Adequate surveillance in place to track and contain. Exposure to infected canines required (no human-to-human transmission).
Moderate	The effects are likely to be notable for affected individuals.	Most dogs exposed become infected. Clinical signs are moderate and require veterinary intervention. Most dogs exposed become infected but only a subset will show clinical signs. If clinical signs are evident, they are severe and may be fatal. Effective treatment exists and is accessible.	Most people exposed become infected. Clinical signs are moderate and require medical intervention. Most people exposed become infected but only a subset will show clinical signs. If clinical signs are evident, they are severe and may be fatal. Effective treatment exists and is accessible.	The effects are likely limited to the local / regional level.	Further spread occurs within a region. Morbidity notable within a region, mortality rare. Professional knowledge on disease limited, which would delay interventions. Sufficient laboratory capacity exists to detect disease.	Further spread occurs within a region. Morbidity notable within a region, mortality rare. Professional knowledge on disease limited, which would delay interventions. Sufficient laboratory capacity exists to detect disease. Some surveillance in place or can be rapidly implemented to track and contain. Human to human transmission documented, but typically limited.
High	The effects are likely to be catastrophic for affected individuals.	Most dogs exposed become infected. Clinical signs are severe and may be fatal. Effective treatment does not exist or is not accessible.	Most people exposed become infected. Clinical signs are severe and may be fatal. Effective treatment does not exist or is not accessible.	The effects are likely experienced at the provincial / national level.	Further spread documented across several regions. Morbidity and mortality high.	Further spread documented across several regions. Morbidity and mortality high. Professional knowledge minimal, which would delay interventions.

Impact estimate	Individual			Population		
	Interpretation	Canine	Human	Interpretation	Canine	Human
					Professional knowledge minimal, which would delay interventions.<br>Sufficient laboratory capacity does not exist to detect disease.<br>Effective interventions are difficult or impossible to implement.	Sufficient laboratory capacity does not exist to detect disease.<br>Inadequate surveillance in place to track and contain.<br>Sustained human to human transmission possible.<br>Effective interventions are difficult or impossible to implement.

Appendix 1 Table 3. List of hazards compiled during hazard identification for infectious agents associated with canine importation. Superscript notations indicate agents that were assessed together, with the accompany group name indicated.

Bacteria	Viruses	Parasites	Transmissible neoplasia
Bartonella spp.	Canine adenovirus type 1	*Babesia canis* (subsp. *vogeli*, *rossi*, *canis*)[iii]	Transmissible venereal tumor
Bordetella bronchiseptica[i]	Canine adenovirus type 2[i]	*Babesia gibsoni*[iii]	
Brucella canis	Canine distemper virus	*Giardia duodenalis* (assemblages A-D)	
Burkholderia pseudomallei	Canine herpes virus	*Alaria* spp.	
Campylobacter jejuni[ii]	Canine papilloma virus	*Ancylostoma caninum*[iv]	
Campylobacter upsaliensis[ii]	Canine parvovirus	*Ancylostoma braziliense*[iv]	
Capnocytophaga spp.	Canine parainfluenza virus[i]	*Ancylostoma ceylanicum*[iv]	
Ehrlichia canis	Influenza A (H3N8, H3N2) virus	*Angiostrongylus vasorum*	
Ehrlichia ewingii	Rabies virus	*Clonorchis sinensis*[v]	
Leptospira spp.		*Crenosoma vulpis*[vi]	
Microsporum spp.		*Cryptosporidium canis*	
Mycoplasma hemocanis		*Dioctophyma renale*	
Neorickettsia helminthoeca		*Dipylidium caninum*	
Rickettsia conorii		*Dirofilaria immitis*	
		Echinococcosis vogeli	
		Echinococcus granulosus	
		Echinococcus multilocularis	
		Eucoleus aerophilus[vi]	
		Filaroides hirthi	
		Heptazoon americanum	
		Heptazoon canis	
		Heterobilharzia americana	
		Leishmania infantum[vii]	
		Leishmania brazielesis [vii]	
		Neospora caninum	
		Onchocerca lupi	
		Opisthorchis felineus[v]	
		Opisthorchis viverrini[v]	
		Paragonimus spp.	
		Physaloptera spp.	
		Sarcocytis cruzi[viii]	
		Sarcocytis capracanis[viii]	
		Sarcocytis hircicanis[viii]	
		Sarcocytis meisheriana[viii]	
		Sarcocytis fayeri[viii]	
		Sarcoptes scabei var. canis	
		Spirocerca lupi	
		Strongyloides stercoralis	
		Taenia multiceps[ix]	
		Taenia serialis[ix]	
		Taenia crassiceps[ix]	
		Taenia hydatigena[ix]	
		Taenia taeniaeformis[ix]	
		Taenia pisiformis[ix]	
		Toxocara canis	
		Trichuris vulpis	
		Trypanosoma cruzi	
		Trypanosoma congolese[x]	
		Trypanosoma brucei brucei[x]	
		Uncinaria stenocephala[iv]	

[i]canine infectious respiratory disease complex agents
[ii]*Campylobacter* spp.
[iii]*Babesia* spp.
[iv]canine hookworms
[v]canine liver flukes
[vi]canine lungworms
[vii]*Leishmania* spp.
[viii]*Sarcocytis* spp.
[ix]canine tapeworms
[x]tsetse fly-transmitted *Trypanosoma* spp.

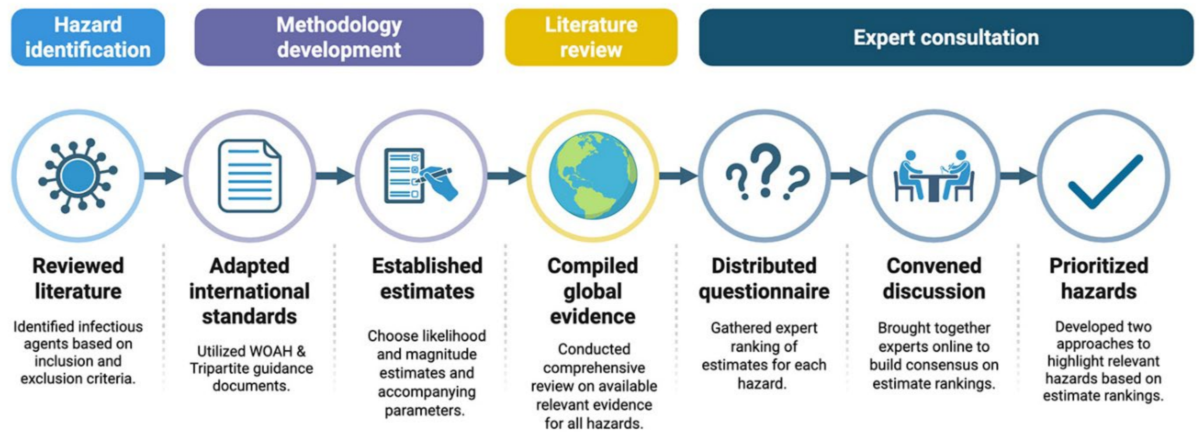

Appendix 1 Figure 1. Methodological flowchart of the key steps for the qualitative risk assessment of canine importation into Canada, 2023–2024. This figure was created in BioRender (BioRender, Toronto, ON, 2025).

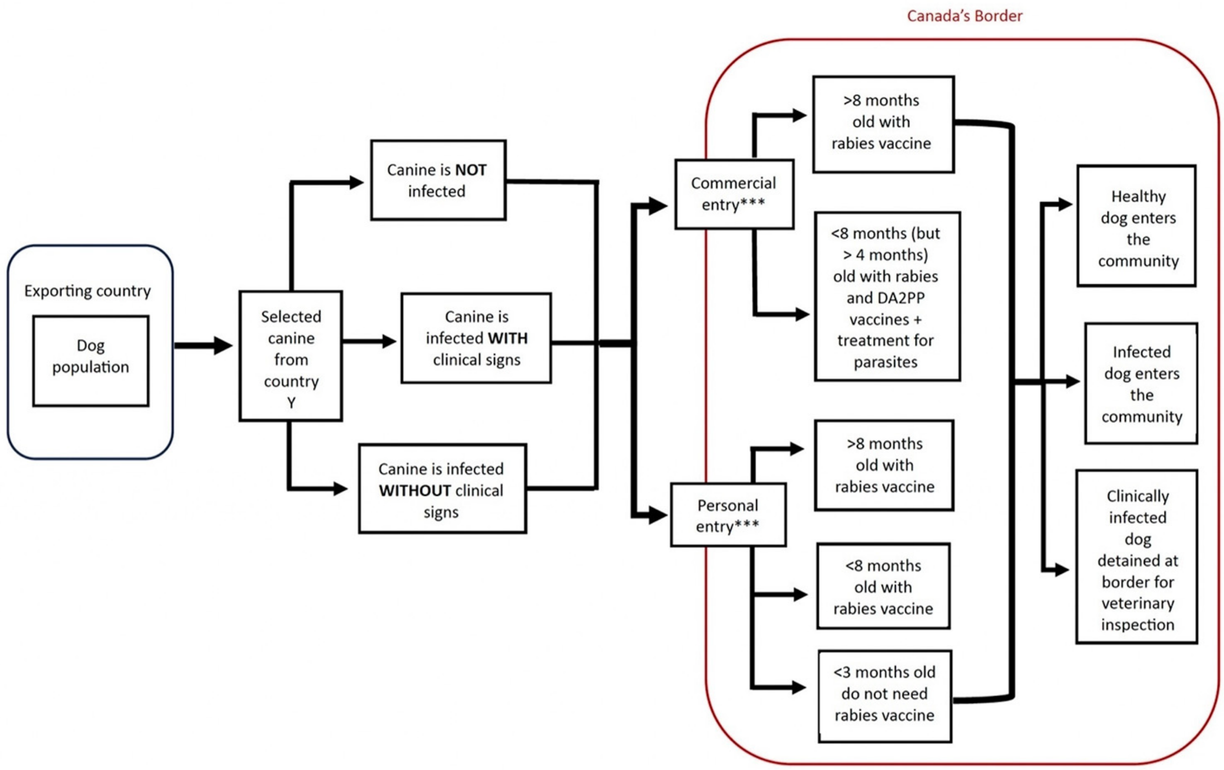

Appendix 1 Figure 2. Generic entry pathways to depict various scenarios of canine importation into Canada. These pathways should not be considered exhaustive. This figure was produced to assist with conceptualization and assignment of likelihood of entry of a hazard via an imported canine during the expert consultation. Asterisks indicate where Canadian importation regulations should be consulted.

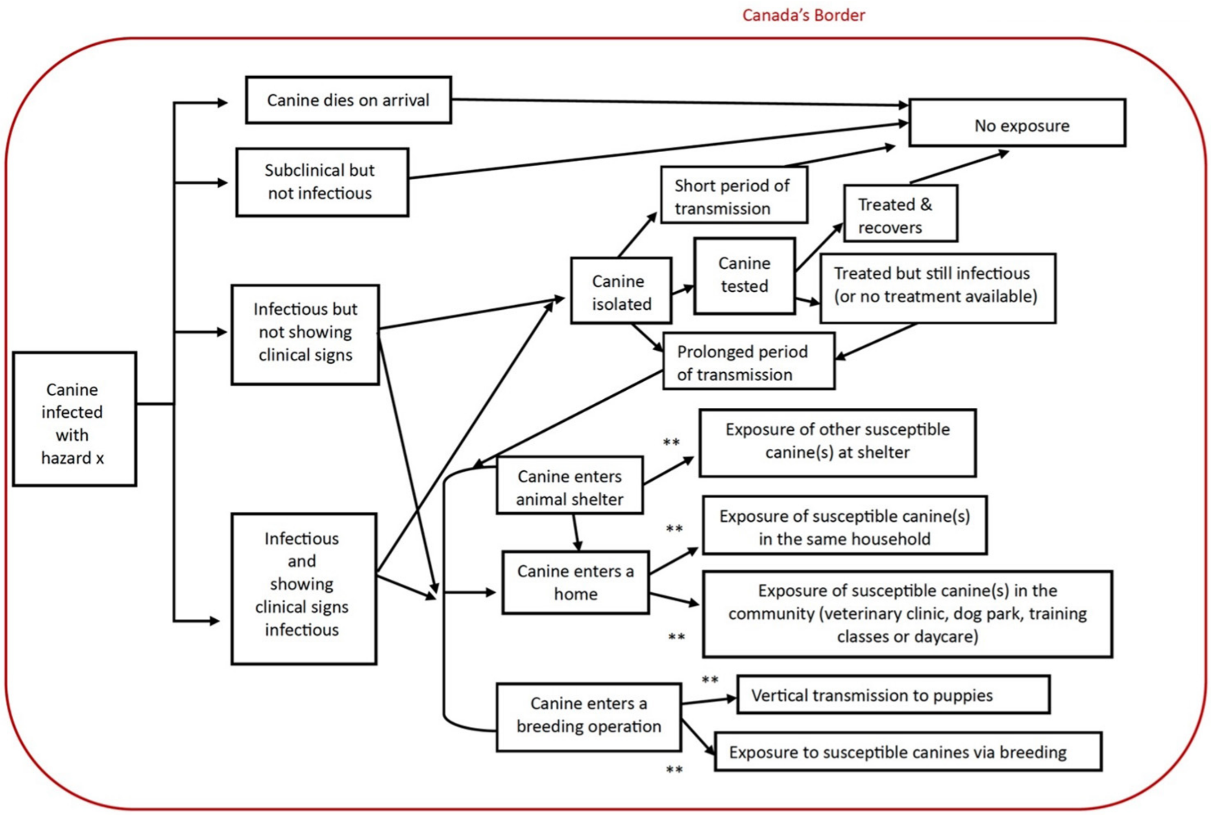

Appendix 1 Figure 3. Generic imported dog to domestic dog exposure pathways to depict various scenarios of canine importation into Canada. These pathways should not be considered exhaustive. This figure was produced to assist with conceptualization and assignment of likelihood of exposure of a domestic canine to a hazard via an imported canine during the expert consultation. ** indicates where spread scenarios should be considered (Appendix 1 Figure 5).

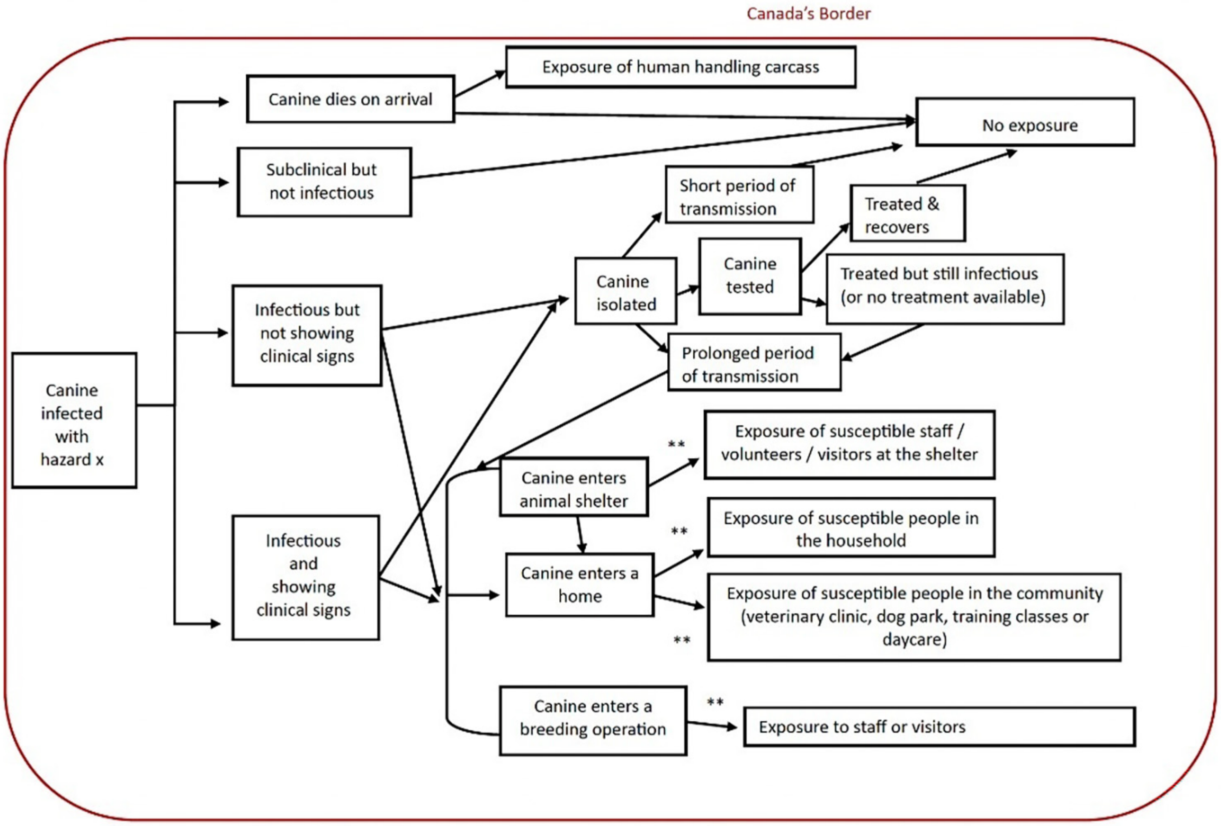

Appendix 1 Figure 4. Generic imported dog to human exposure pathways to depict various scenarios of canine importation into Canada. These pathways should not be considered exhaustive. This figure was produced to assist with conceptualization and assignment of likelihood of exposure of a human to a hazard via an imported canine during the expert consultation. ** indicates where spread scenarios should be considered (Appendix 1 Figures 6 and 7)

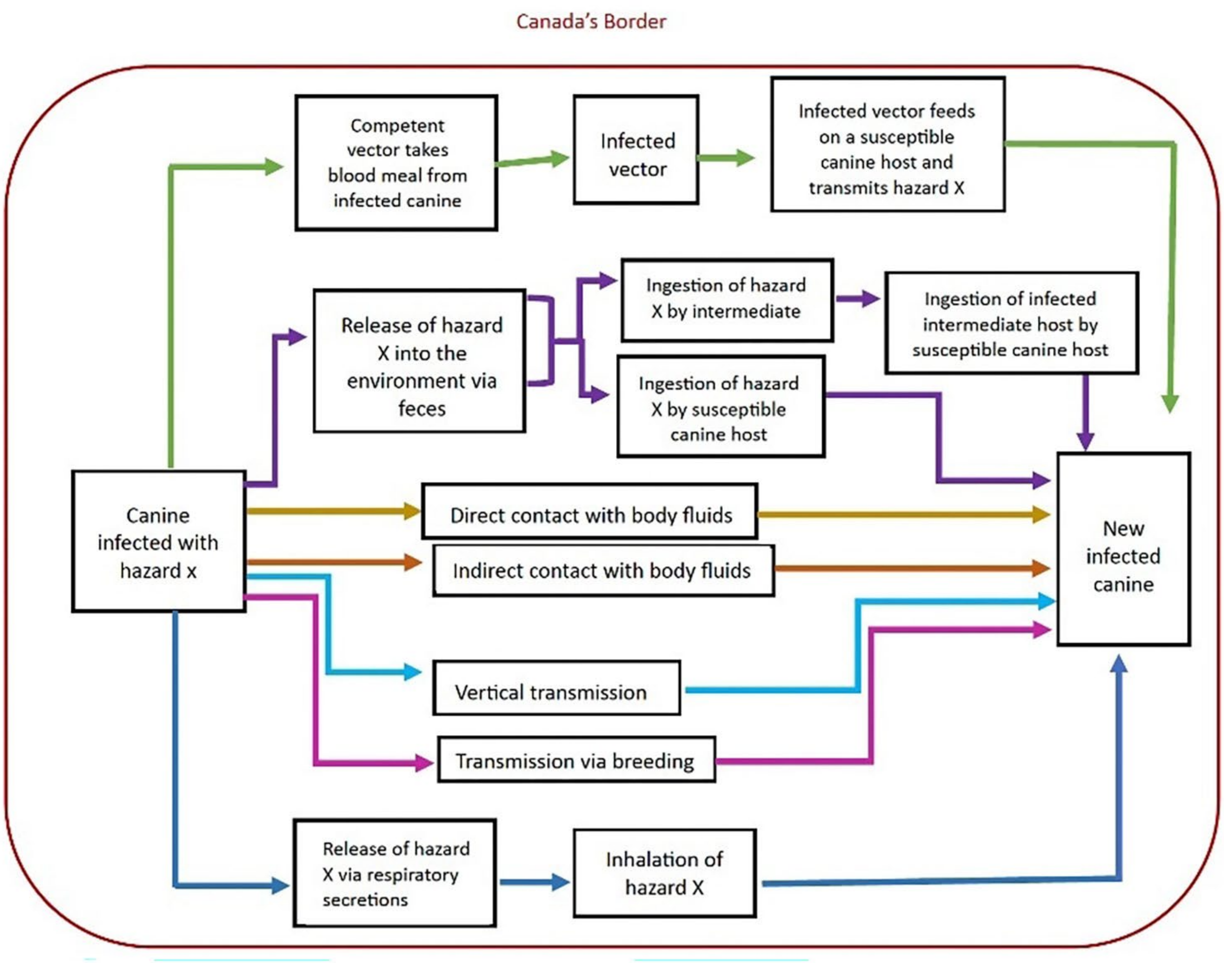

Appendix 1 Figure 5. Potential hazard spread scenarios from an infected imported dog to domestic dog(s). These spread scenarios should not be considered exhaustive. This figure was produced to assist with conceptualization and assignment of likelihood of exposure of a domestic canine to a hazard via an imported as well as the magnitude of impact of exposure at the canine population level.

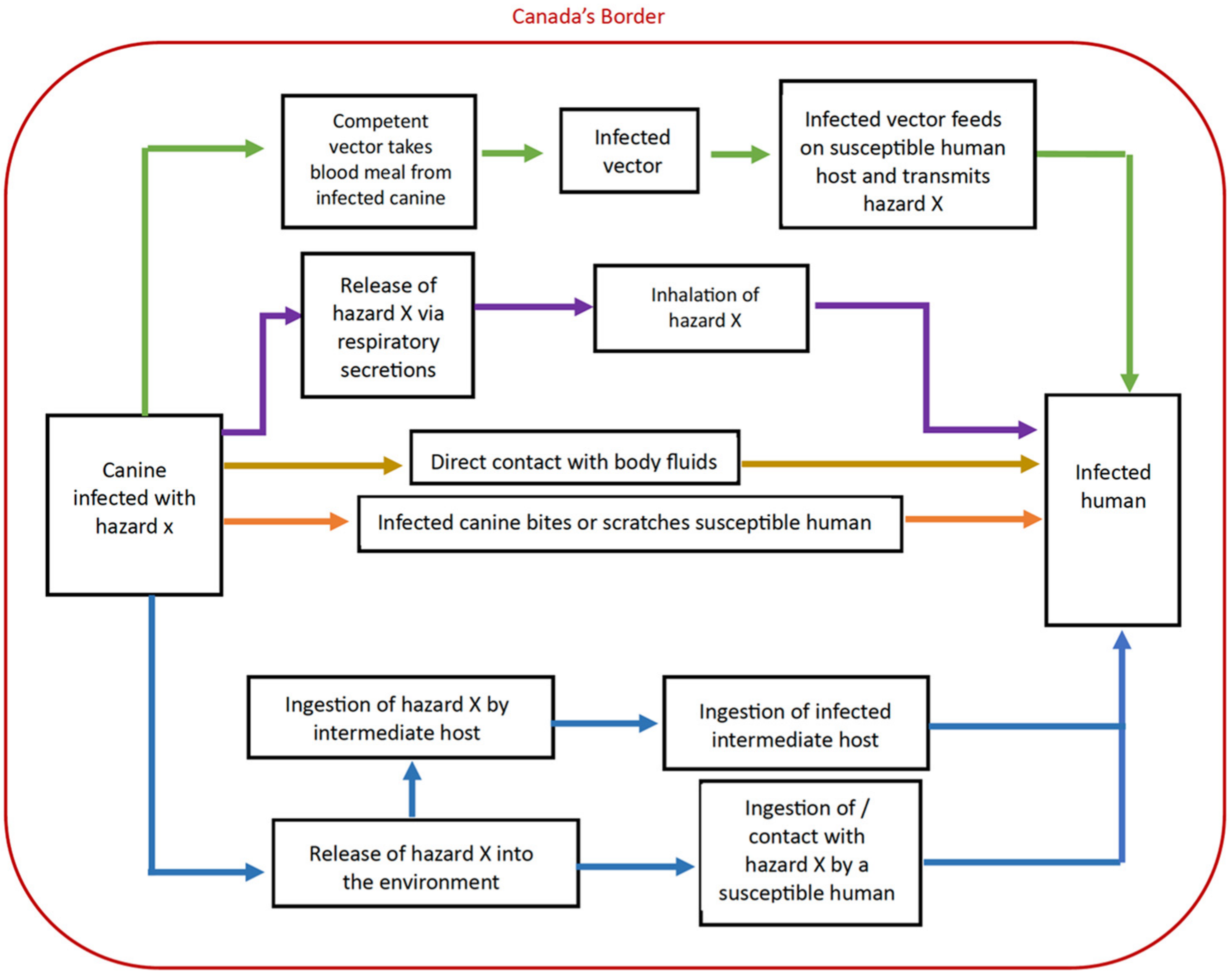

Appendix 1 Figure 6. Potential hazard spread scenarios from an infected imported dog to a human. These spread scenarios should not be considered exhaustive. This figure was produced to assist with conceptualization and assignment of likelihood of exposure of a humans to a hazard via an imported as well as the magnitude of impact of exposure at the individual human level.

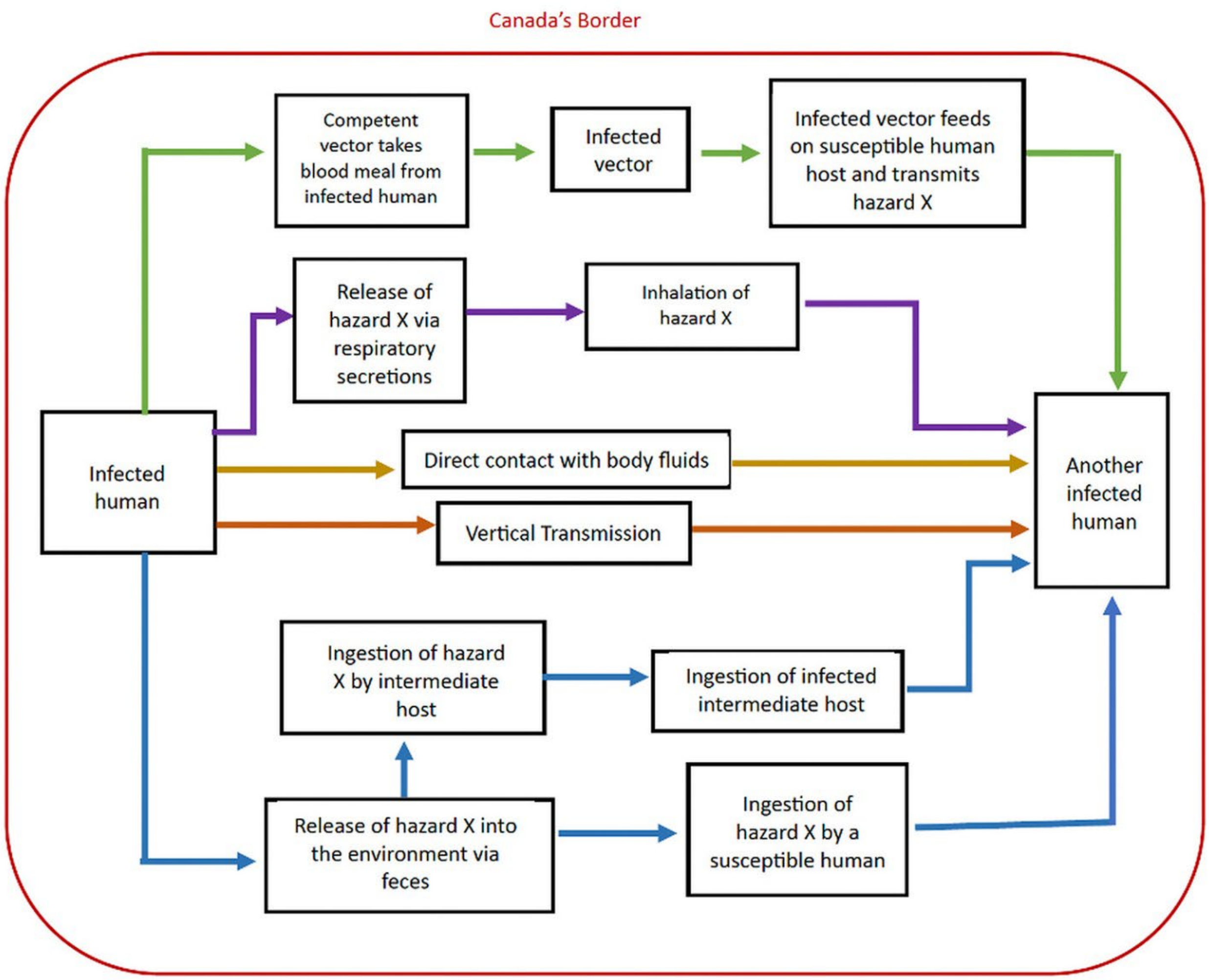

Appendix 1 Figure 7. Potential hazard spread scenarios from a human infected with a hazard by an imported dog to other humans. These spread scenarios should not be considered exhaustive. This figure was produced to assist with conceptualization and assignment of the magnitude of impact of exposure at the human population level.

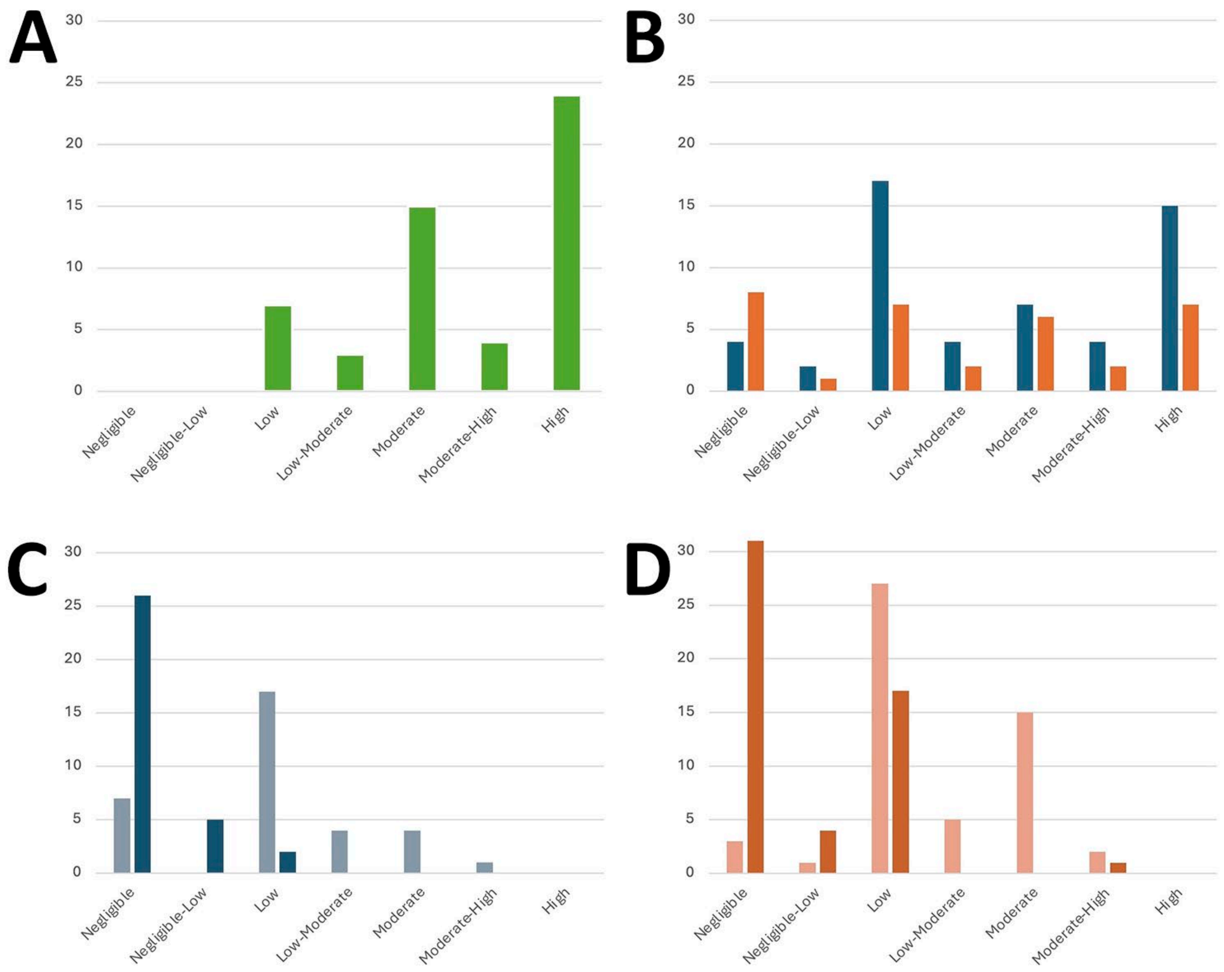

Appendix 1 Figure 8. The number of hazards in each qualitative ranking level for the estimates of: (A) likelihood of entry, (B) likelihood of canine exposure (blue bars) and likelihood of human exposure (orange bars), (C) magnitude of impact of exposure for an individual canine (light blue bars) and the canine population (dark blue bars) and (D) magnitude of impact of exposure for an individual human (light orange bars) and human population (dark orange bars). For entry and all canine estimates, 53 hazards were assessed. For estimates related to humans, only 33 hazards were assessed based on zoonotic potential.
