# Supplementary material for: Qualitative Risk Assessment of Infectious Agents Associated with Canine Importation into Canada, 2023–2024
Source: Emerg Infect Dis. 2026 Aug;32(8):1231–40. doi: 10.3201/eid3208.251602 (PMC13426852; doi:10.3201/eid3208.251602)
Supplement: Appendix 2 — Additional information used for likelihood estimates for a qualitative risk assessment of infectious agents associated with canine importation into Canada, 2023–2024 [file 25-1602-Techapp-s2.pdf]

Article DOI: https://doi.org/10.3201/eid3208.251602

EID cannot ensure accessibility for supplementary materials supplied by authors. Readers who have difficulty accessing supplementary content should contact the authors for assistance.

Qualitative Risk Assessment of Infectious Agents Associated with Canine Importation into Canada, 2023–2024

Appendix 2

Appendix 2 Table. Data compiled from a comprehensive literature search and shared with experts prior to questionnaire completion to inform the estimates for likelihood of entry, likelihood of canine exposure, and likelihood of human exposure for a qualitative risk assessment of 53 hazards potentially associated with canine importation into Canada.

HAZARD	CATEGORY	GEOGRAPHIC DISTRIBUTION	STATUS IN MAJOR IMPORTING COUNTRIES[i]	PATHOGEN DYNAMICS	OTHER INFORMATION
Bartonella vinsonii subsp. berkhoffii (Bartonellosis) UNCERTAINTY = High *More than 10 *Bartonella* spp. have been documented to infect canines as incidental hosts (1,2).	Bacteria	Suspected to have worldwide distribution (1,2). Predominately related to the distribution of the vector(s) (2). *Ctenocephalides felis* (cat flea) is confirmed, the brown dog tick (*Rhipicephalus* species complex) is suspected (3,4). High seroprevalence has been reported in warm temperate and tropic areas, as well as in free-roaming dogs (2).	**United States (EL)**: Seroprevalence is low in owned dogs; higher (~30%) in some free-roaming dogs in southeastern and western regions (1). **Ukraine (SUS):** No prevalence data in dogs was found. *Bartonella* spp. (no further differentiation) was detected in 9.1% *Ixodes ricinus* ticks and 6.2% in *Dermacentor reticulatus* ticks (5) **Mexico (EC)**: 32.2% of 31 stray dogs tested in central Mexico were seropositive for *B. vinsonii* subsp. *berkhoffii* (6). Limited information was found on the prevalence in domestic animals or humans. **Slovakia (SUS)**: No prevalence data in dogs was found. A study of small mammals detected four species of Bartonella in 64.8% animals sampled, but none were identified as *B. vinsonii* subsp. *berkhoffii* (7) **Hungary (UK):** No prevalence data in dogs was found. **South Korea (UK):** No prevalence data in dogs was found. **Poland (UK):** No prevalence data in dogs was found. **Russia (UK):** No prevalence data in dogs was found. *Bartonella* spp. infections are highly prevalent in rodents in Russia (8). **Australia (UK)**: No prevalence data in dogs was found. **Taiwan (UK)**: No prevalence data in dogs was found. Not isolated from dogs or their fleas (9).	Predominately vector-borne transmission. *Ctenocephalides felis* (cat flea) is confirmed, *R. sanguineus* (brown dog tick) is suspected (3,4). Prolonged periods of high bacteremia, as well as subclinical infection, are common (1).	*Bartonella* spp. are difficult to culture, so many studies rely on seroprevalence. Antibody cross-reactivity can also occur between species, which further complicates surveillance (1).
**Canine infectious respiratory disease (CIRD) complex agents**	Bacteria & viruses	Pathogens associated with CIRD complex have worldwide distribution (10).	**United States (EC)**: In a study, ocular and oronasal swabs from shelter dogs were tested using qPCR – 47.7% of asymptomatic dogs were positive for at least one CIRD	Transmission via direct contact with respiratory secretions (11). Incubation period is generally 2 to 10 days (11).	

HAZARD	CATEGORY	GEOGRAPHIC DISTRIBUTION	STATUS IN MAJOR IMPORTING COUNTRIES[i]	PATHOGEN DYNAMICS	OTHER INFORMATION
(Canine infectious respiratory disease)<br>UNCERTAINTY = Moderate		Proportion associated with each etiological agent are unknown as diagnostic testing is rarely completed and coinfections are common (11).<br>Highest prevalence typically in dogs housed in high densities (i.e., kennels, shelters) (12).	pathogen. *B. bronchiseptica* (19.5%), CAV-2 (12.5%), CDV (7.4%) and CPIV (3.2%) were the most prevalent (13).<br>Between 2018-2019, tonsillar, conjunctival and nasal cavity swabs were pooled from 133 client-owned asymptomatic dogs and 295 asymptomatic shelter dogs. Samples were tested with PCR - 11.3% and 40.3% of client owned dogs and shelter dogs were positive for at least one CIRD complex pathogen respectively (14).<br>**Ukraine (SUS):** No prevalence data in dogs was found.<br>**Mexico (SUS):** A study examined the lungs of 35 dogs that had died from acute or subacute pneumonia with immunohistochemistry. 77%, 57% and 51% were positive for CDV, CAD-2 and CPIV, respectively (15).<br>**Slovakia (SUS):** In a study comparing 20 dogs with CIRDC and 10 healthy dogs – 60% of the CIRDC dogs were ELISA positive for CHV-1 whereas none of the healthy dogs tested positive (16).<br>**Hungary (EC):** Of the 109 nasal and oropharyngeal swabs and serum samples sent to the Royal Veterinary College, 39.2% and 43.3% were seropositive for canine respiratory coronavirus and canine parainfluenza virus, respectively. Additionally, 7.4% and 32.4% were PCR positive for canine respiratory coronavirus and canine parainfluenza virus, respectively (17).<br>**South Korea (EC):** 300 nasal swabs were collected from dogs in the Gwangju metropolitan city animal shelter in 2019 – 49.3% were PCR positive for at least one pathogen. 31.7%, 2.3%, 2.0%, 0.7% samples were PCR positive for CHV-1, BB, CPIV, CRCOV, respectively (18).<br>**Poland (EC):** Swabs of the upper respiratory tract and tracheal lavages from 40 dogs exhibiting signs of CIRD were analyzed – 65% had coinfections of 2 or more pathogens. Canine herpesvirus had the highest prevalence (32/40), followed by canine parainfluenza virus (27/40), *Bordetella bronchiseptica* (13/40) and canine adenovirus-2 (4/40) (19).<br>**Russia (SUS):** No prevalence data in dogs was found.<br>**Australia (SUS):** Canine infectious tracheobronchitis is present in Australia, however no prevalence data in dogs was found.<br>**Taiwan (SUS):** No prevalence data in dogs was found.<br>**Other:**<br>In **Austria**, nasal and tonsillar swabs were taken from 214 dogs affected with infectious respiratory disease and 50 healthy dogs – the most common viruses detected were canine respiratory coronavirus (7.5%) and canine parainfluenza virus (6.5%) (20).<br>In 61 dogs with acute respiratory signs in **Germany**, 78.7% were PCR positive for *B. bronchiseptica*. In the 90 clinically healthy dogs also tested, prevalence was 45.6% (21).	Dogs can remain infectious for at least a month (11)<br>Subclinical infection is possible (24).	

HAZARD	CATEGORY	GEOGRAPHIC DISTRIBUTION	STATUS IN MAJOR IMPORTING COUNTRIES[i]	PATHOGEN DYNAMICS	OTHER INFORMATION
			In **New Zealand**, 94 dogs were tested for CIRD pathogens – 21% were positive for at least one pathogen by qPCR. Prevalence of *B. bronchiseptica* was 6% (22).<br>In southern **Italy**, serum samples were collected from 590 adult dogs and 100 puppies. The samples were tested for canine respiratory coronavirus using ELISA – 23.3% of all samples were positive (23).<br>Seroprevalence of canine coronavirus in domestic dogs is 59.1% in **Canada**, 54.4% in the **United States,** 36% in the **United Kingdom**, 30.3% in the R**epublic of Ireland** and 17.8% in **Japan** (23).		
Brucella canis<br>(Canine brucellosis)<br>UNCERTAINTY = High	Bacteria	Suspected to have worldwide distribution, although consistent prevalence studies are lacking (25,26).<br>Predominantly found in Central and South America, Asia and the Southern USA (27).<br>Highest seroprevalence in areas of high dog density, such as breeding kennels (26).	**United States (ER):** Most common in southern USA; seroprevalence in shelters ranged from 0% to 8.6% (28).<br>**Ukraine (SP):** The first report of canine brucellosis occurred in July 2020 (27).<br>**Mexico (EC):** In Central and South America, seroprevalence varied from 3.3% to 30.5% (including Mexico, Argentina, Brazil, Peru, Columbia) (29).<br>**Slovakia (UK):** No prevalence data in dogs was found.<br>**Hungary (SP):** The first outbreak of *B. canis* in a kennel was in 2011. *Brucella canis* was cultured from 3/31 dogs and rapid slide agglutination test was positive in 7/31 (30). Cases of *B. canis* are considered rare in Hungary (31).<br>**South Korea (ER):** A study collected 2,394 samples from companion and stray dogs in 2015-2016. 1.3% of samples were positive for *B. canis* antibodies (0.9% companion, 2.5% stray) (Jung et al., 2018). Prevalence of disease using serological and bacteriological tests in companion dogs was 2.1% in Incheon and 2.1% in Jeolla. 7.9% of stray dogs from Daejeon were positive (32).<br>**Poland (ER):** In a study screening the database of the European veterinary laboratory, 3.7% of all dog samples received from 20 European countries (including Poland) were PCR positive for *Brucella* spp. - 6.7% of these positive samples were from Poland specifically (31).<br>**Russia (UK):** No prevalence data in dogs was found.<br>**Australia (AB):** Australia and New Zealand considered free of *B. canis* (33).<br>**Taiwan (SP):** Although cases have been reported in Taiwan, no prevalence data in dogs was found.<br>Sporadic cases have been reported in Europe and Africa (33–37).	Transmission is direct via breeding or contact with bodily fluids, especially reproductive secretions. Vertical transmission also occurs (33).<br>Dogs are typically chronically (months to years) infected without clinical signs (25)	Case numbers may be underestimated in central Europe because *B. canis* is not notifiable to the OIE or to the EU (38).
Burkholderia pseudomallei<br>(Melioidosis)<br>UNCERTAINTY = High	Bacteria	Generally restricted to 25 degrees north or south of the equator, with the highest incidence reported from Southeast Asia (Northern Thailand) and Northern Australia (39).	**United States (Sp):** Non-endemic, with sporadic cases linked to travel to endemic regions (40).<br>**Ukraine (Ab):**<br>Non-endemic. No data found in dogs.	Soil and water borne, particularly moist clay soils and pooled surface water. Infection can occur through open skin contact with contaminated soil, bites of arthropod vectors, inhalation of dust or aspiration of contaminated	Considered to be an emerging disease in humans. Believed to be markedly under-reported in humans with very little

HAZARD	CATEGORY	GEOGRAPHIC DISTRIBUTION	STATUS IN MAJOR IMPORTING COUNTRIES[i]	PATHOGEN DYNAMICS	OTHER INFORMATION
			**Mexico (Sus):** Sporadic cases reported in humans. Considered possibly endemic. No data found for dogs. **Slovakia (Ab):** Non-endemic. No data found in dogs. **Hungary (Ab):** Non-endemic. No data found in dogs. **South Korea (Ab):** Rare human cases reported but linked to travel to endemic regions (41). No data found in dogs. **Poland (Ab):** Non-endemic. No data found in dogs. **Russia (Ab):** Non-endemic. No data found in dogs. **Australia (EL):** Endemic in northern Australia (42). No data found in dogs. **Taiwan (ER):** Considered endemic with cases reported in humans (43). No reported data in dogs. **Other:** Shelter dogs were sampled in Thailand (n=156) to examine potential exposure to *B. pseudomallei*. 5.77% were seropositive (44).	water, or ingestion of contaminated water, or animal products (including milk if infection occurs in the mammary gland). Bacteria can be aerosolized following heavy rains (e.g., monsoons) (39).	information available in canines (39).
Campylobacter Jejuni & C. upsaliensis (Campylo-bacteriosis) UNCERTAINTY = High	Bacteria	Worldwide distribution. Prevalence higher in dogs housed in higher density settings, as well as younger versus older animals (45).	**United States (EC):** A study conducted between 2016-2020 in pet store puppies found that 117 out of 121 human patients with *Campylobacter* had reported contact with a dog the week before symptom onset – 88% had had contact with a pet store puppy. Additionally, the study identified drug-resistant *C. jejuni* infections (46). As structured survey from 2017-2018 performed by the American Pet Products Association found that 17% of dog owners feed their pets raw human food and 3% purchase raw dog food (47). No prevalence data in dogs was found. **Ukraine (EC):** Infections with *Campylobacter* species are endemic in all European countries (48). No prevalence data in dogs was found. **Mexico (UK):** No prevalence data in dogs was found. **Slovakia (EC):** Infections with *Campylobacter* species are endemic in all European countries (48). 135 dogs in eastern Slovakia were tested via PCR, with the overall prevalence of 30.4% (*C. jejuni* - 51.2%, *C. upsaliensis* – 39% and *C. coli* - 9.8% (49). **Hungary (EC):** Infections from *Campylobacter* species are endemic in all European countries (48). No prevalence data in dogs was found. **South Korea (UK):** No prevalence data in dogs was found. 63.6% of harvest mice sampled over 2 years were positive for *C. jejuni* (50). **Poland (EC):** Infections with *Campylobacter* species are endemic in all European countries (48). A study from 2013 in Bydgoszcz recorded a *Campylobacter* prevalence of 4.81% in dogs (*C. jejuni* and *C. coli*) (51). **Russia (UK):** No prevalence data in dogs was found.	Fecal-oral transmission via direct or indirect contact with infected animals or consumption of contaminated food and water. Prolonged subclinical infection common with shedding of the bacteria in feces (45).	Surveillance and prevalence information differs greatly per country (45).

HAZARD	CATEGORY	GEOGRAPHIC DISTRIBUTION	STATUS IN MAJOR IMPORTING COUNTRIES[i]	PATHOGEN DYNAMICS	OTHER INFORMATION
			**Australia (EC):** In 1999, 34% of feral dogs carried *C. upsaliensis* and 7% carried C. jejuni (50).<br>In 2008 a study estimated roughly 5,000 human cases of campylobacteriosis annually could be attributed to contact with dogs <6 month (52).<br>**Taiwan (EC):** In 2005, 2.7% of household dogs and 23.8% of stray dogs were positive for *Campylobacter - C. jejuni was* the most prevalent comprising 86.8% of the positive cases (53).<br>**Other:**<br>Portugal: 125 rectal swabs were collected from 54 hunting dogs and 71 companion dogs. 32 tested positive, of which 44% were *C. jejuni* (54).		
Capnocytophaga canimorsus & C. cynodegmi (Capnocytophaga) UNCERTAINTY = Moderate	Bacteria	Suspected to have worldwide distribution (55).	Part of the normal bacterial flora of the oral cavity of dogs and cats (56).<br>Prevalence estimates range from 24% to >80% of dogs.<br>**United States (EC):** Samples of tooth plaque were collected from 131 dogs – 49.2% dogs carried a species of *Capnocytophaga*. Of those 21.7% carried *C. canimorsus* and 11.7% carried *C. cynodegmi* (57).<br>**Ukraine (UK):** No prevalence data in dogs was found.<br>**Mexico (UK):** No prevalence data in dogs was found.<br>**Slovakia (UK):** No prevalence data in dogs was found.<br>**Hungary (UK):** No prevalence data in dogs was found.<br>**South Korea (UK):** No prevalence data in dogs was found.<br>**Russia (UK):** No prevalence data in dogs was found.<br>**Australia (UK):** No prevalence data in dogs was found.<br>**Taiwan (EC):** Oral swabs were taken from 82 companion dogs and 16 shelter dogs. *Capnocytophaga* sp. were detected via PCR in 51% of dogs – of these *C. cynodegmi* made up 48% and *C. canimorsus* 1% (58).<br>**Other:**<br>In Japan, oral swabs taken from 325 shelter dogs in Kanagawa between 2004-2006 were tested using PCR. Prevalence of *C. canimorsus* was 74% and *C. cynodegmi* was 86% (59).<br>In Switzerland, saliva was tested via PCR from 105 dogs from Basel, Lausanne and Valis. Prevalence of *C. canimorsus* was recorded at 60% (60).<br>In Iran, oral swabs were taken from 125 dogs and the prevalence of *C. canimorsus* and *C. cynodegmi* was 50% and 90% respectively (61).<br>In 1995, oral swabs from 90 dogs were collected from a military camp in Northern France and 25.5% were *C. canimorsus* positive (62).	Considered to be commensal bacteria in the oral cavity. Dogs are long-term carriers (55).<br>Incubation period of 1-8 days (63).	
Ehrlichia canis	Bacteria	Wide distribution in warm temperate and tropic areas that coincides with distribution	**United States (EC):** Highest in the central United States (14.5%-45% seroprevalence); <5% in west, mid-west and	Tick-borne transmission (65).	Canada does not have established populations of

HAZARD	CATEGORY	GEOGRAPHIC DISTRIBUTION	STATUS IN MAJOR IMPORTING COUNTRIES[i]	PATHOGEN DYNAMICS	OTHER INFORMATION
(Canine monocytic ehrlichiosis)<br>4 genogroups have been identified: United States (US), Taiwan (TWN), Brazil (BR) and Costa Rica (CR) (64)<br>UNCERTAINTY = Moderate		of main vector, the brown dog tick (*Rhipicephalus* species complex) (65).	northeast. Out of 7,056,709 dogs tested, over 200,000 had *Ehrlichia* antibodies. Among dogs with suspected tick-borne illness, *E. ewingii* is the most prevalent (3.8%), followed by *E. chaffeensis* (3.1%) and *E. canis* (1.8%) (66).<br>**Ukraine (UK):** No prevalence data on *E. canis* in dogs was found.<br>**Mexico (EC):** Of the 1706 dogs sampled from 74 veterinary centres, 51% were seropositive for *Ehrlichia* spp. using SNAP 4DX tests (67).<br>**Slovakia (EC):** Serum samples from 78 dogs in eastern Slovakia were tested for *E. canis* using IFA and ELISA. 37.2% were IFA positive and 6.8% were ELISA positive (68).<br>**Hungary (ER):** Blood samples from 1305 healthy pet dogs from 19 counties were tested. Only 0.16% of dogs were seropositive for *E. canis* (68).<br>**South Korea (ER):** *Rhipicephalus sanguineus* is rarely found in Korea (69).<br>Of 229 hunting dogs, 6.1% were *E. canis* positive. Of 692 stray dogs, none tested positive for *E. canis* (70).<br>**Poland (ER):** 400 dogs from 23 clinics were tested between 2011-2014 using PCR and SNAP 4DX tests seroprevalence of *E. canis* was 1.5%. No dogs tested positive on PCR (71).<br>Another study showed that out of 3,094 dog samples, only 0.26% were seropositive *for Ehrlichia spp.* (72).<br>**Russia (ER):** The most common tick species in Russian cities are *Dermacentor reticulatus, Ixodes persulcatus, and I. ricinus* (73).<br>In a study testing ticks found on dogs, *E. canis* was only detected in one *R. sanguineus* tick from Sevastopol (73).<br>Australia (EC): *Rhipicephalus linnaei* is present in central and northern regions (74).<br>The first detection of *E. canis* was in May 2020 (74)<br>*Ehrlichia canis* is considered widespread in Indigenous communities (75).<br>A convenience sample of *R. linnaei* from dogs around cities and in Indigenous communities detected *E. canis* is 62.9% of ticks (76).<br>**Taiwan (EC):** The enzootic cycle of *E. canis* has been documented in dogs (77).<br>Enzootic cycles of *Ehrlichia* spp. between ticks and wild animals are widespread in Korea, Japan and southern China, all in close geographic proximity to Taiwan (77).<br>In 2009, blood samples were taken from 87 dogs presenting to veterinary hospitals throughout Taiwan with signs consistent with ehrlichiosis, of which 17 had *E. canis* morulae (78). In a study in Nantou County, 15/175 dogs and 3/306 *R. sanguineus* ticks were PCR positive for *E. canis* (79).	Main vector is the brown dog tick, (*Rhipicephalus* species complex), with transmission occurring as early as 3 hours after tick has begun feeding. No transovarial transmission occurs in the tick (65).<br>Dog can be persistent subclinical carriers for years (65).	the tick vector. Adventitial introductions are reported. Local household infestations of brown dog ticks have occurred (80).

HAZARD	CATEGORY	GEOGRAPHIC DISTRIBUTION	STATUS IN MAJOR IMPORTING COUNTRIES[i]	PATHOGEN DYNAMICS	OTHER INFORMATION
Ehrlichia ewingii<br>(Canine Ehrlichiosis)<br>UNCERTAINTY = Moderate	Bacteria	Reported predominately in the United States. Distribution mirrors the main vector, *Amblyomma americanum* (65).	**United States (EC):** Out of 7,056,709 dogs tested, over 200,000 had *Ehrlichia* antibodies. Among dogs with suspected tick-borne illness, *E. ewingii* is the most prevalent (3.8%), followed by *E. chaffeensis* (3.1%) and *E. canis* (1.8%) (66).<br>**Ukraine (AB):** *Ehrlichia ewingii* has not been detected in Europe (81).<br>**Mexico (UK):** A study evaluating tick-borne pathogens in Mexican dogs did not identify *E. ewingii* (82).<br>**Slovakia (AB):** *Ehrlichia ewingii* has not been detected in Europe (81)<br>**Hungary (AB):** *Ehrlichia ewingii* has not been detected in Europe (81).<br>**South Korea (SP):**<br>The first domestic case of *E. ewingii* occurred in 2018. *Amblyomma americanum* is not present in South Korea, but studies have shown *E. ewingii* is present in wild rodents (83).<br>**Poland (AB):** To date *E. ewingii* has not been recorded in Poland (71).<br>**Russia (UK):**<br>No prevalence data on *E. ewingii* in dogs was found.<br>**Australia (AB):**<br>*Ehrlichia ewingii* is not present in Australia.<br>**Taiwan (UK):**<br>No prevalence data on *E. ewingii* in dogs was found.<br>**Other:**<br>Outside of the USA, *E. ewingii* has been detected in dogs from Brazil and Cameroon (65).	Tick-borne transmission (65).<br>Main vector is the lone star tick, *Amblyomma americanum* (65).<br>Dogs can be subclinically infected and maintain the infection for 5 months to 2 years. White-tailed deer also have important role in maintaining bacteria (65).<br>Incubation period is 2 to 3 weeks (65).	Canada does not have established populations of the tick vector. Adventitial introductions are reported (84).
Leptospira interrogans & Leptospira kirschneri<br>(Leptospirosis)<br>Within these two species, at least 10 serovars have been associated with disease dogs including: Icterohaemorrhagiae, Canicola, Pomona, Grippotyphosa (85).<br>UNCERTAINTY = Moderate	Bacteria	Recognized as the zoonosis with the largest distribution in the world (86).<br>Endemic in many countries, particularly tropic and subtropical areas with high rainfall, and often has a seasonal distribution linked to warm temperatures and high rainfall (87).<br>Latin America, the Caribbean, the Indian subcontinent, Southeast Asia, Oceania (which includes Australasia, Melanesia, Micronesia and Polynesia) and parts of Eastern Europe are the most significant foci of disease (88).	**United States (EC):**<br>Found across the United States.<br>*Leptospira interrogans* serovars Canicola and Icterohemorrhagiae were the most common serovars in dogs in the USA prior to vaccine development (89).<br>A cross-sectional study examined 40,118 canine leptospirosis PCR tests run between 2009-2016 and recorded an overall test-positive proportion of 5.4% across the USA with the southwest having the highest prevalence (8.1%) (90).<br>**Ukraine (EC):** 2.75% of dogs were seropositive for *L. interrogans* between 2001 and 2015 in Lviv Oblast region (91).<br>**Mexico (EC):** 4.9% of stray dogs tested in southern urban areas were seropositive for *L. interrogans* (Jimenez-Coello et al., 2010).<br>400 stray dogs tested for leptospirosis in the municipality of Merida Yucatan had an overall prevalence of 35% with the most prevalent serogroups being Canicola and Icterohaemorrhagiae (92).	Dogs can be incidental hosts of any *Leptospira* strain (85).<br>The incubation period is usually 2 days to 4 weeks. Subclinical or recovered dogs may still shed viable bacteria in their urine sporadically for days or months (85).<br>Spirochetes can remain viable for months in moist soil saturated with urine (85).<br>Any age, breed or sex of dog is susceptible to infection (89).	In some areas there is seasonal influence, with incidence of infection increasing in later summer and early fall or, where winters are rainy and cold, the winter (85).<br>Vaccination is the main strategy to control disease.<br>The overall protection is roughly 84% and protection against renal carrier status is 88% (104).

HAZARD	CATEGORY	GEOGRAPHIC DISTRIBUTION	STATUS IN MAJOR IMPORTING COUNTRIES[i]	PATHOGEN DYNAMICS	OTHER INFORMATION
			**Slovakia (EC):** Of 314 domestic dogs in Eastern Slovakia tested, 12.4% had *Leptospira* antibodies with the most common serovar being Icterohaemorrhagiae (93).<br>**Hungary (SUS):** A study testing rodents from urban environments showed that 18% were carriers of various Leptospira species (94). L. pomona is recorded in dog populations in Hungary. In the neighbouring country of Serbia, overall seroprevalence from 1045 canine sera samples collected from shelter dogs was 5.45%: 33.3% icterohaemorrhagiae, 29.8% pomona, 14% canicola, 3.5% grippotyphosa, 1.7% Bataviae and 1.7% Sejroe (95).<br>**South Korea (UK):** The main reservoirs involved in human leptospirosis are wild rodents, such as Apodemus agrarius (96). No prevalence data in dogs was found<br>**Poland (EC):** Red foxes are commonly exposed to Leptospira serovars in both central and eastern Poland (97). In a study, blood samples taken from 130 dogs recorded a seroprevalence of 21.5% - with serovars Sejroe and Canicola being the most common (98). In wild animals such as foxes, brown bears and small mammals, the Saxkoebing serovar is the most prevalent (99).<br>**Russia (EC):** Between 2012-2017, 720 domestic dogs were sampled in St-Petersburg and 22.9% had specific antibodies to leptospiridae (100).<br>**Australia (EC):** In mainland Australia, the overall prevalence of canine leptospirosis obtained from sampling dog shelters was 1.9%. Throughout the country, many species such as cattle, pigs, horses, dogs, cats, possums, bandicoots, rats, mice, seals and Tasmanian devils have been found to carry *Leptospira* species (101).<br>**Taiwan (EC):**<br>45.6% of shelter dogs in the northern regions of Taiwan, were seropositive 45.6% (102).<br>7.22% of stray dogs sampled in central Taiwan between 2009 and 2011 were seropositive (103).		
Mycoplasma haemocanis<br>UNCERTAINTY= Moderate	Bacteria	Worldwide distribution, prevalence varies geographically (105).<br>In Europe, encountered in areas with Mediterranean and sub-Mediterranean climate (106).	**United States (Sp):** A study found a prevalence of 0.6% from 506 canine blood samples throughout the United States (107).<br>**Ukraine (Sp):** A study examining 52 ticks and 23 dog blood samples from Kiev found a prevalence of 4% (1/23) in dog blood samples and a prevalence of 0% in ticks (108).<br>**Mexico (Sp):** A study examining tick and flea-borne pathogens in 19 stray dogs found a prevalence of 5.3% (1/19 dogs) (109).<br>**Slovakia (Sus):** A study examining 300 red foxes in eastern Slovakia found 1 positive case of *M. haemocanis* (110).<br>No data in dogs was found.	Natural mode of transmission is uncertain. Suspected transmission is through the bite of *Rhipicephalus sanguineus* (brown dog tick) (105).<br>Once infected, *M. haemocanis* enters blood steam → attaches to surface of RBCs → multiply and colonize on RBCs. Parasitize amino acids, fatty acids, cholesterol, vitamins from RBCs → trigger immune response that causes RBC damage/death (115).	

HAZARD	CATEGORY	GEOGRAPHIC DISTRIBUTION	STATUS IN MAJOR IMPORTING COUNTRIES[i]	PATHOGEN DYNAMICS	OTHER INFORMATION
			**Hungary (ER):** A study examining 78 dogs from Hungary found 1 positive case (111). A study examining 79 confiscated fighting dogs found a prevalence of 40.5% of dogs (32/79). Likely more common in fighting dogs because of transfer of blood (112). **South Korea (EC):** A study examining 510 across South Korea found a prevalence of 0.2% (113). A study examining 532 outdoor dogs found a prevalence of 38.2% (168/532) (69). **Poland (Uk):** No data in dogs was found. **Russia (Uk):** No data in dogs was found **Australia (Sp):** A study found a prevalence of 0.8% from 251 canine blood samples from Sydney (114). **Taiwan (Sp):** A study examining 93 dogs from countries throughout southeast Asia found 2 dogs from Taiwan that tested positive for *M. haemocanis* (9).		
Rickettsia conorii* subsp. *conorii (Mediterranean Spotted Fever) UNCERTAINTY = Moderate	Bacteria	Identified in the Mediterranean Basin (e.g., southern Europe, northern Africa), the Middle East and the Indian subcontinent (116,117). Originally only thought to be present in southern Europe, Northern Africa and India – reports show it is also present in central Europe and southern Africa (118). Includes range of primary vector, the brown dog tick (*Rhipicephalus* species complex*)* (116,119).	Seroprevalence in endemic regions ranges from 15-74% (120). Bacteria can be detrimental to tick survival, so infection prevalence within ticks is generally <15% (118,121). **United States (SP):** Although brown dog ticks are endemic in the USA, MSF is not endemic in the USA. Less than 50 imported cases have been clinically or serologically identified by the CDC, however this may be due to inadequate recognition (122). **Ukraine (EC):** MSF is endemic in countries surrounding the Black Sea – Turkey, Bulgaria, Ukraine and Romania (123). No prevalence data in dogs was found. **Mexico (AB):** Not found in this region. **Slovakia (EC):** Serum from 239 humans (26 professionally exposed to domestic and wild animals and 213 suspected of having rickettsial infection) were screened for reactivity to rickettsiae and ELISA positive sera were confirmed by immunofluorescence. Of the 23.4% ELISA-positive sera, 8.9% had IgG antibodies to *R. conorii*. The same testing procedure was done on serum samples from 391 dogs (366 hunting dogs and 25 companion dogs). Out of 45 ELISA-positive sera, 12 dogs had antibodies to *R. conorii* (124). **Hungary (UK):** Although MSF is endemic in neighboring countries, no prevalence data was found in dogs **South Korea (AB):** Not found in this region. **Poland (UK):** Although MSF is endemic in neighboring countries, no prevalence data was found in dogs **Russia (AB):** Not found in this region. **Australia (AB):** Not found in this region. **Taiwan (AB):** Not found in this region. **Other:**	Transstadial transmission documented, but bacteria detrimental to tick survival (118,128). Bacteremia in dogs is low and of limited duration, so role as reservoirs is transient (118,129). The prevalence of ticks infected by *R. conorii* in the wild is low (125). Dogs are considered sentinels of *R. conorii* (130). Incubation period is typically 5-7 days (127).	Canada does not have established populations of *Rhipicephalus* spp. but they are frequently introduced via imported dogs (131,132).

HAZARD	CATEGORY	GEOGRAPHIC DISTRIBUTION	STATUS IN MAJOR IMPORTING COUNTRIES[i]	PATHOGEN DYNAMICS	OTHER INFORMATION
			Serum samples were collected from 100 dogs attending a veterinary clinic in Catalonia (Spain) in 2012-2013. The seroprevalence was 42% (125).<br>Serum samples collected from 194 dogs from different areas in Croatia were tested using IFA. 88% had antibodies for *R. conorii* (126).<br>Cases are still being recorded in new locations such as Algeria, Malta, Cyprus, Slovenia, Croatia, Kenya, Somalia and South Africa (123).<br>Seroprevalence in humans in Europe varies: Italy (3.9%), Spain (11.9%), Serbia (23%) (127).		
Microsporum canis & M. gypseum<br>(Ringworm)<br>UNCERTAINTY = Moderate	Fungus	<u>*M. canis:*</u><br>Found worldwide but is primarily in continental Europe, the eastern Mediterranean, and South America (133).<br><u>*M. gypseum:*</u><br>Circumglobal distribution (134)<br><u>*T. mentagrophytes:*</u><br>Circumglobal distribution (134)<br>In dogs, *M. canis* comprises more than 60% of dermatophyte infections reported (135).	**United States (EL):** The Louisiana State University found the prevalence of positively cultured dermatophytes to be 3.8% among canines sampled across the southern USA (136).<br>**Ukraine (EC):** In a study of 1,277 domestic dogs in Kharkiv, dermatophytosis was detected in 126 dogs (18.98%) (137).<br>**Mexico (EC):** In a study evaluating dermatophyte prevalence in humans and animals – 57/8,684 human patients (0.65%) had zoophilic dermatophytes. The most common was *M. canis* (75.5%), followed by *T. mentagrophytes* (22.9%) and *M. nanum* (1.6%). 33 of 377 (8.73%) of dogs had positive culture for *M. canis* (72.7%), *Trichophyton terrestre* (12.15%), *M. gypseum* (9.1%) and *T. mentagrophytes* (6.1%) (138).<br>**Slovakia (SUS):** A study in rabbits and guinea pigs recorded a prevalence of *T. mentagrophytes* in 6.25% and 10.68% respectively (139). Although this shows presence of dermatophytes in the country, no prevalence data in dogs was found.<br>**Hungary (SUS):** Countries reporting the highest incidence of *M. canis* are mostly in the Mediterranean but also bordering countries such as Austria, Hungary, Germany and Poland (140). No prevalence data in dogs was found.<br>**South Korea (SUS)**: In a case study from 2016, a 6-year-old girl infected with *M. canis* had a history of contact with a dog (141). Although this proves the presence of dermatophytes in Korea, no prevalence data in dogs was found<br>**Poland (SUS):**<br>*See Hungary<br>In a study evaluating dermatophytosis prevalence in 163 rodents (rabbits, guinea pigs and hamsters) , 37.4% were involved in dermatophytosis. *M. canis* was the most common isolated agent (52.7%) (140). No prevalence data in dogs was found<br>**Russia (ER):** 1,010 dogs suspected for dermatophytosis were examined. The average incidence in the Moscow region was 4.5% (142).<br>**Australia (SUS)**: No prevalence data in dogs was found.	The incubation period for dermatophytes is seven to 21 days, after which single or multiple cutaneous lesions are likely to be observed most commonly on the face, distal limbs, and tail (151).<br>Infectious spores are capable of surviving on environmental surfaces for up to 12 to 20 months under optimal conditions (i.e., warmth and humidity); therefore, transportation facilitates (i.e., cargo areas, vehicles) may also be a source of infection among imported canines (152).<br>Risk factors for the disease in dogs include psychological stress and overcrowded environments (153).	

HAZARD	CATEGORY	GEOGRAPHIC DISTRIBUTION	STATUS IN MAJOR IMPORTING COUNTRIES[i]	PATHOGEN DYNAMICS	OTHER INFORMATION
			**Taiwan (ER):** In a study examining 250 pet rabbits, 29 (11.6%) were positive for dermatophytes – 28 samples were identified as *T. mentagrophytes* and 1 sample was *M. canis* (143). Of the dogs (n= 1013) infected with *Demodex canis,* 4.4% had *M. canis* and 2.2% had *T. mentagrophyte* (144).<br>***Other:***<br>A study in the **United Kingdom** found that 10% of samples collected from dogs yielded a positive culture for dermatophytes (145).<br>In **Italy**, the reported prevalence of dermatophytosis was 7.5-20.5% in dogs (146).<br>Studies conducted in Europe indicate a higher prevalence of infection ranging from 19% to 44% among companion animals sampled (147–149).<br>Global dermatophytosis contributes to less than 4% of all skin disorders reported among dogs and cats. However, dermatophytosis is not a reportable disease, and its prevalence is likely underestimated (150).		
**Cancer cells of canine transmissible venereal tumor**<br>(Transmissible venereal tumor)<br>UNCERTAINTY = Low	Transmissible neoplasia	CTVT has been reported in all continents, except Antarctica (154).<br>Most commonly in tropical and subtropical countries, particularly in areas where large populations of stray and free-roaming dogs exist (154).<br>Uncommonly reported in North and Central Europe (i.e., the Czech Republic, Finland, the Netherlands, Sweden, Switzerland, and the United Kingdom) and North America (i.e., Canada) due to the use of control measures for stray dog populations, pre-breeding examination and effective treatment of clinical cases (155,156).<br>Cases in these regions usually associated with importation events of dogs from countries where the disease is still endemic, including Puerto Rico, Papua New Guinea, and the southwest of France (154,156).<br>It has also been reported in southeast Europe, Ireland, China, Indonesia, India, Central and South America and parts of Africa (157).	**United States (EL):** CTVT was reported to be endemic only in remote Indigenous communities – including Indian reservations in Arizona and North Dakota (155).<br>**Ukraine (UK):** No prevalence data in dogs was found.<br>**Mexico (EC):** 300 stray bitches from the municipal animal shelter in Merida, capital city of Yucatan were examined and CTVT was identified in 15.3% bitches (158).<br>**Slovakia (UK):** No prevalence data in dogs was found.<br>**Hungary (UK):** No prevalence data in dogs was found.<br>**South Korea (UK):** No prevalence data in dogs was found.<br>**Poland (UK):** No prevalence data in dogs was found.<br>**Russia (SUS):** Although CTVT was first described by a Russian veterinarian in 1870 (159), no prevalence data in dogs was found<br>**Australia (EL):** CTVT was reported to be endemic only in remote indigenous communities - Northern Territory and Western Australia (155).<br>**Taiwan (UK):** No prevalence data in dogs was found.<br>***Other:***<br>Some studies have reported the highest incidence of CTVT in dogs in **India**, ranging from 23% to 43% (160).<br>In the **Bahamas, Japan, and India,** CTVT is the most common canine tumour (156).<br>CTVT is underdiagnosed in **Colombia** due to limited literature available. The Boyacá department has a prevalence of 2.7%. At the Universidad de los Llanos, 8.6% of cancers diagnosed in dogs were identified as CTVT (161).	CTVT growths can typically be seen between two to six months after direct implantation of tumour cells, although some reports have shown they can be observed as early as 15 days after mating (160).<br>Tumour size can vary from small nodules (5 mm) to a large mass (>10 cm), most commonly located on the genitalia (157,160,162).<br>Often these tumours are ulcerated, resulting in clinical signs of the condition, such as bloody vaginal or preputial discharge, genital swelling, intermittent or persistent ulcerated skin lesions, and excessive licking of the tumour site (160).	

HAZARD	CATEGORY	GEOGRAPHIC DISTRIBUTION	STATUS IN MAJOR IMPORTING COUNTRIES[i]	PATHOGEN DYNAMICS	OTHER INFORMATION
***Alaria* spp.**<br>(Intestinal flukes)<br>UNCERTAINTY = Low	Parasite	Temperate climates across North America from Canada to Louisiana, Mexico, and South America; Europe; Middle East; and Africa (163).	**United States (Sp):** The diagnosis of *Alaria* infections is sporadic around the country, occurring primarily in temperate regions, and most cases are associated with dogs that prey on small mammals, amphibians, or reptiles (164). A study found a prevalence of 0.19% (14/ 7,409) from fecal samples of client-owned dogs submitted to the Oklahoma State University from 2007-2018 (165). A study in Oklahoma found a prevalence of 1.8% (15/837) in shelter/rescue dogs and 0.2% (9/5,417) in client-owned dogs from 2006-2014 (166).<br>**Ukraine (Sus):** A study in east Ukraine found a prevalence of 67.13% (143/213) for *Alaria alata* in wild foxes (167). No prevalence data for dogs was found.<br>**Mexico (Sp):** Two stray dogs were found to be naturally infected with *A. nasuae* in 1989 (168). *Alaria* spp. has been reported in coyotes (169).No prevalence data in dogs was found.<br>**Slovakia (Sus):** A study from 3 protected regions of east Slovakia found a prevalence of 1.6% (4/256) for *A. alata* in grey wolves (170). A study found a prevalence of 1.2% (14/1198) for *A. alata* in red foxes from the Slovak Republic (171). No prevalence data in dogs was found.<br>**Hungary (Sus):** A study from across the country found a prevalence of 49.2% (413/840) for *A. alata* in red foxes (172). A study found a prevalence of 10% (2/20) for *A. alata* in golden jackals from 2010-2012 (173). No prevalence data in dogs was found.<br>**South Korea (Sus):** Sporadic cases of *A. alata* have been reported in Korean raccoon dogs (174). No prevalence data in dogs was found<br>**Poland (Sus):** A study in northwest Poland found a prevalence of 54.7% (339/620) for *A. alata* in red foxes (175). A study from northern Poland found a prevalence of 25% (24/96) for *A. alata* in raccoon dogs (176). No prevalence data in dogs was found<br>**Russia (Sus):** A study in Yakutia, Russia found a prevalence of 31.6% for *A. alata* in red foxes (177). No prevalence data in dogs was found<br>**Australia (Ab):** No data in dogs was found.<br>**Taiwan (Ab):** No data in dogs was found.	Indirect life cycle that requires one or two intermediate hosts to reach their infective stage (178).<br>**DH**: dogs, cats, and a variety of other wild canids, felids, and small mammals including wolves, red fox, grey fox, mountain lion, striped skunk, coyote, mink, racoon, badger, wild boars, raccoon dog (163).<br>**1st IH**: freshwater snail species (e.g., *Helisoma*, *Planorbis* spp) (163).<br>**2nd IH**: frogs/tadpoles (frogs of the Ranidae family in the U.S.) (163)<br>**PH**: Several nonessential hosts such as rodents, garter snakes, feral swine, and domestic chickens (163).<br>**Lifecycle**: Adults reside in the small intestine of DH → adults lay eggs which are excreted in the feces → in aquatic environments miracidia (first larval stage) hatch from eggs → infect 1st IH (snail) → miracidia develop into sporocysts which hatch cercariae (larval stage) → cercariae leaves snail and penetrates 2nd IH (a tadpole or frog) → develop into non-reproductive mesocercariae (infective larval stage) → DH gets infected through consumption of an infected 2nd IH (tadpole or frog) OR through the consumption of an infected PH (178,179).	
Ancylostoma* spp.**<br>- *A. caninum*<br>-*A. braziliense*<br>-*A. ceylanicum*<br>Uncinaria stenocephala***<br>(Hookworms)<br>UNCERTAINTY = Low	Parasite	*A. caninum*<br>-Broadly distributed in wild and domestic canids worldwide (180). Occurs in warmer parts of the world (181).<br>*A. braziliense*<br>-Restricted to the southeastern US and coastal areas of the Atlantic, the Caribbean, eastern coastal areas of Mexico, Central and South America,	**United States (EC):** The CAPC prevalence map, showing test results for 12, 317,683 canine fecal samples from 2023, found a national prevalence of 2.40% (295,295/12, 317,683), with highest infection risks in Oklahoma, Louisiana, Mississippi, Alabama, Florida, and West Virginia. Hookworm species not specified (183). A study including 1,199,293 canine fecal samples submitted to Antech Diagnostics in 2006 found a national prevalence of 2.5% (29,556/1,199,293), with the highest prevalence in the southern region. Hookworm species not specified (184). A	**DH:**<br>-*A. caninum*: canid species (domestic dog, wolves, coyotes, foxes).<br>-*A. braziliense*: canids and felids<br>- *A ceylanicum*: canids, felids, humans<br>-*U. stenocephala*: canid species (domestic dog, wolves, coyotes, foxes)<br>**PH:** Rodents (e.g., mice). Larvae ingested by PH do not further develop in the host, but become dormant until ingested by DH (202)	

HAZARD	CATEGORY	GEOGRAPHIC DISTRIBUTION	STATUS IN MAJOR IMPORTING COUNTRIES[i]	PATHOGEN DYNAMICS	OTHER INFORMATION
		southeast Asia, and the northern coast of Australia (182).<br>*A. ceylanicum*<br>-Occurs in India, Taiwan, central Thailand, Malaysia, Indonesia, Borneo, Surinam (in South America), and likely New Guinea (182).<br>*U. stenocephala*<br>-Northern US and Canada, EU, Africa, Australia in cool-weathered regions (180,182).	study examining 148 dogs from Florida found 2 positive cases of *A. braziliense* (185).<br>**Ukraine (SUS):** A study from the East Slovak Lowland, the region near the border of Ukraine, found a prevalence of 10.09% (81/803) for *Ancylostomatidae* spp found in dog fecal samples collected from public places and dwellings (186).<br>**Mexico (EC):** A study in Mexico City examining 1603 pet dogs found 30 dogs to be positive for *Ancylostoma* spp. (prevalence = 1.9%). Authors attribute low prevalence to most pet dogs likely being on a deworming program (187). A study in Tabasco, southeast Mexico examining 302 pet dogs found that *A. caninum* had a prevalence of 15.9% (48/302) (188). A study from a rural community in Yucatan (southern Mexico) examining 130 household dogs found that *A. caninum* had a prevalence of 73.8% (96/130) (189).<br>**Slovakia (EC):** A study examining 495 dogs (pet, shelter, guard, working, and hunting) from across Slovakia found that *A. caninum* / *Uncinaria spp.* had a prevalence of 8.3% (190). A study examining 270 dogs from south-eastern Slovakia found a *U. stenocephala* prevalence of 9.6% (26/270) (191).<br>**Hungary (EC):** A study examining 490 dogs from eastern and northern Hungary found that *Ancylostomatidae* had a prevalence of 8.1-13.1% (192). A study examining 20 golden jackals from 7 towns across Hungary found a prevalence of 45% (9/20) for *A. caninum* and 40% (8/20) for *U. stenocephala* (173)<br>**South Korea (EC):** A study in 1981 found a prevalence of 8% in dogs from across South Korea (193). A study in 1981 examining 102 stray dogs from Gyonggido, Korea, found a prevalence of 26% for *A. caninum* (193). A study examining 245 dogs from 1992-1995 found an infection rate of 17.1% for *A. caninum* (193).<br>**Poland (EC):** A study examining 100 companion dogs from both rural and urban areas found a prevalence of 16% (16/100) for *Ancylostomatidae* (194).<br>A study in east-central Poland examining 3,712 dogs from 2012-2015 found a prevalence of 2.99% for *Ancylostomatidae* (111/3,712) (195).<br>**Russia (EC):** A study from Vladivostok, Russia examining 782 owned dogs found a prevalence of 6.5% (51/782) for *U. stenocephala* (196). A study from Vladivostok, Russia examining 144 household dogs found a prevalence of 6.9% (10/144) for *A. caninum* (197).<br>**Australia (EC):** A study from Queensland and New South Wales examining 201 peri-urban wild dogs found a combined prevalence of 28.8% for *A. caninum* and *U. stenocephala* (198). A study examining 285 canine fecal samples from remote communities across Australia found a prevalence of	**Lifecycle:** Adults reside and mate in the small intestine of DH → eggs released in feces → under favourable environmental conditions (moisture, warmth, shade), a 1st stage larva develops within each egg and hatches in 1-2 days → develops into 3rd stage (infective) within 5-8 days under favourable conditions (temperatures > 15C) → can survive 3-4 weeks in environment. Can be either:<br>Ingested (free from the environment or from an infected PH) → mucosal migration to GI tract<br>OR<br>Penetrate dog skin at the paw → follow a semi-tracheal migration from subcutaneous tissue →vasculature →right heart → lungs →trachea → pharynx → GI tract<br>*A. caninum* is the only species that infects neonates through transmammary transmission:<br>-In dogs >6 mo of age, larvae increasingly undergo somatic migration following ingestion/skin penetration → GI tract → portal vessels → liver → right heart → lungs → left heart → somatic tissue → larvae become dormant or arrested → larvae become activated during pregnancy → larvae accumulate in the mammary glands and are secreted in the milk → transmammary transmission (182).	

HAZARD	CATEGORY	GEOGRAPHIC DISTRIBUTION	STATUS IN MAJOR IMPORTING COUNTRIES[i]	PATHOGEN DYNAMICS	OTHER INFORMATION
			30.2% (86/285). *A. braziliense, A. ceylanicum, and U. stenocephala* were not detected (199).<br>A study examining 1400 fecal samples from across Australia found a national prevalence of 6.7% for Hookworm (200).<br>**Taiwan (EC):** Natural infections of *A. ceylanicum* have been sporadically reported in humans. From the limited data published, it appears to be present in dogs in Taiwan (201). No prevalence data in dogs was found.		
Angiostrongylus vasorum<br>(French heartworm)<br>UNCERTAINTY = Moderate	Parasite	Known to be endemic in various parts of Europe:<br>Croatia, Denmark, France, Germany, Greece, Hungary, Ireland, Italy, The Netherlands, Portugal, Spain, Sweden, Switzerland, Turkey and the United Kingdom (203).<br>Also known to be endemic in parts of Africa, South America and Canada (203).	**United States (SP):** *A. vasorum* was identified in the lungs of a red fox in West Virginia in 2011 and a few imported cases have been reported (204). No prevalence data in dogs was found.<br>**Ukraine (AB):** In a study testing Hungarian dogs for *A. vasorum*, no positive samples were detected in the regions bordering the Ukraine (205). No prevalence data in dogs was found.<br>**Mexico (UK):** No prevalence data in dogs was found.<br>**Slovakia (ER):** The first autochthonous case of *A. vasorum* was identified in 2013 in a 7-month-old Maltese (206). The prevalence of A. vasorum was recorded ~5% (207).<br>**Hungary (ER)**: Serum was collected from 1,247 dogs from various veterinary hospital – between 1 to 3% were positive (varied by test) (205).<br>**South Korea (AB):** There has been no documentation of autochthonous cases of *A. vasorum* in Asia (208).<br>**Poland (ER)**: In a study of red foxes in Augustowska Primeval Forest, 76 foxes were necropsied – 5.3% (4/76) had *A. vasorum* nematodes in their cardiopulmonary system (209). In a study testing the serum of 3,345 dogs from veterinary clinics from all over the country – 1.79% (n=60) had antibodies to *A. vasoum* and 1.29% had parasite antigens (n=43) (210).<br>**Russia (UK)**: No prevalence data in dogs was found.<br>**Australia (AB):** Not present, however cases have been identified in imported dogs (211).<br>**Taiwan (AB):** There has been no documentation of autochthonous cases of *A. vasorum* in Asia (208).<br>**Other:**<br>Endemic in Canada in Newfoundland and Labrador as well as Nova Scotia (203,212). Recently it has also been identified in the red fox population in Prince Edward Island (213). A necropsy survey of dogs close to St John's found that 10.7% (6/56) were infected (214).<br>Prior to the 1960s, known to be present in discrete pockets in the southwest regions of France (203).<br>In a study that sampled 1,748 dogs in Italy and tested them for *A. vasorum* using the Baermann method and serology, 2.1% (n=37) dogs tested positive (215).	<u>Definitive hosts:</u> various species of canids (particularly foxes, but also dogs, wolves and coyotes) (216).<br><u>Intermediate host</u>: pulmonate gastropod (slugs/snails) or frogs (216)<br><u>Cycle:</u><br>L1 larvae are ingested by intermediate host → develops into L3 larvae in intermediate host for 16-18 days → IH ingested by DH → larvae penetrate the gut wall and migrate to the pulmonary arteries and right ventricle → mature into adult worms → adults mate and produce eggs → eggs hatch into L1 larvae in the lungs → L1 larvae are coughed up, swallowed, and pass in feces (216).	Occupies the same location as canine heartworm (*Dirofilaria immitis*) (203).<br>Prepatent period is 38-57 days but can range from 28-108 (203).<br>Dogs can shed larvae for more than 5 years from a single (203).<br>In infected geographic areas, dogs kept in groups (e.g., kennels) are more likely to spread the parasite amongst themselves (216).

HAZARD	CATEGORY	GEOGRAPHIC DISTRIBUTION	STATUS IN MAJOR IMPORTING COUNTRIES[i]	PATHOGEN DYNAMICS	OTHER INFORMATION
Babesia canis* spp.**<br>-*B. vogeli*<br>-*B. rossi*<br>-*B. canis*<br>Babesia gibsoni***<br>(Canine babesiosis)<br>UNCERTAINTY = Low	Parasite	***B. vogeli***<br>-Worldwide distribution (Africa, Asia, Australia, Europe, and the Americas) (217,218).<br>Most common in humid, warm regions of the world (217,219).<br>***B. rossi***<br>-CONFINED to sub-Saharan Africa (217,218).<br>***B. canis canis***<br>Widespread in Europe and Africa (217).<br>***B. gibsoni***<br>Worldwide distribution (Africa, Asia, Australia, North and South America, Europe). Originally endemic to Asia. Was likely spread through fighting dogs in areas where competent tick vectors are not widely distributed in North America and Europe (217).	**United States (EL):**<br>*B. vogeli:*<br>A study found a prevalence of 0.31% (29/9367) in blood samples from across the US that were submitted to a diagnostic laboratory (220).<br>A study conducting an antibody serosurvey of dogs in Florida found 46% of 393 racing greyhounds were seroreactive and 0% of 50 pet dogs of various breeds were seroreactive (221).<br>*B. canis canis:*<br>A study found a prevalence of 0.19% (18/9367) in blood samples from across the US that were submitted to a diagnostic laboratory (220).<br>Disease is most often diagnosed in southern regions (217).Higher prevalence rates are commonly found in rural or suburban areas adjacent to prairies/woodlands (219).<br>*B. gibsoni:*<br>A study found a prevalence of 2% (186/9367) in blood samples from across the US that were submitted to a diagnostic laboratory (220).<br>A study including 33 Pit Bulls and 87 dogs of various other breeds from the southeastern US found positive cases for 55% of Pit Bulls (18/33) and 0 positive cases in the other breeds (222).<br>Lacks a competent vector in most of North America. Main mode of transmission is likely through dog fighting (217).<br>*B. rossi*<br>No data in dogs was found.<br>**Ukraine (EC):**<br>*B. vogeli:*<br>A study found a prevalence of 0% (0/52) in ticks collected from pet dogs in Sevastopol city (Southeastern Ukraine) (73).<br>No data in dogs was found.<br>*B. canis canis:*<br>A study found a prevalence of 26.1% (6/23) in client-owned dogs from Kiev (223). A study found a prevalence of 29% (45/155) in pet dogs from Western Ukraine (224).<br>*B. gibsoni:*<br>No data in dogs was found.<br>*B. rossi*<br>No data in dogs was found.<br>**Mexico (EC):**<br>*B. vogeli:*<br>A study found a prevalence of 10% (3/30) in dogs already showing clinical signs suggestive of babesiosis or similar disease (225).<br>*B. canis canis:*<br>No data in dogs was found.<br>*B. gibsoni:*	***B. vogeli*** -Vector: Brown dog tick (Rhipicephalus sanguineus)<br>Disease occurs throughout the year in endemic regions (217).<br>***B. canis canis:***<br>-Vector: Ornate cow tick (Dermacentor reticularis)<br>Incidence of infection is highest in the fall and spring (219).<br>***B. rossi:***<br>-Vector: Yellow dog tick (Haemaphysalis elliptica)<br>Incidence of infection is highest during the summer months (219).<br>***B. gibsoni***<br>-Vector: Haemaphysalis bispinosa and *Haemaphysalis longicornis* (in Asia) (112,219). R. sanguineus (possibly) in Africa, North America, Europe (112,219).<br>**Life cycle**<br>Mammalian stage:<br>Infected tick takes a blood meal from dog, inoculating the host with sporozoites from its saliva → sporozoites invade RBCs and multiply asexually by binary fission → the resultant merozoites rupture the RBCs and invade additional RBCs<br>It is unclear whether transformation from merozoite to gametocyte begins in the dog or tick<br>Tick stage:<br>Tick ingests blood meal from infected host → ingested gametocytes fuse to form zygote → zygote invades epithelial cells of tick mid gut and undergoes meiosis → gives rise to ookinetes → ookinetes invade either:<br>Salivary gland → ookinetes transform into sporozoites, the infective stage (transstadial transmission)<br>Or<br>Ovary (transovarial transmission) (219,234).	***B. gibsoni***<br>-Higher prevalence in fighting dog breeds due to transmission through bite wounds (217).<br>- In the US, most infections are American pit bull terriers and American Staffordshire's. This is believed to be due to fighting (217,234).<br>-Greyhounds appear to have a higher rate of exposure and infection than other breeds (219).

HAZARD	CATEGORY	GEOGRAPHIC DISTRIBUTION	STATUS IN MAJOR IMPORTING COUNTRIES[i]	PATHOGEN DYNAMICS	OTHER INFORMATION
			A study found a prevalence of 0% (0/116) using results of PCR-tested canine blood samples submitted to IDEXX laboratories (226).<br>*B. rossi*<br>No data in dogs was found.<br>**Slovakia (EC):**<br>*B. vogeli:*<br>No data in dogs was found.<br>*B. canis canis:*<br>A study found a prevalence of 20.5% (24/117) in dogs from Southern Slovakia and a prevalence of 0% in dogs from Western Slovakia (227).<br>*B. gibsoni:*<br>First clinical case was described in 2016 regarding 2 American pit bull terriers used for dogfighting (228).<br>No prevalence data in dogs was found.<br>*B. rossi*<br>No data in dogs was found.<br>**Hungary (EC):**<br>*B. vogeli:*<br>A study in Germany examining 78 stray dogs being imported from Hungary found a prevalence of 1.3% (1/78) (111).<br>*B. canis canis:*<br>A study in Germany examining 78 stray dogs being imported from Hungary found a prevalence of 50% (39/78) (111). study found a prevalence of 5.7% (37/651) in dogs from Budapest and Debrecen (229). A study found a prevalence of 5.5% (5/90) in pet dogs from Southwestern Hungary (230).<br>*B. gibsoni:*<br>A study in Germany examining 78 stray dogs being imported from Hungary found a prevalence of 1.3% (1/78) (111). In a study including 79 American Staffordshire Terrier dogs (confiscated for illegal dog fights), a prevalence of 40.5% was found (32/79) (112).<br>*B. rossi*<br>No data in dogs was found<br>**South Korea (EC):**<br>*B. vogeli:*<br>No data in dogs was found<br>*B. canis canis:*<br>No data in dogs was found<br>*B. gibsoni:*<br>A study found a prevalence of 10.6% (232/2185) using results of PCR-tested canine blood samples submitted to IDEXX laboratories (226).<br>*B. rossi*<br>No data in dogs was found.<br>**Poland (EC):**<br>*B. vogeli:*		

HAZARD	CATEGORY	GEOGRAPHIC DISTRIBUTION	STATUS IN MAJOR IMPORTING COUNTRIES[i]	PATHOGEN DYNAMICS	OTHER INFORMATION
			No data in dogs was found.<br>*B. canis canis:*<br>A study found a prevalence of 30.4% (72/237) in pet dogs from central Poland (224). A study found a prevalence of 28% (23/82) in sled dogs from central Poland (231). A study found a prevalence of 8.5% (14/165) using results of PCR-tested canine blood samples submitted to IDEXX laboratories (226).<br>*B. gibsoni:*<br>A study found a prevalence of 0% (0/165) using results of PCR-tested canine blood samples submitted to IDEXX laboratories (226).<br>*B. rossi*<br>No data in dogs was found.<br>**Russia (Sp):**<br>*B. vogeli:*<br>A study found a prevalence of 0% (0/43) in ticks collected from domestic dogs across Southern Russia (73). No data in dogs was found.<br>*B. canis canis:*<br>A study found a prevalence of 20.3% (82/404) in ticks collected from domestic dogs across Southern Russia (73). No data in dogs was found.<br>*B. gibsoni:*<br>Sporadic cases in dogs have been reported from Moscow, the Lipetsk Oblast, Ufa, St Petersburg and Oryol (73).<br>*B. rossi*<br>No data in dogs was found.<br>**Australia (ER):**<br>*B. vogeli:*<br>A study found a prevalence of 3.6% (27/740) in dogs across Australia (226).<br>A study found a prevalence of 0% (0/100) in pet dogs from southeastern Queensland (Eastern Australia) (232).<br>The same study found a prevalence of 10% (13/130) in free roaming dogs from Northern Australia (232).<br>*B. canis canis:*<br>-No data in dogs was found.<br>*B. gibsoni:*<br>Endemic in American pit bulls ONLY (232).<br>A study found a prevalence of 0.27% (2/740) using results of PCR-tested canine blood samples submitted to IDEXX laboratories (226).<br>*B. rossi*<br>No data in dogs was found.<br>**Taiwan (EC):**<br>*B. vogeli:*<br>A study found a prevalence of 9.5% (37/388) in stray/free roaming dogs across Taiwan (233).		

HAZARD	CATEGORY	GEOGRAPHIC DISTRIBUTION	STATUS IN MAJOR IMPORTING COUNTRIES[i]	PATHOGEN DYNAMICS	OTHER INFORMATION
			B. canis canis:<br>No data in dogs was found.<br>_B. gibsoni:_<br>A study found a prevalence of 15.7% (61/388) in free roaming dogs. Majority of positive cases were from Northern Taiwan (56/61) (233).<br>_B. rossi_<br>No data in dogs was found.		
Crenosoma vulpis ***Eucoleus (Capillaria) aerophilus*** (Canine Lungworm) UNCERTAINTY = Low	Parasite	_C. vulpis_<br>-Endemic in the northeastern quarter of North America and areas of temperate climate in Europe and Asia (235).<br>-The prevalence of infection in dogs is unknown for most regions (235).<br>-Reports of naturally occurring *C. vulpis* infections in the domestic dog are few and occur sporadically worldwide (236).<br>_E. aerophilus_<br>-Worldwide distribution (235).	**United States (EL):**<br>_C. vulpis_<br>-Endemic infection in dogs or wild canids has been reported in Illinois, Massachusetts, and NY (235).<br>-2 positive cases in domestic dogs were reported in New England, United States in 2021 (236).<br>-No prevalence data for dogs was found.<br>_E. aerophilus_<br>-A study found a prevalence of 0.4% from 6458 canine fecal samples (CAPC, 2018).<br>-A retrospective study found a prevalence of 0.7% in client owned cats from 2007-2018 (165).<br>**Ukraine (Sus):**<br>_C. vulpis_<br>-Has been reported in foxes, wolves, and badgers (238).<br>-No data for dogs was found.<br>_E. aerophilus_<br>-Has been reported to wolves and red foxes (238).<br>-No data for dogs was found.<br>**Mexico (Ab):**<br>_C. vulpis_<br>-No data for dogs was found.<br>_E. aerophilus_<br>-No data for dogs was found.<br>**Slovakia (Sp):**<br>_C. vulpis_<br>-a study examining 257 fecal samples from household dogs found 2 samples positive for C. *vulpis* (239).<br>-a study examining canine fecal samples from 2013-2017 found a prevalence of 0.59% (2/339) (240).<br>_E. aerophilus_<br>-a study examining 257 fecal samples from household dogs found a prevalence of 4.3% (239).<br>**Hungary (Sus)**<br>_C. vulpis_<br>-a study found a prevalence of 24.6% in red foxes (241).<br>-No data for dogs was found.<br>_E. aerophilus_<br>-a study found a prevalence of 61.7% in red foxes (241).	_C. vulpis_<br>**IH**: mollusk (multiple snail or slug species can be suitable IH)<br>**DH**: fox (natural DH), wild canids, and domestic dogs<br>**Lifecycle:**<br>- Has indirect lifecycle<br>-Adults reside free on the mucosa of the trachea, bronchi, and bronchioles of DH → females lay embryonated eggs in the bronchiole, releasing L1 larvae→ L1 ascend bronchial tree, are swallowed, and released in feces of DH → IH (snail/slug) ingests L1 → L1 mature into L3 (infective) in IH→ after ingestion of infected IH by DH, L3 reach the respiratory tree via lymphatics and blood, where they become adults (249).<br>_E. aerophilus_<br>**IH**: possibly earthworms,<br>**DH**: dogs, cats, wild carnivores, (ex. Mustelids, foxes), occasionally people<br>**Lifecycle:**<br>-Can either have a direct or indirect lifecycle<br>-Direct: Adults reside in the epithelium of bronchioles, bronchi, and trachea of DH → females release eggs that are coughed up, swallowed, and released in feces → 1st stage larvae (infective) develops in the egg in 5-6 wks → DH ingests infective larvated egg from environment → larva hatches and travels through small intestine wall, entering lymphatics or bloodstream to the lungs → enters into airways and completes development into adult<br>-Indirect: infective larvated egg is ingested by an earthworm (IH) → 1st stage larvae hatch in earthworm →DH ingests earthworm containing infective larvae → follow same path as direct lifecycle (250).<br>Prepatent period is 6 wks (250).	

HAZARD	CATEGORY	GEOGRAPHIC DISTRIBUTION	STATUS IN MAJOR IMPORTING COUNTRIES[i]	PATHOGEN DYNAMICS	OTHER INFORMATION
			-a study found a prevalence of 24.56% in 57 stray cats from hunting areas in Hungary (242).<br>-No data for dogs was found.<br>**South Korea (Sp):**<br>*C. vulpis*<br>-A dog was diagnosed with a natural infection in Daejeon, Korea in 2013. This was the first evidence of infection of a dog in Korea (243)<br>*E. aerophilus*<br>-No data for dogs was found.<br>**Poland (Sus):**<br>*C. vulpis*<br>-in the neighboring country of the Czech Republic, a prevalence of 2% in dogs was found (5/253) (244).<br>*E. aerophilus*<br>-a prevalence of 76.16% was recorded in foxes (262/344) (245).<br>-a prevalence of 2.47% was recorded in free-ranging cats (2/81) (246).<br>-No data for dogs was found.<br>**Russia (Sus):**<br>*C. vulpis*<br>-No data for dogs was found.<br>*E. aerophilus*<br>-A prevalence of 3.38% (5/148) was found in 148 domestic and stray cats (247).<br>- No data for dogs was found.<br>**Australia (Sus):**<br>*C. vulpis*<br>-No data for dogs was found.<br>*E. aerophilus*<br>-a prevalence of 10% (2/20) was found in foxes (248).<br>-a prevalence of 0.09% (1/1063) was recorded in cat feces (200).<br>-No data for dogs was found.<br>**Taiwan (Ab):**<br>*C. vulpis*<br>-No data for dogs was found.<br>*E. aerophilus*<br>-No data for dogs was found.		
Cryptosporidium canis<br>UNCERTAINTY = Low	Parasite	*Cryptosporidium spp.* in dogs have been detected worldwide (251,252).<br>A systematic review and meta-analysis examining global prevalence of *C. canis* in dogs estimated a global prevalence of 8% (253).	**United States (EC):** A study examining 129 canine fecal samples in Colorado reported a prevalence of 0.8% (1/129) (254).<br>A study examining 200 canine fecal samples in Sain Bernardino, California found a prevalence of 2% (255).<br>A study examining 49 canine fecal samples in Georgia found a prevalence of 10.2% (256).<br>**Ukraine (UK):** No data in dogs was found.	**Host**<br>Dog (252,266).<br>**Asexual reproduction**<br>Oocyte ingested by dog → Oocysts excyst and sporozoites locate in the microvilli within parasitophorous vacuoles, where they develop into trophozoites → trophozoites undergo asexual replication to form merozoites	

HAZARD	CATEGORY	GEOGRAPHIC DISTRIBUTION	STATUS IN MAJOR IMPORTING COUNTRIES[i]	PATHOGEN DYNAMICS	OTHER INFORMATION
			**Mexico (EC):** A study examining 148 canine fecal samples in Mexicali found a prevalence of 8.1% (257).<br>A study examining 183 canine fecal samples in Mexico City found a prevalence of 11.5% (258).<br>**Slovakia (EC):** A study examining 125 canine fecal samples in Eastern Slovakia found a prevalence of 7.2% in dogs > 7 months old and a prevalence of 1.4% in dogs < 7 months old (259).<br>**Hungary (Sp):** Sporadic *Cryptosporidium* spp. infections have been found in puppies (260).<br>**South Korea (EC):** A study examining 257 canine fecal samples found a prevalence of 9.7% (261).<br>A study examining 345 canine fecal samples from shelters within 6 provinces found a prevalence of 5.51% (19/345) (262)<br>**Poland (ER):** A study examining 264 canine fecal samples found a prevalence of 3% (263).<br>**Russia (ER):** A study examining 2208 canine fecal samples in Moscow from 2018-2021 found a prevalence of 2.72% (264).<br>**Australia (ER):** A study examining 1400 canine fecal samples across Australia reported a prevalence of 0.6% (200).<br>**Taiwan (EC):** A study examining 250 canine fecal samples found a prevalence of 9.6% (265).<br>**Other:** A study of young dogs (< 1 yr old) on Prince Edward Island found *C. canis* in 8% of 62 dogs from an animal shelter and 10% of 78 dogs from veterinary clinics (252).<br>- *C. canis* has been found in all Canadian provinces (252).	contained within a type I meront → merozoites are released and invade additional mucosal cells → can form additional type I meronts to continue asexual reproduction or form type II meronts to undergo sexual reproduction (252,256).<br>**Sexual reproduction**<br>Merozoites from type II meronts invade new cells and form either macrogametes or microgametes → microgametes fertilize macrogametes, producing either thick-walled or thin-walled sporulated oocytes → thick-walled oocytes are passed in feces, while thin-walled oocytes reinfect new cells (autoinfection) (252,256).	
Dioctophyma renale<br>(Giant kidney worm)<br>UNCERTAINTY= Moderate	Parasite	Temperate and subarctic areas in North and South America, Asia, and sporadically in Europe (267)	**United States (ER):** A comprehensive review found prevalence in dogs to be 0.3% (268).<br>**Ukraine (Ab):** A comprehensive review found no reports of infection in dogs in the literature (268).<br>**Mexico (Sp):** A comprehensive review found 1 case of an infected dog reported in the literature (268).<br>**Slovakia (Ab):** No data in dogs was found.<br>**Hungary (Ab):** No data in dogs was found.<br>**South Korea (Ab):** Human infection has been reported (269).<br>-no data in dogs was found.<br>**Poland (EC):** A comprehensive review found prevalence in dogs to range between 3.7% – 10.5% (268).<br>**Russia (Sp):** A comprehensive review found 39 cases of infected mammals (including dogs) reported in the literature (268).<br>**Australia (Ab):** Human infection has been reported (270).<br>- A comprehensive review found no cases of infected mammals reported in the literature (268)	**DH**: Mink (most common), dog, fox, ferret, otter, raccoon. Occasionally cats, pigs, horses, cattle (267,272).<br>**IH**: oligochaete worm (272).<br>**PH**: Numerous freshwater fish and amphibian species (272).<br>Unembryonated eggs are shed in the urine of DH → Eggs become embryonated in environment (water) → Eggs ingested by IH. Larvae develop to third stage (L3) → PH consumes IH. L3 larvae encyst in tissue → DH becomes infected after ingesting IH or PH containing L3 larvae → larvae migrate to the kidney (typically right one) → larvae become adults about 6 months after ingestion → eggs are laid in the kidney and passed to the urinary bladder (272).	

HAZARD	CATEGORY	GEOGRAPHIC DISTRIBUTION	STATUS IN MAJOR IMPORTING COUNTRIES[i]	PATHOGEN DYNAMICS	OTHER INFORMATION
			**Taiwan (Ab):** no data in dogs was found.<br>**Other:** A study found a prevalence of 7.94% in 130 dogs from northern communities in Ontario and Manitoba (271).		
Dipylidium caninum<br>(Flea tapeworm)<br>UNCERTAINTY = Low	Parasite	Worldwide distribution (273).<br>In Canada they can be found in any location where *Ctenocephalides* spp. are present – mainly in relatively warm, damp regions (274,275)<br>*Ctenocephalides*, the main intermediate host, is the most common flea on dogs/cats worldwide (274).<br>In dogs, the prevalence of disease varies between 1-60% depending on geographic location (276).<br>Human DC cases have been recorded in Europe, Philippines, China, Japan, Latin America and the USA (276).	**USA (EC):** Prevalence in the USA is highly variable, 0.29-49.5%. Dogs living in urban areas had a lower prevalence compared to dogs living in non-urban areas (0.7-5.7% vs. 1.3-13.1%) (277).<br>**Ukraine (EC):** In the territory of Poltava, the average prevalence of *Ctenocephalides* spp. on domestic dogs was 49.48% - with a *D. caninum* prevalence of 13.47% for *Ct. felis* and 11.23% for *Ct canis* (278).<br>**Mexico (EC):** In Yucatan and Queretaro, 34% and 54% of stray dogs 6 months and older were infected with *D. caninum,* respectively (276).<br>**Slovakia (ER):** In 2014, a study evaluating dog fecal samples from 8 towns and 3 villages documented the prevalence at 0.2% (279).<br>**Hungary (ER):** In a study analyzing fecal samples from dogs, prevalence was 1% (280).<br>**South Korea (Uk):** No prevalence data in dogs was found.<br>**Poland (ER):** In a study analyzing fecal samples from dogs in Chelmno between 2012 and 2014, prevalence was 5.2% (280).<br>**Russia (EC?):** Infected dogs are frequently found in various regions of Russia (281)<br>-No prevalence data in dogs was found.<br>**Australia (Sp):** The national prevalence of DC was 0.1% in 2008 (282).<br>**Taiwan (EC):** The prevalence in stray dogs in 1995 was 0.35% in central Taiwan, 63.3% in southern Taiwan, and 34% in northern Taiwan (283).<br>**Other:**<br>*D. caninum* was detected in sampled dogs at 23% in Palestine (284), 21% in Ethiopia (285), 11.8% in Pakistan (286), 5.4% in Serbia (287) and 1.67% in Indonesia (288).	Definitive Host: Domestic and wild carnivores – dogs, cats, foxes, wolves, jackals, hyenas, coyotes, racoon dogs and cheetahs (289).<br>Intermediary Host: Larval stage of dog or cat flea, occasionally the dog louse (a) Accidental hosts include humans, most frequently children (289).<br>Gravid proglottids (segment of tapeworm containing eggs) release eggs into the environment → eggs are ingested by larval stages of *Ctenocephalides* spp (dog/cat fleas) → oncosphere within the egg is released into flea's intestine, migrates into the body, and forms a cysticercoid → the cysticercoid remains until flea becomes adult → the vertebrate host ingests the infected flea → the cysticercoid develops into an adult tapeworm in 1 month → gravid proglottids are shed into the feces (290).<br>The prepatent period is 2-3 weeks (289).	
Dirofilaria immitis<br>(Canine heartworm)<br>UNCERTAINTY = Low	Parasite	Worldwide distribution (291).<br>Endemic in North, Central and South America, the Caribbean, the coastal regions of Africa, southern Europe, Asia, Japan, Indonesia and Australia (291).	**USA (EC):** Considered at least regionally endemic and has been diagnosed in all 50 states. No transmission documented in Alaska (291).<br>23 species of mosquitos known to be *D. immitis* vectors are present in the USA (291).<br>*D. immitis* is one of the most ubiquitous parasites of dogs in the USA with over 100,000 cases recorded annually. In the southeastern USA canine HW incidence rose 17.9% between 2013 and 2016 (292)<br>The prevalence in coyote populations in the western regions is greater than 90% (293). Prevalence varies in other regions with infection rates in unprotected dogs in the Gulf Coast,	Definitive Hosts:<br>Domestic dogs and wild canids (jackals, coyotes, wolves, etc.) (305).<br>Aberrant hosts: cats and ferrets – still become infected but microfilaria is usually low or absent and therefore serve as limited source of infection to mosquitos (305).<br>Mosquito takes a bloodmeal from an infected host and ingests the microfilaria → microfilaria matures into stage 3 larvae in mosquito → mosquito transmits larvae to new host during bloodmeal → larvae molts into stage 4 larvae and migrates into subcutaneous tissue →	Heartworm transmission is linked to mosquito life cycle and therefore is reduced during colder months.<br>Microenvironments present in urban areas may prevent the risk of transmission from becoming negligible (306).<br>Regularly additional species of mosquitos are shown to be able to

HAZARD	CATEGORY	GEOGRAPHIC DISTRIBUTION	STATUS IN MAJOR IMPORTING COUNTRIES[i]	PATHOGEN DYNAMICS	OTHER INFORMATION
			lower Atlantic Coast and Mississippi River being more than 90% (294).<br>**Ukraine (ER):** Between 2009 and 2019, *Dirofilaria* larvae were found in 4.6% of mosquitoes surveyed in Kharkiv (Paliy et al., 2022) Between the same time period 2.86 +/- 0.45% of blood samples collected from dogs in Kharkiv were positive for *D. immitis* (295).<br>**Mexico (EC):** In 2020, prevalence of *D. immitis* in the stray dog population in Puebla was 2.12% (296).<br>In a countrywide study, *D. immitis* prevalence in dogs was 5.3% with a higher prevalence in the northeastern region (67).<br>**Slovakia (EL):** *D. immitis* is considered endemic (297). A study conducted in 2021 surveyed 68 dogs and confirmed a *D. immitis* prevalence of 3.26% (298). The low prevalence recorded is not believed to be due to poor surveillance but from low prevalence of the parasite (298).<br>**Hungary (EC):** *D. immitis* considered endemic. First autochthonous case was detected in 2007. Until 2011, *D. immitis* infections were low and limited to a small part of the Great Hungarian Plain. The prevalence has steadily risen with *D. immitis* being recorded in 5.5% (57/1038) of dogs examined from 2015-2018 (299).<br>**South Korea (EC):** *D. immitis* is endemic. A survey conducted in 2001 recorded a prevalence of approximately 40%. In a survey conducted in 2008 the prevalence in Chungnam province was 20% (300).<br>**Poland (ER):** Recent reports have confirmed the presence of *D. immitis* in Poland. The prevalence was <1% in 2014 (301).<br>**Russia (EC):** The prevalence of *D. immitis* in dog populations throughout Russia varies from 3.4% to 47.8% (302).<br>**Australia (ER):** Although present, prevalence of *D. immitis* is considered low throughout the country (303).<br>**Taiwan (EC):** *D. immitis* was first reported in Taiwan in 1935. Prevalence in stray and pet dogs in 13 cities in Taiwan recorded in 2003 was 57% and 26.5% respectively. This did not include dogs on preventatives. The overall prevalence in 2016 was 22.8% (304).	larvae matures into juvenile worms, enters the circulatory system and migrates to the heart and pulmonary vasculature → final maturation into adult worms occurs and microfilaria are released (291).	transmit the microfilaria (307).<br>Immature worms travel the circulatory system for roughly 2 months before establishing in the heart and pulmonary arteries.<br>Female adult worms begin releasing increasing levels of detectable antigen between 210-270 days post-infection (291).<br>The number of worms is not the sole factor in determining severity of disease (306).
Echinococcosis vogeli<br>(Polycystic neotropical echinococcosis)<br>UNCERTAINTY= Moderate		Cases in humans and dogs are CONFINED to Central and South America (308–310).<br>*E. vogeli* range is limited to the range of the bush dog (natural DH), ie., from Panama in the north to Paraguay and southern Brazil in the south (308).	**United States (Ab):** No data in dogs was found.<br>**Ukraine (Ab):** No data in dogs was found.<br>**Mexico (Ab):** A study examining 54 human cases of *E. vogei* found 1 case from the nearby country of Nicaragua (311)<br>-No data in dogs was found<br>**Slovakia (Ab):** No data in dogs was found.<br>**Hungary (Ab):** A systematic review examining human Echinococcosis cases in Europe found no reports of *E. vogeli* from Hungary (312).	**IH**: Pacas (*Cuniculus paca*) (natural IH), possibly agoutis (*Dasyprocta* spp.) (308,310,314).<br>**DH**: the bush dog (*Speothos venaticus*) (natural DH), and likely domestic dogs or other canids (308,314,315)<br>Domestic dogs may be involved in cycles in endemic rural areas of South America	*E. vogeli* is a natural focal disease i.e., Parasites and their natural hosts exist independently in nature, and the involvement of domestic animals or humans is incidental (317).<br>Bush dog (DH) is a rare species in the wild (318).

HAZARD	CATEGORY	GEOGRAPHIC DISTRIBUTION	STATUS IN MAJOR IMPORTING COUNTRIES[i]	PATHOGEN DYNAMICS	OTHER INFORMATION
			-No data in dogs was found<br>**South Korea (Ab):** No data in dogs was found.<br>**Poland (Ab):** A systematic review examining human Echinococcosis cases in Europe found no reports of *E. vogeli* from Poland (312).<br>-No data in dogs was found<br>**Russia (Ab):** No data in dogs was found.<br>**Australia (Ab):** No data in dogs was found.<br>**Taiwan (Ab):** No data in dogs was found.<br>**Other:** *E. vogeli* in humans has increased within the last 10 years, going from 72 persons in 11 countries to 172 in 12 countries (Nicaragua, Costa Rica, Panama, Colombia, Ecuador, Venezuela, Surinam, Brazil, Uruguay, Argentina, Chile, Peru). Brazil had 58% of all reported cases (311)<br>-A study recorded the first record of *E. vogeli* in domestic dogs in the Arce region of Brazil in 2014 (313).	Adult parasite resides in the bush dog (DH) small intestine → Proglottid containing eggs released in feces → eggs are ingested by the paca (IH) →<br>metacestode stage develops in the liver (may also occur in other organs) → forms fluid-filled cysts in the lungs, liver, and other internal organs → bush dog consumes infected paca containing larvae in the cysts→ larvae mature in small intestine of bush dog (308,314,316).	Domestic dogs are rarely infected (308).
Echinococcus granulosus<br>(Granulomatous/ Cystic Echinococcosis)<br>Many genotypes of EG have been identified and differ host range and morphological features. The current distribution is an area of ongoing research (316).<br>Genotypes of EG (sensu lato):<br>- sensu stricto (G1-G3)<br>- *ortleppi* (G5)<br>- *canadensis* (G6,7,8 & 10) (316).<br>UNCERTAINTY = Moderate	Parasite	Found in Eurasia, the Middle East, Africa (east and north), North and South America and Australia (316,319).<br>In Europe, EG is highest in the Mediterranean countries (Italy, Spain) and Eastern countries (Romania and Bulgaria) (319).<br>China is one of the most important endemic regions for EG (320).	**USA (EL):** Some areas of the USA are considered endemic (320). A few human cases have been identified in Arizona and New Mexico in sheep farming areas. Most infections in USA are in immigrants that come from countries where EG is endemic (316). Human cases reported in Alaska are of the *E. canadensis* G8 strain in cervids (321). Sporadic cases in Indigenous populations have been recorded in Alaska, Arizona and New Mexico (320). No prevalence data in domestic dogs found.<br>**Ukraine (EL):** The parasite is established is with sheep in the southern steppe zone and the northern forest-steppe zone. *E. canadensis* and *E. granulosus* s.s. were identified in pigs in northern Ukraine (322).<br>No published data on EG infections in Ukrainian dogs (323).<br>**Mexico (SP):** A case of *E. ortleppi* infection was identified in a 38-year-old woman (324). *Echinococcus granulosus* (G1 & G7) has been found in pigs (325). No prevalence data in dogs was found.<br>**Slovakia (Ab):** In a study that collected 495 fecal samples from various dogs (working, guard, shelter, pet and hunting) between 2016 and 2021, no dogs were positive (190).<br>**Hungary (ER):** In a study looking at EG prevalence in slaughterhouses, the rate of infection was 0.013% in sheep, 0.007% in cattle and 0.001% in swine (323). Low prevalence of EG was recorded in the golden jackal population (1.7%) in southwest Hungary (326). No prevalence data in dogs was found.<br>**South Korea (SP):** Sporadic cases have been recorded in individuals that immigrated to South Korea or had recently traveled (327). No prevalence data in dogs was found.<br>**Poland (EC):** The first recorded case of EG G1 was identified in a sheep (328). CE cases in humans have been sporadic, with only 260 patients treated in the last 10 years	**Definitive host (DH)**: domestic and wild canids (dogs, foxes, wolves, coyotes, jackals), large cats, (lions, leopards) (316,320). Dogs usually DH for *E. granulosus* s.s. in the domestic cycle (314).<br>**Intermediate host (varies with genotype):**<br>*E. granulosus* sensu stricto<br>- Ungulates (sheep/goats) are the most common<br>Sheep are thought to be most important intermediate hosts, but infections have been found in many other species including goats, cattle, water buffalo, yaks, pigs, wild boar, warthogs, camelids, horses and other equids, cervids and other wild ungulates, European hares and cats (314).<br>*E. ortleppi*, (G5)<br>- Mostly cattle but also reported in water buffalo, sheep, goats, pigs, camels (314).<br>*E. canadensis*, G6<br>- Camels (314).<br>*E. canadensis*, G8, 10<br>- Cervids (314).<br>Adult tapeworm present in small intestine of definitive host → Eggs passed in feces → Ingestion by intermediate host → Penetration of intestinal wall and migration through lymph and circulatory system → Settle in organs (usually liver and lungs) and develop into thick-walled cysts → Consumption of cyst-containing organs by definitive host continues the cycle (316).	Zoonotic<br>Adult tapeworms develop in 32-80 days inside DH (316).<br>The incubation period can be as long as 20-30 years in cystic echinococcosis if the cyst is not in a critical location (314).<br>If echinococcosis is not suspected, infected animals can be missed because the eggs are morphologically indistinguishable from *Taenia* spp., and tiny proglottids are rarely noticed in feces (314).<br>Underreporting of infections in both animals and humans is common; challenging to determine true prevalence in dogs as they are rarely impacted (319).<br>Lack of reporting means imprecise mapping of EG prevalence (316).<br>Stray dogs were more likely to be infected compared to owned domestic dogs (331).

HAZARD	CATEGORY	GEOGRAPHIC DISTRIBUTION	STATUS IN MAJOR IMPORTING COUNTRIES[i]	PATHOGEN DYNAMICS	OTHER INFORMATION
			(329). The main genotype isolated in domestic dogs is *E. canadensis* (G7) (329). No prevalence data in dogs was found. **Russia (EC):** EG is widespread in all Russian territories (330). Out of 320 dogs examined in the Republic of Dagestan, 66.8% tested positive for E.G. The prevalence in North Caucasus was between 80-100% (281). **Australia (EC):** EG is prevalent in dingoes and dingo/domestic dog hybrids and has been reported in foxes (331). Prevalence in wild dogs ranges between 25-100% in Victoria and New South Wales and between 76-100% in eastern Queensland. In a study of rural dogs, 29% of 344 dogs in New South Wales and 18% of 218 Victorian dogs tested positive for EG (332). **Taiwan (SP):** Sporadic cases in individuals that immigrated or had recently traveled recorded (333). No prevalence data in dogs was found. **Other:** **Kazakhstan** recorded an EG prevalence of 19.5% in its wolves (331). EG has been recorded in jackal species and African wild dogs in **Africa** (331). **Canada** and some areas of the **USA** are considered endemic (320). In **Turkey**, fecal samples collected from 446 stray dogs between October 2015 and February 2016 were positive for E.G. (41 *E. granulosis* sensu stricto, 3 *equinus*, 1 *ortleppi* and 3 *canadensis*) (334).		Many studies have found that E.G. prevalence is higher in younger dogs – supporting hypothesis that protective immunity increases with age (331). Children are more likely to be infected due to closer contact with dogs and improper hygiene habits (335).
Echinococcus multilocularis (Alveolar echinococcosis) UNCERTAINTY = Moderate	Parasite	Emerging in parts of Europe, Asia and North America Endemic areas found in **Russia**, China, Northern Japan and North America (336). Historically endemic areas of Europe where EM was known to be present in foxes were Austria, France, Germany, Switzerland (337). **Canada**: EM is endemic in the Northern Tundra Zone and southern Alberta, Saskatchewan and Manitoba. More recently cases discovered in Ontario (338).	**United States (EL):** Commonly found in the northern central region (eastern Montana to central Ohio) and in Alaska (316). A Labrador Retriever from Virginia was PCR positive for EM in 2018 (339). No prevalence data in dogs was found. **Ukraine (SUS):** Infected foxes were identified in the westernmost regions. Possible expansion to other regions may have occurred since. No published data on EM infections in Ukrainian dogs (340). **Mexico (AB):** EM has not been reported in Mexico (341). **Slovakia (ER):** >10% prevalence in wild red foxes (342). A study on EM prevalence and genetic diversity in grey wolves showed a positive fecal sample in 1/3 of wolves sampled from central and eastern parts of the country. Prevalence in fecal samples collected from dogs was 2.7% (343). **Hungary (ER):** >10% prevalence in wild red foxes (342). Out of 3,265 foxes tested between 2008-2019, 18/19 counties identified EM, with the average prevalence at 7.6%. Golden jackals have also been identified as definitive hosts in Hungary (337). No prevalence data in dogs was found.	**Definitive host:** canids (mainly wild canids such as foxes and coyotes but domestic dogs are included) (349). **Intermediate host:** small mammals (mainly rodents) (349). **Cycle:** adult tapeworm inhabits intestinal tract of definitive host → eggs shed into environment via fecal matter → infective eggs consumed by intermediate host → larval stages produce tumor-like lesions in the liver and other organs → consumption of intermediate host by definitive host continues the cycle (349). Transmission by intermediate hosts cannot occur from casual contact (350). Shedding of eggs by dogs is uncommon, however can occur in endemic regions where prevalence is relatively high (349). Prevalence in domestic canids is correlated to prevalence in wild canids (349).	Generally considered parasite of the northern hemisphere but designation as endemic region for EM cannot be consistently attributed as there are large differences in surveillance efforts and detection capacity (351). Slow developing disease – therefore change in definitive host ecology and transmission patterns will not be reflected until years later (336). Certain countries likely grossly underestimate burden as only wealthy individuals can afford medical care. Therefore,

HAZARD	CATEGORY	GEOGRAPHIC DISTRIBUTION	STATUS IN MAJOR IMPORTING COUNTRIES[i]	PATHOGEN DYNAMICS	OTHER INFORMATION
			**South Korea (SP):** Although autochthonous cases of AE have been reported, the presence of an indigenous life cycle of EM in South Korea is unknown (344).<br>**Poland (EC):** >10% prevalence in wild red foxes (342). Prevalence in red foxes in low endemic areas in the western half of the country ranges from 2-5.7%, whereas high endemic areas range from 11.8-50% (345). No prevalence data in dogs was found.<br>**Russia (EL):** EM has a Holarctic distribution and is widespread in most parts of Russia (significant in Yakutia region) in rodents (336,346). No prevalence data in dogs was found.<br>**Australia (AB):** Not present<br>**Taiwan (AB):** Cases of human AE have been recorded but all cases were proven to be non-autochthonous (347). No prevalence data in dogs was found.<br>**Other:**<br>Highly endemic to the Qinghai province of China (348).<br>**Prevalence of EM in red foxes:**<br><u>Low ≤ 1%</u> (Denmark, Slovenia, Sweden) (342)<br><u>Medium >1% to <10%</u> (Austria, Belgium, Croatia, Hungary, Italy, Netherlands, Romania, Ukraine) (342).<br><u>High >10%</u> (Czech Republic, Estonia, France, Germany, Latvia, Lithuania, Poland, Slovakia, Liechtenstein, Switzerland) (342).<br><u>EM is absent in</u> Finland, Ireland, Malta, the United Kingdom and Norway (349).	Incubation periods is between 28-35 days (350).<br>Experimentally foxes and dogs can shed eggs into the environment for 1-4 months. Shedding is more variable in later stages (350).<br>Presentation usually only involves progressive abdominal enlargement – however, sometimes other symptoms may occur (350).<br>EM can survive for roughly 5 months in a definitive host; however, DHs can become reinfected (350).	most cases may go undiagnosed (340).
***Filaroides* spp.**<br>*-F. Hirthi*<br>*- F. osleri*<br>(Lung/Bronchial worm)<br>UNCERTAINTY = Moderate	Parasite	Infection with Filaroides species occurs sporadically, and periodically appears in some kennels or in individual dogs. It is an extremely RARE lungworm of dogs (352–354).<br>Low prevalence with worldwide distribution (352). *F. hirthi* has been reported from the USA, Canada, Europe, Australia, Israel, and Japan (235).<br>Diagnosis is relatively rare in non-research colony dogs (235).	**United States (Sp):**<br><u>*F. hirthi*</u><br>Infected client-owned dogs have been reported from Alabama, Georgia, New York, Pennsylvania, Texas, Washington (235).<br>-Has been reported in laboratory research dogs (beagles), however, there have been no reports since 1996 (235).<br>-No prevalence data in dogs was found.<br><u>*F. osleri*</u><br>- a literature search found 17 cases in domestic dogs across 9 states: NY, Kansas, Colorado, Connecticut, Pennsylvania, Texas, Georgia, Massachusetts, Wyoming (355).<br>-Infection of domestic dogs by *F. osleri* is considered uncommon. Only 26 confirmed cases have been reported in North America to date, involving nine US states and four Canadian provinces (355).<br>-No prevalence data for dogs was found.<br>**Ukraine (Ab):** No data for dogs was found.<br>**Mexico (Ab):** No data for dogs was found.	**Host**: dogs and other canids<br>Has a direct lifecycle in which 1st stage larvae are immediately infective to other dogs (354).<br>**Lifecycle**: Adults live threaded through the lung parenchyma (*F. hirthi*) or the trachea and bronchi (*F. osleri*) of dog → eggs hatch inside female worm, releasing 1st stage larvae (infective) into the tracheal lumen of host → larvae move to pharynx and are swallowed → larvae are passed in feces (352).<br>From the point of ingestion, larvae appear in feces after 5 weeks (358).<br>*F. osleri* larvae can persist within the mesenteric lymph nodes for extended periods and serve as a source of autoinfection (358).<br>Infection can be from larvae in sputum, feces, or vomitus (235). Dogs are probably commonly infected with *F. osleri* and *F. hirthi* as puppies by the transmission of larvae in sputum by the licking and cleaning of the mother or through regurgitated food (358).	The rare detection of this parasite in wild canids could be partly because *F. hirthi* is a very small, fragile nematode located in the bronchioles and alveoli and, consequently, difficult to detect by direct examination (359).

HAZARD	CATEGORY	GEOGRAPHIC DISTRIBUTION	STATUS IN MAJOR IMPORTING COUNTRIES[i]	PATHOGEN DYNAMICS	OTHER INFORMATION
			**Slovakia (Ab):** The first autochthonous infection of a dog with *F. osleri* was reported in the neighboring country of the Czech Republic in 2011 (356).<br>-No data for dogs was found.<br>**Hungary (Ab):** No data for dogs was found.<br>**South Korea (Ab):** No data for dogs was found.<br>**Poland (Ab):** No data for dogs was found.<br>**Russia (Ab):** No data for dogs was found.<br>**Australia (Sp):** Infection has previously been reported in laboratory research dogs however, there have been no reports since 1996 (235).<br>-Single isolated cases of *F. osleri* have been detected in domestic dogs from New South Wales, Queensland, and Western Australia (357).<br>-A study found a prevalence of 41.2% for *F. osleri* in dingoes/wild dogs (357).<br>**Taiwan (Ab):** No data for dogs was found.	Prepatent period of *F. hirthi* is 35 d (235).	
***Giardia duodenalis* (assembles A-D)**<br>UNCERTAINTY = Low	Parasite	Worldwide prevalence in dogs (360,361).	**United States (EC):** A study examining 16, 114 dogs with vomiting and/or diarrhea found a prevalence of 15.6% (362). A study examining *G. duodenalis* in dogs from New York City found a prevalence of 15% (363).<br>**Ukraine (Sus):** Cases in dogs have been reported in the neighboring countries of Poland and Slovakia (260,364)<br>**Mexico (EC):** A study found a prevalence of 25% (assemblage A) in 402 dogs (from shelter, breeding establishments, domestic, and stray) in the central region of Mexico (365).<br>**Slovakia (ER):** A study examining 752 canine fecal samples (pet dogs, guard dogs, shelter dogs, hunting dogs) from 8 districts of Slovakia found a prevalence of 1.6% (364).<br>**Hungary (Sus):** Cases in dogs have been reported in the neighboring country of Slovakia and nearby country of Poland (260,364)<br>**South Korea (EC):** A study found a prevalence of 15.5% (assemblages C, D) in 640 canine fecal samples from sheltered, companion, and special purpose dogs) throughout Korea (366).<br>A study found a prevalence of 11.2% in 472 dogs throughout South Korea (367).<br>**Poland (EC):** A study found a prevalence of 9.14% (assemblages A, C, D) in dogs from Warsaw (260).<br>**Russia (EC):** A study in Moscow found infection rates to be 18.2% in dogs 1-12 months old and 3.8% in dogs older than 12 months (264).<br>**Australia (EC):** A study found a prevalence of 9.3% from 1400 fecal samples from pet dogs across Australia (282).	2 life cycles:<br>-trophozoites<br>-cysts<br>Trophozoites inhabit the lumen of the small intestine → absorb nutrients, multiply by binary fission → migrate down the small intestine over the course of infection, encysting in the small or large intestine → cysts passed in feces and are immediately infectious → transmission via the fecal-oral route either by direct contact with infected host or through an infected environment (360).	

HAZARD	CATEGORY	GEOGRAPHIC DISTRIBUTION	STATUS IN MAJOR IMPORTING COUNTRIES[i]	PATHOGEN DYNAMICS	OTHER INFORMATION
			**Taiwan (EC):** A study found a prevalence of 9.3% in 118 fecal samples from stray dogs in eastern Taiwan. 5.93% were assemblage C, 3.39% were assemblage D (368).		
Hepatozoon americanum (American Canine Hepatozoonosis) UNCERTAINTY = Low	Parasite	Infections occur primarily in the south-eastern and south-central United States (369).<br>The geographic distribution of ACH aligns closely with the range of the Gulf Coast tick. It is endemic in the states that surround the Gulf of Mexico; however, its range is expanding, and it has been found in arid regions of Arizona and as far north as Kansas, Illinois, Kentucky and the Mid-Atlantic states of Virginia, Maryland, and Delaware (369).<br>Reports of *H. americanum* infection in dogs outside of the U.S. are lacking (184).	**United States (EL):** American canine hepatozoonosis has been described from the southern states including Alabama, Florida, Georgia, Louisiana, Mississippi, Oklahoma, Tennessee, and Texas (369).<br>- a study examining 614 dogs from the southeastern US with clinical signs of hepatozoonosis found a prevalence of 27.2% (167/614) (370).<br>- A study examining 274 blood samples from across the country found 24.8% (68/274) to be positive for *H. americana* and 2.6% (7/274) to have a coinfection of *H. canis* and *H. americana* (371).<br>**Ukraine (Ab):** No data for dogs was found.<br>**Mexico (Sp):** In Mexico, there are isolated reports of canine hepatozoonosis. Two cases of canine hepatozoonosis, suspected to be from *H. americanum* were recorded: one in Tamaulipas and the other in Paso del Norte Region, both border regions with Texas (372).<br>**Slovakia (Ab):** No data for dogs was found.<br>**Hungary (Ab):** No data for dogs was found.<br>**South Korea (Ab):** No data for dogs was found.<br>**Poland (Ab):** No data for dogs was found.<br>**Russia (Ab):** No data for dogs was found.<br>**Australia (Ab):** No data for dogs was found.<br>**Taiwan (Ab):** No data for dogs was found.	**IH**: domestic dogs, coyotes, and possibly other mammals<br>**DH**: *Amblyomma maculatum* (Gulf Coast tick)<br>**Lifecycle:**<br>Dog stage:<br>-dog ingests infected tick containing oocysts (either through paratenic host or grooming) → exposure of parasite to bile causes the release of sporozoites → sporozoites penetrate intestinal epithelial wall and travel to skeletal muscle within monocytic host cells → layers of mucopolysaccharide are laid down by the monocytic host cell, forming an "onion-skin cyst" → merogony occurs within the cyst → once meront is fully developed, it ruptures, releasing merozoites from the cyst → two forms of merozoites exist: circulating merozoites continue the asexual cycle by invading new cells. Other merozoites enter leukocytes and develop into gametes<br>Tick stage:<br>-tick ingests gamonts from dog leukocytes → gametogenesis and fertilization occur in tick → the resulting zygote divides through sporogony and develops into an oocyst in the hemocoel of the tick → oocysts mature while the tick molts to the nymphal or adult stage (369).	There are no established populations of the Gulf Coast tick in Canada. Sporadic introductions have been reported.
Hepatozoon canis (Old World Heptazoonosis) UNCERTAINTY = Low		Worldwide distribution including Asia, Africa, southern Europe, the Middle East, North America, South America, and Australia. Geographic distribution is closely tied to the distribution of *R. sanguineus* (369).	**United States (EL):** has been reported in dogs from southeastern states including Alabama, Georgia, Louisiana, Mississippi, Oklahoma, New Jersey, and Virginia (369).<br>-A study examining 614 dogs from the southeastern US with clinical signs of hepatozoonosis found 5% of animals had either *H. canis* infections or coinfections *of H. canis* with *H. americanum* (370).<br>-A study examining 274 blood samples from across the country found 0.7% (2/274) to be positive for *H. canis* and 2.6% (7/274) to have a coinfection *of H. canis* and *H. americana* (371).<br>**Ukraine (EC):** a study found a prevalence of 51% (27/53) in stray dogs rescued from Ukraine that were being hosted in shelters in Poland following the Russian invasion (373).<br>-a study found a prevalence of 4% (1/23) in EDTA-blood samples from client-owned dogs from Kiev (223).	**IH**: domestic dogs, some wild canids (eg. foxes, jackals, African wild dogs, coyotes)<br>**DH**: *Rhipicephalus sanguineus* (brown dog tick) and possibly other species (*Amblyomma ovale, Rhipicephalus tuanicus, R. microplus, Haemaphysalis longicornis, H. flava*) (369,375).<br>**Lifecycle:**<br>Dog stage:<br>-dog (IH) ingests infected tick containing oocysts → oocysts release sporocysts that excyst in the presence of bile, releasing infectious sporozoites into dog GI tract → sporozoites penetrate intestinal lining and travel to hemolymphatic organs (bone marrow, spleen, lymph nodes, liver, kidney, lungs) → merogony occurs in the tissue forming two types of meronts: one forms macromerozoites which create secondary meronts, continuing	There are no established populations of *R. sanguineus* in Canada, although this tick is routinely introduced via imported dogs, and can form indoor infestations (382).

HAZARD	CATEGORY	GEOGRAPHIC DISTRIBUTION	STATUS IN MAJOR IMPORTING COUNTRIES[i]	PATHOGEN DYNAMICS	OTHER INFORMATION
			**Mexico (EC):** a study from a fragmented rainforest area in southeast Mexico found a prevalence of 63.3% (19/30) in dogs (374).<br>**Slovakia (ER):** a study found a prevalence of 1% (3/293) in canine blood samples from outdoor client-owned dogs from across the country (375).<br>**Hungary (EC):** a study found a prevalence of 26% (33/126) in dogs (shepherd, hunting, and stray) from southern Hungary, an area considered to be free of *R. sanguineus* (376).<br>**South Korea (EC):** a study found a prevalence of 8.7% (16/184) in dogs from the province of Jeju-do in 2008 (377).<br>-a dog was diagnosed with *H. canis* in 2015 in Seoul (377).<br>**Poland (Sp):** A study identified 11 positive cases from 2020-2022. Dogs were either stray, shelter dogs, or from rural areas, with no known history of foreign travel, indicating the likelihood of autochthonous infection (378).<br>**Russia (Uk):** No data for dogs was found.<br>**Australia (Sp):** a study found a prevalence of 0% in a group of 230 dogs (pound dogs and aboriginal community dogs) (232).<br>-a study found a prevalence of 0% in a group of 238 client-owned dogs (379).<br>- The first report of *H. canis* infection in a dog in Australia was in 2014 (380).<br>**Taiwan (Sus):** a study examining *R. sanguineus* ticks removed from stray dogs found *H. canis* to have an infection rate of 22.8% in males, 29.4% in females, and 20.8% in nymphs (381).<br>-There has not been any clinical cases of *H. canis* recorded among dogs in Taiwan yet (381).<br>-No data for dogs was found.	the asexual cycle of merogony. The other forms micromerozoites → micromerozoites invade neutrophils and monocytes, developing into gamonts<br>Tick stage:<br>-tick (DH) ingests leukocytes containing gamonts → gametogenesis and fertilization occur in tick → zygote divides and sporogony occurs with the formation of oocysts that are released into the tick's hemocoel (369).<br>Transplacental transmission has been documented (369).	
Heterobilharzia americana (American Canine Schistosomiasis)<br>UNCERTAINTY = Low	Parasite	Endemic to the Gulf Coast and South Atlantic region of North America (383).<br>Range appears to be expanding (163).<br>Records of *Galba cubensis* and *G. humulis* (IH) exist in GenBank from Ontario and Quebec (384).<br>*G. Cubensis* was initially described from Cuba. Has also been recorded in the Antilles, the West Indies, and the south of the United States, from Florida to southern Texas. *G. cubensis* was reported in Spain in 2015. This is the first report of its occurrence in Europe (385).<br>*G. humilis* is distributed throughout North America. Records of *G. humilis* exist in GenBank from the U.S. (New Mexico, NY,	United States (EL): Majority of cases are in Texas and Louisiana.<br>-Endemic to south Atlantic and Gulf Coast states (Alabama, Florida, Georgia, North Carolina, Virginia, Mississippi, Texas, Louisiana). Has now been documented in new states including Tennessee, Oklahoma, Arkansas, Kansas, Utah (163,383,387).<br>-Was recently found in the Colorado River in southern California in *G. cubensis* and *G. humulis* (383).<br>-A study examining 30 free-roaming dogs and 81 client-owned dogs from southeastern Louisiana found a prevalence of 0.9% (1/111) (388).<br>-A study confirmed 238 cases in Texas over a period of 22 yrs (1999-2012) (389).	**DH**: Raccoons (natural DF), dogs, rabbits, opossums, bobcats, coyotes, other mammals (163,383,392).<br>**IH**: *Galba cubensis, Pseudosuccinia columella, Galba humulis* (small conical aquatic lymnaeid snails) (163).<br>**Life cycle**: Adult male and female worms reside in the mesenteric veins of DH → female releases eggs containing developed miracidia in mesenteric veins → eggs migrate through the intestinal wall and pass in feces → eggs get deposited in fresh water → larval miracidium hatch → infect IH → miracidium undergo asexual multiplication through 2 sporocyst larval stages → cercarial larvae released from IH into water → penetrate intact skin of DH →	-The limited number of reported cases in dogs appears to be incongruent with the high prevalence reported in raccoons and the broad range of naturally infected hosts. Suggests that canine infections are often not detected because routine parasitological examinations don't include the fecal sedimentation technique or owners choose euthanasia after

HAZARD	CATEGORY	GEOGRAPHIC DISTRIBUTION	STATUS IN MAJOR IMPORTING COUNTRIES[i]	PATHOGEN DYNAMICS	OTHER INFORMATION
		Tennessee, North Carolina, South Carolina, Virginia, Michigan). A 2022 study recorded its non-indigenous presence in Japan for the first time (384).<br>A literature review found reports of *P. columella* in South America (Brazil, Columbia, Venezuela, Peru, Argentina, Paraguay), Africa (South Africa, Cameroon, Mayotte, La Reunion, Namibia, Egypt, Zimbabwe, Madagascar), North America (Cuba, Guadeloupe, USA, Hawaii), Europe (Romania, Italy, France), and Oceania (Australia, French Polynesia) (386).	-A study found a prevalence of 37% (11/30) in raccoons from Kansas (390), while another study found a prevalence of 47.2% (17/36) in racoons from north-central Texas (391).<br>-Prevalence in dogs from endemic regions is not well known (392)<br>Ukraine (Ab): No data in dogs was found.<br>Mexico (Sus): A retrospective study examining *H. americana* prevalence in Texan counties found cases in counties along the Gulf of Mexico and in counties bordering Mexico (389).<br>-Populations of *G. cubenis*, a host for *H. americana* have been recorded in Mexico (383).<br>Slovakia (Ab): No data in dogs was found.<br>Hungary (Ab): No data in dogs was found.<br>South Korea (Ab): No data in dogs was found.<br>Poland (Ab): No data in dogs was found.<br>Russia (Ab): No data in dogs was found.<br>Australia (Ab): No data in dogs was found.<br>Taiwan (Ab): No data in dogs was found.	transform into schistosomula (juvenile form) → migrate to liver and develop into male and female → migrate to mesenteric veins (163,383,392).<br>Prepatent period estimated at 10 weeks (392).	diagnostic testing fails (387).
Leishmania infantum, leishmania brazielensis<br>(Leishmaniosis)<br>* Low genetic variability<br>*Other *Leishmania* species can infect dogs but the role of dogs as reservoirs is limited<br>UNCERTAINTy =<br>Low	Parasite	Endemic in at least 88 countries (393).	**United States (ER):** Common among foxhounds in kennels and hunt clubs (~8.9%) but limited in other groups domestic dogs (394).<br>*Lutzomyia* spp. sandflies present with relatively widespread distribution documented for *L. shannoni* (395).<br>**Ukraine (EC):**<br>Of 353 dogs tested between 2008 and 2012 using an ELISA, 26% were seropositive (396).<br>**Mexico (EC):**<br>In Merida Yucatan, of 218 dogs that were tested for antibodies, 11.9% were seropositive (397).<br>The primary vector is widely distributed in Mexico (398).<br>**Slovakia (SUS):** Although sand flies have been reported, no autochthonous cases of canine leishmaniasis have been published (396).<br>**Hungary (SP):**<br>Required sandfly vectors are present and the first autochthonous case was recorded in 2007. Since then, sporadic cases have been confirmed where Phlebotomus sand flies are found (396).<br>**South Korea (SUS):** The first autochthonous case was recorded in 2013 (399). Although no prevalence data was found, it is endemic in surrounding countries, with roughly 5% of dogs living in the Beijing mountain region shown to be infected with the parasite (400).<br>**Poland (AB):**<br>No species of Phlebotomus sandflies present (396).<br>**Russia (SP):** Infections have been recorded in jackals and foxes in the regions bordering Russia. The presence of Phlebotomus sand flies suggests natural transmission could	Primarily transmitted from one infected dog to another by the bite of a phlebotomine sandfly (*Lutzomyia* and *Phlebotomus* spp.). Vertical transmission as well as transmission via breeding and blood transfusion have been documented. Dog to dog contact (bites, licks) has been suspected (393).<br>Dogs remain infected and infective (to varying degrees) for life (404).<br>Not all dogs exposed develop disease. Ratio of subclinical to clinical varies by region and infecting parasite (393).<br>Incubation period ranges between 3 months and 7 years (393).<br>Clinical signs range from mild to severe (405).	Canada does not have any documented populations of phlebotomine sandflies.

HAZARD	CATEGORY	GEOGRAPHIC DISTRIBUTION	STATUS IN MAJOR IMPORTING COUNTRIES[i]	PATHOGEN DYNAMICS	OTHER INFORMATION
			occur if the parasite was introduced (401). Sporadic cases have been recorded (402).<br>**Australia (AB):** Considered disease free. All the recorded cases have been from imported dogs (403).<br>**Taiwan (SUS):** Not considered endemic. Seropositive cases have been recorded in surrounding countries (China, Vietnam, Thailand and the Philippines) (403).<br>Some other countries where the parasite is endemic include: Spain, south of France, Malta, Italy, Greece, Turkey, Israel, Egypt, Tunisia, Morocco, Iraq, Algeria, Iran, Pakistan and some areas of China (393).		
Nanophyetus salmincola & Neorickettsia helminthoeca<br>(Salon Poisoning Fluke)<br>UNCERTAINTY = Low	Parasite	Geographically restricted to coastal regions of northern California, Oregon, and Washington in the US, southern British Columbia in Canada, and eastern Russia. This reflects the distribution of *Oxytrema silicula* (snail intermediate host) (406,407).	**United States (EL):** A retrospective study examining parasitic infection in client-owned dogs presented at Oklahoma State University found a prevalence of 0.08% (6/7,409) (165).<br>-A retrospective study examining dogs presented at the University of California Veterinary Hospital found 29 positive cases of *N. salmincola* over a 19-year period (1990-2010) (408).<br>-24 cases of salmon poisoning disease associated with *N. salmincola* were reported in the Los Angelos County between 2006-2024 (409).<br>**Ukraine (Uk):** A study in eastern Ukraine found a prevalence of 0.93% (2/213) in red foxes (167).<br>-No prevalence data in dogs was found.<br>**Mexico (Ab):** No data in dogs was found.<br>**Slovakia (Ab):** No data in dogs was found.<br>**Hungary (Ab):** No data in dogs was found.<br>**South Korea (Ab):** No data in dogs was found.<br>**Poland (Ab):** No data in dogs was found.<br>**Russia (EL):** Nanophyetiasis, an infection caused by *N. salmincola,* is commonly detected in humans in the basins of the Amur River and Ussuri River and on Sakhalin Island in Russia. The prevalence of Nanophyetus infection in these areas may reach 82% among domestic carnivores and 17.6% among wild carnivores (410).<br>**Australia (Ab):** No data in dogs was found.<br>**Taiwan (Ab):** No data in dogs was found.	**DH**: fish-eating mammals including dogs and wild canids (most common), cats, wild mustelids, bears, and people (407).<br>**1st IH**: river snail (*Oxytrema silicula*)<br>**2nd IH**: salmonoids (salmon and its relatives)<br>**Lifecycle:** Adults reside in the small intestine of DH → reproduce sexually → eggs shed in feces of DH → in an aquatic environment, a miracidium (first larval stage) develops in each egg and is released into the water → miracidium penetrates the skin of 1st IH (snail) → sporocyst develops → redia develop within each sporocyst and is released into tissue when sporocyst bursts → cercariae develop within each redia, which bursts → cercariae leave the snail and penetrate the skin of 2nd IH (salmon) to develop metacercariae (infective larval stage) → ingestion of infected salmon by DH (407).	*N. salmincola* itself is considered non-pathogenic, but in North America it transmits the rickettsial pathogen *Neorickettsia helminthoeca* that is the causative agent of salmon poisoning disease (406,407).<br>Intact male dogs and Labrador retrievers appear to be overrepresented among dogs with salmon poisoning disease, possibly because they are popular with individuals engaged in fishing activities, although dogs of any sex and breed can be affected (406).<br>Occasionally, dogs from areas not endemic for salmon poisoning disease develop disease after they ingest fish that is exported from endemic areas (406).
Neospora caninum<br>(Neosporosis)<br>UNCERTAINTY = Low	Parasite	Worldwide distribution (411).<br>In North America, evidence of infection has been identified in sheep, goats, bison, black-tailed deer, caribou, moose, muskoxen, raccoons, cats and polar bears (412).<br>*N. caninum* was first discovered in Norway in dogs in 1984 (413).	**USA (EC):** In a study conducted in 1999, 7% of 1,077 dogs sampled from 35 different states had *N. caninum* antibodies (414).<br>**Ukraine (EC):** In a study conducted in 2016 looking at prevalence in red foxes, *N. caninum* DNA was detected in 18.3% of foxes from human-influenced landscapes and 9.1% of foxes from protected mountain regions (415).	Definitive host: dogs and wild canids (dingoes, wolves, coyotes, foxes) (412).<br>Intermediate hosts: cattle, white-tailed deer and other ungulates (412).<br>DHs shed oocysts in their feces → IHs ingest oocysts in contaminated food and water → tissue cysts form in nervous system and muscles of IHs → tissue cysts ingested by uninfected DHs through consumption of	Diagnosis in dogs is difficult as they only shed oocysts for a few weeks post infection (411).

HAZARD	CATEGORY	GEOGRAPHIC DISTRIBUTION	STATUS IN MAJOR IMPORTING COUNTRIES[i]	PATHOGEN DYNAMICS	OTHER INFORMATION
			**Mexico (EC):** In a study conducted in 2003, 51% of 27 Hildago farm dogs and 20% of 30 Hildago city dogs had *N. caninum* antibodies (413).<br>**Slovakia (EC):** Infections are very common in red foxes (415). In a study conducted in 2011, the seroprevalence of *N. caninum* in rural dogs was 56% while none of the urban dogs tested positive (416).<br>**Hungary (EC):** In a study conducted in 2006, 1% of 402 urban dogs had *N. caninum* antibodies (413). In another study conducted in 2006, 20% of rural dogs (n=50) had *N. caninum* antibodies (413).<br>**South Korea (EC):** In a study conducted in 2003, 21.6% of 51 dairy farm dogs had *N. caninum* antibodies (417).<br>**Poland (EC):** Infections are very common in red foxes in eastern Slovakia bordering Poland (415). In a study conducted in 2011, serum samples from 257 dogs were analyzed in the Mazovian Voivodeship in Central Poland and antibodies were detected in 21.7% (418).<br>**Russia (EC):** In a study conducted in 2000, 391 cattle were tested and 9.9% had *N. caninum* antibodies (413).<br>**Australia (EC):** In a study conducted in 1997, 150 dogs in Sydney and 94 dogs in Perth were tested for *N. caninum* and 12% and 14% of dogs had *N. caninum* antibodies, respectively (413).<br>**Taiwan (EC):** In a study conducted in 2000, 23% of 13 dairy farm dogs had *N. caninum* antibodies (413).	placenta, fetus or IH tissue infected with *N. caninum* (419).<br>The prepatent period ranges between 5-7 days (412).<br>*N. caninum* eggs are very resistant and can survive in the environment for long periods (411).	
Oncherocerca lupi<br>(Onchocerciasis)<br>UNCERTAINTY = Moderate	Parasite	Emerging zoonotic parasite in the United States (420); common in dogs in Europe and the Middle East (421).<br>Has been shown to infect dogs in Greece, Portugal, Germany, Hungary, Romania and Switzerland (421).<br>Human cases have been reported in Albania, Crimea, Iran, Tunisia and Turkey (421).	**USA (EL)**<br>- Canine cases have been reported (421). All human cases in the USA have been non-ocular (421).<br>Most infections in dogs, cats and humans have occurred in the southwestern states including New Mexico, Arizona, California, Colorado, Nevada and Utah. Cases have also been reported in Florida (422). No prevalence data in dogs was found.<br>**Ukraine (Uk):** Three cases of human ocular onchocerciasis have been reported (423). No prevalence data in dogs was found.<br>**Mexico (Uk):** No prevalence data in dogs was found.<br>**Slovakia (Sus):** The surrounding countries Austria (424) and Hungary (421) both have reported cases of *O. lupi.*<br>**Hungary (ER):** Canine cases have been reported (421). In 2001, 4 cases of onchocerciasis were reported in dogs (425).<br>**South Korea (Uk):** No prevalence data in dogs was found.<br>**Poland (Uk):** Although other *Onchocerca* species have been reported, there has been no reports of *O. lupi*<br>**Russia (Sus):** Cases have been reported in cats and dogs. The first report of *O. lupi* was in a wolf in the Republic of	Reservoir: dogs, wolves<br>Vector likely black flies (*Simuliium* spp.) and possibly biting midges (*Culicoides* spp.) (422,427).<br>Gravid females in infected host release microfilariae → microfilaria are ingested by an arthropod vector → microfilaria develop into stage 3 larvae → arthropods transfer stage 3 larvae while feeding on new host (422).<br>Currently no data available on the incubation period (422).<br>Time required for microfilaria development into stage 3 larva in arthropod vector is unknown but likely temperature dependent (422).<br>The lifespan of adult worms in dogs is estimated to be between 3 and 8 years old (425).	

HAZARD	CATEGORY	GEOGRAPHIC DISTRIBUTION	STATUS IN MAJOR IMPORTING COUNTRIES[i]	PATHOGEN DYNAMICS	OTHER INFORMATION
			Georgia bordering Russia (425). No prevalence data in dogs was found.<br>**Australia (Uk):** No prevalence data in dogs was found.<br>**Taiwan (Uk):** No prevalence data in dogs was found.<br>Other:<br>Two dogs infected with *O. lupi* were imported into Canada in 2012 and 2014 from the southwestern USA (421).<br>In a study conducted in Greece and Portugal on 107 dogs, 8% tested positive for *O. lupi* (426).<br>In 2021, Austria reported its first autochthonous case of *O. lupi* (424).		
***Opisthorchis* spp.**<br>- *O. felineus*<br>- *O. viverrini*<br>*Clonorchis sinensis*<br>(Liver Fluke)<br>UNCERTAINTY = Low	Parasite	*Opisthorchis felineus* occurs in Italy, eastern Europe, Russia, Kazakhstan, and parts of Asia (428).<br>*Opisthorchis viverrini* is confined to southeast Asia – associated with fish culture practices and food habits of this area (428).<br>*C. sinensis* is endemic in East Asia – including China, Japan, South Korea, northern Vietnam, Taiwan and parts of Russia (429).	**United States (Ab):** Liver fluke infections are rare in humans in the U.S (429).<br>-Sporadic infections of *C. sinensis* and *Opisthorchis* spp. have been reported in humans, mainly from individuals that have visited/lived in Asia (430).<br>-No data found in dogs.<br>**Ukraine (Sus):** According to the estimation based on WHO and UN data, in 2005 the infection risk was 2% in people in Ukraine for *O. felineus* (431).<br>-In Ukraine, opisthorchiasis in humans is limited to the Sumy, Poltava, and Chernigov Districts of the Dnieper River basin, where the prevalence is 5%–40% (432).<br>-No prevalence data in dogs found.<br>**Mexico (Ab):** No data in dogs was found.<br>**Slovakia (Sus):** A study found a prevalence of 0.3% (4/1198) for *O. felineus* in red foxes from the Slovak Republic (433).<br>-No data for dogs was found<br>**Hungary (Ab):** A literature review found no reports of *O. felineus* in Hungary (434).<br>**South Korea (Ab):** A nationwide positive rate of *C. sinensis* was found to be 2.4% in dogs (435).<br>-*C. sinensis* is endemic in humans in South Korea (435).<br>**Poland (Uk):** Human cases have historically been diagnosed in Poland (before 1937), however, no recent information on the occurrence of the infection in humans is available (432,436).<br>-No data in dogs was found.<br>**Russia (EC):** A study found a prevalence of 6.5% for *O. felineus* in Dagestan and 5.6% in Alatai in household dogs (281).<br>- In Russia, *C. sinensis* is mainly distributed in the southern Far East and approximately 3 000 people are estimated to be infected (437).<br>**Australia (Ab):** No data in dogs was found.<br>**Taiwan (Sus):** A study from subtropical southern China, a neighboring country, found a prevalence of 20.5% (103/503) in dogs (438).<br>-*C. sinensis* is endemic in humans in Taiwan (434).	**DH:**<br>-*O. viverrini*: dogs, cats, pigs, rodents, human, other fish-eating mammals<br>-*O. felineus*: cats, canids, mustelids, humans<br>-*C. sinensis*: dogs and wild canids, cats, mice, humans, fish-eating birds, mustelids<br>**1st IH**: several species of freshwater snail (e.g., *Parafossarulus, Thiara, Bithynia*)<br>**2nd IH**: freshwater cyprinids (eg. carp, bream, roach)<br>**Lifecycle**: Adult flukes reside in the biliary and pancreatic ducts of the DH → deposit fully developed eggs that are passed in the feces of DH → eggs ingested by 1st IH (snail) → eggs release miracidia → miracidia undergo several developmental stages (sporocysts, rediae, cercariae) → cercariae are released from snail and penetrate 2nd IH (freshwater fish) → encyst as metacerariae (infective) → ingested by DH → metacerariae excyst and develop in the biliary ducts (440).	Freshwater snail species have wide distribution. *Thiara* and *Parafossarulus* species appear to be limited to warm temperate and tropical areas. *Parafossarulus* (IH for *C. sinensis* in Asia) is widely distributed in China, Japan, Korea, and Taiwan (441).<br>Some species of Bithynia (e.g., *Bithynia tentaculate*) can be found in the Great Lakes region. The native range of *B. tentaculate* includes Europe, spanning from Scandanavia to Greece. Non-indigenous occurrences have been observed in the U.S. (DC, IL, IN, IA, MD, MI, MN, MT, NJ, NY, OH, PA, VT, VA, WI) (442).<br>A study found *O. viverrini* in 1.73% (246/14183) of *Bithynia siamensis goniomphalos* collected in North-east Thailand (443).<br>Cyprinids (IH) have the widest area of distribution of any of the freshwater fish (Africa, Asia, North America, and Europe). Southeast Asia is the center of origin for this family of fish (444).

HAZARD	CATEGORY	GEOGRAPHIC DISTRIBUTION	STATUS IN MAJOR IMPORTING COUNTRIES[i]	PATHOGEN DYNAMICS	OTHER INFORMATION
			-No data in dogs was found.<br>**Other:** In a study conducted in northern Thailand, the prevalence of *O. viverrini* infection in dogs was 3.8% and 36.4% in cats (439).		
Paragonimus kellicotti, P. westermani<br>(Lung flukes)<br>UNCERTAINTY = Moderate	Parasite	*P. kellicotti* is the only *Paragonimus* spp. that is endemic in wild and domestic animals in North America (445).<br>*P. kellicotti* may also extend into Central and South America however this is still controversial (445).	**USA (ER)**<br>- In North America, *P. kellicotti* can be found throughout the Mississippi and Great Lake waterways (446) – found within the Mississippi River basin, including central USA between the Rocky and Appalachian Mountains to the Gulf of Mexico (447).<br>**Ukraine (AB)**<br>- Endemic *Paragonimus* species have yet to be reported (445).<br>**Mexico (SUS)**<br>- No reports of *P. kellicotti* being present<br>- Based on the close proximity of the parasite to the Mexican border, it may be present in Mexico (447).<br>**Slovakia (AB)**<br>- Endemic *Paragonimus* species have yet to be reported (445).<br>**Hungary (AB)**<br>- Endemic *Paragonimus* species have yet to be reported (445).<br>**South Korea (AB)**<br>- No reports of *P. kellicotti* being present.<br>**Poland (AB)**<br>- Endemic *Paragonimus* species have yet to be reported (445).<br>**Russia (AB)**<br>- No reports of *P. kellicotti* being present.<br>**Australia (AB)**<br>- Endemic *Paragonimus* species have yet to be reported (445).<br>**Taiwan (AB)**<br>- No reports of *P. kellicotti* being present	*P. kellicotti* definitive host: Natural DH is mink, can also infect dogs, cats and people (446).<br>*P. kellicotti* intermediate Host: freshwater snails and the freshwater crayfish (446).<br>*P. kellicotti* and *P. westermani*: Eggs are passed in feces → miracidia hatch and penetrate 1st IH (freshwater snail) → mature into cercariae → cercariae invade 2nd IH (freshwater crayfish) → DHs ingest raw crayfish containing cercariae → cercariae penetrate the intestinal wall into the peritoneal cavity → cercariae pass through the diaphragm and establish in the lungs forming cysts → eggs are formed and pass through the cyst wall → eggs are coughed up and swallowed by the DH and passed in the feces (448).<br>Immature flukes form cysts in bronchioles 2-3 weeks after they reach the lungs (446).<br>Immature flukes mature into adults after 6-10 weeks (449).	
***Physaloptera* spp.**<br>UNCERTAINTY = Moderate	Parasite	Dog infections occur primarily in North America, South America, China and Africa and Australia (450,451).<br>There have only been a few reports in Europe and Africa in domestic dogs and cats (451).<br>In dogs, *P. preputialis* is distributed virtually worldwide and *P. rara* is mostly found in North America (451).<br>Prevalence in domestic dogs and cats in most studies is usually < 5% (451). Dogs usually have a prevalence of <2% (451).	**USA (ER)**<br>- Infections are common in the midwestern states (206).<br>- *P. rara* is considered the most common stomach worm in dogs in the USA (452).<br>**Ukraine (Uk)**<br>**-** No data was found on the status/prevalence in dogs<br>**Mexico (ER)**<br>**-** In a study in 2008 evaluating stray dogs in Queretaro city, only 1.9% were positive for *Physaloptera* spp. (453).<br>**Hungary (Uk)**<br>**-** No data was found for dogs<br>**South Korea (Uk)**	The life stage involves definitive, intermediate and paratenic hosts (455).<br>Larvated eggs are passed in the feces of definitive hosts → Ingestion of feces by intermediate insect host → development of 3rd stage larva inside insect IH → ingestion of infected insect by definitive host or paratenic host (450,452).<br>Ingestion by definitive host → larvae mature to adult nematodes<br>Ingestion by paratenic hosts → remain 3rd stage larvae	Dogs will rarely vomit up the parasite as it is firmly attached to the gastric mucosa<br>Fecal exams are unreliable and can often miss *Physaloptera* eggs, which likely contributes to underestimation of prevalence (452).<br>Additionally, low worm burden, fewer eggs

HAZARD	CATEGORY	GEOGRAPHIC DISTRIBUTION	STATUS IN MAJOR IMPORTING COUNTRIES[i]	PATHOGEN DYNAMICS	OTHER INFORMATION
		In wildlife species the prevalence can be higher than 50% in some areas (451).	**-** No data was found for dogs<br>**Poland (Uk)**<br>**-** No data was found on the status/prevalence in dogs<br>**Slovakia (Sus)**<br>- Endoparasites in 1198 red foxes were examined from different areas. *Physaloptera rara* was found to have a prevalence of 1.1% (433).<br>-286 birds of prey were examined. 2 birds were found to be infected with *Physaloptera alta*. This is the first record of *P. alta* in Slovakia (454).<br>- No data was found for dogs<br>**Russia (Sus)**<br>- In a study in 2017 that involved sampling soil from a dog training centre in the Perm region, traces of *Physaloptera* spp eggs were detected (Sivkova et al., 2019)<br>- No data was found for dogs<br>**Australia (Sus)**<br>- Although occurring in both dogs and cats globally, *P. praeputialis* has only been found in cats in Australia (Australian Society for Parasitology, 2015)<br>**Taiwan (Sus)**<br>**-** 95 rodents and shrews were surveyed from different areas in Taichung and a prevalence of 1.1% was recorded (Tung et al., 2009)<br>- No data was found for dogs	Adult nematodes can infect the digestive tract of amphibians, reptiles, birds and mammals (>90 species). Humans can also be infected by certain species (455).<br>Definitive hosts: various carnivorous wildlife (i.e., coyotes), as well as domestic cats and dogs (452).<br>Intermediate hosts: crickets, cockroaches, beetles, etc. (452)<br>Paratenic hosts: frogs, snakes, mice, etc. (206)<br>In Canada, the parasite has been reported in cats, badgers, bobcats, raccoons, foxes, lizards, skunks and coyotes, dogs, lynx, black bears and wolverines (450).<br>The prepatent period ranges between 2-5 months (206).	produced by females and single-sex infections can make diagnosis difficult.<br>Infection is considered uncommon, but the true prevalence in owned domestic dogs is unknown.<br>Prevalence could be decreasing with increased use of preventatives (451).
Sarcocystis spp.<br>*S. cruzi*<br>*S. capracanis*<br>*S. hircicanis*<br>*S. meisheriana*<br>*S. fayeri*<br>*S. tenella*<br>(Sarcocytosis)<br>UNCERTAINTY = Moderate	Parasite	Several species can be found in both domestic and free-ranging animals and birds worldwide, including Canada (456).<br>In humans, sarcocystosis infections have been reported with highest prevalence in Europe. Cases have also been found in Asia and in much lower frequency in Australia and North and South America (457).<br>Some species may be restricted to certain locations by distribution of their definitive and intermediate hosts (458).<br>Human reports in Africa, South and Central America and the Middle East are rarer (459).	Sarcocystosis species occur in all countries (460).<br>**USA (EC)**<br>- After consumption of beef products from a local grocery store in Maryland, 2 dogs became infected. No human infection occurred; therefore the species was suspected to be S. cruzi (459).<br>- Prevalence of *S. fayeri* in equids reported between 0.5 to 21% (461).<br>- No data on the status of *Sarcocystis* spp. in dogs<br>**Ukraine (EC)**<br>**-** No data was found on the status/prevalence in dogs<br>**Mexico (EC)**<br>- In a study evaluating digestive parasites in dogs in Aguascalientes, the prevalence of *Sarcocystis* species was 5.3% (462).<br>**Hungary (EC)**<br>- A study surveying 151 cattle found *S. cruzi* prevalence of 64% (463).<br>**South Korea (EC)**<br>**-** No data was found on the status/prevalence in dogs<br>**Poland (EC)**<br>**-** Human infections have been recorded (459).<br>**-** No data was found on the status/prevalence in dogs	Species of sarcocystis show some host specificity (456).<br>Life cycle involves a carnivore DH and an omnivore/herbivore IH (456).<br>Sporocysts in the environment are ingested by an intermediate host → Sporozoites enter endothelial cells and reproduce asexually to produce meronts containing merozoites → merozoites enters muscle cells and form sarcocysts → DH ingests sarcocysts → sexual reproduction occurs in DH intestines to produce oocysts à shedding of the oocyst wall releases sporocysts that are eliminated with stool (456).<br>Serious illness usually only occurs in the intermediate host (458).<br>***S. cruzi:***<br>- DH: Dog<br>- IH: Cattle<br>***S. capracanis***<br>- DH: Dog<br>- IH: Sheep/goats<br>***S. hiricanis***<br>- DH: Dog	Sarcocysts are very common in wildlife and in livestock raised outside high biosecurity barns (WCVM, 2021)<br>Dogs that are fed uncooked meat, used for hunting or that could scavenge wildlife are the most at risk (WCVM, 2021)<br>Sporocysts shed into feces remain infective for months to years in the environment (WCVM, 2021)<br>There is no available vaccination (Merck, 2018)

HAZARD	CATEGORY	GEOGRAPHIC DISTRIBUTION	STATUS IN MAJOR IMPORTING COUNTRIES[i]	PATHOGEN DYNAMICS	OTHER INFORMATION
			**Slovakia (EC)**<br>**-** Human infections have been recorded (459).<br>**-** No data was found on the status/prevalence in dogs<br>**Russia (EC)**<br>**-** No data was found on the status/prevalence in dogs<br>**Australia (EC)**<br>- *S. cruzi* is the most common species infecting cattle in western Australia. A study evaluating 714 cattle found an overall prevalence of 52%. More cases were found in the tropical and temperate regions (464).<br>- Human infections have been recorded (459).<br>**Taiwan (EC)**<br>- Human, cattle, and horse cases have been recorded in the neighboring country of Japan (465).<br>Minor:<br>- *Sarcocystis fayeri* infections have been identified linked to consumption of raw horsemeat in Japan (465).<br>- 354 dogs were surveyed in Brazil and the prevalence of *Sarcocystis cruzi* was 4.81% (466).	- IH: Sheep/goats<br>***S. meisheriana***<br>- DH: Dog<br>- IH: Pig<br>***S. fayeri***<br>- DH: Dogs<br>- IH: horses<br>Infective eggs start to be shed 1-2 weeks after ingestion of sarcocysts. Shedding can last for several months (467).	
Sarcoptes scabei* var *canis<br>(Scabies)<br>UNCERTAINTY = Low	Parasite	Worldwide prevalence (468).	**United States (EC):** A study found a prevalence of 16% from 310 coyotes in Illinois (469).<br>-Cases have been reported in coyotes throughout southern Texas and the mid-western USA. Cases have been reported in wolves in the Midwestern USA and the northern Rocky Mountains (470).<br>-No data in dogs was found<br>**Ukraine (Uk):** No data in dogs was found.<br>**Mexico (EC):** A study found a prevalence of 7% from 200 stray dogs from Yucatan, Mexico (471).<br>**Slovakia (EC):** A study from the Kosice region found a prevalence of 8.8% in 2241 dogs (472).<br>**Hungary (Uk):** No data in dogs was found.<br>**South Korea (EC):** A study found a prevalence of 19.4% in stray dogs (20/103) from Gwang-ju City (473).<br>A study found a prevalence of 5.66% from 565 stray dogs throughout the country (474).<br>**Poland (EC):** A study found a prevalence of 7.4% in companion dogs from southern Poland (475).<br>**Russia (Uk):** No data in dogs was found.<br>**Australia (Sus):** *S. scabiei* has been found in wombats and other Australian wildlife (476).<br>No data in dogs was found.<br>**Taiwan (Uk):** A study examining vector borne pathogens and ectoparasites in Southeast Asia found a prevalence of 0% from 132 dogs from Taiwan (477).	Life cycle takes less than 3 weeks and occurs totally on the host (478).<br>Adult mites mate in small depressions in the epidermis → females dig tunnels into the stratum corneum and lay eggs → larvae hatch from eggs → move to skin surface and make shallow superficial burrows where they feed and molt → develop into nymph, then into adults → move to the skin surface to mate (478,479).	

HAZARD	CATEGORY	GEOGRAPHIC DISTRIBUTION	STATUS IN MAJOR IMPORTING COUNTRIES[i]	PATHOGEN DYNAMICS	OTHER INFORMATION
Spirocerca lupi<br>UNCERTAINTY = Low	Parasite	Infections in canids occur in the southern US, as well as in many tropical and subtropical regions worldwide (480).<br>The most common reports are from Israel, Greece, Turkey, India, Pakistan, the southern United States, Brazil, Kenya, and South Africa (481).	**United States (EL):** While *S. lupi* is rare in canids, it has been detected in the southeastern parts of the country (480).<br>A study found that 33.5% of fecal samples from 106 rural dogs from Alabama and Mississippi, U.S.A., were infected with *S. lupi* (482).<br>A study found 3% of 142 coyotes were infected with *S. lupi.* Coyotes were from western Oklahoma to northern Texas (483).<br>**Ukraine (Sus):** *S. lupi* has been found in red foxes with a prevalence of 0.6% (238).<br>No data on dogs was found.<br>**Mexico (ER):** A study found a prevalence of 4.5% in 378 stray dogs from Queretaro, Mexico (453).<br>**Slovakia (ER):** A study examining 752 canine fecal samples (pet dogs, guard dogs, shelter dogs, hunting dogs) from 8 districts of Slovakia found a prevalence of 0.7% (364).<br>**Hungary (ER):** A study examining the prevalence of *S. lupi* in dogs presented for endoscopy from 2007-2016 found 30 positive cases and an increasing trend in cases throughout the time period (484).<br>**South Korea (Ab):** No data in dogs was found.<br>**Poland (Sus):** A study found a prevalence of 11.5% in 103 fecal samples from wild and captive wolves (485).<br>No data in dogs was found.<br>**Russia (Sp):** A study examining helminths in 1752 domestic dog fecal samples from Moscow found *S. lupi* to have a prevalence of 0.05% (486).<br>**Australia (ER):** Cases in domestic dogs have been found in Australia (487).<br>**Taiwan (Ab):** No data in dogs was found.	**IH:** Dung beetle (481).<br>**PH:** Various animals including poultry, wild birds, lizards, rodents, hedgehogs, rabbits (481).<br>**DH:** Dogs and other canids (481).<br>Adult worms reside in nodules in esophageal wall of DH → Adults lay eggs containing larvae which pass from the oesophagus to the GI tract and shed in the feces → IH ingest eggs → larvae encyst and develop into L3 larvae within 2 months → IH may be directly ingested by DH or ingested by PH and then ingested by DH → larvae penetrate stomach wall and migrate to the gastric arteries, travelling to the aorta → L3 larvae develop into L4 larvae I nodules in the aorta → L4 larvae migrate from aorta into esophageal wall.<br>Larvae can also transfer between PHs (481,488).	
Strongyloides stercoralis<br>(Strongyloidosis)<br>UNCERTAINTY = Moderate	Parasite	Widely distributed in humans throughout both tropical and subtropical areas (489).<br>In humans, it is endemic in Southeast Asia, Latin America, sub-Saharan Africa and parts of the Southern USA (490).<br>Dogs have the highest prevalence in the southeast Asia (491).<br>Human incidence has been increasing in southern, eastern, and central Europe as well as the Caribbean (490).<br>Prevalence in Canadian dogs is low. Only isolated cases have been reported in Ontario (492).	**USA (EL)**<br>- Present in rural of the south and southeastern states (493).<br>**Ukraine (EL)**<br>- The prevalence was 3.0% (n=130) in shelter dogs and 2.5% (n=234) in housed dogs in Kyiv and surrounding region (494).<br>**Mexico (EC)**<br>- In 2015, of 101 shelter dogs evaluated in Veracruz, 15.8% was positive for *S. stercoralis* (495).<br>**Slovakia (ER)**<br>- In a study conducted in eastern Slovakia, 6 of 50 dogs were positive (496).<br>**Hungary (Sp)**<br>- A 7-week-old chihuahua that came from Hungary tested positive in 2013 in Zurich (497).<br>**South Korea (Sp)**<br>- Cases have been sporadically reported<br>- Given the high prevalence in Southeast Asia, presence also very likely (491).	DH: Primarily humans, others include non-human primates and domestic dogs. It can also infect wild canids (489).<br>The parasite has two phases in its life cycle: free-living and parasitic cycles (489).<br>Free-Living cycle: Rhabditiform larvae are excreted in stool of an infected DH and larvae develop either into **filariform larvae** or **free-living adults**<br>*Free-living adults mate and produce eggs, which turn into rhabditiform larvae and mature into filariform larvae. This second generation of filariform cannot mature into adults and must find a host*<br>Parasitic Cycle: Filariform larvae can penetrate a host's skin and larvae migrate to the small intestine through various pathways. The larvae molt twice to become adult females. Females	Due to autoinfection, if left untreated, infection may persist for decades even in a non-endemic area (489).<br>Transmission is more often reported during summer months in temperate areas (489).<br>Infections are most commonly seen in rural or remote areas, places with poor sanitation, institutions, and among socially marginalized groups (489).<br>May be underdiagnosed as it is not usually detected

HAZARD	CATEGORY	GEOGRAPHIC DISTRIBUTION	STATUS IN MAJOR IMPORTING COUNTRIES[i]	PATHOGEN DYNAMICS	OTHER INFORMATION
			**Poland (Sp)**<br>- No data from dogs.<br>- In a study in the Dabrowa Bialostocka District, 7.78% of 120 hospitalized children were positive for *S. stercoralis* (498).<br>**Russia (Sp)**<br>- Both human and canine *S. stercoralis* have been identified in Russia (281).<br>**Australia (EC)**<br>- The prevalence in dogs living across the Northern Territory was 21.9% (199).<br>**Taiwan (Sp)**<br>- The current prevalence of *S. stercoralis* is unknown but Southeast Asia thought to have the highest burden globally (491).	embed in submucosa of intestinal wall and shed eggs (489).<br>Migration to the small intestines takes 4-5 days and the prepatent period can range from 5 to 21 days (499).<br>Egg shedding capacity reaches its maximum at 20 days post infection. Shedding can continue at a lower level for up to 11 weeks (499).<br>*Auto-infection may occur as the eggs hatch internally and produce rhabditiform larvae*	on routine fecal flotation (492).<br>True prevalence underestimated due to lack of precise surveillance data in endemic areas.<br>Infections occur more commonly in canines when they are stray.
Taenia spp.<br>*T. hydatigena*<br>*T. taeniaeformis*<br>*T. multiceps*<br>*T. crassiceps*<br>*T. serialis*<br>*T. pisiformis*<br>(Taeniasis)<br>UNCERTAINTY = Low	Parasite	*Taenia spp. a*re found globally and infect all age groups of dogs (165).<br>*Taenia* spp. are commonly found in dogs throughout North America (500).	**USA (EC):** A study based in Oklahoma found a prevalence of 7.2% for shelter dogs infected with *Taenia spp*. (501).<br>-A retrospective study found a prevalence of 0.47% for household dogs having *Taenia* proglottids or eggs in their feces in North America (165).<br>**Ukraine (Sus):** There have been cases of *T. taeniaeformis, T. crassiceps, T. hydatigena, T. pisiformis* reported in wild Canidae (502).<br>- No data was found on the prevalence in dogs<br>**Mexico (EC):** A study in the northwest area found *Taenia spp* to have a prevalence of 3.9% in stray dogs (503).<br>A study in the farming and cattle region of Mexico determined *Taenia spp* prevalence to be 6.79% in stray dogs (503).<br>**Slovakia (ER):** A study found *Taenia hydatigena* to have a prevalence of 0.4% in dogs (including shelter, guard, hunting, and pets) (190).<br>**Hungary (ER):** In a study of 490 canine fecal samples, *Taenia* eggs were found in 8 dogs from the eastern region and 5 dogs from the northern region (192).<br>-A study in Serbia, a neighboring country, found *Taenia spp* prevalence in stray, household, and military dog fecal samples to have a prevalence of 6.6% (504).<br>**South Korea (Sus):** In 1981, *T. pisiformis* was found to have a prevalence of 9% in stray dogs *in* Ejungbu City (193).<br>**Poland (ER):** A study in the Western Pomerania area found the prevalence of *Taenia* spp in dog fecal samples to be 3.45% (505).<br>**Russia (ER):** A study based in Vladivostock city determined the prevalence of *Taenia spp* in dogs to be 2.1% (506).<br>**Australia (ER):** A study found *Taenia spp* prevalence (including *T. pisiformis and T. serialis*) to be 4.5% in peri-urban wild dogs (198).<br>**Taiwan (UK):** No data was found on the status/prevalence in dogs.	Definitive host: dog, cat<br>Intermediate host: mouse, rabbit, deer, sheep etc.<br>Segments containing eggs mature in the DH intestine → segments released through feces → segments ingested by IHà tapeworm develops in abdomen of IH for 2 months → IH is consumed by DH, resulting in the consumption of the tapeworm → tapeworm finishes development in DH → segments mature (507,508).	Prevalence data generated by fecal flotation alone almost certainly underestimate the frequency of infection because proglottids (and thus eggs) are focally distributed in fecal material (500).

HAZARD	CATEGORY	GEOGRAPHIC DISTRIBUTION	STATUS IN MAJOR IMPORTING COUNTRIES[i]	PATHOGEN DYNAMICS	OTHER INFORMATION
Toxocara canis<br>(Toxocariasis)<br>UNCERTAINTY = Low	Parasite	Cases of toxocariasis are circumglobally distributed. Most prevalent in tropical and subtropical regions where the eggs can survive better in the environment (509,510).<br>Infections are often reported in regions with large populations of domestic dogs (509).<br>A systematic review and meta-analysis study found the overall prevalence of *Toxocara* infection in dogs was 11.1% worldwide, ranging from 6.4% to 19.2% across different regions (511).<br>The pooled prevalence of *Toxocara* infection in dogs was highest in the Eastern Mediterranean region (19.2%), including studies Iran (15.7%)<br>Egypt (14.0%)<br>Iraq (43.9%)<br>Pakistan (37.0%)<br>Jordan (1.2%)<br>Tunisia (34.4%) (511).<br>Prevalence in dogs was lowest in the Western Pacific region (6.4%)<br>Japan (2.0%)<br>Australia (3.7%)<br>China (39.1%)<br>Cambodia (6.9%)<br>Malaysia (7.4%)<br>Philippines (11.5%)<br>Vietnam (37.7%)<br>Taiwan (1.1%) (511).<br>Pooled prevalence of *Toxocara* infection in dogs in other regions:<br>Africa (18.0%)<br>South-East Asia (11.9%)<br>North America (11.1%)<br>South America (10.9%)<br>Europe (10.8%) (511).	**USA (EC)**<br>- The national prevalence of *T. canis* eggs in dogs feces between 2011 and 2014 was 1.8-2.0% (512).<br>**Ukraine (EC)**<br>- Based on studies conducted in the Kharkiv region, examination of various animal fecal samples from a rural site revealed 100% were contaminated with helminth eggs, including 3 dogs infected *with T. canis*. In the urban site, 100% of animal fecal samples were contamination with helminths, including 22 dogs infected with *T. canis* (513).<br>**Mexico (EC)**<br>- In a study conducted in northwest Mexico, the prevalence in stray dogs was 7.1% (503).<br>**Slovakia (EC)**<br>- In a study conducted in small towns in western Slovakia, the prevalence of *T. canis* in fecal samples from 1436 dogs was 16.5% (514).<br>**Hungary (EC)**<br>- A study evaluating stool samples from dogs in eastern and northern regions of Hungary found a prevalence of *T. canis* of 24.3% -30.1% (192).<br>**South Korea (ER)**<br>- A study conducted between June and September 2004 evaluating 662 dogs recorded a prevalence of 0.9% (515).<br>**Poland (EC)**<br>- Out of 3309 soil samples from rural and urban areas of Poland, 14.9% contained eggs contaminated with *T. cati* and *T. canis* (516).<br>**Russia (EC)**<br>-A study examining the prevalence of intestinal parasites in dogs in Moscow found that T. canis had a prevalence of 2% (264).<br>-A systematic review and meta-analysis examining Toxocara prevalence in dogs found that *T.canis* prevalence varied from 3-100% (mean prevalence rate across Russia was 33%) depending on region/city and if dogs were pets or strays (517).<br>**Australia (EC)**<br>- A systematic review and meta-analysis study found the overall prevalence of *Toxocara* infection in dogs was 3.7% (511).<br>**Taiwan (EC)**<br>- A systematic review and meta-analysis study found the overall prevalence of *Toxocara* infection in dogs was 1.1% (511).	Definitive Host: domestic dogs, wild canids such as hyaenids (518).<br>Direct:<br>Unembryonate eggs are shed in the feces in the definitive host à Eggs embryonate for 1-4 weeks and become infectious (L3) à ingestion by definitive host à eggs hatch and penetrate the gut (510).<br>Young dogs: larvae migrate through the lungs where they are coughed up and swallowed → adult worms develop in intestines (510).<br>Older dogs: larvae typically fail to complete the migration back to intestines, becoming dormant "hypobiotic" larvae in somatic tissues (i.e. kidneys, liver, muscle) (518). Ocular signs, such as orbital cellulitis and multifocal retinal disease, have been described in adult dogs with *T. canis* infections (518).<br>Larvae become reactivated in females dogs during late gestation (510).<br>Indirect:<br>Ingestion of *T. canis* by paratenic host results in larvae migrating and encysting in tissues → ingestion by definitive host continues the life cycle (510).<br>Paratenic hosts: numerous mammalian, bird and invertebrate species (510).<br>An adult female worm can release 200,000 eggs per day (519).<br>In dogs, mature *T. canis* worms can survive for long periods (510).<br>The pre-patent period after ingesting *T. canis* eggs is between four to five weeks in pups, and in older animals, it can be up to two months (518).	Transportation facilities cannot be ruled out as a source of infection to other commodities, as studies have shown *T. canis* eggs can be recovered from indoor environments (Overgaauw et al., 1998)
Trichuris vulpis<br>UNCERTAINTY = Low	Parasite	Prevalence surveys confirm that *T. vulpis* occurs in dogs worldwide (520).	**United States (EC):** Found in as many as 14.3% of shelter dogs sampled in the U.S. and 10% of dogs presented to veterinary teaching hospitals (520).	**Host**: Dogs and wild canids<br>Adult *T. vulpis* live in the caecum, with their anterior ends threaded into the mucosa →	

HAZARD	CATEGORY	GEOGRAPHIC DISTRIBUTION	STATUS IN MAJOR IMPORTING COUNTRIES[i]	PATHOGEN DYNAMICS	OTHER INFORMATION
			**Ukraine (EC):** A study from Poltava city, Ukraine found a prevalence of 25.9% (521).<br>A study from the Kyiv region found a prevalence of 27.1% (521).<br>**Mexico (EC):** A study in Yucatan, Mexico found a frequency of 1% of fecal samples (1/100 samples) positive for *T. vulpis* eggs (522).<br>A study from Tabasco, Mexico found 1 fecal sample positive for *T. vulpis* out of 302 samples (188).<br>A study from Durango, Mexico found a prevalence of 4% in 50 household dog fecal samples and a prevalence of 4% in 50 stray dog samples (523).<br>**Slovakia (EC):** A study in the Slovak lowlands found a prevalence of 14.07% in 803 dog fecal samples (186).<br>**Hungary (EC):** A study examining 490 canine fecal samples from eastern and northern Hungary found a prevalence of 20.4-23.3% (192).<br>**South Korea (Uk):** No data in dogs was found.<br>**Poland (EC):** A study from Western Pomerania found a prevalence of 0.27% in 763 fecal samples collected from dogs from the cities (505).<br>A study found a prevalence of 22.2% in 207 fecal samples from dogs living in rural areas in cental and southern Poland (524).<br>**Russia (EC):** A study found a prevalence of 8.95% based on 67 autopsy examinations from the Kursk region (281).<br>**Australia (ER):** A study examining 1400 canine fecal samples from across Australia found a prevalence of 1.8% (200).<br>A study examining 1425 rural dogs from the mainland and Tasmania found a prevalence of up to 21.2% (525).<br>**Taiwan (Uk):** No data in dogs was found.	eggs are passed in the feces → eggs larvate in the environment (ideally warm + wet) in 10 days → larvated eggs (containing 1st stage larvae, the infective stage) are ingested by host → eggs hatch in intestinal lumen → larvae undergo mucosal migration and 4 moults to become adults → adults consume blood, tissue fluids, mucosal epithelium<br>Prepatent period is 74-90 days (520).	
Trypanosoma cruzi<br>(Chagas disease/American trypanosomiasis)<br>UNCERTAINTY = Low	Parasite	Endemic to South and Central America, Mexico, and in the southern United States (e.g., Texas) (526).	**United States (Sp):** Studies from Texas, Oklahoma, and Louisiana report prevalence to be 3.6-22.1% and up to 57.6% in some multi-dog kennels (527).<br>-A study sampling 1,610 working dogs from across the United States (41 states) found a seropositivity rate of 7.5% (528).<br>-Chagas disease in dogs has been observed in southern states such as Texas, Louisiana, South Carolina, Virginia, Georgia, Tennessee, and Oklahoma (529).<br>**Ukraine (Uk):** No data in dogs was found.<br>**Mexico (EC):** A study sampling 180 domestic dogs in the Sonora region found a seropositivity rate of 4.44% (530).<br>A study sampling 102 dogs from the Tejupilco Sanitary Region (southern Mexico) found a seroprevalence of 24.5% and infection rate of 34.01% (531).<br>A study sampling 209 dogs from Jalisco, Mexico found a seropositivity rate of 8.1% (532).	**Invertebrate host (vector)**: Triatomine bug (536).<br>**Mammalian hosts (reservoir)**: humans, dogs, armadillos, opossums, raccoons, woodrats and other rodents, cats, rabbits…etc (302).<br>3 developmental stages:<br>-trypomastigotes<br>-epimastigotes<br>-amastigotes<br>**Triatomine bug stage:**<br>Triatomine bug takes blood meal from infected mammalian host, ingesting trypomastigotes → trypomastigotes transform into epimastigotes in midgut → Differentiate into infective metacyclic trypomastigotes in the hindgut (302)<br>**Mammalian stage**: Infected triatomine bug takes blood meal, releasing metacyclic	In non-endemic countries, non-vectorial routes are responsible for infection. These include transplacental (vertical), through contaminated blood transfusion or tissue and organ transplantation, and during laboratory accidents (535).

HAZARD	CATEGORY	GEOGRAPHIC DISTRIBUTION	STATUS IN MAJOR IMPORTING COUNTRIES[i]	PATHOGEN DYNAMICS	OTHER INFORMATION
			Government dogs working along the USA-Mexico border had a seroprevalence of up to 18.9% (527).<br>**Slovakia (Uk):** No data in dogs was found.<br>**Hungary (Uk):** No data in dogs was found.<br>**South Korea (Ab):** Chagas disease in humans has not been reported in Korea yet (533).<br>- No data in dogs was found.<br>**Poland (Uk):** No data in dogs was found.<br>**Russia (Uk):** No data in dogs was found.<br>**Australia (Sp):** *T. cruzi* is unlikely to become established in Australia and has received little attention, with the belief that it was restricted to South America where its known triatomid vectors are found (534).<br>A study estimated Australia had 1928 infected human residents in 2011 (535).<br>No data in dogs was found.<br>**Taiwan (Uk):** No data in dogs was found.	trypomastigotes in its feces → metacyclic trypomastigotes from feces enter mammalian host through bite wound or mucosal membranes → metacyclic trypomastigotes invade cells and differentiate into amastigotes → differentiate into trypomastigotes and enter blood stream → trypomastigotes in blood stream infect cells in various tissues; there, they transform into intracellular amastigotes and cause symptomatic infection (302).	
Trypanosoma congolense, T brucei brucei<br>(Canine Trypanosomiasis)<br>UNCERTAINTY = Low	Parasite	Limited to Central Africa. Distribution is limited by the tsetse fly which is only found in Africa along the tsetse belt (537,538).	**United States (Ab):** No data in dogs was found.<br>**Ukraine (Ab):** In Europe, autochthonous cases of canine African trypanosomiasis have not been described thus far (539).<br>-No data in dogs was found.<br>**Mexico (Ab):** No data in dogs was found.<br>**Slovakia (Ab):** In Europe, autochthonous cases of canine African trypanosomiasis have not been described thus far (539).<br>-No data in dogs was found.<br>**Hungary (Ab):** In Europe, autochthonous cases of canine African trypanosomiasis have not been described thus far (539).<br>-No data in dogs was found.<br>**South Korea (Ab):** No data in dogs was found.<br>**Poland (Ab):** In Europe, autochthonous cases of canine African trypanosomiasis have not been described thus far (539).<br>**-** No data in dogs was found.<br>**Russia (Ab):** No data in dogs was found.<br>**Australia (Ab):** No data in dogs was found.<br>**Taiwan (Ab):** No data in dogs was found.<br>**Other:** 3 reports of *T. congolense* have been documented in France from dogs who were returning from Senegal and Sub-Saharan Africa (540–542).<br>-A case of *T. congolense* was documented in the UK in 2007 from a JRT returning from South Africa. This was the first case reported in the UK (543).<br>-A case of *T. congolense* was documented in Israel from 2 dogs imported from Africa (544).	**Vector**: Tsetse flies (*Glossina* species).<br>**Hosts:** Cattle (reservoir host), sheep, pigs, sheep, goat, dog, horse (additionally cats and camels for *T. brucei brucei*) (547).<br>Two major lifecycle stages:<br>-blood stream form<br>-procyclic insect form<br>**Lifecycle:**<br>Tsetse fly:<br>tsetse fly takes blood meal from infected host → ingests bloodstream trypanosomes → bloodstream trypanosomes develop into procyclics in the midgut and proliferate → procyclics migrate to tsetse fly salivary gland → transform into epimastigotes which attach to the probiscis and multiply → invade the hypopharynx and differentiate into metacyclics, the infective stage for mammals<br>Mammals:<br>Infected tsetse fly bites mammal, injecting metacyclics into the host's skin → metacyclics transform into bloodstream trypomastigotes → multiply by binary fission in various body fluids (548,549).	*T. congolense* and *T. brucei brucei* pose a serious economic constraint to livestock and agriculture production in sub-Saharan Africa, causing livestock deaths and reduced productivity (Museux et al., 2011).

HAZARD	CATEGORY	GEOGRAPHIC DISTRIBUTION	STATUS IN MAJOR IMPORTING COUNTRIES[i]	PATHOGEN DYNAMICS	OTHER INFORMATION
			-A study found that *T. congolense* had a prevalence of 30.1% from 123 outdoor watch dogs from the Ivory Coast (West Africa) (545).<br>-A study in eastern Zambia found 3/237 hunting dogs monolytically infected with *T. congolense*, 4/237 with *T. brucei brucei*, and 2/237 coinfected with *T. congolense* and *T. brucei brucei* (546).		
**Canine adenovirus type 1**<br>Infectious canine hepatitis<br>UNCERTAINTY =<br>High	Virus	Well-controlled among the domestic dog population due to widespread vaccination (550).<br>Sporadic occurrences reported in Europe, Asia, North America, and South America (551).<br>Predominately associated with puppy trading organizations in eastern Europe (552).	**United States (Sp):** Encephalopathy from CAV-1 was recorded in 9 5-week-old Labrador puppies in Arkansas from the same litter from an unvaccinated bitch (553).<br>**Ukraine (UK):** No prevalence data in dogs was found.<br>**Mexico (EC):** In a study on disease prevalence in Latin America, 35.1% (194/552) of veterinary practitioners in Mexico reported that CAV (either type 1 or 2) was present in their veterinary practice (554). No prevalence data in dogs was found.<br>**Slovakia (UK)**: No prevalence data in dogs was found.<br>**Hungary (SUS):** Although an outbreak of CAV-1 in Italy resulted from the importation of 2 purebred puppies from Hungary (555), no prevalence data in dogs was found<br>**South Korea (UK):** No prevalence data in dogs was found<br>**Poland (UK):** No prevalence data in dogs was found<br>**Russia (UK):** No prevalence data in dogs was found<br>**Australia (ER):** The overall prevalence of serum antibodies for CAdV in European red foxes was 23.2% (308/1326) (556). A novel recombinant CAV-1 was detected in two dogs from Adelaide Hill – resulted in vaccine failure and increased virulence (557).<br>**Taiwan (UK):** No prevalence data in dogs was found<br>**Other:**<br>Samples (rectal swabs from live dogs and tissue samples from dead dogs) were collected from 291 dogs in southern **Italy** between 2017-2020 who showed clinical signs or anatomopathological lesions related to infectious gastrointestinal disease – CadV-1 was detected in 2.1% of dogs, all among dead dogs (558).<br>In a veterinary teaching hospital in northern **India**, fecal samples were collected from 216 dogs with gastroenteritis between 2017-2019. 5.56% were PCR positive for CadV-1 (559).<br>Prevalence in Central Europe has decreased due to routine vaccination; may be be eliminated in some areas (560).	Direct transmission via contact with saliva, urine, or feces of infected dog (552).<br>Fleas and ticks may act as mechanical vectors (552).<br>Clinical signs present four to nine days after exposure (552).<br>Viral shedding in the urine detected up to 9 months post-infection (552).<br>The virus can survive for months at room temperature but is easily inactivated by disinfectants (552).	Vaccination very effective (552).
Canine distemper virus<br>*Canine distemper*<br>*At least 8 genotypes exist based on sequence of*	Virus	Worldwide distribution but varies significantly by strain (561–563).<br>In North America, America-2 genotype is the most common, with America-1 reported in wildlife in the USA. Europe-2 and -3	**United States (ER):**<br>7.4% of dogs who were entering shelters in the USA were positive for CDV via PCR. Higher value linked to small outbreak in western USA associated (13).	Shed in all bodily secretions as early as 5 days after exposure. Usually ceases one to two weeks post infection but can last three to four months (151).<br>Incubation period is 3 to 6 days.	CDV is part of the core vaccinations recommended for dogs in Canada (580). At this time, it is still believed that the

HAZARD	CATEGORY	GEOGRAPHIC DISTRIBUTION	STATUS IN MAJOR IMPORTING COUNTRIES[i]	PATHOGEN DYNAMICS	OTHER INFORMATION
hemagglutinin surface protein (561–563).<br>UNCERTAINTY = Moderate		have been reported in dogs and likely linked to canine importation (151,564).	2.4% of previously collected respiratory samples from a diagnostic laboratory in Georgia were positive for CDV via PCR (565).<br>**Ukraine (UK):**<br>No prevalence data in domestic dogs was found.<br>**Mexico (EC):**<br>62% of dogs sampled in a northwest community near a wildlife reservoir were seropositive with free roaming owned more likely to be positive (566).<br>**Slovakia (SUS):**<br>No prevalence data in domestic dogs was found.<br>**Hungary (EC):**<br>Of 150 samples collected from dogs displaying respiratory, gastrointestinal, or neurological signs, 30.66% were PCR positive for CDV (567).<br>**South Korea (EC):**<br>No prevalence data in domestic dogs was found.<br>44.1% of 102 tested raccoon dogs were seropositive (568).<br>**Poland (EC):**<br>22% of 224 dogs with clinical signs of distemper were antigen positive (569).<br>**Russia (EC):**<br>CVD antibodies were detected in 27% of 464 dogs sampled across three communities in the far east (570).<br>**Australia (UC):**<br>5.7% of dogs sampled on three islands in the Torres Strait were seropositive to CDV (571).<br>A retrospective study identified 48 infected dogs and ferrets from 2006-2014 (570).<br>**Taiwan (ER):**<br>Widespread vaccination has reduced burden of disease, but outbreak still reported, especially in puppies (572).<br>Prevalence in stray dogs was 18.6% in 2006 and decreased to 3.6% in 2011 (573).<br>**Other:**<br>• 28.5% of dogs sampled from veterinary teaching hospitals and local clinics in Wenzhou, China were antigen positive for CDV (574).<br>• 9% of stray dogs sampled from three areas in Turkey were seropositive (575).<br>• 4% of owned dogs that presented to a veterinary referral hospital in Iran and had at least two symptoms consistent with CDV infection were positive (576).<br>• Estimates of seroprevalence in Brazil range from 7% to >30%, depending on the region (577).	~50% infected experience subclinical or mild infection (151,564).	current vaccination is effective against various subtypes, but as new subtypes get introduced to new areas, additional research is required (563).

HAZARD	CATEGORY	GEOGRAPHIC DISTRIBUTION	STATUS IN MAJOR IMPORTING COUNTRIES[i]	PATHOGEN DYNAMICS	OTHER INFORMATION
			• 82% of apparently healthy unvaccinated free-ranging communal dogs in Zimbabwe were seropositive (578).<br>*Seroprevalence values likely an underestimate >50% succumb to the infection (579)		
Canine Herpes Virus-1<br>*Canine herpes*<br>UNCERTAINTY = Moderate	Virus	Worldwide distribution (581).<br>Seroprevalence ranges from 15% to 100% in dogs in high density environments (e.g., kennels, colonies) (581).<br>Disease reported in Canada, United States, Australia, Japan, United Kingdom, and Germany (581).	**United States (ER):** 0.8% of dogs who were entering shelters in the USA were positive for CHV-1 via PCR (13)<br>**Ukraine (UK):** No prevalence data in dogs was found.<br>**Mexico (EC):** Dogs from 8 kennels in Mexico City, the State of Mexico, Queretaro and Guadalajara Jalisco were tested for CHV-1 antibodies using ELISA. 87% of dogs tested were positive (582).<br>**Slovakia (EC):** In a study comparing 20 dogs with CIRDC and 10 healthy dogs – 60% of the CIRDC dogs were ELISA positive for CHV-1 whereas none of the healthy dogs tested positive (16).<br>**Hungary (UK):** No prevalence data in dogs was found.<br>**South Korea (EC):** 300 nasal swabs were collected from dogs in the Gwangju metropolitan city animal shelter in 2019. 31.7% were PCR positive for CHV-1 (18).<br>**Poland (EC):** 32 of 40 dogs (80%) of with upper respiratory symptoms were PCR-positive for CHV-1 (19).<br>**Russia (UK):** No prevalence data in dogs was found.<br>**Australia (SUS):** The overall prevalence of serum antibodies for CHV-1 recorded in European red foxes (n=1,375) was 2.2% (556). No prevalence data in dogs was found.<br>**Taiwan (UK):** No prevalence data in dogs was found.<br>**Other:**<br>• 22.9% of owned (n=47) and 19.1% of kenneled (n=35) dogs were seropositive for CHV-1 in southeast Iran (583).<br>• No dogs were PCR-positive for CHV-1 among 90 clinically healthy and 61 with CIRD in Germany (24).<br>• 80% (n=193) of breeding bitches from a research colony in Norway were seropositive for CHV-1 (584).<br>• ~15% (n=849) of owned and breeding dogs sampled in southern Italy were seropositive for CHV-1 (585).<br>• In the Ankara province in Turkey, 110 blood serum samples taken from clinically healthy Kangal dogs unvaccinated for CHV-1 were tested. 71.8% were seropositive (586).	Transmission occurs via direct contact with mucosal secretions from the respiratory and genital tracts. Transplacental transmission can occur as well (581).<br>Incubation period is six to ten days, with subclinical infection common (581).<br>Lifelong latent infection has been documented.<br>Reactivation commonly coincides with immunosuppression (e.g., pregnancy, high stress, current infection) (581)	

HAZARD	CATEGORY	GEOGRAPHIC DISTRIBUTION	STATUS IN MAJOR IMPORTING COUNTRIES[i]	PATHOGEN DYNAMICS	OTHER INFORMATION
***Canis familiaris* Papillomavirus**<br>Types 1 and 6 (plus others, at least 23 types have been identified)<br>Viral papilloma<br>UNCERTAINTY = Moderate	Virus	Worldwide distribution (587).	**United States (ER):** A retrospective study done in the USA and Canada to evaluate signalment, history, treatment and outcome of pedal papilloma (CPV-2) cases was conducted, confirming its presence in North America (588).<br>**Ukraine (UK):** No prevalence data was found in dogs.<br>**Mexico (UK):** No prevalence data was found in dogs.<br>**Slovakia (UK)**: No prevalence data was found in dogs.<br>**Hungary (UK):** No prevalence data was found in dogs.<br>**South Korea (ER):** In 2008, 10% of dogs (n=400) developed oral papillomatosis (CPV-1) on a breeding farm. Diagnosis was achieved through histopathology and immunohistochemistry. The papilloma type tumours are infrequently reported in Korea (589).<br>**Poland (EC):** A analysis of samples from domestic and exotic animals in Lower Silesia and other parts of Poland between 2014 and 2017 was conducted. The largest group was canine tumours (n= 9,745); of these, 265 were located within the oral cavity. 12.08% (n=32) of tumours in the oral cavity were identified as papillomas (590).<br>**Russia (EC):** 662 samples from small domestic animals were collected. Of these, 58.92% were canine tumours (n=390). Epithelial tumours comprised 23.71% (n=93) of canine tumours and of those, papillomas made up 79.56% (n=74) (591).<br>**Australia (ER):** A study evaluated puppies in a kennel of Labrador Retrievers (n=20 adults, 30 puppies) in Victoria. Saliva samples were collected from all 30 puppies. CPV types detected included one sample of CPV 8, one sample of CPV 17 and one sample of CPV 4 (592).<br>**Taiwan (EC):** 314 clinical samples were collected from dogs from various animal hospitals. 102 cases of CPV were identified (76% on skin, 22% in the oral cavity, 2% other). The types identified included type 1, 2, 6, 9, 15 and 16 (593).<br>**Other:**<br>In **Turkey**, 40 dogs showing clinical signs of oral papillomatosis were recruited from a teaching hospital in Ankara. Of these, all were positive for CPV1 DNA and 87.5% of serum samples had detectable levels of antibody against the virus (594).<br>In **Grenada**, a retrospective study on 462 cases of tumor conditions in dogs between 2001 and 2010 was conducted. Of these tumors, 5% (n= 23) were identified as papillomas (595).	Direct contact with infected dog; inoculation generally via microtrauma into cutaneous or mucosal membranes (587)<br>Incubation period ranges between 4-8 weeks and typically will regress on its own after 4-8 weeks (596).	
Canine Parvovirus<br>*Parvoviral entertitis*<br>UNCERTAINTY = Moderate	Virus	Worldwide distribution (597).<br>Different viral variants exist. CPV-2b appears to be the most common in Canada (598).	**United States (ER):** In a study evaluating the incidence of parvovirus in puppies participating in animal relocation programs, 2.3% were positive (n=4,088) (599).	Transmission is fecal-oral via contact with feces or vomitus of infected dog (597).<br>Highly resilient in the environment. Can survive for months outside of the host (597).<br>Incubation period is one to two weeks (612)	

HAZARD	CATEGORY	GEOGRAPHIC DISTRIBUTION	STATUS IN MAJOR IMPORTING COUNTRIES[i]	PATHOGEN DYNAMICS	OTHER INFORMATION
			**Ukraine (ER):** In a study in the Sumy region, the overall prevalence of CPV in dogs from the private veterinary clinic "Health" between 2015-2018 was 2.1% (600)<br>**Mexico (EC):** Rectal swabs collected from 146 dogs with clinical gastroenteritis were tested using PCR. 90 dogs (62%) were positive for CPV with 77.8% were under 6 months of age. All samples corresponded to the CPV-2c variant (601).<br>**Slovakia (UK):** No prevalence data in dogs was found.<br>**Hungary (EC):** Variant CPV-2a has been detected (602). In a study analyzing samples collected between 2004 and 2008, parvovirus was detected in 53 of 195 (27.2%) dogs tested were PCR positive for CPV-2a (603).<br>**South Korea (EC):** Out of 405 canine serum samples collected from stray dogs, overall seropositivity was 93.8%. The regional CPV-2a prevalence was 100% (8/8) in Jeju, 95.1% (232/244) in Gyeonggi, 94.7% (36/38) in Jeonra, 92.9% (13/14) in Gangwon, 92.7% (38/41) in Chungcheong, and 88.3% (53/60) in Gyeungsang province (604).<br>**Poland (EC):** In a study using SpeedParvo (PCR) test on 116 dogs from southeastern Poland, infection was confirmed in 50 dogs (43%) (605).<br>**Russia (SUS):** Although cases have been reported in Russia, no prevalence data in dogs was found.<br>**Australia (EC):** 4,870 cases of CPV were reported from an Australian disease surveillance system between September 2009 and July 2014 (606).<br>**Taiwan (EC):** Considered free of CPV-2c until first report occurred in 2015. During 2014-2016 rectal swabs from 99 dogs were collected and 88 cases were confirmed by PCR (607).<br>**Other:**<br>• Of 71 feces samples collected from suspected diarrheic dogs in and around Bhubaneswar, Odisha (India), 40.9% were PCR positive for CPV (608).<br>• 122 suffering from vomiting and diarrhea in Egypt were screened by rapid antigen test for CPV. The overall prevalence of CPV was 59.7% (609).<br>• The prevalence of CPV in central Turkey (n=80 dogs) was 86.27% (610).<br>• Variant CPV-2a is mainly prevalent in Australia, India, Hungary, Korea, China and Greece (611).	Viral shedding can occur before the onset of clinical signs and up to two weeks post-infection.<br>Subclinical infection has been documented in adult dogs (597).	
Influenza A H3n8 and h3n2<br>*Canine influenza*<br>UNCERTAINTY = Moderate	Virus	<u>H3N8</u>: Only reported in the United States (613)<br><u>H3N2</u>: Endemic in China, South Korea, and the United States (151,614,615)	(H3N8/H3N2)<br>**United States (EC/EC):** H3N2 was first reported in the United States in 2015. Neither H3N2 and H3N8 are considered endemic in the USA (614)	Transmission via direct contact with respiratory secretions or indirect contact with contaminated fomites (but only survives for minutes to hours) (625).	

HAZARD	CATEGORY	GEOGRAPHIC DISTRIBUTION	STATUS IN MAJOR IMPORTING COUNTRIES[i]	PATHOGEN DYNAMICS	OTHER INFORMATION
			Blood samples taken from 74 dogs housed in a City of Philadelphia animal shelter recorded a seroprevalence of 42% for H3N8 (616).<br>**Ukraine (AB/SUS):** Serum from 88 dogs was collected in 2016 from veterinary hospitals in Kyiv and tested using ELISA. 5.7% possessed influenza A virus antibodies (non-specific) (617).<br>**Mexico (ER/ER):** In 2012, 113 blood samples were collected from household dogs in Mexico City. Hemagglutination inhibition (HI) test was used to detect antibodies and the seroprevalence was 4% for subtype H3N8 A/equine/Kentucky/97 and 0% for subtype H3N8 A/equine/2/Miami/63. Seroprevalence was 0.9% for dogs with antibodies against both H3N2 and H1N1N (618).<br>**Slovakia (UK/UK):** No prevalence data in dogs was found.<br>**Hungary (UK/UK)**: No prevalence data in dogs was found.<br>**South Korea (AB/EC):** Canine influenza A H3N2 was first detected in dogs in South Korea in 2007 (614). The prevalence of H3N2 in dogs is 5.1% (619).<br>**Poland (ER/ER):** 496 canine sera samples submitted to the National Veterinary Research Institute in Pulawy in 2016-2017 were tested using ELISA and hemagglutination inhibition (IH). 7.25% were seropositive for Influenza A virus using HI (1.41% for H3N8, 4.23% for H3N2 and 1.61% for H1N1) (620).<br>**Russia (AB/SUS):** No prevalence data in dogs for CIV H3N8 or H3N2 was found. Suspected presence of H3N2 based on endemicity in neighbouring countries (China, South Korea).<br>**Australia (SP/AB):** In an equine influenza (H3N8) outbreak in 2007. 40 dogs housed with or near infected horses were tested with ELISA and HI and 23 were seropositive (621).<br>**Taiwan (AB/UK):** Influenza H6N1 virus is the most common naturally occurring avian influenza virus in Taiwan. 474 serum samples were collected between 2012-2013 from dogs from the National Taiwan University Veterinary Hospital and free-roaming dogs in rural areas. 1.1% of household dogs and 3.1% of free roaming dogs were seropositive for influenza A virus (622). No prevalence data in dogs for H3N2 was found.<br>**Other:**<br>• Blood samples from 272 clinically healthy dogs and 35 dogs with respiratory signs in Germany were screened with ELISA. The prevalence of H3N8 was 0%, comparable with the results of other countries other than the USA (21).<br>• In China, the first case of H3N2 among dogs was reported in Guangdong province in 2006. Between 2011-2012, 6 large-scale dog farms were selected, and 360 nasal swabs and 540 blood samples were	Subclinical infections can occur and are common (~20% for H3N8) (626).<br>Incubation period two to eight days (626).<br>Shedding begins two to four days after exposure and clinical signs may be absent (625).	

HAZARD	CATEGORY	GEOGRAPHIC DISTRIBUTION	STATUS IN MAJOR IMPORTING COUNTRIES[i]	PATHOGEN DYNAMICS	OTHER INFORMATION
			collected from dogs found at these farms. Additionally, 500 nasal swabs and 900 blood samples were collected from 60 different pet hospitals. H3N2 seroprevalence was 12.22% and 5.3% in pet dogs and farmed dogs respectively (623).<br>• As of December 2021, outbreaks of H3N2 canine influenza have been reported in Korea, China, Thailand and the United States (624).		

[1] The following categories were used to classify the hazard in exporting countries: **EC**: hazard is **e**ndemic and **c**ommon throughout the country; **ER**: hazard is **e**ndemic but **r**are throughout the country; **EL**: hazard is **e**ndemic but **l**imited to one or more zones throughout the country; **Sp**: **sp**oradic occurrence of hazard has been identified in the country but insufficient data to determine country level status; **Sus**: hazard is **sus**pected based on the prevalence in surrounding geographic area or wild canid populations; **Ab**: hazard is **ab**sent in country; **Uk**: Status of hazard is **u**n**k**nown.

References

1. Diniz PPVP, Chomel BB, Guptill L, Breitschwerdt EB. 70 - Bartonellosis. In: Sykes JE, editor. Greene's Infectious Diseases of the Dog and Cat (Fifth Edition). Philadelphia: W.B. Saunders; 2021 [cited 2025 Feb 21]. p. 853–75. Available from: https://www.sciencedirect.com/science/article/pii/B9780323509343000707

2. Chomel BB, Boulouis HJ, Maruyama S, Breitschwerdt EB. *Bartonella* Spp. in pets and effect on human health. Emerg Infect Dis. 2006 Mar;12(3):389–94.

3. Cockwill KR, Taylor SM, Philibert HM, Breitschwerdt EB, Maggi RG. *Bartonella vinsonii* subsp. *berkhoffii* endocarditis in a dog from Saskatchewan. Can Vet J. 2007 Aug;48(8):839–44.

4. Breitschwerdt EB, Hegarty BC, Hancock SI. Sequential evaluation of dogs naturally infected with *Ehrlichia canis, Ehrlichia chaffeensis, Ehrlichia equi, Ehrlichia ewingii,* or *Bartonella vinsonii*. J Clin Microbiol. 1998 Sep;36(9):2645–51.

5. Levytska VA, Mushinsky AB. Monitoring of vector-borne diseases in the west part of Ukraine. Sci Messenger LNU Vet Med Biotechnol Ser Vet Sci. 2019 Dec 14;21(96):14–8.

6. Tobar BZ, Lapsley WD, Swain WL, Jaffe DA, Setien AA, Galvez-Romero G, et al. *Bartonella* in dogs and fleas from Tulancingo, Hidalgo, Mexico. Med Vet Entomol. 2020 Sep;34(3):302–8.

7. Špitalská E, Minichová L, Kocianová E, Škultéty Ľ, Mahríková L, Hamšíková Z, et al. Diversity and prevalence of *Bartonella* species in small mammals from Slovakia, Central Europe. Parasitol Res. 2017 Nov;116(11):3087–95.

8. Jiyipong T, Jittapalapong S, Morand S, Rolain JM. *Bartonella* species in small mammals and their potential vectors in Asia. Asian Pac J Trop Biomed. 2014 Oct 1;4(10):757–67.

9. Zarea AAK, Bezerra-Santos MA, Nguyen VL, Colella V, Dantas-Torres F, Halos L, et al. Occurrence and bacterial loads of *Bartonella* and haemotropic *Mycoplasma* species in privately owned cats and dogs and their fleas from East and Southeast Asia. Zoonoses Public Health. 2022 Sep;69(6):704–20.

10. Reagan KL. 55 - Bordetellosis. In: Sykes JE, editor. Greene's Infectious Diseases of the Dog and Cat (Fifth Edition). Philadelphia: W.B. Saunders; 2021 [cited 2025 Feb 21]. p. 669–78. Available from: https://www.sciencedirect.com/science/article/pii/B9780323509343000550

11. Reagan KL, Sykes JE. Canine Infectious Respiratory Disease. Vet Clin North Am Small Anim Pract. 2020 Mar;50(2):405–18.

12. Datz C. VetFolio. 2019 [cited 2025 Feb 17]. Bordetella Infections in Dogs and Cats: Treatment and Prevention. Available from: https://www.vetfolio.com/learn/article/bordetella-infections-in-dogs-and-cats-treatment-and-prevention

13. Lavan R, Knesl O. Prevalence of canine infectious respiratory pathogens in asymptomatic dogs presented at US animal shelters. J Small Anim Pract. 2015 Sep;56(9):572–6.

14. Okonkowski LK, Szlosek D, Ottney J, Coyne M, Carey SA. Asymptomatic carriage of canine infectious respiratory disease complex pathogens among healthy dogs. J Small Anim Pract. 2021 Aug;62(8):662–8.

15. Damián M, Morales E, Salas G, Trigo FJ. Immunohistochemical detection of antigens of distemper, adenovirus and parainfluenza viruses in domestic dogs with pneumonia. J Comp Pathol. 2005 Nov;133(4):289–93.

16. Vojtek B, Smrčo P, Haladová E, Mojzisova J, Hipikova V. The occurrence of antibodies against canine herpesvirus in infectious tracheobronchitis of dogs. Folia Vet. 2010;54:96–7.

17. Mitchell JA, Cardwell JM, Leach H, Walker CA, Le Poder S, Decaro N, et al. European surveillance of emerging pathogens associated with canine infectious respiratory disease. Vet Microbiol. 2017 Dec;212:31–8.

18. Koh, Ba-Ra-Da, Kim, Han-Na, Kim, Hyo-Jung, et al. A survey of respiratory pathogens in dogs for adoption in Gwangju metropolitan city animal shelter, South Korea. Korean J Vet Serv. 2020 Jun 30;43(2):67–77.

19. Kaczorek E, Schulz P, Małaczewska J, Wójcik R, Siwicki AK, Stopyra A, et al. Prevalence of respiratory pathogens detected in dogs with kennel cough in Poland. Acta Vet Brno. 2017 Jan 30;85(4):329–36.

20. Hiebl A, Auer A, Bagrinovschi G, Stejskal M, Hirt R, Rümenapf HT, et al. Detection of selected viral pathogens in dogs with canine infectious respiratory disease in Austria. J Small Anim Pract. 2019 Oct;60(10):594–600.

21. Schulz B, Klinkenberg C, Fux R, Anderson T, De Benedictis P, Hartmann K. Prevalence of canine influenza virus A (H3N8) in dogs in Germany. Vet J. 2014 Oct;202(1):184–5.

22. Sowman H, Cave N, Dunowska M. A survey of canine respiratory pathogens in New Zealand dogs. N Z Vet J. 2018 Sep 3;66(5):236–42.

23. Priestnall SL, Pratelli A, Brownlie J, Erles K. Serological Prevalence of Canine Respiratory Coronavirus in Southern Italy and Epidemiological Relationship with Canine Enteric Coronavirus. J Vet Diagn Invest. 2007 Mar;19(2):176–80.

24. Schulz BS, Kurz S, Weber K, Balzer HJ, Hartmann K. Detection of respiratory viruses and *Bordetella bronchiseptica* in dogs with acute respiratory tract infections. Vet J Lond Engl. 2014 Sep;201(3):365–9.

25. Santos RL, Souza TD, Mol JPS, Eckstein C, Paíxão TA. Canine Brucellosis: An Update. Front Vet Sci. 2021 Mar 2 [cited 2025 Feb 2];8. Available from: https://www.frontiersin.org/journals/veterinary-science/articles/10.3389/fvets.2021.594291/full

26. Sykes J, Davidson A. Canine Brucellosis. In: Canine and Feline Infectious Diseases 1st ed. Elsevier/Saunders; 2014. p. 512–9.

27. Bolotin VI, Pikun OYu, Marchenko NV, Kozhevnik IYa, Rudova NG, Solodiankin OS, et al. First report of vanine brucellosis in Ukraine: Pathogen isolation and characterization. In: Journal for Veterinary Medicine, Biotechnology and Biosafety. 2020 [cited 2025 Jan 12]. p. 5–8. Available from: http://jvmbbs.kharkov.ua/archive/2020/volume6/issue4/article1.php

28. Hubbard K, Wang M, Smith DR. Seroprevalence of brucellosis in Mississippi shelter dogs. Prev Vet Med. 2018 Nov 1;159:82–6.

29. López-Diez L, Ortiz-Román L, Sanabria-Gonzalez W, Sanchez-Nodarse R, Henao-Correa E, Olivera-Angel M. Seroprevalence of *Brucella canis* and *Leptospira spp*. in canines in the city of Medellín, Colombia. Vet Zootencía. 2020;14(1):34–48.

30. Holst BS, Löfqvist K, Ernholm L, Eld K, Cedersmyg M, Hallgren G. The first case of *Brucella canis* in Sweden: background, case report and recommendations from a northern European perspective. Acta Vet Scand. 2012 Mar 27;54(1):18.

31. Buhmann G, Paul F, Herbst W, Melzer F, Wolf G, Hartmann K, et al. Canine Brucellosis: Insights into the epidemiologic situation in Europe. Front Vet Sci. 2019 May 31;6:151.

32. Jung JY, Yoon SS, Lee SH, Park JW, Lee JJ, Her M, et al. Prevalence state of canine brucellosis in South Korea during 2015 and 2016. Korean J Vet Res. 2018 Sep 30;58(3):125–9.

33. Spickler AR. The Center for Food Security & Public Health. 2013. Canine Brucellosis: Brucella Canis. Available from: https://www.cfsph.iastate.edu/FastFacts/pdfs/canine_brucellosis_F.pdf

34. Brennan SJ, Ngeleka M, Philibert HM, Forbes LB, Allen AL. Canine brucellosis in a Saskatchewan kennel. Can Vet J. 2008 Jul;49(7):703–8.

35. Nöckler K, Kutzer P, Reif S, Rosenberger N, Draeger A, Bahn P, et al. Canine brucellosis--a case report. Berl Munch Tierarztl Wochenschr. 2003;116(9–10):368–72.

36. Gyuranecz M, Szeredi L, Rónai Z, Dénes B, Dencso L, Dán Á, et al. Detection of *Brucella canis*-induced reproductive diseases in a kennel. J Vet Diagn Investig Off Publ Am Assoc Vet Lab Diagn Inc. 2011 Jan;23(1):143–7.

37. Corrente M, Franchini D, Decaro N, Greco G, D'Abramo M, Greco MF, et al. Detection of *Brucella canis* in a dog in Italy. New Microbiol. 2010 Oct;33(4):337–41.

38. Djokic V, Freddi L, de Massis F, Lahti E, van den Esker MH, Whatmore A, et al. The emergence of *Brucella canis* as a public health threat in Europe: what we know and what we need to learn. Emerg Microbes Infect. 12(2):2249126.

39. O'Brien C, Sykes JE. 77 - Miscellaneous Bacterial Infections. In: Greene's Infectious Diseases of the Dog and Cat. 5th ed. Elsevier; 2023. p. 948–59.

40. Ryan CW, Bishop K, Blaney DD, Britton SJ, Cantone F, Egan C, et al. Public health response to an imported case of canine melioidosis. Zoonoses Public Health. 2018 Jun;65(4):420–4.

41. Lee SW, Yi J, Joo SI, Kang YA, Yoon YS, Yim JJ, et al. A case of melioidosis presenting as migrating pulmonary infiltration: The first case in Korea. J Korean Med Sci. 2005;20(1):139.

42. Sprague LD. Merck Veterinary Manual. 2022 [cited 2025 Feb 20]. Melioidosis in Animals - Infectious Diseases. Available from: https://www.merckvetmanual.com/infectious-diseases/melioidosis/melioidosis-in-animals

43. Liu L, Gee J, Blaney D. Melioidosis | CDC Yellow Book 2024. 2023 [cited 2025 Feb 20]. Available from: https://wwwnc.cdc.gov/travel/yellowbook/2024/infections-diseases/melioidosis

44. Fungwithaya P, Boonhoh W, Sontigun N, Hayakijkosol O, Klangbud WK, Wongtawan T. Seroprevalence of melioidosis and its association with blood profiles and pathogens in sheltered dogs in southern Thailand. Vet World. 2024 Mar;705–11.

45. Acke E. 65 - Campylobacteriosis. In: Sykes JE, editor. Greene's Infectious Diseases of the Dog and Cat (Fifth Edition) [Internet]. Philadelphia: W.B. Saunders; 2021 [cited 2025 Feb 21]. p. 774–84. Available from: https://www.sciencedirect.com/science/article/pii/B9780323509343000653

46. Francois Watkins LK, Laughlin ME, Joseph LA, Chen JC, Nichols M, Basler C, et al. Ongoing outbreak of extensively drug-resistant *Campylobacter jejuni* infections associated with US pet store puppies, 2016-2020. JAMA Netw Open. 2021 Sep 15;4(9):e2125203.

47. Candellone A, Badino P, Girolami F, Cerquetella M, Nebbia P, Aresu L, et al. Concomitant Campylobacteriosis in a puppy and in its caregiver: A One Health perspective paradigm in human-pet relationship. Vet Sci. 2023 Apr;10(4):244.

48. Rijks JM, Cito F, Cunningham AA, Rantsios AT, Giovannini A. Disease risk assessments involving companion animals: an Overview for 15 selected pathogens taking a European perspective. J Comp Pathol. 2016 Jul 1;155(1, Supplement 1):S75–97.

49. Badlík M, Holoda E, Pistl J, Koscová J, Sihelská Z. Prevalence of zoonotic *Campylobacter* spp. in rectal swabs from dogs in Slovakia: special reference to *C. jejuni* and *C. coli*. Berl Munch Tierarztl Wochenschr. 2014;127(3–4):144–8.

50. Olvera-Ramírez AM, McEwan NR, Stanley K, Nava-Diaz R, Aguilar-Tipacamú G. A systematic review on the role of wildlife as carriers and spreaders of *Campylobacter* spp. Animals. 2023 Jan;13(8):1334.

51. Andrzejewska M, Szczepańska B, Klawe JJ, Śpica D, Chudzińska M. Prevalence of *Campylobacter jejuni* and *Campylobacter coli* species in cats and dogs from Bydgoszcz (Poland) region. Pol J Vet Sci. 2013 Mar 1;16(1):115–20.

52. Stafford RJ, Schluter PJ, Wilson AJ, Kirk MD, Hall G, Unicomb L, et al. Population-attributable risk estimates for risk factors associated with *Campylobacter* infection, Australia. Emerg Infect Dis. 2008 Jun;14(6):895–901.

53. Tsai HJ, Huang HC, Lin CM, Lien YY, Chou CH. Salmonellae and campylobacters in household and stray dogs in northern Taiwan. Vet Res Commun. 2007 Nov;31(8):931–9.

54. Lemos ML, Nunes A, Ancora M, Cammà C, Costa PM da, Oleastro M. *Campylobacter jejuni* in different canine populations: Characteristics and zoonotic potential. Microorganisms. 2021 Oct 26;9(11):2231.

55. Lion C, Escande F, Burdin JC. *Capnocytophaga canimorsus* infections in human: review of the literature and cases report. Eur J Epidemiol. 1996 Oct;12(5):521–33.

56. Zajkowska J, Król M, Falkowski D, Syed N, Kamieńska A. *Capnocytophaga canimorsus* – an underestimated danger after dog or cat bite – review of literature. Przegl Epidemiol. 2016;70(2):289–95.

57. Dilegge SK, Edgcomb VP, Leadbetter ER. Presence of the oral bacterium *Capnocytophaga canimorsus* in the tooth plaque of canines. Vet Microbiol. 2011 May 5;149(3–4):437–45.

58. Lai CH, Lin YS, Wang CM, Chang PC, Shia WY. A Novel 16S rRNA PCR-restriction fragment length polymorphism assay to accurately distinguish zoonotic *Capnocytophaga canimorsus* and *C. cynodegmi*. Microbiol Spectr. 2023 Jun 15;11(3):e0291622.

59. Suzuki M, Kimura M, Imaoka K, Yamada A. Prevalence of *Capnocytophaga canimorsus* and *Capnocytophaga cynodegmi* in dogs and cats determined by using a newly established species-specific PCR. Vet Microbiol. 2010 Jul 29;144(1–2):172–6.

60. Mally M, Paroz C, Shin H, Meyer S, Soussoula LV, Schmiediger U, et al. Prevalence of *Capnocytophaga canimorsus* in dogs and occurrence of potential virulence factors. Microbes Infect. 2009 Apr;11(4):509–14.

61. Tabatabaei M, Rouhani HR. Polymerase Chain Reaction Detection of *Capnocytophaga canimorsus* and *Capnocytophaga cynodegmi* as the emerging zoonosis. Avicenna J Clin Microbiol Infect. 2019 Feb 28;6(1):21–5.

62. Blanche P, Bloch E, Sicard D. *Capnocytophaga canimorsus* in the oral flora of dogs and cats. J Infect. 1998 Jan;36(1):134.

63. Ahmad S, Yousaf A, Inayat F, Anjum S. *Capnocytophaga canimorsus*-associated sepsis presenting as acute abdomen: do we need to think outside the box? BMJ Case Rep. 2019 Mar 14;12(3):e228167.

64. Navarrete MG, Hodžić A, Corona-González B, Cordeiro MD, da Silva CB, Báez LC, et al. Novel *Ehrlichia canis* genogroup in dogs with canine ehrlichiosis in Cuba. Parasit Vectors. 2022 Aug 23;15(1):295.

65. Harrus S, Waner T, Mylonakis ME, Sykes JE, Qurollo B. 44 - Ehrlichiosis. In: Sykes JE, editor. Greene's Infectious Diseases of the Dog and Cat (Fifth Edition). Philadelphia: W.B. Saunders; 2021 [cited 2025 Feb 21]. p. 522–41. Available from: https://www.sciencedirect.com/science/article/pii/B9780323509343000446

66. Gettings JR, Self SCW, McMahan CS, Brown DA, Nordone SK, Yabsley MJ. Local and regional temporal trends (2013–2019) of canine *Ehrlichia* spp. seroprevalence in the USA. Parasit Vectors. 2020 Mar 30;13:153.

67. Movilla R, García C, Siebert S, Roura X. Countrywide serological evaluation of canine prevalence for *Anaplasma* spp., *Borrelia burgdorferi* (sensu lato), *Dirofilaria immitis* and *Ehrlichia canis* in Mexico. Parasit Vectors. 2016 Jul 29;9(1):421.

68. Letkova V, Mojzisova J, Winkler R, Curlik J, Letko M, Bajova V. The seroprevalence of *Ehrlichia canis* in dogs in East Slovakia. Folia Vet. 2004;48(3):135–8.

69. Suh GH, Ahn KS, Ahn JH, Kim HJ, Leutenegger C, Shin S. Serological and molecular prevalence of canine vector-borne diseases (CVBDs) in Korea. Parasit Vectors. 2017 Mar 16;10(1):146.

70. Lim S, Irwin PJ, Lee S, Oh M, Ahn K, Myung B, et al. Comparison of selected canine vector-borne diseases between urban animal shelter and rural hunting dogs in Korea. Parasit Vectors. 2010 Apr 8;3(1):32.

71. Dzięgiel B, Adaszek Ł, Carbonero A, Łyp P, Winiarczyk M, Dębiak P, et al. Detection of canine vector-borne diseases in eastern Poland by ELISA and PCR. Parasitol Res. 2016 Mar;115(3):1039–44.

72. Krämer F, Schaper R, Schunack B, Połozowski A, Piekarska J, Szwedko A, et al. Serological detection of *Anaplasma phagocytophilum*, *Borrelia burgdorferi* sensu lato and *Ehrlichia canis* antibodies and *Dirofilaria immitis* antigen in a countrywide survey in dogs in Poland. Parasitol Res. 2014 Sep 1;113(9):3229–39.

73. Livanova NN, Fomenko NV, Akimov IA, Ivanov MJ, Tikunova NV, Armstrong R, et al. Dog survey in Russian veterinary hospitals: tick identification and molecular detection of tick-borne pathogens. Parasit Vectors. 2018 Nov 14;11(1):591.

74. Australian Government. Ehrlichiosis: additional veterinary guidelines for managing CME in Australia - DAFF. 2023 [cited 2025 Feb 13]. Available from: https://www.agriculture.gov.au/biosecurity-trade/pests-diseases-weeds/animal/ehrlichiosis-in-dogs/veterinarians

75. Wildlife Health Australia. *Ehrlichia canis* and wild dingoes in Australia disease risk assessment. Animal Health Committee; 2022 [cited 2025 Feb 13]. Available from: https://wildlifehealthaustralia.com.au/Portals/0/ResourceCentre/BiosecurityMgmt/E_canis_dingos_DRA_Feb2022.pdf

76. Chaber AL, Easther R, Cumming B, Irving R, Keyburn A, Smart C, et al. *Ehrlichia canis* rapid spread and possible enzooty in northern South Australia and distribution of its vector *Rhipicephalus linnaei*. Aust Vet J. 2022 Nov;100(11):533–8.

77. Tsai KH, Chang SF, Yen TY, Shih WL, Chen WJ, Wang HC, et al. Prevalence of antibodies against *Ehrlichia* spp. and *Orientia tsutsugamushi* in small mammals around harbors in Taiwan. Parasit Vectors. 2016 Jan 27;9(1):45.

78. Huang CC, Hsieh YC, Tsang CL, Chun YT. Sequence and phylogenetic analysis of the gp200 protein of *Ehrlichia canis* from dogs in Taiwan. J Vet Sci. 2010 Dec;11(4):333-340. https://doi.org/10.4142/jvs.2010.11.4.333

79. Yuasa Y, Tsai YL, Chang CC, Hsu TH, Chou CC. The prevalence of *Anaplasma platys* and a potential novel *Anaplasma* species exceed that of *Ehrlichia canis* in asymptomatic dogs and *Rhipicephalus sanguineus* in Taiwan. J Vet Med Sci. 2017 Sep 12;79(9):1494–502.

80. Western College of Veterinary Medicine. 2021 [cited 2025 Feb 13]. *Rhipicephalus sanguineus*: brown dog tick. Available from: https://wcvm.usask.ca/learnaboutparasites/parasites/rhipicephalus-sanguineus-brown-dog-tick.php

81. Sainz Á, Roura X, Miró G, Estrada-Peña A, Kohn B, Harrus S, et al. Guideline for veterinary practitioners on canine ehrlichiosis and anaplasmosis in Europe. Parasit Vectors. 2015 Feb 4;8(1):75.

82. Ojeda-Chi MM, Rodriguez-Vivas RI, Esteve-Gasent MD, Pérez de León AA, Modarelli JJ, Villegas-Perez SL. *Ehrlichia canis* in dogs of Mexico: Prevalence, incidence, co-infection and factors associated. Comp Immunol Microbiol Infect Dis. 2019 Dec;67:101351.

83. Han JI, Na KJ. *Ehrlichia ewingii* infection in a dog from South Korea – A case report. Korean J. Vet. Serv. 2018;41(4).

84. Lindsay LR. Summary: Tick Species in Ontario. Public Health Ontario; 2023 [cited 2025 Feb 13]. Available from: https://www.publichealthontario.ca/-/media/Documents/T/2023/tick-species-ontario-lyme.pdf

85. Schuller S, Moore GE, Sykes JE. 68 - Leptospirosis. In: Sykes JE, editor. Greene's Infectious Diseases of the Dog and Cat (Fifth Edition). Philadelphia: W.B. Saunders; 2021 [cited 2025 Feb 21]. p. 802–23. Available from: https://www.sciencedirect.com/science/article/pii/B9780323509343000689

86. Browne ES, Callefe JLR, Jesus ERSD, Zeppelini CG, Cremonese C, Costa F. A Systematic Review of the geographic distribution of pathogenic *Leptospira* serovars in the Americas, 1930-2017. An Acad Bras Cienc. 2022;94(3):e20201026.

87. World Health Organization. Leptospirosis: Fact Sheet. 2009 [cited 2025 Feb 13]. Available from: https://www.who.int/publications/i/item/B4221

88. Pappas G, Papadimitriou P, Siozopoulou V, Christou L, Akritidis N. The globalization of leptospirosis: worldwide incidence trends. Int J Infect Dis. 2008 Jul;12(4):351–7.

89. Katharine F. Lunn. Merck Veterinary Manual. [cited 2025 Feb 13]. Leptospirosis in Dogs - Infectious Diseases. Available from: https://www.merckvetmanual.com/infectious-diseases/leptospirosis/leptospirosis-in-dogs

90. Smith AM, Arruda AG, Evason MD, Weese JS, Wittum TE, Szlosek D, et al. A cross-sectional study of environmental, dog, and human-related risk factors for positive canine leptospirosis PCR test results in the United States, 2009 to 2016. BMC Vet Res. 2019 Nov 15;15(1):412.

91. Zubach O, Semenyshyn O, Hatsji L, Demchyshyn M, Zinchuk A. *Leptospira interrogans* in mammals in Lviv Oblast, Ukraine, 2001-2015. Caimano MJ, editor. PLoS Negl Trop Dis. 2019 Dec 2;13(12):e0007793.

92. Jimenez-Coello M, Ortega-Pacheco A, Guzman-Marin E, Guiris-Andrade DM, Martinez-Figueroa L, Acosta-Viana KY. Stray Dogs as Reservoirs of the Zoonotic Agents *Leptospira interrogans*, *Trypanosoma cruzi*, and *Aspergillus* spp. in an Urban Area of Chiapas in Southern Mexico. Vector-Borne Zoonotic Dis. 2010 Mar;10(2):135–41.

93. Skardova I, Prokopcako H, Cislakova L, Sesztakova E, Skarda J. Monitoring Leptospirosis affecting dogs in Eastern Slovakia. Pak J Biol Sci. 2000 Feb 15;3(3):433–5.

94. Kurucz K, Madai M, Hederics D, Bali D, Kemenesi G, Jakab F. Molecular survey of zoonotic agents in rodents from an urban environment, Hungary. Int J Infect Dis. 2019 Feb;79:67–8.

95. Vojinović D, Bogićević N, Vasić A, Manić M, Elezović Radovanović M, Rogožarski D, et al. Seroepidemiological survey of leptospiral infection in stray dogs in Serbia. Turk J Vet Anim Sci. 2015;39:719–23.

96. Kim MJ. Leptospirosis in the republic of Korea: historical perspectives, current status and future challenges. Infect Chemother. 2013 Jun;45(2):137–44.

97. Żmudzki J, Arent Z, Jabłoński A, Nowak A, Zębek S, Stolarek A, et al. Seroprevalence of 12 serovars of pathogenic *Leptospira* in red foxes (*Vulpes vulpes*) in Poland. Acta Vet Scand. 2018 Dec;60(1):34.

98. Krawczyk M. Serological evidence of leptospirosis in animals in northern Poland. Vet Rec. 2005 Jan 15;156(3):88–9.

99. Scahill K, Windahl U, Boqvist S, Pelander L. *Leptospira* seroprevalence and associated risk factors in healthy Swedish dogs. BMC Vet Res. 2022 Oct 22;18(1):376.

100. Stoyanova NA, A SN, Tokarevich NK, K TN. Immunological screening of leptospirosis in dogs in St. Petersburg. Russ J Infect Immun. 2018 Dec 30;8(4):550.

101. Orr B, Westman ME, Malik R, Purdie A, Craig SB, Norris JM. Leptospirosis is an emerging infectious disease of pig-hunting dogs and humans in North Queensland. Blevins J, editor. PLoS Negl Trop Dis. 2022 Jan 18;16(1):e0010100.

102. Lai CJ, Liu CC, Ho D, Pan MJ. Seroprevalence of *Leptospira* infection among stray dogs at Northern Taiwan. Taiwan Vet J. 2005 Mar 1;31(1).

103. Chou CH, Yeh TM, Lu YP, Shih WL, Chang CD, Chien CH, et al. Prevalence of zoonotic pathogens by molecular detection in stray dogs in central Taiwan. Thai J Vet Med. 2014 Sep 1;44(3):363–75.

104. Bergmann Esteves S, Moreira Santos C, Ferreira Salgado F, Paldês Gonçales A, Gil Alves Guilloux A, Marinelli Martins C, et al. Efficacy of commercially available vaccines against canine leptospirosis: A systematic review and meta-analysis. Vaccine. 2022 Mar;40(12):1722–40.

105. Barker EN, Tasker S. 58 - Hemotropic Mycoplasma Infections. In: Sykes JE, editor. Greene's Infectious Diseases of the Dog and Cat (Fifth Edition). Philadelphia: W.B. Saunders; 2021 [cited 2025 Feb 21]. p. 690–703. Available from: https://www.sciencedirect.com/science/article/pii/B9780323509343000586

106. Novacco M, Meli ML, Gentilini F, Marsilio F, Ceci C, Pennisi MG, et al. Prevalence and geographical distribution of canine hemotropic mycoplasma infections in Mediterranean countries and analysis of risk factors for infection. Vet Microbiol. 2010 May 19;142(3):276–84.

107. Compton SM, Maggi RG, Breitschwerdt EB. Candidatus *Mycoplasma haematoparvum* and *Mycoplasma haemocanis* infections in dogs from the United States. Comp Immunol Microbiol Infect Dis. 2012 Dec;35(6):557–62.

108. Hamel D, Silaghi C, Zapadynska S, Kudrin A, Pfister K. Vector-borne pathogens in ticks and EDTA-blood samples collected from client-owned dogs, Kiev, Ukraine. Ticks Tick-Borne Dis. 2013 Feb 1;4(1):152–5.

109. Arenas P, Gil-Alarcón G, Sánchez-Montes S, Soto-Trujillo MP, Fernández-Figueroa E, Rangel-Escareño C. Molecular detection of *Bartonella*, *Ehrlichia* and *Mycoplasma* in feral dogs of El Pedregal de San Angel Ecological Reserve in Mexico City. Rev Bras Parasitol Veterinária. 2019 Nov 7;28:728–34.

110. Koneval M, Miterpáková M, Hurníková Z, Blaňarová L, Víchová B. Neglected intravascular pathogens, *Babesia vulpes* and haemotropic *Mycoplasma* spp. in European red fox (*Vulpes vulpes*) population. Vet Parasitol. 2017 Aug 30;243:176–82.

111. Hamel D, Silaghi C, Lescai D, Pfister K. Epidemiological aspects on vector-borne infections in stray and pet dogs from Romania and Hungary with focus on *Babesia* spp. Parasitol Res. 2012 Apr;110(4):1537–45.

112. Tuska-Szalay B, Vizi Z, Hofmann-Lehmann R, Vajdovich P, Takács N, Meli ML, et al. *Babesia gibsoni* emerging with high prevalence and co-infections in "fighting dogs" in Hungary. Curr Res Parasitol Vector-Borne Dis. 2021 Sep 24;1:100048.

113. Seo MG, Kwon OD, Kwak D. Molecular detection and phylogenetic analysis of canine tick-borne pathogens from Korea. Ticks Tick-Borne Dis. 2020 Mar;11(2):101357.

114. Hetzel NJL, Barker EN, Helps CR, Tasker S, Arteaga A, Barrs VR, et al. Prevalence of canine haemotropic *Mycoplasma* infections in Sydney, Australia. Vet Rec. 2012;171(5):126–126.

115. Messick JB. Hemotrophic mycoplasmas (hemoplasmas): a review and new insights into pathogenic potential. Vet Clin Pathol. 2004;33(1):2–13.

116. Spickler AR. The Center for Food Security and Public Health. 2023 [cited 2025 Feb 2]. Spotted Fevers including Rocky Mountain spotted fever and Mediterranean spotted fever. Available from: https://www.cfsph.iastate.edu/Factsheets/pdfs/RMSF.pdf

117. Centers for Disease Control and Prevention. Imported spotted fevers. 2024 [cited 2025 Feb 15]. Available from: https://www.cdc.gov/other-spotted-fever/hcp/imported/index.html

118. Rovery C, Brouqui P, Raoult D. Questions on Mediterranean spotted fever a century after its discovery. Emerg Infect Dis. 2008 Sep;14(9):1360–7.

119. Spernovasilis N, Markaki I, Papadakis M, Mazonakis N, Ierodiakonou D. Mediterranean spotted fever: Current knowledge and recent advances. Trop Med Infect Dis. 2021 Sep 24;6(4):172.

120. Solano-Gallego L, Caprì A, Pennisi MG, Caldin M, Furlanello T, Trotta M. Acute febrile illness is associated with *Rickettsia* spp infection in dogs. Parasit Vectors. 2015 Apr 10;8(1):216.

121. Raoult D, Dupont HT, Chicheportiche C, Peter O, Gilot B, Drancourt M. Mediterranean Spotted Fever in Marseille, France: Correlation between prevalence of hospitalized patients, seroepidemiology, and prevalence of infected ticks in three different areas. Am J Trop Med Hyg. 1993 Feb;48(2):249–56.

122. Oaks JB, Lasam G, LaCapra G. Mediterranean spotted fever: A rare non-endemic disease in the USA. Cureus. 2017 Jan 12;9(1):e974.

123. Rovery C, Raoult D. Mediterranean spotted fever. Infect Dis Clin North Am. 2008 Sep;22(3):515–30, ix.

124. Kováčová E, Sekeyová Z, Trávniček M, Bhide MR, Mardzinová S, Čurlik J, et al. Monitoring of humans and animals for the presence of various rickettsiae and *Coxiella burnetii* by serological methods. Ann N Y Acad Sci. 2006 Oct;1078(1):587–9.

125. Espejo E, Andrés M, Pérez J, Prat J, Guerrero C, Muñoz MT, et al. Prevalence of antibodies to *Rickettsia conorii* in human beings and dogs from Catalonia: a 20-year perspective. Epidemiol Infect. 2016 Jul;144(9):1889–94.

126. Punda-Polić V, Bradarić N, Klišmanić-Nuber Z, Mrljak V, Giljanović M. Antibodies to spotted fever group rickettsiae in dogs in Croatia. Eur J Epidemiol. 1995 Aug;11(4):389–92.

127. MacConnachie K, Tishkowski K. Boutonneuse Fever. In: StatPearls. Treasure Island (FL): StatPearls Publishing; 2023 [cited 2025 Feb 15]. Available from: http://www.ncbi.nlm.nih.gov/books/NBK560914/

128. Matsumoto K, Brouqui P, Raoult D, Parola P. Experimental infection models of ticks of the *Rhipicephalus sanguineus* group with *Rickettsia conorii*. Vector-Borne Zoonotic Dis. 2005 Dec;5(4):363–72.

129. Sexton DJ, Banks J, Graves S, Hughes K, Dwyer B. Prevalence of antibodies to spotted fever group Rickettsiae in dogs from southeastern Australia. Am J Trop Med Hyg. 1991 Aug;45(2):243–8.

130. Ortuño A, Pons I, Nogueras MM, Castellà J, Segura F. The dog as an epidemiological marker of *Rickettsia conor*ii infection. Clin Microbiol Infect. 2009 Dec;15:241–2.

131. Wagner B. research-groups-cpep. 2019 [cited 2025 Feb 15]. Ticks and Mites - Canadian Parasitology Expert Panel Guidelines | CPEP. Available from: https://research-groups.usask.ca/cpep/parasites/ticks-and-mites-supplement.php

132. Herrin BH, Peregrine AS, Goring J, Beall MJ, Little SE. Canine infection with *Borrelia burgdorferi, Dirofilaria immitis, Anaplasma* spp. and *Ehrlichia* spp. in Canada, 2013–2014. Parasit Vectors. 2017 May 19;10(1):244.

133. Pasquetti M, Min A, Scacchetti S, Dogliero A, Peano A. Infection by *Microsporum canis* in paediatric patients: A veterinary perspective. Vet Sci. 2017 Sep 19;4(3):46.

134. Moriello KA, Coyner K. 78 - Dermatophytosis. In: Sykes JE, editor. Greene's Infectious Diseases of the Dog and Cat (Fifth Edition). Philadelphia: W.B. Saunders; 2021 [cited 2025 Feb 21]. p. 961–77. Available from: https://www.sciencedirect.com/science/article/pii/B9780323509343000781

135. Sykes JE, Outerbridge C. Dermatophytosis. In: Canine and Feline Infectious Diseases. 1st ed. Elsevier/Saunders; 2014. p. 558–69.

136. Lewis DT, Foil CS, Hosgood G. Epidemiology and clinical features of dermatophytosis in dogs and cats at Louisiana State University: 1981–1990. Vet Dermatol. 1991 Jun;2(2):53–8.

137. Ponomarenko GV, Kovalenko VL, Ponomarenko OV, Severyn RV, Gontar AM, Shostak VI, et al. Monitoring of dermatophytosis incidence in domestic dogs and cats in Kharkiv, Ukraine. J Vet Med Biotechnol Biosaf. 2019 Mar 25;5(1):17–9.

138. Torres–Guerrero E, González De Cossío A, Segundo Z, Cervantes O, Ruiz EJ, Arenas R. *Microsporum canis* and other dermatophytes isolated from humans, dogs and cats in Mexico City. Glob Dermatol. 2016;3(2):275–8.

139. Kottferová L, Molnár L, Čonková E, Major P, Sesztáková E, Szarková A, et al. Fungal flora in asymptomatic pet guinea pigs and rabbits. Anim. 2022 Sep 13;12(18):2387.

140. Ginter-Hanselmayer G, Weger W, Ilkit M, Smolle J. Epidemiology of tinea capitis in Europe: current state and changing patterns. Mycoses. 2007;50 Suppl 2:6–13.

141. Jung ES, Lee SK, Lee IJ, Nam KH, Yun SK, Kim HU, et al. Three cases of recalcitrant pediatric tinea capitis successfully treated with griseofulvin. Ann Dermatol. 2023 Nov;35(Suppl 2):S247–51.

142. Savinov VA, Kapustin AV, Ovchinnikov RS, Shastin PN, Laishevtsev AI. Incidence and seasonal variation of pet dermatophytosis in Moscow region. IOP Conf Ser Earth Environ Sci. 2020 Aug 1;548(7):072048.

143. Chang CC, Wechtaisong W, Chen SY, Cheng MC, Chung CS, Lin LS, et al. Prevalence and risk factors of zoonotic dermatophyte infection in pet rabbits in Northern Taiwan. J Fungi Basel Switz. 2022 Jun 13;8(6):627.

144. Tsai YJ, Chung WC, Wang LC, Ju YT, Hong CL, Tsai YY, et al. The dog mite, *Demodex canis*: prevalence, fungal co-infection, reactions to light, and hair follicle apoptosis. J Insect Sci Online. 2011;11:76.

145. Sparkes AH, Gruffydd-Jones TJ, Shaw SE, Wright AI, Stokes CR. Epidemiological and diagnostic features of canine and feline dermatophytosis in the United Kingdom from 1956 to 1991. Vet Rec. 1993 Jul 17;133(3):57–61.

146. Galuppi R, Leveque JFC, Beghelli V, Bonoli C, Mattioli M, Ostanello F, et al. Cortisol levels in cats' hair in presence or absence of *Microsporum canis* infection. Res Vet Sci. 2013 Dec;95(3):1076–80.

147. Chermette R, Ferreiro L, Guillot J. Dermatophytoses in animals. Mycopathologia. 2008;166(5–6):385–405.

148. Cafarchia C, Romito D, Sasanelli M, Lia R, Capelli G, Otranto D. The epidemiology of canine and feline dermatophytoses in southern Italy. Mycoses. 2004 Dec;47(11–12):508–13.

149. Seker E, Dogan N. Isolation of dermatophytes from dogs and cats with suspected dermatophytosis in Western Turkey. Prev Vet Med. 2011 Jan;98(1):46–51.

150. Moriello KA, Coyner K, Paterson S, Mignon B. Diagnosis and treatment of dermatophytosis in dogs and cats: Clinical Consensus Guidelines of the World Association for Veterinary Dermatology. Vet Dermatol. 2017 Jun;28(3):266-e68.

151. Sykes JE. Canine and Feline Infectious Diseases. Elsevier; 2014 [cited 2024 Oct 9]. Available from: https://linkinghub.elsevier.com/retrieve/pii/C20090413709

152. Spickler AR. The Center for Food Security & Public Health. 2013. Dermatophytosis. Available from: https://www.cfsph.iastate.edu/Factsheets/pdfs/dermatophytosis.pdf

153. Moriello KA. Merck Veterinary Manual. 2020 [cited 2025 Feb 17]. Dermatophytosis in Dogs and Cats - Integumentary System. Available from: https://www.merckvetmanual.com/integumentary-system/dermatophytosis/dermatophytosis-in-dogs-and-cats

154. University of Cambridge. 2015 [cited 2025 Feb 17]. Canine transmissible venereal tumour (CTVT). Available from: https://www.tcg.vet.cam.ac.uk/about/ctvt

155. Strakova A, Murchison EP. The changing global distribution and prevalence of canine transmissible venereal tumour. BMC Vet Res. 2014 Sep 3;10:168.

156. Ganguly B, Das U, Das AK. Canine transmissible venereal tumour: a review. Vet Comp Oncol. 2016 Mar;14(1):1–12.

157. Kutzler M. Merck Veterinary Manual. 2024 [cited 2025 Feb 17]. Transmissible Venereal Tumor in Dogs - Dog Owners. Available from: https://www.merckvetmanual.com/dog-owners/reproductive-disorders-of-dogs/transmissible-venereal-tumor-in-dogs

158. Ortega-Pacheco A, Acevedo-Arcique M, Sauri-Arceo C. Prevalence of transmissible venereal tumor of stray dogs in Merida Yucatan Mexico. Rev Biomed. 2003;14(2):83-87.

159. Beuchat C. The Institute of Canine Biology. 2016 [cited 2025 Feb 17]. The world's oldest cancer...in dogs. Available from: http://www.instituteofcaninebiology.org/7/post/2016/08/the-worlds-oldest-cancerin-dogs.html

160. Abeka YT. Review on Canine Transmissible Venereal Tumor (CTVT). Cancer Ther Oncol Int J [Internet]. 2019 Jul 30 [cited 2025 Feb 17];14(4). Available from: https://juniperpublishers.com/ctoij/CTOIJ.MS.ID.555895.php

161. Arcila-Villa A, Dussán-Lubert C, Universidad de Caldas, Pedraza-Ordoñez F, Universidad de Caldas. Distribution and prevalence of transmissible venereal tumor in the Colombian canine population. Rev Colomb Cienc Pecu. 2018 Jul;31(3):180–7.

162. Mello Martins MI, Ferreira de Souza F, Gobello C. The canine transmissible venereal tumor: Etiology, pathology, diagnosis and treatment. Recent Adv Small Anim Reprod. 2005;

163. Snowden KF, Ketzis JK. 118 - Trematodes. In: Sykes JE, editor. Greene's Infectious Diseases of the Dog and Cat (Fifth Edition). Philadelphia: W.B. Saunders; 2021 [cited 2025 Feb 21]. p. 1528–49. Available from: https://www.sciencedirect.com/science/article/pii/B978032350934300118X

164. Szafranski N, Gerhold R. Just a Fluke? Trematode Infections of Dogs in the United States. Today's Veterinary Practice. 2022 [cited 2024 Jul 2]. Available from: https://todaysveterinarypractice.com/parasitology/trematode-infections-of-dogs-in-the-united-states/

165. Nagamori Y, Payton ME, Looper E, Apple H, Johnson EM. Retrospective survey of endoparasitism identified in feces of client-owned dogs in North America from 2007 through 2018. Vet Parasitol. 2020 Jun 1;282:109137.

166. Johnson EM, Nagamori Y, Duncan-Decocq RA, Whitley PN, Ramachandran A, Reichard MV. Prevalence of *Alaria* infection in companion animals in north central Oklahoma from 2006 through 2015 and detection in wildlife. J Am Vet Med Assoc. 2017 Apr 15;250(8):881–6.

167. Lyulin P, Fedorova O. Zoonotic intestinal fluke diseases of foxes (*Vulpes vulpes*) in Ukraine. J Vet Med Biotechnol Biosaf. 2019 Jun 24;5:11–4.

168. Shoop WL, Salazar MA, Vega CS, Font WF, Infante F. *Alaria nasuae* (Trematoda: Diplostomidae) from domestic dogs. J Parasitol. 1989 Apr;75(2):325–7.

169. Luna-Estrada M, Mosqueda-Cabrera MÁ, Servín J. Nuevos registros de helmintos en coyote Canis latrans impavidus (Carnivora: Canidae) en México. Rev Mex Biodivers. 2017 Mar;88(1):250–2.

170. Čabanová V, Guimaraes N, Hurníková Z, Chovancová G, Urban P, Miterpáková M. Endoparasites of the grey wolf (*Canis lupus*) in protected areas of Slovakia. Ann Parasitol. 2017;63(4):283–9.

171. Miterpáková M, Hurníková Z, Antolová D, Dubinský P. Endoparasites of red fox (*Vulpes vulpes*) in the Slovak Republic with the emphasis on zoonotic species *Echinococcus multilocularis* and *Trichinella* spp. Helminthologia. 2009 Jun 1;46(2):73–9.

172. Széll Z, Tolnai Z, Sréter T. Environmental determinants of the spatial distribution of *Alaria alata* in Hungary. Vet Parasitol. 2013 Nov 15;198(1):116–21.

173. Takács A, Szabó L, Juhász L, Takács AA, Lanszki J, Takács PT, et al. Data on the parasitological status of golden jackal (Canis aureus L., 1758) in Hungary. Acta Vet Hung. 2014 Mar;62(1):33–41.

174. Lee HW, Hong EJ, Kim HC, Ryu SY, Park BK. *Alaria alata* (Digenea: Diplostomidae) from Korean Raccoon Dog, *Nyctereutes procyonoides koreensis*, in Korea. Korean J Parasitol. 2022 Oct 21;60(5):361–5.

175. Tylkowska A, Pilarczyk B, Pilarczyk R, Zyśko M, Tomza-Marciniak A. The presence of *Alaria alata* fluke in the red fox (*Vulpes vulpes*) in north-western Poland. Jpn J Vet Res. 2018 Aug;66(3):203–8.

176. Pilarczyk BM, Tomza-Marciniak AK, Pilarczyk R, Rząd I, Bąkowska MJ, Udała JM, et al. Infection of raccoon dogs (*Nyctereutes procyonoides*) from Northern Poland with gastrointestinal parasites as a potential threat to human health. J Clin Med. 2022 Feb 26;11(5):1277.

177. Kokolova, Gavrilieva, Okhlopkov, Sivtseva, Stepanova, Mamaev, et al. Study on the distribution of trematode *Alaria alata* (Goeze, 1782) in the red fox (*Vulpes vulpes*) in Yakutia. Theory Pract Parasit Dis Control. 2022 Apr 18;(23):234–9.

178. Companion Animal Parasite Council. Alaria. 2020 [cited 2024 Jul 2]. Available from: https://capcvet.org/guidelines/alaria/

179. Western College of Veterinary Medicine. [cited 2024 Jul 2]. *Alaria* species. Available from: https://wcvm.usask.ca/learnaboutparasites/parasites/alaria-species.php

180. Centers for Disease Control and Prevention. CDC - DPDx - Zoonotic Hookworm. 2019 [cited 2024 Jul 2]. Available from: https://www.cdc.gov/dpdx/zoonotichookworm/index.html

181. Western College of Veterinary Medicine. 2020 [cited 2024 Jul 2]. *Ancylostoma caninum*. Available from: https://wcvm.usask.ca/learnaboutparasites/parasites/ancylostoma-caninum.php

182. Bowman DD, Lucio-Forster A, Lee ACY. 113 - Hookworms. In: Sykes JE, editor. Greene's Infectious Diseases of the Dog and Cat (Fifth Edition). Philadelphia: W.B. Saunders; 2021 [cited 2025 Feb 21]. p. 1436–43. Available from: https://www.sciencedirect.com/science/article/pii/B9780323509343001130

183. Companion Animal Parasite Council. Parasite prevalence maps. [cited 2024 Jul 2]. Available from: https://capcvet.org/maps/

184. Little SE, Johnson EM, Lewis D, Jaklitsch RP, Payton ME, Blagburn BL, et al. Prevalence of intestinal parasites in pet dogs in the United States. Vet Parasitol. 2009 Dec 3;166(1):144–52.

185. Liotta JL, Youn H, Aksel S, Bienhoff SE, Bowman DD. Prevalence of *Ancylostoma braziliense* in dogs from Alachua and Marion Counties, Florida, United States. J Parasitol. 2012 Oct;98(5):1039–40.

186. Ihnacik L, Šmigová J, Šoltys J, Blišťan P, Kovanič L, Blišťanová M, et al. Risk factors associated with dog endoparasites infection spread in East Slovak Lowland. Helminthologia. 2023 Jun 1;60(2):152–60.

187. Romero C, Mendonza GE, Pineda MA, Nava N, Bautista LG, Heredia R. Prevalence of intestinal parasites with zoonotic potential in canids in Mexico City. Acta Sci Vet Impr. 2015;1–6.

188. Torres-Chablé OM, García-Herrera RA, Hernández-Hernández M, Peralta-Torres JA, Ojeda-Robertos NF, Blitvich BJ, et al. Prevalence of gastrointestinal parasites in domestic dogs in Tabasco, southeastern Mexico. Rev Bras Parasitol Veterinária. 2015 Dec 4;24:432–7.

189. Rodríguez-Vivas RI, Gutierrez-Ruiz E, Bolio-González ME, Ruiz-Piña H, Ortega-Pacheco A, Reyes-Novelo E, et al. An epidemiological study of intestinal parasites of dogs from Yucatan, Mexico, and their risk to public health. Vector Borne Zoonotic Dis Larchmt N. 2011 Aug;11(8):1141–4.

190. Jarošová J, Antolová D, Lukáč B, Maďari A. A Survey of intestinal helminths of dogs in Slovakia with an emphasis on zoonotic species. Animals. 2021 Oct 19;11(10):3000.

191. Štrkolcová G, Mravcová K, Mucha R, Mulinge E, Schreiberová A. Occurrence of hookworm and the first molecular and morphometric identification of *Uncinaria stenocephala* in dogs in Central Europe. Acta Parasitol. 2022 Jun 1;67(2):764–72.

192. Fok E, Szatmári V, Busák K, Rozgonyi F. Epidemiology: Prevalence of intestinal parasites in dogs in some urban and rural areas of Hungary. Vet Q. 2001 Apr;23(2):96–8.

193. Youn H. Review of zoonotic parasites in medical and veterinary fields in the Republic of Korea. Korean J Parasitol. 2009 Oct;47(Suppl):S133–41.

194. Studzińska MB, Demkowska-Kutrzepa M, Borecka A, Meisner M, Tomczuk K, Roczeń-Karczmarz M, et al. Variations in the rate of infestations of dogs with zoonotic nematodes and the contamination of soil in different environments. Int J Environ Res Public Health. 2017 Sep 1;14(9):1003.

195. Bartosik J, Łojek J, Puchała M, Kaczyk J, Górski P, Długosz E, et al. Prevalence of intestinal parasites detected in routine coproscopic methods in dogs and cats from the Masovian voivodeship in 2012-2015. Med Weter. 2019;75(01):6153–2019.

196. Shchelkanov MY, Tabakaeva TV, Fomenko PV, Kim EM, Tabakaev AV, Galkina IV. A retrospective study of *Uncinaria stenocephala* in domestic dogs: Age, sex distribution, and risk factors. Vet World. 2021 Jan;14(1):265–9.

197. Moskvina TV, Atopkin DM. The prevalence of intestinal parasites of domestic cats and dogs in Vladivostok, Russia during 2014–2017. Zool Ecol. 2018 Jul 3;28(3):180–4.

198. Harriott L, Gentle M, Traub R, Magalhães RJS, Cobbold R. Zoonotic and economically significant pathogens of peri-urban wild dogs across north-eastern New South Wales and south-eastern Queensland, Australia. Wildl Res. 2019 Mar;46(3):212–21.

199. Beknazarova M, Whiley H, Traub R, Ross K. Opportunistic mapping of *Strongyloides stercoralis* and hookworm in dogs in remote Australian communities. Pathogens. 2020 May 21;9(5):398.

200. Palmer CS, Thompson RCA, Traub RJ, Rees R, Robertson ID. National study of the gastrointestinal parasites of dogs and cats in Australia. Vet Parasitol. 2008 Feb 14;151(2):181–90.

201. Traub RJ, Hobbs RP, Adams PJ, Behnke JM, Harris PD, Thompson RCA. A case of mistaken identity – reappraisal of the species of canid and felid hookworms (*Ancylostoma*) present in Australia and India. Parasitology. 2007 Jan;134(1):113–9.

202. Companion Animal Parasite Council. Hookworms. 2023 [cited 2024 Jul 2]. Available from: https://capcvet.org/guidelines/hookworms/

203. Conboy GA. Canine angiostrongylosis: The French heartworm: An emerging threat in North America. Vet Parasitol. 2011 Mar;176(4):382–9.

204. Kistler WM, Brown JD, Allison AB, Nemeth NM, Yabsley MJ. First report of *Angiostrongylus vasorum* and *Hepatozoon* from a red fox (*Vulpes vulpes*) from West Virginia, USA. Vet Parasitol. 2014 Feb;200(1–2):216–20.

205. Schnyder M, Schaper R, Lukács Z, Hornok S, Farkas R. Combined serological detection of circulating *Angiostrongylus vasorum* antigen and parasite-specific antibodies in dogs from Hungary. Parasitol Res. 2015 Aug;114(S1):145–54.

206. Miterpáková M, Hurníková Z, Zalewski A. The first clinically manifested case of angiostrongylosis in a dog in Slovakia. Acta Parasitol. 2014 Jan 1;59(4):661–5.

207. Hurníková Z, Čabanová V, Karpjak P, Kasenčák M, Miterpáková M. Rare case of *Angiostrongylus vasorum* intraocular infestation in an asymptomatic dog. Helminthologia. 2019 Nov 5;56(4):319–22.

208. Uribe M, Segeritz L, Schnyder M, Taubert A, Hermosilla C, López-Osorio S, et al. Nationwide seroprevalence survey of *Angiostrongylus vasorum*-derived antigens and specific antibodies in dogs from Colombia. Microorganisms. 2022 Aug 4;10(8):1565.

209. Demiaszkiewicz A, Pyziel A, Kuligowska I, Lachowicz J. The first report of *Angiostrongylus vasorum* (Nematoda; Metastrongyloidea) in Poland, in red foxes (*Vulpes vulpes*). Acta Parasitol. 2014;59(4):758–62.

210. Schnyder M, Schaper R, Pantchev N, Kowalska D, Szwedko A, Deplazes P. Serological detection of circulating *Angiostrongylus vasorum* antigen- and parasite-specific antibodies in dogs from Poland. Parasitol Res. 2013 Aug;112(S1):109–17.

211. Tebb A, Johnson V, Irwin P. *Angiostrongylus vasorum* (French heartworm) in a dog imported into Australia. Aust Vet J. 2007 Jan;85(1–2):23–8.

212. Western College of Veterinary Medicine. 2021 [cited 2025 Feb 18]. *Angiostrongylus vasorum*. Available from: https://wcvm.usask.ca/learnaboutparasites/parasites/angiostrongylus-vasorum.php

213. Mahjoub HA, Robbins WT, Galeuzzi O, Graham KF, Jones MEB, Buote MA, et al. First report of *Angiostrongylus vasorum* (French heartworm) in red foxes (*Vulpes vulpes*) on Prince Edward Island. Can Vet J Rev Veterinaire Can. 2022 Jun;63(6):637–40.

214. Bourque AC, Conboy G, Miller LM, Whitney H. Pathological findings in dogs naturally infected with *Angiostrongylus vasorum* in Newfoundland and Labrador, Canada. J Vet Diagn Investig Off Publ Am Assoc Vet Lab Diagn Inc. 2008 Jan;20(1):11–20.

215. Brianti E. Proceedings of 6th European *Dirofilaria* and *Angiostrongylus* Days: Belgrade, Serbia. July 5 - 7, 2018. Parasit Vectors. 2018 Dec;11(S1):623, s13071-018-3183-z.

216. Newfoundland and Labrador Department of Fisheries, Forestry and Agriculture. French heartworm infection of dogs and foxes in Newfoundland. 2011 [cited 2025 Feb 18]. Available from: https://www.gov.nl.ca/ffa/files/agrifoods-animals-health-pdf-ds-08-009.pdf

217. Birkenheuer AJ. Chapter 75 - Babesiosis. In: Sykes JE, editor. Canine and Feline Infectious Diseases. Saint Louis: W.B. Saunders; 2014 [cited 2024 Jul 2]. p. 727–38. Available from: https://www.sciencedirect.com/science/article/pii/B9781437707953000752

218. Carter P, Rolls P. Babesiosis in Animals. 2022 [cited 2024 Jul 2]. Available from: https://www.merckvetmanual.com/circulatory-system/blood-parasites/babesiosis-in-animals

219. Birkenheuer AJ. 97 - Babesiosis. In: Sykes JE, editor. Greene's Infectious Diseases of the Dog and Cat (Fifth Edition). Philadelphia: W.B. Saunders; 2021 [cited 2025 Feb 21]. p. 1203–17. Available from: https://www.sciencedirect.com/science/article/pii/B9780323509343000975

220. Barash NR, Thomas B, Birkenheuer AJ, Breitschwerdt EB, Lemler E, Qurollo BA. Prevalence of *Babesia* spp. and clinical characteristics of *Babesia vulpes* infections in North American dogs. J Vet Intern Med. 2019;33(5):2075–81.

221. Taboada J, Harvey JW, Levy MG, Breitschwerdt EB. Seroprevalence of babesiosis in Greyhounds in Florida. J Am Vet Med Assoc. 1992 Jan 1;200(1):47–50.

222. Macintire DK, Boudreaux MK, West GD, Bourne C, Wright JC, Conrad PA. *Babesia gib*soni infection among dogs in the southeastern United States. J Am Vet Med Assoc. 2002 Feb 1;220(3):325–9.

223. Hamel D, Silaghi C, Zapadynska S, Kudrin A, Pfister K. Vector-borne pathogens in ticks and EDTA-blood samples collected from client-owned dogs, Kiev, Ukraine. Ticks Tick-Borne Dis. 2013 Feb 1;4(1):152–5.

224. Bajer A, Kowalec M, Levytska VA, Mierzejewska EJ, Alsarraf M, Poliukhovych V, et al. Tick-Borne Pathogens, *Babesia* spp. and *Borrelia burgdorferi* s.l. in Sled and Companion Dogs from Central and North-Eastern Europe. Pathogens. 2022 Apr 21;11(5):499.

225. Lira-Amaya JJ, Rojas-Martinez C, Alvarez A, Pelaez-Flores A, Martinez-Ibañez F. First molecular detection of *Babesia canis vogeli* in dogs and *Rhipicephalus sanguineus* from Mexico. Arch Palliat Care. 2017;2(2):1013.

226. Birkenheuer AJ, Buch J, Beall MJ, Braff J, Chandrashekar R. Global distribution of canine *Babesia* species identified by a commercial diagnostic laboratory. Vet Parasitol Reg Stud Rep. 2020 Dec 1;22:100471.

227. Kubelová M, Sedlák K, Panev A, Široký P. Conflicting results of serological, PCR and microscopic methods clarify the various risk levels of canine babesiosis in Slovakia: A complex approach to *Babesia canis* diagnostics. Vet Parasitol. 2013 Jan 31;191(3):353–7.

228. Víchová B, Horská M, Blaňarová L, Švihran M, Andersson M, Peťko B. First molecular identification of *Babesia gibsoni* in dogs from Slovakia, central Europe. Ticks Tick-Borne Dis. 2016 Feb 1;7(1):54–9.

229. Hornok S, Edelhofer R, Farkas R. Seroprevalence of canine babesiosis in Hungary suggesting breed predisposition. Parasitol Res. 2006 Nov 1;99(6):638–42.

230. Hornok S, Horváth G, Takács N, Kontschán J, Szőke K, Farkas R. Molecular identification of badger-associated *Babesia* sp. DNA in dogs: updated phylogeny of piroplasms infecting Caniformia. Parasit Vectors. 2018 Apr 11;11(1):235.

231. Welc-Falęciak R, Rodo A, Siński E, Bajer A. *Babesia canis* and other tick-borne infections in dogs in Central Poland. Vet Parasitol. 2009 Dec 23;166(3):191–8.

232. Hii S, Kopp S, Thompson M, O'Leary C, Rees R, Traub R. Canine vector-borne disease pathogens in dogs from south-east Queensland and north-east Northern Territory. Aust Vet J. 2012 Apr;90(4):130–5.

233. Su BL, Liu PC, Fang JC, Jongejan F. Correlation between *Babesia* apecies affecting dogs in Taiwan and the local distribution of the vector ticks. Vet Sci. 2023 Mar 16;10(3):227.

234. Companion Animal Parasite Council. *Babesia* spp. 2019 [cited 2024 Jul 2]. Available from: https://capcvet.org/guidelines/babesia/

235. Conboy GA, Sykes JE. 117 - Nematode Infections of the Respiratory Tract. In: Sykes JE, editor. Greene's Infectious Diseases of the Dog and Cat (Fifth Edition). Philadelphia: W.B. Saunders; 2021 [cited 2025 Feb 21]. p. 1505–27. Available from: https://www.sciencedirect.com/science/article/pii/B9780323509343001178

236. Pohly AG, Nijveldt EA, Stone MS, Walden HDS, Ossiboff RJ, Conrado FO. Infection with the fox lungworm (*Crenosoma vulpis*) in two dogs from New England – Two clinical reports and updated geographic distribution in North America. Vet Parasitol Reg Stud Rep. 2022 May 1;30:100714.

237. Companion Animal Parasite Council. *Eucoleus aerophilus*. 2018 [cited 2024 Jul 2]. Available from: https://capcvet.org/guidelines/eucoleus-aerophilus/

238. Varodi E, Malega A, Kuzmin Y, Kornyushin V. Helminths of wild predatory mammals of Ukraine. Nematodes. Vestn Zool. 2017 Jan 27;51.

239. Šmigová J, Papajová I, Šoltys J, Pipiková J, Šmiga Ľ, Šnábel V, et al. The occurence of endoparasites in Slovakian household dogs and cats. Vet Res Commun. 2021 Dec 1;45(4):243–9.

240. Cabanova V, Hurnikova Z, Miterpakova M, Dirbakova K, Bendova A, Kocak P. Lungworm infections in dogs from Central Europe. Veterinární Medicína. 2018 Aug 31;63(8):367–72.

241. Tolnai Z, Széll Z, Sréter T. Environmental determinants of the spatial distribution of *Angiostrongylus vasorum*, *Crenosoma vulpis* and *Eucoleus aerophilus* in Hungary. Vet Parasitol. 2015 Jan 30;207(3):355–8.

242. András T, Péter T. Data on worm infestation of domestic cats (*Felis catus*) in Hungarian hunting areas. Magy Allatorvosok Lapja. 2002 Jan 1;124:26–30.

243. Choi S, Sim C, Kim HC, Choi HJ, Park BK. Natural infection of *Crenosoma vulpis* (Nematoda: Crenosomatidae) in an urban Korean dog. Korean J Vet Res. 2014 Jun 30;54(2):127–9.

244. Hajnalová M, Svobodová V, Schnyder M, Schaper R, Svoboda M. Faecal detection of the lungworms *Crenosoma vulpis* and *Angiostrongylus vasorum* and serological detection of *A. vasorum* in dogs from the Czech Republic. Acta Vet Brno. 2017;86(4):393–8.

245. Karamon J, Dąbrowska J, Kochanowski M, Samorek-Pieróg M, Sroka J, Różycki M, et al. Prevalence of intestinal helminths of red foxes (*Vulpes vulpes*) in central Europe (Poland): a significant zoonotic threat. Parasit Vectors. 2018 Dec;11(1):436.

246. Wierzbowska IA, Kornaś S, Piontek AM, Rola K. The prevalence of endoparasites of free ranging cats (*Felis catus*) from urban habitats in southern Poland. Animals. 2020 Apr 24;10(4):748.

247. Shamaev N, Fedotova A, Galiullina AV, Mukminov M, Shuralev E. Prevalence and risk factors of Toxoplasma-like and intestinal parasites in cats from urbanized area of Tatarstan, Russia. Dusunen Adam. 2018 Nov 1;9:465–71.

248. Calvani NED, Wright M, White J, Stepkovitch B, Francis E, Rivory P, et al. What the fox? Cryptic *Eucoleus* [*Capillaria*] sp. in the respiratory tract of a cat from Australia. Curr Res Parasitol Vector-Borne Dis. 2021 Jan 1;1:100028.

249. Centorrino F. *Crenosoma vulpis*. Microbiologia Italia. 2022 [cited 2024 Jul 2]. Available from: https://www.microbiologiaitalia.it/parassitologia/crenosoma-vulpis/

250. Western College of Veterinary Medicine. 2021 [cited 2024 Jul 2]. *Eucoleus (Capillaria) aerophila*. Available from: https://wcvm.usask.ca/learnaboutparasites/parasites/eucoleus-capillaria-aerophila.php

251. Ryan U, Zahedi A, Feng Y, Xiao L. An Update on Zoonotic *Cryptosporidium* Species and Genotypes in Humans. Anim. 2021 Nov 19;11(11):3307.

252. Western College of Veterinary Medicine. [cited 2024 Jul 3]. *Cryptosporidium* species. Available from: https://wcvm.usask.ca/learnaboutparasites/parasites/cryptosporidium-species.php

253. Taghipour A, Olfatifar M, Bahadory S, Godfrey SS, Abdoli A, Khatami A, et al. The global prevalence of *Cryptosporidium* infection in dogs: A systematic review and meta-analysis. Vet Parasitol. 2020 May 1;281:109093.

254. Wang A, Ruch-Gallie R, Scorza V, Lin P, Lappin MR. Prevalence of *Giardia* and *Cryptosporidium* species in dog park attending dogs compared to non-dog park attending dogs in one region of Colorado. Vet Parasitol. 2012 Mar 23;184(2):335–40.

255. el-Ahraf A, Tacal JV, Sobih M, Amin M, Lawrence W, Wilcke BW. Prevalence of cryptosporidiosis in dogs and human beings in San Bernardino County, California. J Am Vet Med Assoc. 1991 Feb 15;198(4):631–4.

256. Scorza V, Lappin MR. 103 - Cryptosporidiosis and Cyclosporiasis. In: Sykes JE, editor. Greene's Infectious Diseases of the Dog and Cat (Fifth Edition). Philadelphia: W.B. Saunders; 2021 [cited 2025 Feb 21]. p. 1285–300. Available from: https://www.sciencedirect.com/science/article/pii/B9780323509343001038

257. Gonzalez-Saldívar DS, Trasviña-Muñoz E, López-Valencia G, Haro P, Monge-Navarro FJ, Herrera-Ramírez JC, et al. Frequency and risk factors of intestinal parasites in pet dogs from Mexicali, Mexico. Austral J Vet Sci. 2023;55(2):131–6.

258. Martínez-Barbabosa I, Gutiérrez M, Ruiz LA, Fernández AM, Gutiérrez EM, Aguilar JM, et al. Detección de *Cryptosporidium* spp. y otros parásitos zoonóticos entéricos en perros domiciliados de la Ciudad de México. Arch Med Vet. 2015;47(3):347–53.

259. Goldova M, Valencakova A, Mojzisova J, Halanova M, Letkova V, Ravaszova P. Occurrence of *Giardia* and *Cryptosporidium* in dogs. In Southern European Veterinary Conference; 2011.

260. Plutzer J, Lassen B, Jokelainen P, Djurković-Djaković O, Kucsera I, Dorbek-Kolin E, et al. Review of *Cryptosporidium* and *Giardia* in the eastern part of Europe, 2016. Eurosurveillance. 2018 Jan 25;23(4):16.

261. Kim JT, Wee SH, Lee CG. Detection of *Cryptosporidium* oocysts in canine fecal samples by immunofluorescence assay. Korean J Parasitol. 1998 Jun;36(2):147–9.

262. Yun CS, Moon BY, Lee K, Hwang SH, Ku BK, Hwang MH. Prevalence and genotype analysis of *Cryptosporidium* and *Giardia duodenalis* from shelter dogs in South Korea. Vet Parasitol Reg Stud Rep. 2024 Apr 1;55:101103.

263. Piekara-Stępińska A, Piekarska J, Gorczykowski M. *Cryptosporidium* spp. in dogs and cats in Poland. Ann Agric Environ Med. 2021;28(2):345–7.

264. Kurnosova OP, Panova OA, Arisov MV. The prevalence of potentially zoonotic intestinal parasites in dogs and cats in Moscow, Russia. Helminthologia. 2023 Mar 1;60(1):44–51.

265. Hsu CH, Liang C, Chi SC, Lee KJ, Chou CH, Lin CS, et al. An epidemiological assessment of *Cryptosporidium* and *Giardia* spp. infection in pet animals from Taiwan. Animals. 2023 Jan;13(21):3373.

266. Companion Animal Parasite Council. Cryptosporidium. 2022 [cited 2024 Jul 3]. Available from: https://capcvet.org/guidelines/cryptosporidium/

267. Western College of Veterinary Medicine. 2021 [cited 2024 Jul 3]. *Dioctophyma renale*. Available from: https://wcvm.usask.ca/learnaboutparasites/parasites/dioctophyma-renale.php

268. Eiras J, Zhu XQ, Yurlova N, Pedrassani D, Yoshikawa M, Nawa Y. *Dioctophyme renale* (Goeze, 1782) (Nematoda, Dioctophymidae) parasitic in mammals other than humans: A comprehensive review. Parasitol Int. 2021 Apr 1;81:102269.

269. Park HY, Seo JW, Lee BH, Lee JY, Kim SY, Cha SJ, et al. Simultaneous occurrence of malignant fibrous histiocytoma of the ureter and *Dioctophyma Renale* infection: A case report. J Korean Soc Radiol. 2013;68(5):411.

270. Russo ZH, Callirgos JC, García-Ayachi A, Wetzel EJ. Review of *Dioctophyme renale*: Etiology, morphology, biology, ecoepidemiology, pathogenesis, symptomatology, diagnosis, treatment, and prevention. J Parasitol. 2022 Apr;108(2):180–91.

271. Greer T, Amaro AA, Wilson D, Smrdelj M. Giant red kidney worm (*Dioctophyma renale*) screening and treatment protocol and aberrant worm migration in dogs from Ontario and Manitoba, Canada. J Parasitol. 2021 Apr;107(2):358–63.

272. Centers for Disease Control and Prevention. Dioctophymiasis. 2019 [cited 2024 Jul 3]. Available from: https://www.cdc.gov/dpdx/dioctophymiasis/index.html

273. Centers for Disease Control and Prevention. *Dipylidium caninum*. 2019 [cited 2024 Jul 3]. Available from: https://www.cdc.gov/dpdx/dipylidium/index.html

274. Western College of Veterinary Medicine. 2021 [cited 2024 Jul 3]. *Ctenocephalides (felis) felis* (cat flea) *Ctenocephalides (felis) canis* (dog flea). Available from: https://wcvm.usask.ca/learnaboutparasites/parasites/ctenocephalides.php

275. Western College of Veterinary Medicine. 2021 [cited 2024 Jul 3]. *Dipylidium caninum*. Available from: https://wcvm.usask.ca/learnaboutparasites/parasites/dipylidium-caninum.php

276. Cabello RR, Ruiz AC, Feregrino RR, Romero LC, Feregrino RR, Zavala JT. *Dipylidium caninum* infection. BMJ Case Rep. 2011 Nov 15;2011:bcr0720114510.

277. O'Quin J. *Dipylidium caninum*: A potential zoonosis easily missed on routine fecal diagnostics. 2023 [cited 2024 Jul 3]. Available from: https://www.cliniciansbrief.com/article/dipylidium-caninum-potential-zoonosis-easily-missed-routine-fecal-diagnostics

278. Yevstafieva V, Horb K, Melnychuk V, Bakhur T, Feshchenko D. ectoparasites in the composition of mixed infestations in domestic dogs from Poltava, Ukraine. Folia Vet. 2020 Sep 1;64(3):47–53.

279. Papajová I, Pipiková J, Papaj J, Čižmár A. Parasitic contamination of urban and rural environments in the Slovak Republic: dog's excrements as a source. Helminthologia. 2014 Dec 1;51(4):273–80.

280. Felsmann M, Michalski M, Felsmann M, Sokół R, Szarek J, Strzyżewska-Worotyńska E. Invasive forms of canine endoparasites as a potential threat to public health – A review and own studies. Ann Agric Environ Med. 2017 May 11;24(2):245–9.

281. Moskvina TV, Ermolenko AV. Helminth infections in domestic dogs from Russia. Vet World. 2016 Nov;9(11):1248–58.

282. Palmer CS, Robertson ID, Traub RJ, Rees R, Andrew Thompson RC. Intestinal parasites of dogs and cats in Australia: The veterinarian's perspective and pet owner awareness. Vet J. 2010 Mar 1;183(3):358–61.

283. Shin JW, Liao WT. Humoral immune response to *Dipylidium caninum* infection of stray dogs in Taiwan. Vet Parasitol. 2002 Apr 2;104(4):351–6.

284. Othman RA, Abuseir S. The prevalence of gastrointestinal parasites in native dogs in Palestine. Iran J Parasitol. 2021;16(3):435–42.

285. Gutema FD, Yohannes GW, Abdi RD, Abuna F, Ayana D, Waktole H, et al. *Dipylidium caninum* infection in dogs and humans in Bishoftu Town, Ethiopia. Diseases. 2020 Dec 22;9(1):1.

286. Khan W, Nisa NN, Ullah S, Ahmad S, Mehmood SA, Khan M, et al. Gastrointestinal helminths in dog feces surrounding suburban areas of Lower Dir district, Pakistan: A public health threat. Braz J Biol. 2019 Oct 21;80:511–7.

287. Ilić T, Nišavić U, Gajić B, Nenadović K, Ristić M, Stanojević D, et al. Prevalence of intestinal parasites in dogs from public shelters in Serbia. Comp Immunol Microbiol Infect Dis. 2021 Jun 1;76:101653.

288. Rabbani IAR, Mareta FJ, Kusnoto, Hastutiek P, Lastuti NDR, Mufasirin, et al. Zoonotic and other gastrointestinal parasites in cats in Lumajang, East Java, Indonesia. Infect Dis Rep. 2020 Jul 7;12(Suppl 1):8747.

289. Rousseau J, Castro A, Novo T, Maia C. *Dipylidium caninum* in the twenty-first century: epidemiological studies and reported cases in companion animals and humans. Parasit Vectors. 2022 May 10;15(1):131.

290. Centers for Disease Control and Prevention. *Dipylidium caninum*. 2019 [cited 2025 Mar 24]. Available from: https://www.cdc.gov/dpdx/dipylidium/index.html

291. Nelson CT. 111 - Heartworm and Related Nematodes. In: Sykes JE, editor. Greene's Infectious Diseases of the Dog and Cat (Fifth Edition). Philadelphia: W.B. Saunders; 2021 [cited 2025 Feb 21]. p. 1399–417. Available from: https://www.sciencedirect.com/science/article/pii/B9780323509343001117

292. Scavo NA, Zecca IB, Sobotyk C, Saleh MN, Lane SK, Olson MF, et al. High prevalence of canine heartworm, *Dirofilaria immitis*, in pet dogs in south Texas, USA, with evidence of *Aedes aegypti* mosquitoes contributing to transmission. Parasit Vectors. 2022 Nov 3;15(1):407.

293. Sacks BN. Increasing prevalence of canine heartworm in coyotes from California. J Wildl Dis. 1998 Apr;34(2):386–9.

294. McTier TL, McCall JW, Dzimianski MT. Epidemiology of heartworm infection in beagles naturally exposed to infection in three southeastern states. In: Proceedings of the Heartworm Symposium '92. American Heartworm Society; 1992. p. 47–57.

295. Paliy AP, Sumakova NV, Pavlichenko OV, Palii AP, Reshetylo OI, Kovalenko LM, et al. Monitoring of animal dirofilariosis incidence in Kharkiv Region of Ukraine. zoodiversity. 2022;56(2):153–64.

296. Zumaquero L, Simón F, Carretón E, Hernández I, Sandoval C, Morchón R. Prevalence of canine and human dirofilariosis in Puebla, Mexico. Vet Parasitol. 2020 Jun;282:109098.

297. Alsarraf M, Carretón E, Ciuca L, Diakou A, Dwużnik-Szarek D, Fuehrer HP, et al. Diversity and geographic distribution of haplotypes of *Dirofilaria immitis* across European endemic countries. Parasit Vectors. 2023 Sep 12;16(1):325.

298. Martina M, Zuzana H, Daniela V, Lenka B. Different epidemiological pattern of canine dirofilariosis in two neighboring countries in Central Europe—the Czech Republic and Slovakia. Parasitol Res. 2021 Feb;120(2):547–52.

299. Széll Z, Bacsadi Á, Szeredi L, Nemes C, Fézer B, Bakcsa E, et al. Rapid spread and emergence of heartworm resulting from climate and climate-driven ecological changes in Hungary. Vet Parasitol. 2020 Apr;280:109067.

300. Song KH, Park JE, Lee DH, Lee SH, Shin HJ. Serological update and molecular characterization of *Dirofilaria immitis* in dogs, South Korea. Res Vet Sci. 2010 Jun;88(3):467–9.

301. Alsarraf M, Levytska V, Mierzejewska EJ, Poliukhovych V, Rodo A, Alsarraf M, et al. Emerging risk of *Dirofilaria* spp. infection in Northeastern Europe: high prevalence of *Dirofilaria repens* in sled dog kennels from the Baltic countries. Sci Rep. 2021 Jan 13;11(1):1068.

302. Moskvina TV, Ermolenko AV. Dirofilariasis in Russian Federation: a big problem with large distribution. Russ Open Med J. 2018 Feb 22;7(1):e0102.

303. Noack S, Harrington J, Carithers DS, Kaminsky R, Selzer PM. Heartworm disease – Overview, intervention, and industry perspective. Int J Parasitol Drugs Drug Resist. 2021 Aug;16:65–89.

304. Lu TL, Wong JY, Tan TL, Hung YW. Prevalence and epidemiology of canine and feline heartworm infection in Taiwan. Parasit Vectors. 2017 Nov 9;10(2):484.

305. Centers for Disease Control and Prevention. Dirofilariasis. 2019 [cited 2025 Feb 19]. Available from: https://www.cdc.gov/dpdx/dirofilariasis/index.html

306. Nelson CT, McCall JW, Jones S, Moorhead A. Current canine guidelines for the prevention, diagnosis, and management of heartworm (*Dirofilaria immitis*) infection in dogs. American Heartworm Society; 2018 [cited 2025 Feb 18]. Available from: https://www.heartwormsociety.org/images/pdf/2018-AHS-Canine-Guidelines.pdf

307. Demirci B, Bedir H, Taskin Tasci G, Vatansever Z. Potential mosquito vectors of *Dirofilaria immitis* and *Dirofilaira repens* (Spirurida: Onchocercidae) in Aras Valley, Turkey. Healy K, editor. J Med Entomol. 2021 Mar 12;58(2):906–12.

308. Eckert J, Deplazes P. Biological, epidemiological, and clinical aspects of Echinococcosis, a zoonosis of increasing concern. Clin Microbiol Rev. 2004 Jan;17(1):107–35.

309. Romig T. Epidemiology of echinococcosis. Langenbecks Arch Surg. 2003 Sep 1;388(4):209–17.

310. Merck Veterinary Manual. [cited 2024 Jul 3]. Zoonotic Diseases: Parasitic Diseases. Available from: https://www.merckvetmanual.com/multimedia/table/zoonotic-diseases-parasitic-diseases

311. D'Alessandro A, Rausch RL. New aspects of neotropical polycystic (*Echinococcus vogeli*) and unicystic (*Echinococcus oligarthrus*) Echinococcosis. Clin Microbiol Rev. 2008 Apr;21(2):380–401.

312. Casulli A, Massolo A, Saarma U, Umhang G, Santolamazza F, Santoro A. Species and genotypes belonging to *Echinococcus granulosus* sensu lato complex causing human cystic echinococcosis in Europe (2000–2021): a systematic review. Parasit Vectors. 2022 Mar 28;15(1):109.

313. das Neves LB, Teixeira PEF, Silva S, de Oliveira FB, Garcia DD, de Almeida FB, et al. First molecular identification of *Echinococcus vogeli* and *Echinococcus granulosus* (sensu stricto) G1 revealed in feces of domestic dogs (Canis familiaris) from Acre, Brazil. Parasit Vectors. 2017 Jan 14;10(1):28.

314. Spickler AR. The Center for Food Security and Public Health. 2020 [cited 2024 Jul 3]. Echinococcosis. Available from: https://www.cfsph.iastate.edu/Factsheets/pdfs/echinococcosis.pdf

315. Centers for Disease Control and Prevention. Echinococcosis. 2019 [cited 2024 Jul 3]. Available from: https://www.cdc.gov/dpdx/echinococcosis/index.html

316. Centers for Disease Control and Prevention. Echinococcosis. 2019 [cited 2025 Feb 19]. Available from: https://www.cdc.gov/dpdx/echinococcosis/index.html

317. Organisation mondiale de la santé, Office international des épizooties, editors. WHO-OIE manual on echinococcosis in human and animals: a public health problem of global concern. Paris: OIE; 2001.

318. Souza L, Sampaio R, Gomes AP, Morato R, Chiarello A, Souza L, et al. Occurrence of potential wild hosts of *Echinococcus vogeli* in the forests of southwestern Brazilian Amazonia. Biota Neotropica. 2022 Sep 30;22.

319. Tamarozzi F, Legnardi M, Fittipaldo A, Drigo M, Cassini R. Epidemiological distribution of *Echinococcus granulosus* s.l. infection in human and domestic animal hosts in European

Mediterranean and Balkan countries: A systematic review. Torgerson PR, editor. PLoS Negl Trop Dis. 2020 Aug 10;14(8):e0008519.

320. Public Health Agency of Canada. Pathogen Safety Data Sheets: Infectious Substances – *Echinococcus granulosus*. 2014 [cited 2025 Feb 19]. Available from: https://www.canada.ca/en/public-health/services/laboratory-biosafety-biosecurity/pathogen-safety-data-sheets-risk-assessment/echinococcus-granulosus-pathogen-safety-data-sheet.html

321. Moro P, Schantz PM. Cystic echinococcosis in the Americas. Parasitol Int. 2006 Jan;55:S181–6.

322. Šnábel V, Kuzmina TA, Antipov AA, Yemets OM, Cavallero S, Miterpáková M, et al. Molecular study of *Echinococcus granulosus* cestodes in Ukraine and the first genetic identification of *Echinococcus granulosus* sensu stricto (G1 Genotype) in the country. Acta Parasitol. 2022 Mar;67(1):244–54.

323. Dán Á, Rónai Z, Széll Z, Sréter T. Prevalence and genetic characterization of *Echinococcus* spp. in cattle, sheep, and swine in Hungary. Parasitol Res. 2018 Sep 1;117(9):3019–22.

324. Maravilla P, Andrew Thompson RC, Palacios-Ruiz JA, Estcourt A, Ramirez-Solis E, Mondragon-de-la-Peña C, et al. *Echinococcus granulosus* cattle strain identification in an autochthonous case of cystic echinococcosis in central Mexico. Acta Trop. 2004 Nov;92(3):231–6.

325. Villalobos N, González LM, Morales J, De Aluja AS, Jiménez MI, Blanco MA, et al. Molecular identification of *Echinococcus granulosus* genotypes (G1 and G7) isolated from pigs in Mexico. Vet Parasitol. 2007 Jun;147(1–2):185–9.

326. Balog T, Nagy G, Halász T, Csányi E, Zomborszky Z, Csivincsik Á. The occurrence of *Echinococcus* spp. in golden jackal (*Canis aureus*) in southwestern Hungary: Should we need to rethink its expansion? Parasitol Int. 2021 Feb 1;80:102214.

327. Yoo JS, Kang MK, Park JG, Kim HJ, Choi JH. Clinical implications for the comprehensive interpretation of radiologic and immunodiagnostic tests in patients suspected of parasitic hepatic cyst, a rare case in Korea. Trop Med Infect Dis. 2023 Mar 2;8(3):155.

328. Sałamatin R, Kowal J, Nosal P, Kornaś S, Cielecka D, Jańczak D, et al. Cystic echinococcosis in Poland: genetic variability and the first record of *Echinococcus granulosus* sensu stricto (G1 genotype) in the country. Parasitol Res. 2017 Nov;116(11):3077–85.

329. Dybicz M, Borkowski PK, Dąbrowska J, Chomicz L. Cases of *Echinococcus granulosus* sensu stricto isolated from Polish patients: imported or indigenous? BioMed Res Int. 2015;2015:1–5.

330. Konyaev SV, Yanagida T, Nakao M, Ingovatova GM, Shoykhet YN, Bondarev AY, et al. Genetic diversity of *Echinococcus* spp. in Russia. Parasitology. 2013 Nov;140(13):1637–47.

331. Otero-Abad B, Torgerson PR. A systematic review of the epidemiology of Echinococcosis in domestic and wild animals. PLoS Negl Trop Dis. 2013 Jun 6;7(6):e2249.

332. Jenkins D, Mckinlay A, Duolong H, Bradshaw H, Craig P. Detection of *Echinococcus granulosus* coproantigens in faeces from naturally infected rural domestic dogs in south eastern Australia. Aust Vet J. 2006 Jan;84(1–2):12–6.

333. Yuan W, Lee R, Chou Y, Chiang J, Chen Y, Hsu H. Hydatid cyst of the liver: A case report and literature review. Kaohsiung J Med Sci. 2005 Sep;21(9):418–23.

334. Avcioglu H, Guven E, Balkaya I, Kirman R, Akyuz M, Mebarek Bia M, et al. The situation of echinococcosis in stray dogs in Turkey: the first finding of *Echinococcus multilocularis* and *Echinococcus ortleppi*. Parasitology. 2021 Aug;148(9):1092–8.

335. Victoria Department of Health. Hydatid disease (echinococcosis). State Government of Victoria, Australia; 2022 [cited 2025 Feb 19]. Available from: https://www.health.vic.gov.au/infectious-diseases/hydatid-disease-echinococcosis

336. Enemark HL, Thamsborg SM. The Spread of *Echinococcus multilocularis* and Its Effects. In: World Small Animal Veterinary Association Congress Proceedings, 2017. 2017.

337. Dezsényi B, Dubóczki Z, Strausz T, Csulak E, Czoma V, Káposztás Z, et al. Emerging human alveolar echinococcosis in Hungary (2003–2018): a retrospective case series analysis from a multi-centre study. BMC Infect Dis. 2021 Feb 10;21(1):168.

338. Denstedt E. *Echinococcus multilocularis* as an emerging public health threat in Canada: A knowledge synthesis and needs assessment. University of Guelph; 2017.

339. Zajac A, Fairman D, McGee E, Wells B, Peregrine A, Jenkins E, et al. Alveolar echinococcosis in a dog in the eastern United States. J Vet Diagn Invest. 2020 Sep;32(5):742–6.

340. Finnish Food Authority. Echinococcus risk from Ukranian pets. 2022 [cited 2025 Feb 19]. Available from: https://www.ruokavirasto.fi/en/animals/animal-health-and-diseases/animal-diseases/diseases-common-to-several-animal-species/echinococcus/echinococcus-risk-from-ukranian-pets/

341. Wen H, Vuitton L, Tuxun T, Li J, Vuitton DA, Zhang W, et al. Echinococcosis: Advances in the 21st Century. Clin Microbiol Rev. 2019 Mar 20;32(2):e00075-18.

342. Oksanen A, Siles-Lucas M, Karamon J, Possenti A, Conraths FJ, Romig T, et al. The geographical distribution and prevalence of *Echinococcus multilocularis* in animals in the European Union and adjacent countries: a systematic review and meta-analysis. Parasit Vectors. 2016 Sep 28;9(1):519.

343. Torgerson PR, Keller K, Magnotta M, Ragland N. The global burden of alveolar echinococcosis. Brooker S, editor. PLoS Negl Trop Dis. 2010 Jun 22;4(6):e722.

344. Jeong JS, Han SY, Kim YH, Sako Y, Yanagida T, Ito A, et al. Serological and molecular characteristics of the first Korean case of *Echinococcus multilocularis*. Korean J Parasitol. 2013 Oct 31;51(5):595–7.

345. Umhang G, Karamon J, Hormaz V, Knapp J, Cencek T, Boué F. A step forward in the understanding of the presence and expansion of *Echinococcus multilocularis* in Eastern Europe using microsatellite EmsB genotyping in Poland. Infect Genet Evol. 2017 Oct;54:176–82.

346. Makarikov AA, Dokuchaev NE. Tapeworms in rodents from the Lower Anabar River Basin, with a review of species diversity of cestodes in Yakutia, Russia. Acta Parasitol. 2021 Sep 1;66(3):1012–20.

347. Baumann S, Shi R, Liu W, Bao H, Schmidberger J, Kratzer W, et al. Worldwide literature on epidemiology of human alveolar echinococcosis: a systematic review of research published in the twenty-first century. Infection. 2019 Oct 1;47(5):703–27.

348. Cai H, Zhang J, Zhang X, Guan Y, Ma X, Cao J, et al. Prevalence of *Echinococcus* species in wild foxes and stray dogs in Qinghai Province, China. Am J Trop Med Hyg. 2022 Feb 2;106(2):718–23.

349. Weese JS, Evason M, editors. A Color Handbook: Infectious Diseases of the Dog and Cat. 1st ed. CRC Press; 2019 [cited 2025 Feb 19]. Available from: https://www.taylorfrancis.com/books/9781498775533

350. Ministry of Health and Long-Term Care. Management of *Echinococcus multilocularis* infections in animals guideline, 2019. 2019 [cited 2025 Feb 19]. Available from: https://www.rcdhu.com/wp-content/uploads/2019/04/Management-of-EM-Infections-in-Animals-2019.pdf

351. Deplazes P, Rinaldi L, Alvarez Rojas CA, Torgerson PR, Harandi MF, Romig T, et al. Global distribution of alveolar and cystic echinococcosis. In: Advances in Parasitology. Elsevier; 2017 [cited 2025 Feb 19]. p. 315–493. Available from: https://linkinghub.elsevier.com/retrieve/pii/S0065308X1630104X

352. Companion Animal Parasite Council. *Filaroides hirthi*. 2018 [cited 2024 Jul 4]. Available from: https://capcvet.org/guidelines/filaroides-hirthi/

353. Morelli S, Diakou A, Di Cesare A, Colombo M, Traversa D. Canine and feline parasitology: Analogies, differences, and relevance for human health. Clin Microbiol Rev. 2021 Aug 11;34(4):e00266-20.

354. Western College of Veterinary Medicine. 2021 [cited 2024 Jul 4]. *Oslerus (Filaroides) osleri*. Available from: https://wcvm.usask.ca/learnaboutparasites/parasites/oslerus-filaroides-osleri.php

355. Yao C, O'Toole D, Driscoll M, McFarland W, Fox J, Cornish T, et al. *Filaroides osleri* (*Oslerus osleri*): Two case reports and a review of canid infections in North America. Vet Parasitol. 2011 Jun 30;179(1):123–9.

356. Husnik R, Just R, Weidenhöfer T, Kralova-Kovarikova S, Jurankova J, Koudela B. First autochtonous infection of a dog with *Oslerus (Filaroides) osleri* in the Czech Republic. Schweiz Arch Tierheilkd. 2016 Feb 5;158(2):115–20.

357. Dunsmore JD, Spratt DM. The life history of *Filaroides osleri* in wild and domestic canids in Australia. Vet Parasitol. 1979 Aug 1;5(2):275–86.

358. Bowman D. DVM360. 2009 [cited 2024 Jul 4]. Canine and feline lungworms (Proceedings). Available from: https://www.dvm360.com/view/canine-and-feline-lungworms-proceedings

359. Martínez-Rondán FJ, Ruiz de Ybáñez MR, López-Beceiro AM, Fidalgo LE, Berriatua E, Lahat L, et al. Cardiopulmonary nematode infections in wild canids: Does the key lie on host-prey-parasite evolution? Res Vet Sci. 2019 Oct 1;126:51–8.

360. Robertson LJ. Giardiasis in animals. 2021 [cited 2024 Jul 4]. Available from: https://www.merckvetmanual.com/digestive-system/giardiasis-giardia/giardiasis-in-animals

361. Western College of Veterinary Medicine. 2021 [cited 2024 Jul 4]. *Giardia* species. Available from: https://wcvm.usask.ca/learnaboutparasites/parasites/giardia-species.php

362. Carlin EP, Bowman DD, Scarlett JM. Prevalence of *Giardia* in symptomatic dogs and cats in the United States. Vet Ther. 2006;7(3):199–206.

363. Munoz J, Mayer DCG. *Toxoplasma gondii* and *Giardia duodenalis* infections in domestic dogs in New York City public parks. Vet J. 2016 May 1;211:97–9.

364. Szabová-Mikolajová E, Juris P, Miterpakova M, Antolová D, Papajová I, Šefčíková H. Prevalence of important zoonotic parasites in dog populations from the Slovak Republic. Helminthologia. 2007 Dec 1;44:170–6.

365. Godínez-Galaz EM, Veyna-Salazar NP, Olvera-Ramírez AM, Milián-Suazo F, Perea-Razo CA, Bernal-Reynaga R, et al. Prevalence and zoonotic potential of *Giardia intestinalis* in dogs of the central region of Mexico. Animals. 2019 Jun;9(6):325.

366. Kim HY, Lee H, Lee SH, Seo MG, Yi S, Kim JW, et al. Multilocus genotyping and risk factor analysis of *Giardia duodenalis* in dogs in Korea. Acta Trop. 2019 Nov 1;199:105113.

367. Liu J, Lee SE, Song KH. Prevalence of canine giardiosis in South Korea. Res Vet Sci. 2008 Jun 1;84(3):416–8.

368. Tseng YC, Ho GD, Chen T TW, Huang BF, Cheng PC, Chen JL, et al. Prevalence and genotype of *Giardia duodenalis* from faecal samples of stray dogs in Hualien city of eastern Taiwan. Trop Biomed. 2014 Jun;31(2):305–11.

369. Vincent-Johnson N, Baneth G, Allen KE. 99 - Hepatozoonosis. In: Sykes JE, editor. Greene's Infectious Diseases of the Dog and Cat (Fifth Edition). Philadelphia: W.B. Saunders; 2021 [cited 2025 Feb 21]. p. 1230–47. Available from: https://www.sciencedirect.com/science/article/pii/B9780323509343000999

370. Li Y, Wang C, Allen KE, Little SE, Ahluwalia SK, Gao D, et al. Diagnosis of canine *Hepatozoon* spp. infection by quantitative PCR. Vet Parasitol. 2008 Oct 20;157(1):50–8.

371. Allen KE, Li Y, Kaltenboeck B, Johnson EM, Reichard MV, Panciera RJ, et al. Diversity of *Hepatozoon* species in naturally infected dogs in the southern United States. Vet Parasitol. 2008 Jul 4;154(3):220–5.

372. Rodríguez-Alarcón CA, Figueroa-Millán JV, Lom-Monárrez C, Martínez-Baldón NG, Martín-Orozco U, Adame-Gallegos JR, et al. First report of *Hepatozoon* spp.1 in a dog at the Paso del Norte Region, US-Mexico. Southwest Entomol. 2021 Jan;45(4):1039–44.

373. Bajer A, Alsarraf M, Topolnytska M, Tołkacz K, Dwużnik-Szarek D, Rodo A. Vector-borne parasites in dogs from Ukraine translocated to Poland following Russian invasion in 2022. Parasit Vectors. 2023 Nov 21;16(1):430.

374. Jarquín-Díaz VH, Barbachano-Guerrero A, Maldonado-Rodríguez R, Vásquez-Aguilar AA, Aguilar-Faisal JL. First molecular evidence of *Hepatozoon canis* in domestic dogs and ticks in fragmented rainforest areas in Mexico. Vet Parasitol Reg Stud Rep. 2016 Dec 1;6:4–8.

375. Miterpáková M, Komjáti-Nagyová M, Hurníková Z, Víchová B. Retrospective molecular study on canine hepatozoonosis in Slovakia – Does infection risk for dogs really exist? Ticks Tick-Borne Dis. 2017 Jun 1;8(4):567–73.

376. Hornok S, Tánczos B, Fernández de Mera IG, de la Fuente J, Hofmann-Lehmann R, Farkas R. High prevalence of *Hepatozoon*-infection among shepherd dogs in a region considered to be free of *Rhipicephalus sanguineus*. Vet Parasitol. 2013 Sep 1;196(1):189–93.

377. Kwon SJ, Kim YH, Oh HH, Choi US. First Case of canine infection with *Hepatozoon canis* (Apicomplexa: Haemogregarinidae) in the Republic of Korea. Korean J Parasitol. 2017 Oct 31;55(5):561–4.

378. Tołkacz K, Kretschmer M, Nowak S, Mysłajek RW, Alsarraf M, Wężyk D, et al. The first report on *Hepatozoon canis* in dogs and wolves in Poland: clinical and epidemiological features. Parasit Vectors. 2023 Sep 4;16(1):313.

379. Hii S, Traub R, Thompson M, Henning J, O'Leary C, Burleigh A, et al. Canine tick-borne pathogens and associated risk factors in dogs presenting with and without clinical signs consistent with tick-borne diseases in northern Australia. Aust Vet J. 2015;93(3):58–66.

380. Greay TL, Barbosa AD, Rees RL, Paparini A, Ryan UM, Oskam CL, et al. An Australian dog diagnosed with an exotic tick-borne infection: should Australia still be considered free from *Hepatozoon canis*? Int J Parasitol. 2018 Sep 1;48(11):805–15.

381. Chao LL, Liao HT, Shih CM. First detection and genetic identification of *Hepatozoon canis* in *Rhipicephalus sanguineus* sensu lato ticks collected from dogs of Taiwan. Ticks Tick-Borne Dis. 2019 Jun 1;10(4):929–34.

382. Clow KM. Veterinary Practice News. 2021 [cited 2025 Mar 26]. The brown dog tick: An unwelcome visitor. Available from: https://www.veterinarypracticenews.ca/the-brown-dog-tick-an-unwelcome-visitor/

383. Baniya A, Goldy CJ, Ardpairin J, Achi P, Chang YW, Adrianza RC, et al. Canine schistosomiasis in the west coast: *Heterobilharzia americana i*n two natural intermediate hosts found in the Colorado River, California. Pathogens. 2024 Mar;13(3):245.

384. Saito T. First record of the non-indigenous freshwater snail *Galba humilis* (Say, 1822) (Mollusca: Hygrophila: Lymnaeidae) in Japan. BioInvasions Rec. 2022;11(2):428–39.

385. Schniebs K, Glöer P, Quiñonero-Salgado S, Lopez-Soriano J, Hundsdoerfer AK. The first record of *Galba cubensis* (L. Pfeiffer, 1839) (Mollusca: Gastropoda: Lymnaeidae) from open fields of Europe. Folia Malacol. 2018 Mar 6;26(1):3–15.

386. Ngcamphalala PI, Malatji MP, Mukaratirwa S. Geography and ecology of invasive *Pseudosuccinea columella* (Gastropoda: Lymnaeidae) and implications in the transmission of *Fasciola* species (Digenea: Fasciolidae) – a review. J Helminthol. 2022 [cited 2024 Sep 17];96. Available from: https://www.cambridge.org/core/product/identifier/S0022149X21000717/type/journal_article

387. Johnson E. Canine schistosomiasis in North America: an underdiagnosed disease with an expanding distribution. Compendium. 2010 Mar 1;32(3):E1-4.

388. Devora P, Vatta A, Liu CC. Prevalence of schistosomiasis in asymptomatic dogs in Southeastern Louisiana, United States. J Vet Sci Anim Husb. 2024 Mar 5;11(1):1138.

389. Rodriguez JY, Lewis BC, Snowden KF. Distribution and characterization of *Heterobilharzia americana* in dogs in Texas. Vet Parasitol. 2014 Jun 16;203(1):35–42.

390. McKown RD, Veatch JK, Fox LB. New locality record for *Heterobilharzia americana*. J Wildl Dis. 1991 Jan;27(1):156–60.

391. Kelley S. Heterobilharziasis (Trematoda: Schistosomatidae) in raccoons (*Procyon lotor*) of north-central Texas. Tex J Sci. 2010 Feb 1;62:41–8.

392. Companion Animal Parasite Council. Schistosomiasis. 2013 [cited 2024 Sep 17]. Available from: https://capcvet.org/guidelines/schistosomiasis/

393. Baneth G, Petersen C, Solano-Gallego L, Sykes JE. 96 - Leishmaniosis. In: Sykes JE, editor. Greene's Infectious Diseases of the Dog and Cat (Fifth Edition). Philadelphia: W.B. Saunders; 2021 [cited 2025 Feb 21]. p. 1179–202. Available from: https://www.sciencedirect.com/science/article/pii/B9780323509343000963

394. Petersen CA, Barr SC. Canine leishmaniasis in North America: Emerging or newly recognized? Vet Clin North Am Small Anim Pract. 2009 Nov;39(6):1065–74.

395. Curtin JM, Aronson NE. Leishmaniasis in the United States: Emerging issues in a region of low endemicity. Microorganisms. 2021 Mar 11;9(3):578.

396. Mihalca AD, Cazan CD, Sulesco T, Dumitrache MO. A historical review on vector distribution and epidemiology of human and animal leishmanioses in Eastern Europe. Res Vet Sci. 2019 Apr;123:185–91.

397. Arjona-Jiménez G, Villegas N, López-Céspedes Á, Marín C, Longoni SS, Bolio-González ME, et al. Prevalence of antibodies against three species of *Leishmania* (*L. mexicana, L. braziliensis, L. infantum*) and possible associated factors in dogs from Mérida, Yucatán, Mexico. Trans R Soc Trop Med Hyg. 2012 Apr;106(4):252–8.

398. Marcondes M, Day MJ. Current status and management of canine leishmaniasis in Latin America. Res Vet Sci. 2019 Apr;123:261–72.

399. Bhang DH, Choi US, Kim HJ, Cho KO, Shin SS, Youn HJ, et al. An autochthonous case of canine visceral leishmaniasis in Korea. Korean J Parasitol. 2013 Oct 31;51(5):545–9.

400. Liu G, Wu Y, Wang L, Liu Y, Huang W, Li Y, et al. Re-emergence of canine *Leishmania infantum* infection in mountain areas of Beijing. One Health Adv. 2023 Mar 30;1(1):11.

401. Strelkova MV, Ponirovsky EN, Morozov EN, Zhirenkina EN, Razakov SA, Kovalenko DA, et al. A narrative review of visceral leishmaniasis in Armenia, Azerbaijan, Georgia, Kazakhstan, Kyrgyzstan, Tajikistan, Turkmenistan, Uzbekistan, the Crimean Peninsula and Southern Russia. Parasit Vectors. 2015 Jun 16;8(1):330.

402. Zhdanova, Mangchanti, Nardone, Akulinina, Napisanova. Leishmaniasis in Russia and Italy. Some questions of screening and prophylaxis. Theory Pract Parasit Dis Control. 2019 May 14;(20):226–30.

403. New South Wales Department of Primary Industries. Leishmaniasis. New South Wales Department of Primary Industries; 2017. Available from: https://www.dpi.nsw.gov.au/__data/assets/pdf_file/0007/596833/Leishmaniasis-.pdf

404. Spickler AR. The Center for Food Security and Public Health. 2022 [cited 2025 Feb 20]. Leishmaniasis (Cutaneous and Visceral). Available from: https://www.cfsph.iastate.edu/Factsheets/pdfs/leishmaniasis.pdf

405. Williams K, Llera R, Ward E. VCA. 2022 [cited 2025 Feb 20]. Leishmaniasis in Dogs | VCA Animal Hospitals. Available from: https://vcahospitals.com/know-your-pet/leishmaniasis-in-dogs

406. Sykes JE. 47 - Neorickettsiosis. In: Sykes JE, editor. Greene's Infectious Diseases of the Dog and Cat (Fifth Edition). Philadelphia: W.B. Saunders; 2021 [cited 2025 Feb 21]. p. 571–81. Available from: https://www.sciencedirect.com/science/article/pii/B9780323509343000471

407. Western College of Veterinary Medicine. 2021 [cited 2024 Sep 18]. *Nanophyetus salmincola*. Available from: https://wcvm.usask.ca/learnaboutparasites/parasites/nanophyetus-salmincola.php

408. Sykes J e., Marks S l., Mapes S, Schultz R m., Pollard R e., Tokarz D, et al. Salmon poisoning disease in dogs: 29 C=cases. J Vet Intern Med. 2010;24(3):504–13.

409. LA County Department of Public Health. [cited 2024 Sep 18]. Available from: http://publichealth.lacounty.gov/vet/spd.htm

410. Voronova AN, Chelomina GN, Besprozvannykh VV, Tkach VV. Genetic divergence of human pathogens *Nanophyetus* spp. (Trematoda: Troglotrematidae) on the opposite sides of the Pacific Rim. Parasitology. 2017 Apr;144(5):601–12.

411. McAllister MM. Merck Veterinary Manual. 2020 [cited 2025 Feb 20]. Neosporosis in Animals - Infectious Diseases. Available from: https://www.merckvetmanual.com/infectious-diseases/neosporosis/neosporosis-in-animals

412. Western College of Veterinary Medicine. 2021 [cited 2025 Feb 20]. *Neospora caninum*. Available from: https://wcvm.usask.ca/learnaboutparasites/parasites/neospora-caninum.php

413. Dubey JP, Schares G, Ortega-Mora LM. Epidemiology and control of neosporosis and *Neospora caninum*. Clin Microbiol Rev. 2007 Apr;20(2):323–67.

414. Cheadle MA, Lindsay DS, Blagburn BL. Prevalence of antibodies to *Neospora caninum* in dogs. Vet Parasitol. 1999 Sep 1;85(4):325–30.

415. Reiterová K, Špilovská S, Čobádiová A, Hurníková Z. Prevalence of *Toxoplasma gondii* and *Neospora caninum* in red foxes in Slovakia. Acta Parasitol. 2016 Dec 1;61(4):762–8.

416. Hedmegová M, Pošivák J, Pošiváková S, Reiterová K. Prevalence of neosporosis in dogs in Eastern Slovakia. Folia Vet. 2011;55(2):25–6.

417. Kim JH, Kang MS, Lee BC, Hwang WS, Lee CW, So BJ, et al. Seroprevalence of antibodies to *Neospora caninum* in dogs and raccoon dogs in Korea. Korean J Parasitol. 2003 Dec 20;41(4):243–5.

418. Goździk K, Wrzesień R, Wielgosz-Ostolska A, Bień J, Kozak-Ljunggren M, Cabaj W. Prevalence of antibodies against *Neospora caninum* in dogs from urban areas in Central Poland. Parasitol Res. 2011 Apr;108(4):991–6.

419. Animal Health Ireland. *Neospora caninum*. Animal Health Ireland; 2021. Available from: https://animalhealthireland.ie/assets/uploads/2021/04/AHI-Parasite-Control-Neospora-2021.pdf?dl=1

420. Deak G, Toader S, Soare DG, Ionică AM, Taulescu M, Mihalca AD. Case report: A new geographic area for the presence of the zoonotic ocular nematode, *Onchocerca lupi* in Romania. Front Vet Sci. 2022 Jul 8 [cited 2024 Sep 26];9. Available from: https://www.frontiersin.org/journals/veterinary-science/articles/10.3389/fvets.2022.941303/full

421. Verocai GG, Conboy G, Lejeune M, Marron F, Hanna P, MacDonald E, et al. *Onchocerca lupi* nematodes in dogs exported from the United States into Canada. Emerg Infect Dis. 2016 Aug;22(8):1477–9.

422. Companion Animal Parasite Council. *Onchocerca lupi*. 2018 [cited 2024 Sep 18]. Available from: https://capcvet.org/guidelines/onchocerca-lupi/

423. Bowers Wu D, Ko B, Lopez Hernandez G, Botros J, Spader H, Sapp S, et al. Neuroinvasive *Onchocerca lupi* infection in a ten-year-old girl. Case Rep Infect Dis. 2022;2022(1):9773058.

424. Unterköfler MS, Huck A, Silbermayr K, Fuehrer HP. Autochthonous *Onchocerca lupi* infection of a domestic dog in Austria. Parasit Vectors. 2023 Feb 1;16(1):46.

425. Rojas A, Morales-Calvo F, Salant H, Otranto D, Baneth G. Zoonotic ocular onchocercosis by *Onchocerca lupi*. Yale J Biol Med. 2021 Jun 30;94(2):331–41.

426. Tahir D, Davoust B, Parola P. Vector-borne nematode diseases in pets and humans in the Mediterranean Basin: An update. Vet World. 2019 Oct;12(10):1630–43.

427. Otranto D, Dantas-Torres F, Brianti E, Traversa D, Petrić D, Genchi C, et al. Vector-borne helminths of dogs and humans in Europe. Parasit Vectors. 2013 Dec;6(1):16.

428. Merck Manual Professional Edition. [cited 2024 Sep 27]. Opisthorchiasis - Infectious Diseases. Available from: https://www.merckmanuals.com/en-ca/professional/infectious-diseases/trematodes-flukes/opisthorchiasis

429. Merck Manual Professional Edition. [cited 2024 Sep 27]. Clonorchiasis - Infectious Diseases. Available from: https://www.merckmanuals.com/en-ca/professional/infectious-diseases/trematodes-flukes/clonorchiasis

430. Fried B, Abruzzi A. Food-borne trematode infections of humans in the United States of America. Parasitol Res. 2010 May 1;106(6):1263–80.

431. Mordvinov VA, Yurlova NI, Ogorodova LM, Katokhin AV. *Opisthorchis felineus* and *Metorchis bilis* are the main agents of liver fluke infection of humans in Russia. Parasitol Int. 2012 Mar 1;61(1):25–31.

432. Yossepowitch O, Gotesman T, Assous M, Marva E, Zimlichman R, Dan M. Opisthorchiasis from imported raw fish. Emerg Infect Dis. 2004 Dec;10(12):2122–6.

433. Miterpáková M, Hurníková Z, Antolová D, Dubinský P. Endoparasites of red fox (*Vulpes vulpes*) in the Slovak Republic with the emphasis on zoonotic species *Echinococcus multilocularis* and *Trichinella* spp. Helminthologia. 2009 Jun 1;46(2):73–9.

434. Schuster RK. Opisthorchiidosis--a review. Infect Disord Drug Targets. 2010 Oct;10(5):402–15.

435. Yoo WG, Sohn WM, Na BK. Current status of *Clonorchis sinensis* and clonorchiasis in Korea: epidemiological perspectives integrating the data from human and intermediate hosts. Parasitology. 2022 Sep;149(10):1296–305.

436. Pozio E, Armignacco O, Ferri F, Gomez Morales MA. *Opisthorchis felineus*, an emerging infection in Italy and its implication for the European Union. Acta Trop. 2013 Apr 1;126(1):54–62.

437. Tang ZL, Huang Y, Yu XB. Current status and perspectives of *Clonorchis sinensis* and clonorchiasis: epidemiology, pathogenesis, omics, prevention and control. Infect Dis Poverty. 2016 Dec;5(1):71.

438. Lin RQ, Tang JD, Zhou DH, Song HQ, Huang SY, Chen JX, et al. Prevalence of *Clonorchis sinensis* infection in dogs and cats in subtropical southern China. Parasit Vectors. 2011 Sep 19;4(1):180.

439. Enes JE, Wages AJ, Malone JB, Tesana S. Prevalence of *Opisthorchis viverrini* Infection in the Canine and Feline Hosts in Three Villages, Khon Kaen Province, Northeastern Thailand. Southeast Asian J Trop Med Public Health. 2010 Jan;41(1):36–42.

440. Centers for Disease Control and Prevention. Opisthorchiasis. 2019 [cited 2024 Sep 27]. Available from: https://www.cdc.gov/dpdx/opisthorchiasis/index.html

441. Quang NX, Tu D. Taxonomy and distribution of medically important snails of the genus Parafossarulus (Caenogastropoda , Rissooidea, Bithyniidae) in Vietnam. 2016 [cited 2024 Sep 27]. Available from: https://www.semanticscholar.org/paper/TAXONOMY-AND-DISTRIBUTION-OF-MEDICALLY-IMPORTANT-OF-Quang-Tu/253f3cc42cc3177ed1f19ffe48ed8cee17d3b5d0

442. R.M. Kipp, Benson AJ, Larson J, Fusaro A. USGS nonindigenous aquatic species database. 2019 [cited 2024 Sep 27]. *Bithynia tentaculata*. Available from: https://nas.er.usgs.gov/queries/FactSheet.aspx?SpeciesID=987

443. Namsanor J, Sithithaworn P, Kopolrat K, Kiatsopit N, Pitaksakulrat O, Tesana S, et al. Seasonal transmission of *Opisthorchis viverrini* sensu lato and a Lecithodendriid trematode species in *Bithynia siamensis goniomphalos* snails in Northeast Thailand. Am J Trop Med Hyg. 2015 Jul 8;93(1):87–93.

444. Miller SM, Mitchell MA. Chapter 4 - Ornamental fish. In: Mitchell MA, Tully TN, editors. Manual of Exotic Pet Practice. Saint Louis: W.B. Saunders; 2009 [cited 2024 Sep 27]. p. 39–72. Available from: https://www.sciencedirect.com/science/article/pii/B978141600119550007X

445. Procop GW. North American Paragonimiasis (caused by *Paragonimus kellicotti*) in the context of global Paragonimiasis. Clin Microbiol Rev. 2009 Jul;22(3):415–46.

446. Ontario Animal Health Network. Infosheet: *Paragonimus kellicotti* for veterinarians. 2022 [cited 2024 Sep 27]. Available from: https://www.oahn.ca/resources/paragonimus-kellicotti/

447. Johannesen E, Nguyen V. *Paragonimus kellicotti*: A lung infection in our own backyard. Case Rep Pathol. 2016;2016:2107372.

448. Merck Veterinary Manual. [cited 2024 Sep 27]. Lung flukes in dogs and cats. Available from: https://www.merckvetmanual.com/respiratory-system/respiratory-diseases-of-small-animals/lung-flukes-in-dogs-and-cats

449. Centers for Disease Control and Prevention. Paragonimiasis. 2024 [cited 2024 Sep 27]. Available from: https://www.cdc.gov/dpdx/paragonimiasis/index.html

450. Western College of Veterinary Medicine. 2021 [cited 2024 Sep 27]. *Physaloptera* species. Available from: https://wcvm.usask.ca/learnaboutparasites/parasites/physaloptera-species.php

451. Ketzis JK, Little SE, Wulcan JM. Miscellaneous Nematode Infections. In: Greene's Infectious Diseases of the Dog and Cat. Elsevier; 2021 [cited 2024 Sep 27]. p. 1485–504. Available from: https://linkinghub.elsevier.com/retrieve/pii/B9780323509343001166

452. Companion Animal Parasite Council. *Physaloptera* spp.. 2015 [cited 2024 Sep 27]. Available from: https://capcvet.org/guidelines/physaloptera-spp/

453. Cantó GJ, García MP, García A, Guerrero MJ, Mosqueda J. The prevalence and abundance of helminth parasites in stray dogs from the city of Queretaro in central Mexico. J Helminthol. 2011 Sep;85(3):263–9.

454. Komorová P, Sitko J, Špakulová M, Hurníková Z, Sałamatin R, Chovancová G. New data on helminth fauna of birds of prey (Falconiformes, Accipitriformes, Strigiformes) in the Slovak Republic. Helminthologia. 2017 Dec 1;54(4):314–21.

455. Quadros RMD, Marques SMT, Moura ABD, Antonelli M. Primeiro registro do nematoide Physaloptera praeputialis parasitando puma. Neotropical Biol Conserv. 2014 Sep 24;9(3):186–9.

456. Western College of Veterinary Medicine. *Sarcocystis* species. 2021 [cited 2024 Sep 29]. Available from: https://wcvm.usask.ca/learnaboutparasites/parasites/sarcocystis-species.php

457. Italiano C, Freedman D. Sarcocystosis. 2023 [cited 2024 Sep 29]. Available from: https://www.uptodate.com/contents/sarcocystosis#!

458. Spickler AR. The Center for Food Security and Public Health. 2020 [cited 2024 Sep 29]. Sarcocystosis. Available from: http://www.cfsph.iastate.edu/DiseaseInfo/factsheets.php.

459. Fayer R, Esposito DH, Dubey JP. Human infections with *Sarcocystis* species. Clin Microbiol Rev. 2015 Apr;28(2):295–311.

460. Perez-Marin CC. A Bird's-Eye View of Veterinary Medicine. BoD – Books on Demand; 2012. 630 p.

461. Herd HR, Sula MM, Starkey LA, Panciera RJ, Johnson EM, Snider TA, et al. *Sarcocystis fayeri* – induced granulomatous and eosinophilic myositis in 2 related horses. Vet Pathol. 2015 Nov;52(6):1191–4.

462. Hernandez-Valdivia E, Martínez-Robles J, Valdivia-Flores AG, Cruz-Vazquez C, Ortiz-Martinez R, Quezada-Tristan T. Prevalencia de parásitos digestivos de perros del centro de México. Rev MVZ Córdoba. 2024 May 3;27(3):e2686.

463. Hornok S, Mester A, Takács N, Baska F, Majoros G, Fok É, et al. *Sarcocystis*-infection of cattle in Hungary. Parasit Vectors. 2015 Feb 4;8(1):69.

464. Savini G, Dunsmore JD, Robertson ID, Seneviratna P. The epidemiology of *Sarcocystis* spp. in cattle of Western Australia. Epidemiol Infect. 1992 Feb;108(1):107–13.

465. Murata R, Suzuki J, Hyuga A, Shinkai T, Sadamasu K. Molecular identification and characterization of *Sarcocystis* spp. in horsemeat and beef marketed in Japan. Parasite. 25:27.

466. Oliveira CMC, Oliveira PB, Albuquerque GR, Gondim LFP. Serologic reactivity of canine sera to *Sarcocystis neurona* and *Sarcocystis cruzi* antigens. Vet Parasitol Reg Stud Rep. 2020 Jul;21:100439.

467. More GA. Merck Veterinary Manual. 2021 [cited 2024 Sep 29]. Sarcocystosis in animals. Available from: https://www.merckvetmanual.com/musculoskeletal-system/sarcocystosis/sarcocystosis-in-animals

468. Dryden M. Merck Veterinary Manual. 2023 [cited 2024 Sep 29]. Mange in dogs and cats. Available from: https://www.merckvetmanual.com/integumentary-system/mange/mange-in-dogs-and-cats

469. Wilson EC. The dynamics of sarcoptic mange in an urban coyote (*Canis latrans*) population. The Ohio State University; 2012.

470. Niedringhaus KD, Brown JD, Sweeley KM, Yabsley MJ. A review of sarcoptic mange in North American wildlife. Int J Parasitol Parasites Wildl. 2019 Jun 13;9:285–97.

471. Rodriguez-Vivas RI, Ortega-Pacheco A, Rosado-Aguilar JA, Bolio GME. Factors affecting the prevalence of mange-mite infestations in stray dogs of Yucatán, Mexico. Vet Parasitol. 2003 Jul 10;115(1):61–5.

472. Kočišová A, Letková V, Lukešová D. Ectoparasites of dogs. Slov Veterinársky Časopis. 2007 Jan 31;31(4):228–32.

473. Chee JH, Kwon JK, Cho HS, Cho KO, Lee YJ, Abd El-Aty AM, et al. A survey of ectoparasite infestations in stray dogs of Gwang-ju City, Republic of Korea. Korean J Parasitol. 2008 Mar;46(1):23–7.

474. Yoon SS, Byun JW, Wee SH, Song JY. OL-036 Prevalence of canine scabies in Korean stray dogs. Int J Infect Dis. 2010 Jul;14:S16.

475. Pawełczyk O, Pająk C, Solarz K. The risk of exposure to parasitic mites and insects occurring on pets in Southern Poland. Ann Parasitol. 2016 Sep 25;62:337–44.

476. Fraser TA, Charleston M, Martin A, Polkinghorne A, Carver S. The emergence of sarcoptic mange in Australian wildlife: an unresolved debate. Parasit Vectors. 2016 Jun 2;9(1):316.

477. Colella V, Nguyen VL, Tan DY, Lu N, Fang F, Zhijuan Y, et al. Zoonotic vectorborne pathogens and ectoparasites of dogs and cats in Eastern and Southeast Asia. Emerg Infect Dis. 2020 Jun;26(6):1221–33.

478. Western College of Veterinary Medicine. Sarcoptes species — sarcoptic mange or scabies. [cited 2024 Oct 1]. Available from: https://wcvm.usask.ca/learnaboutparasites/parasites/sarcoptes-species-sarcoptic-mange-or-scabies.php

479. Little SE, Cortinas R. 110 - Mites. In: Sykes JE, editor. Greene's Infectious Diseases of the Dog and Cat (Fifth Edition). Philadelphia: W.B. Saunders; 2021 [cited 2025 Feb 21]. p. 1378–98. Available from: https://www.sciencedirect.com/science/article/pii/B9780323509343001105

480. Merck Veterinary Manual. *Spirocerca lupi* in Small Animals. [cited 2024 Oct 1]. Available from: https://www.merckvetmanual.com/digestive-system/gastrointestinal-parasites-of-small-animals/spirocerca-lupi-in-small-animals

481. van der Merwe LL, Kirberger RM, Clift S, Williams M, Keller N, Naidoo V. *Spirocerca lupi* infection in the dog: A review. Vet J. 2008 Jun 1;176(3):294–309.

482. Dixon KG, McCue JF. Further observations on the epidemiology of *Spirocerca lupi* in the southeastern United States. J Parasitol. 1967 Oct;53(5):1074–5.

483. Morrison EE, Gier HT. Parasitic infection of *Filaroides osleri, Capillaria aerophila* and *Spirocera lupi* in coyotes from the Southwestern United States. J Wildl Dis. 1979 Oct;15(4):557–9.

484. Psáder R, Balogh M, Pápa K, Sterczer Á, Lukács Z, Harnos A. Occurrence of *Spirocerca lupi* infection in Hungarian dogs referred for gastroscopy. Parasitol Res. 2017 Aug;116(S1):99–108.

485. Szafrańska E, Wasielewski O, Bereszyński A. A faecal analysis of helminth infections in wild and captive wolves, Canis lupus L., in Poland. J Helminthol. 2010 Dec;84(4):415–9.

486. Kurnosova OP, Arisov MV, Odoyevskaya IM. Intestinal parasites of pets and other house-kept animals in Moscow. Helminthologia. 2019 Jun 1;56(2):108–17.

487. Rojas A, Dvir E, Farkas R, Sarma K, Borthakur S, Jabbar A, et al. Phylogenetic analysis of *Spirocerca lupi* and *Spirocerca vulpis* reveal high genetic diversity and intra-individual variation. Parasit Vectors. 2018 Dec 14;11(1):639.

488. Austin CM, Kok DJ, Crafford D, Schaper R. The efficacy of a topically applied imidacloprid 10% / moxidectin 2.5% formulation (Advocate®, Advantage® Multi, Bayer) against immature and adult *Spirocerca lupi* worms in experimentally infected dogs. Parasitol Res. 2013 Aug;112 Suppl 1:91–108.

489. Centers for Disease Control and Prevention. Strongyloides. 2024 [cited 2024 Oct 1]. Available from: https://www.cdc.gov/strongyloides/about/index.html

490. Puthiyakunnon S, Boddu S, Li Y, Zhou X, Wang C, Li J, et al. Strongyloidiasis—an insight into its global prevalence and management. PLoS Negl Trop Dis. 2014 Aug 14;8(8):e3018.

491. Gorgani-Firouzjaee T, Kalantari N, Chehrazi M, Ghaffari S, Shahdin S. Global prevalence of *Strongyloides stercoralis* in dogs: A systematic review and meta-analysis. J Helminthol. 2022 Feb 21;96:e11.

492. Western College of Veterinary Medicine. *Strongyloides stercoralis*. 2021 [cited 2024 Oct 1]. Available from: https://wcvm.usask.ca/learnaboutparasites/parasites/strongyloides-stercoralis.php

493. Merck Manual Professional Edition. Strongyloidiasis. [cited 2024 Oct 1]. Available from: https://www.merckmanuals.com/en-ca/professional/infectious-diseases/nematodes-roundworms/strongyloidiasis

494. Dashchenko S, Soroka N, Semenko O. Distribution of *Strongyloides stercoralis* among dogs of different housing groups in Kyiv and Kyiv region, clinical manifestations and diagnostic methods. Eureka Health Sci. 2020 Sep 29;(5):99–107.

495. Alvarado-Esquivel C, Romero-Salas D, Aguilar-Domínguez M, Cruz-Romero A, Ibarra-Priego N, Pérez-de-León AÁ. Epidemiological assessment of intestinal parasitic infections in dogs at animal shelter in Veracruz, Mexico. Asian Pac J Trop Biomed. 2015 Jan 1;5(1):34–9.

496. Štrkolcová G, Goldová M, Bocková E, Mojžišová J. The roundworm *Strongyloides stercoralis* in children, dogs, and soil inside and outside a segregated settlement in Eastern Slovakia: frequent but hardly detectable parasite. Parasitol Res. 2017 Mar;116(3):891–900.

497. Schnyder M, Reichler IM, Eichenberger RM, Hofer-Inteeworn N, Kümmerle-Fraune C, Grimm F. *Strongyloides stercoralis* in Swiss dogs - a retrospective study suggests an increasing occurrence of this potentially zoonotic parasite as a consequence of dog imports. Schweiz Arch Tierheilkd. 2022 Jan;164(1):89–104.

498. Ottino L, Buonfrate D, Paradies P, Bisoffi Z, Antonelli A, Rossolini GM, et al. Autochthonous human and canine *Strongyloides stercoralis* infection in europe: Report of a human case in an Italian teen and systematic review of the literature. Pathogens. 2020 Jun 3;9(6):439.

499. Thamsborg SM, Ketzis J, Horii Y, Matthews JB. *Strongyloides* spp. infections of veterinary importance. Parasitology. 2017 Mar;144(3):274–84.

500. Companion Animal Parasite Council. *Taenia* spp. [cited 2024 Oct 2]. Available from: https://capcvet.org/guidelines/taenia/

501. Adolph C, Barnett S, Beall M, Drake J, Elsemore D, Thomas J, et al. Diagnostic strategies to reveal covert infections with intestinal helminths in dogs. Vet Parasitol. 2017 Nov;247:108–12.

502. Kornyushin VV, Malega OM, Varodi EI, Korol EM, Kuzmina TA, Sokolova O, et al. Review of the helminths of carnivora (Mammalia) in Ukraine: composition and structure of helminth fauna. Zoodiversity. 2022;56(6):495–514.

503. Trasviña-Muñoz E, López-Valencia G, Centeno PÁ, Cueto-González SA, Monge-Navarro FJ, Tinoco-Gracia L, et al. Prevalence and distribution of intestinal parasites in stray dogs in the northwest area of Mexico. Austral J Vet Sci. 2017 May;49(2):105–11.

504. Nikolić A, Dimitrijević S, Katić-Radivojević S, Klun I, Bobić B, Djurković-Djaković O. High prevalence of intestinal zoonotic parasites in dogs from Belgrade, Serbia — Short communication. Acta Vet Hung. 2008 Sep 1;56(3):335–40.

505. Tylkowska A, Pilarczyk B, Gregorczyk A, Templin E. Gastrointestinal helminths of dogs in Western Pomerania, Poland. Wiad Parazytol. 2010;56(3):269–76.

506. Moskvina TV, Zheleznova LV. A survey on endoparasites and ectoparasites in domestic dogs and cats in Vladivostok, Russia 2014. Vet Parasitol Reg Stud Rep. 2015 Dec 1;1–2:31–4.

507. Western College of Veterinary Medicine. *Taenia* species. [cited 2024 Oct 2]. Available from: https://wcvm.usask.ca/learnaboutparasites/parasites/taenia-species.php

508. Brooks W. [cited 2024 Oct 2] 2017 Aug 8; Available from: https://www.vin.com/doc/?id=8156779

509. Winders WT, Menkin-Smith L. *Toxocara canis*. In: StatPearls. Treasure Island (FL): StatPearls Publishing; 2024 [cited 2024 Oct 2]. Available from: http://www.ncbi.nlm.nih.gov/books/NBK538524/

510. Centers for Disease Control and Prevention. Toxocariasis. 2019 [cited 2024 Oct 2]. Available from: https://www.cdc.gov/dpdx/toxocariasis/index.html

511. Rostami A, Riahi SM, Hofmann A, Ma G, Wang T, Behniafar H, et al. Global prevalence of *Toxocara* infection in dogs. Adv Parasitol. 2020;109:561–83.

512. Lucio-Forster A, Mizhquiri Barbecho JS, Mohammed HO, Kornreich BG, Bowman DD. Comparison of the prevalence of *Toxocara* egg shedding by pet cats and dogs in the U.S.A., 2011–2014. Vet Parasitol Reg Stud Rep. 2016 Sep 1;5:1–13.

513. Paliy A, Sumakova N, Petrov R, Shkromada O, Ulko L, Palii A. Contamination of urbanized territories with eggs of helmiths of animals. Biosyst Divers. 2019 May 15;27(2):118–24.

514. Ondriska F, Mačuhová K, Melicherová J, Reiterová K, Valentová D, Beladičová V, et al. Toxocariasis in urban environment of western Slovakia. Helminthologia. 2013 Dec 1;50(4):261–8.

515. Kim YH, Huh S. Prevalence of *Toxocara canis, Toxascaris leonina* and *Dirofilaria immitis* in dogs in Chuncheon, Korea (2004). Korean J Parasitol. 2005 Jun;43(2):65–7.

516. Mizgajska-Wiktor H, Jarosz W, Fogt-Wyrwas R, Drzewiecka A. Distribution and dynamics of soil contamination with *Toxocara canis* and *Toxocara cati* eggs in Poland and prevention measures proposed after 20 years of study. Vet Parasitol. 2017 Jan 30;234:1–9.

517. Lukashev AN, Ruzina MN, Akhmadishina LV. *Toxocara* prevalence in dogs, cats and the environment in Russia. In: Bowman DD, editor. Advances in Parasitology. Academic Press; 2020 [cited 2024 Oct 2]. p. 801–17. (Toxocara and Toxocariasis; vol. 109). Available from: https://www.sciencedirect.com/science/article/pii/S0065308X20300191

518. Spickler AR. The Center for Food Security and Public Health. Toxocariasis. 2016 [cited 2024 Oct 2]. Available from: https://www.cfsph.iastate.edu/Factsheets/pdfs/toxocariasis.pdf

519. Schwartz R, Bidaisee S, Fields PJ, Macpherson MLA, Macpherson CNL. The epidemiology and control of *Toxocara canis* in puppies. Parasite Epidemiol Control. 2022 Feb [cited 2024 Oct 9];16. Available from: https://www.ncbi.nlm.nih.gov/pmc/articles/PMC8669357/

520. Companion Animal Parasite Council. *Trichuris vulpis*. 2022 [cited 2024 Oct 9]. Available from: https://capcvet.org/guidelines/trichuris-vulpis/

521. Saichenko IV, Antipov AA, Bakhur TI, Bezditko LV, Shmayun SS. Co-infection of *Trichuris vulpis* and *Toxocara canis* in different aged dogs: Influence on the haematological indices. Biosyst Divers. 2021 May 20;29(2):129–34.

522. Medina-Pinto RA, Rodríguez-Vivas RI, Bolio-González ME. Zoonotic intestinal nematodes in dogs from public parks in Yucatán, México. Biomed Rev Inst Nac Salud. 2018 Mar 15;38(1):105–10.

523. Aguillon-Gutierrez D. Prevalencia de parásitos en heces fecales de perros de Gómez Palacio, Durango, México. Abanico Vet [Internet]. 2021 Jan 2 [cited 2024 Oct 9];11. Available from: https://abanicoacademico.mx/revistasabanico/index.php/abanico-veterinario/article/view/341

524. Bartosik J, Dziwirek K, Łojek J, Kaczyk J, Górski P. Prevalence of intestinal parasite infection in dogs from selected rural areas of central and southern Poland. Rocz Nauk Pol Tow Zootech. 2017 Mar 31;13(1):61–9.

525. Jenkins D, Lievaart J, Boufana B, Lett W, Bradshaw H, Armua-Fernandez M. *Echinococcus granulosus* and other intestinal helminths: current status of prevalence and management in rural dogs of eastern Australia. Aust Vet J. 2014 Aug;92(8):292–8.

526. Western College of Veterinary Medicine. *Trypanosoma cruzi*. 2021 [cited 2024 Oct 9]. Available from: https://wcvm.usask.ca/learnaboutparasites/parasites/trypanosoma-cruzi.php

527. Busselman RE, Meyers AC, Zecca IB, Auckland LD, Castro AH, Dowd RE, et al. High incidence of *Trypanosoma cruzi* infections in dogs directly detected through longitudinal tracking at 10 multi-dog kennels, Texas, USA. PLoS Negl Trop Dis. 2021 Nov 10;15(11):e0009935.

528. Meyers AC, Purnell JC, Ellis MM, Auckland LD, Meinders M, Hamer SA. Nationwide exposure of U.S. working dogs to the Chagas disease parasite, *Trypanosoma cruzi*. Am J Trop Med Hyg. 2020 May 6;102(5):1078–85.

529. Weir M, Barnette C. Chagas disease in dogs. [cited 2024 Oct 9]. Available from: https://vcacanada.com/know-your-pet/chagas-disease-in-dogs

530. Arce-Fonseca M, Carrillo-Sánchez SC, Molina-Barrios RM, Martínez-Cruz M, Cedillo-Cobián JR, Henao-Díaz YA, et al. Seropositivity for *Trypanosoma cruzi* in domestic dogs from Sonora, Mexico. Infect Dis Poverty. 2017 Dec;6(1):120.

531. Barbabosa-Pliego A, Gil PC, Hernández DO, Aparicio-Burgos JE, De Oca-Jiménez RM, Martínez-Castañeda JS, et al. Prevalence of *Trypanosoma cruzi* in dogs (*Canis familiaris*) and triatomines during 2008 in a sanitary region of the state of Mexico, Mexico. Vector-Borne Zoonotic Dis. 2011 Feb;11(2):151–6.

532. Martínez I, Martínez-Ibarra A, Arce-Fonseca M, Rodríguez-Morales O, Pérez-Morales D, Reyes López PA, et al. Seroprevalence and major antigens recognized by sera from *Trypanosoma cruzi*-infected dogs from Jalisco, México. Rev Argent Microbiol. 2014 Apr;46(2):85–90.

533. Choi MH, Yu JR, Hong ST. Who Neglects Neglected Tropical Diseases? - Korean Perspective. J Korean Med Sci. 2015;30(Suppl 2):S122.

534. Thompson RCA. Exotic parasite threats to australia's biosecurity—trade, health, and conservation. Trop Med Infect Dis. 2018 Jul 18;3(3):76.

535. Jackson Y, Pinto A, Pett S. Chagas disease in Australia and New Zealand: risks and needs for public health interventions. Trop Med Int Health. 2014 Feb;19(2):212–8.

536. Centers for Disease Control and Prevention. American trypanosomiasis. 2021 [cited 2024 Oct 9]. Available from: https://www.cdc.gov/dpdx/trypanosomiasisamerican/index.html

537. Spickler AR. The Center for Food Security and Public Health. African animal trypanosomiasis 2018 [cited 2024 Oct 9]. Available from: https://www.cfsph.iastate.edu/Factsheets/pdfs/trypanosomiasis_african.pdf

538. Hamer SA, Saunders AB, Snowden KF, Sykes JE. 100 - Trypanosomiasis. In: Sykes JE, editor. Greene's Infectious Diseases of the Dog and Cat (Fifth Edition). Philadelphia: W.B. Saunders; 2021 [cited 2025 Feb 21]. p. 1248–62. Available from: https://www.sciencedirect.com/science/article/pii/B9780323509343001002

539. Magri A, Galuppi R, Fioravanti M. Autochthonous *Trypanosoma* spp. in European mammals: A brief journey amongst the neglected Trypanosomes. Pathogens. 2021 Mar 13;10(3):334.

540. Calvet F, Medkour H, Mediannikov O, Girardet C, Jacob A, Boni M, et al. An african canine trypanosomosis case import: Is there a possibility of creating a secondary focus of *Trypanosoma congolense* infection in France? Pathogens. 2020 Aug 27;9(9):709.

541. Museux K, Boulouha L, Majani S, Journaux H. African *Trypanosoma* infection in a dog in France. Vet Rec. 2011;168(22):590–590.

542. Deschamps JY, Desquesnes M, Dorso L, Ravel S, Bossard G, Charbonneau M, et al. Refractory hypoglycaemia in a dog infected with *Trypanosoma congolense*. Parasite. 2016;23:1.

543. Gow AG, Simpson JW, Picozzi K. First report of canine African trypanosomosis in the UK. J Small Anim Pract. 2007;48(11):658–61.

544. Harrus S, Harmelin A, Presenty B, Bark H. *Trypanosoma congolense* infection in two dogs. J Small Anim Pract. 1995 Feb;36(2):83–6.

545. Keck N, Herder S, Kaba D, Solano P, Gomez J, Cuny G, et al. Epidemiological study of canine Trypanosomosis in an urban area of Ivory coast. Parasite. 2009 Dec;16(4):305–8.

546. Lisulo M, Sugimoto C, Kajino K, Hayashida K, Mudenda M, Moonga L, et al. Determination of the prevalence of African trypanosome species in indigenous dogs of Mambwe district, eastern Zambia, by loop-mediated isothermal amplification. Parasit Vectors. 2014 Jan 10;7(1):19.

547. World Organisation for Animal Health. Trypanosomosis (tsetse-transmitted). World Organisation for Animal Health; 2021.

548. Kay C, Peacock L, Gibson W. *Trypanosoma congolense*: In Vitro Culture and Transfection. Curr Protoc Microbiol. 2019 Jun;53(1):e77.

549. Peacock L, Cook S, Ferris V, Bailey M, Gibson W. The life cycle of *Trypanosoma (Nannomonas) congolense* in the tsetse fly. Parasit Vectors. 2012 Dec;5(1):109.

550. Decaro N, Martella V, Buonavoglia C. Canine Adenoviruses and Herpesvirus. Vet Clin North Am Small Anim Pract. 2008 Jul;38(4):799–814.

551. Timurkan MO, Aydin H, Alkan F. Detection and molecular characterization of canine adenovirus type 2 (CAV-2) in dogs with respiratory tract symptoms in shelters in Turkey. Vet Arh. 2018 Jul 1;88(4):467–79.

552. Decaro N. 23 - Infectious Canine Hepatitis and Feline Adenovirus Infection. In: Sykes JE, editor. Greene's Infectious Diseases of the Dog and Cat (Fifth Edition). Philadelphia: W.B. Saunders; 2021 [cited 2025 Feb 21]. p. 289–300. Available from: https://www.sciencedirect.com/science/article/pii/B9780323509343000239

553. Caudell D, Confer AW, Fulton RW, Berry A, Saliki JT, Fent GM, et al. Diagnosis of infectious canine hepatitis virus (CAV-1) infection in puppies with encephalopathy. J Vet Diagn Invest. 2005 Jan;17(1):58–61.

554. Day MJ, Crawford C, Marcondes M, Squires RA. Recommendations on vaccination for Latin American small animal practitioners: a report of the WSAVA Vaccination Guidelines Group. J Small Anim Pract. 2020 Jun [cited 2025 Feb 20];61(6). Available from: https://onlinelibrary.wiley.com/doi/10.1111/jsap.13125

555. Decaro N, Campolo M, Elia G, Buonavoglia D, Colaianni ML, Lorusso A, et al. Infectious canine hepatitis: An "old" disease reemerging in Italy. Res Vet Sci. 2007 Oct;83(2):269–73.

556. Robinson AJ, Crerar SK, Waight Sharma N, Müller WJ, Bradley MP. Prevalence of serum antibodies to canine adenovirus and canine herpesvirus in the European red fox (*Vulpes vulpes*) in Australia. Aust Vet J. 2005 Jun;83(6):356–61.

557. Wong M, Woolford L, Hasan NH, Hemmatzadeh F. A novel recombinant canine adenovirus type 1 detected from acute lethal cases of infectious canine hepatitis. Viral Immunol. 2017 May;30(4):258–63.

558. Mira F, Puleio R, Schirò G, Condorelli L, Di Bella S, Chiaramonte G, et al. Study on the canine adenovirus type 1 (CAdV-1) infection in domestic dogs in Southern Italy. Pathogens. 2022 Oct 28;11(11):1254.

559. Chethan GE, Singh M, Chander VA, Singh D, Rajesh JB, et al. Occurrence of canine parvovirus-2 and canine adenovirus-1 infections in dogs: A hospital based study. Indian J Anim Res. 2020 Mar 24 [cited 2025 Feb 20];(Of). Available from: https://arccjournals.com/journal/indian-journal-of-animal-research/B-3936

560. Gleich S, Kamenica K, Janik D, Benetka V, Möstl K, Hermanns W, et al. Infectious canine hepatitis in central Europe - Canine Adenovirus-(CAV)-1 infection in a puppy in Germany. Vet Med Austria. 2009, 96:227 – 231.

561. Gámiz C, Martella V, Ulloa R, Fajardo R, Quijano-Hernandéz I, Martínez S. Identification of a new genotype of canine distemper virus circulating in America. Vet Res Commun. 2011 Aug;35(6):381–90.

562. Panzera Y, Calderón MG, Sarute N, Guasco S, Cardeillac A, Bonilla B, et al. Evidence of two co-circulating genetic lineages of canine distemper virus in South America. Virus Res. 2012 Jan;163(1):401–4.

563. Kapil S, Allison RW, Johnston L, Murray BL, Holland S, Meinkoth J, et al. Canine distemper virus strains circulating among North American dogs. Clin Vaccine Immunol CVI. 2008 Apr;15(4):707–12.

564. MacLachlan NJ, Dubovi EJ, editors. Paramyxoviridae and Pneumoviridae. In: Fenner's Veterinary Virology (Fifth Edition). Boston: Academic Press; 2017 [cited 2025 Feb 2]. p. 327–56. Available from: https://www.sciencedirect.com/science/article/pii/B9780128009468000179

565. Maboni G, Seguel M, Lorton A, Berghaus R, Sanchez S. Canine infectious respiratory disease: New insights into the etiology and epidemiology of associated pathogens. PloS One. 2019;14(4):e0215817.

566. Almuna R, López-Pérez AM, Sarmiento RE, Suzán G. Drivers of canine distemper virus exposure in dogs at a wildlife interface in Janos, Mexico. Vet Rec Open. 2021 Dec;8(1):e7.

567. Demeter Z, Lakatos B, Palade EA, Kozma T, Forgách P, Rusvai M. Genetic diversity of Hungarian canine distemper virus strains. Vet Microbiol. 2007 Jun 21;122(3–4):258–69.

568. Cha SY, Kim EJ, Kang M, Jang SH, Lee HB, Jang HK. Epidemiology of canine distemper virus in wild raccoon dogs (*Nyctereutes procyonoides*) from South Korea. Comp Immunol Microbiol Infect Dis. 2012 Sep;35(5):497–504.

569. Jóžwik A, Frymus T. Natural distemper in vaccinated and unvaccinated dogs in Warsaw. J Vet Med Ser B. 2002 Nov;49(9):413–4.

570. Wyllie S, Kelman M, Ward M. Epidemiology and clinical presentation of canine distemper disease in dogs and ferrets in Australia, 2006–2014. Aust Vet J. 2016 Jul;94(7):215–22.

571. Ruffalo G, Byers M, Ward MP. A canine distemper virus seroprevalence study of dogs on three islands in the Torres Strait region, Australia. Aust Vet J. 2020 Aug;98(8):371–4.

572. Ke GM, Ho CH, Chiang MJ, Sanno-Duanda B, Chung CS, Lin MY, et al. Phylodynamic analysis of the canine distemper virus hemagglutinin gene. BMC Vet Res. 2015 Dec;11(1):164.

573. Hsu Z, Lai Y, Jiang J, Chen R, Chen M, Yu J. Canine distemper and mixed infections in stray dogs. Anim Health Rep. 2011;11–8.

574. Luo H, Li K, Zhang H. Epidemiology of canine distemper and canine parvovirus in pet dogs in Wenzhou, China. Indian J Anim Res. 2016 Apr 7;51(1):159–61.

575. Gencay A, Oncel T, Karaoglu T, Sancak A, Demir A, Ozkul A. Antibody prevalence to canine distemper virus (CDV) in stray dogs in Turkey. Rev Med Veterinaire. 2004;155(8):432–4.

576. Mousafarkhani F, Sarchahi AA, Mohebalian H, Khoshnegah J, Arbabi M. Prevalence of canine distemper in dogs referred to veterinary hospital of Ferdowsi University of Mashhad, Mashhad, Iran. Vet Res Forum. 2023;14(3):153–60.

577. Headley SA, Amude AM, Alfieri AF, Bracarense APFRL, Alfieri AA. Epidemiological features and the neuropathological manifestations of canine distemper virus-induced infections in Brazil: a review. Semina Ciênc Agrár. 2012 Oct 30;33(5):1945–78.

578. Kelly PJ, Musuka G, Eoghin GN, Tebje-Kelly JBU, Carter S. Serosurvey for canine distemper virus exposure in dogs in communal lands in Zimbabwe. J S Afr Vet Assoc. 2005 Jun 13;76(2):104–6.

579. Cornell Wildlife Health Center. Canine distemper. 2016 [cited 2025 Feb 2]. Available from: https://cwhl.vet.cornell.edu/disease/canine-distemper

580. Canadian Veterinary Medical Association. Vaccination and your Dog. 2016 [cited 2025 Feb 15]. Available from: https://www.canadianveterinarians.net/related-resources/vaccination-and-your-dog/

581. Davidson AP, Sykes JE, Casal ML. 24 - Canine Herpesvirus Infection. In: Sykes JE, editor. Greene's Infectious Diseases of the Dog and Cat (Fifth Edition). Philadelphia: W.B. Saunders; 2021 [cited 2025 Feb 21]. p. 301–9. Available from: https://www.sciencedirect.com/science/article/pii/B9780323509343000240

582. Lara EGV, Solis JIÁ, Verde CC, Crespo JAM, Marín LC, García JCDR, et al. Canine herpesvirus seroprevalence and associated factors in dogs of Mexico. Open J Vet Med. 2016 Oct 27;6(10):149–62.

583. Babaei H, Akhtardanesh B, Ghanbarpour R, Namjoo A. Serological evidence of canine herpesvirus-1 in dogs of Kerman city, south-east of Iran. Transbound Emerg Dis. 2010 Oct;57(5):348–51.

584. Krogenæs A, Rootwelt V, Larsen S, Renström L, Farstad W, Lund A. A serological study of canine herpesvirus-1 infection in a population of breeding bitches in Norway. Acta Vet Scand. 2014 Apr 2;56(1):19.

585. Pratelli A, Colao V, Losurdo M. Serological and virological detection of canine herpesvirus-1 in adult dogs with and without reproductive disorders. Vet J. 2014 May 1;200(2):257–60.

586. Acar A, Gur S, Dogan I, Akca Y. A serologic investigation of canine herpesvirus type 1 infection in Kangal Dogs. J Anim Vet Adv. 2022;8(7):1377–80.

587. Luff JA, Munday JS. 40 - Papillomavirus Infections. In: Sykes JE, editor. Greene's Infectious Diseases of the Dog and Cat (Fifth Edition). Philadelphia: W.B. Saunders; 2021 [cited 2025 Feb 21]. p. 477–88. Available from: https://www.sciencedirect.com/science/article/pii/B9780323509343000409

588. Gould AP, Coyner KS, Trimmer AM, Tater K, Rishniw M. Canine pedal papilloma identification and management: a retrospective series of 44 cases. Vet Dermatol. 2021 Oct;32(5):509.

589. Yhee JY, Kwon BJ, Kim JH, Yu CH, Im KS, Lee SS, et al. Characterization of canine oral papillomavirus by histopathological and genetic analysis in Korea. J Vet Sci. 2010;11(1):21.

590. Ciaputa R, Gach J, Baranowski K, Dzimira S, Janus I, Kandefer-Gola M, et al. Prevalence of tumours and tumour-like lesions in domestic and exotic animals from Lower Silesia and its surrounds in Poland in 2014–2017. J Vet Res. 2022 Aug 30;66(3):427–34.

591. Kudacheva NA. Papillomavirus-associated neoplasms of dogs and cats. Vet Zootekhniya Biotekhnologiya. 2022;11(108):43–51.

592. Bhatta TR, Chamings A, Vibin J, Alexandersen S. Detection and characterization of canine astrovirus, canine parvovirus and canine papillomavirus in puppies using next generation sequencing. Sci Rep. 2019 Mar 14;9(1):4602.

593. Chang CY, Chen WT, Haga T, Yamashita N, Lee CF, Tsuzuki M, et al. The detection and association of canine papillomavirus with benign and malignant skin lesions in dogs. Viruses. 2020 Feb 3;12(2):170.

594. Sancak A, Favrot C, Geisseler MD, Müller M, Lange CE. Antibody titres against canine papillomavirus 1 peak around clinical regression in naturally occurring oral papillomatosis. Vet Dermatol. 2015;26(1):57-e20.

595. Bhaiyat MI, Chikweto A, Tiwari KP, DeAllie C, Pawaiya RS, Inga A, et al. A retrospective study of canine tumors in Grenada, West Indies. Adv Anim Vet Sci. 2013;1(5):134–9.

596. Bianchi MV, Casagrande RA, Watanabe TTN, Wouters ATB, Wouters F, Boos GS, et al. Canine papillomatosis: a retrospective study of 24 cases (2001-2011) and immunohistochemical characterization. Pesqui Veterinária Bras. 2012 Jul;32(7):653–7.

597. Parrish CR, Sykes JE. 29 - Canine Parvovirus Infections and Other Viral Enteritides. In: Sykes JE, editor. Greene's Infectious Diseases of the Dog and Cat (Fifth Edition). Philadelphia: W.B.

Saunders; 2021 [cited 2025 Feb 21]. p. 341–51. Available from: https://www.sciencedirect.com/science/article/pii/B978032350934300029X

598. Gagnon CA, Allard V, Cloutier G. Canine parvovirus type 2b is the most prevalent genomic variant strain found in parvovirus antigen positive diarrheic dog feces samples across Canada. Can Vet J. 2016 Jan;57(1):29–31.

599. DiGangi BA, Craver C, Dolan ED. Incidence and predictors of canine parvovirus diagnoses in puppies relocated for adoption. Animals. 2021 Apr;11(4):1064.

600. Tion MT, Fotina H, Saganuwan SA. Part 1. Veterinary medicine a retrospective study of canine parvovirus in private veterinary clinic "health", Sumy region, Ukraine (2015-2018). J Vet Med Biotechnol Biosaf. 2018;4(3):5–9.

601. Pedroza-Roldán C, Hernández-Almaraz MA, Elizondo-Quiroga D, Gutierrez-Ortega A, Acosta-Monroy CM, Charles-Niño C, et al. Exclusive circulation of canine parvovirus type 2c in the Guadalajara metropolitan area in western Mexico: a five-year study. Arch Virol. 2022 Nov 1;167(11):2109–21.

602. Decaro N, Buonavoglia C. Canine parvovirus—A review of epidemiological and diagnostic aspects, with emphasis on type 2c. Vet Microbiol. 2011 Sep 12;155(1):1.

603. Demeter Z. Polymerase chain reaction-based investigations of canine distemper and parvovirus strains from Hungary. 2009 [cited 2025 Feb 6]; Available from: http://huveta.hu/handle/10832/153

604. Yang DK, Yoon SS, Byun JW, Lee KW, Oh YI, Song JY. Serological survey for canine parvovirus type 2a (CPV-2a) in the stray dogs in South Korea. J Bacteriol Virol. 2010;40(2):77.

605. Kowalczyk M, Majer-Dziedzic B, Kostro K, Szabelak A, Ziętek J, Gryzinska M, et al. Diagnostics and genotyping of canine parvovirus type 2 (CPV-2) from disease cases in south-eastern Poland. Acta Vet (Beogr). 2019 Mar 1;69(1):32–46.

606. Zourkas E, Ward MP, Kelman M. Canine parvovirus in Australia: A comparative study of reported rural and urban cases. Vet Microbiol. 2015 Dec 31;181(3):198–203.

607. Chiang SY, Wu HY, Chiou MT, Chang MC, Lin CN. Identification of a novel canine parvovirus type 2c in Taiwan. Virol J. 2016 Sep 23;13(1):160.

608. Behera M, Panda SK, Sahoo PK, Acharya AP, Patra RC, Das S, et al. Epidemiological study of canine parvovirus infection in and around Bhubaneswar, Odisha, India. Vet World. 2015 Jan;8(1):33–7.

609. Sayed-Ahmed MZ, Elbaz E, Younis E, Khodier M. Canine parvovirus infection in dogs: Prevalence and associated risk factors in Egypt. Worlds Vet J. 2020 Dec 25;571–7.

610. Temizkan MC, Sevinc Temizkan S. Canine parvovirus in Turkey: First whole-genome sequences, strain distribution, and prevalence. Viruses. 2023 Apr 13;15(4):957.

611. Qi S, Zhao J, Guo D, Sun D. A mini-review on the epidemiology of canine parvovirus in China. Front Vet Sci. 2020 Feb 20;7:5.

612. Merck Veterinary Manual. Canine Parvovirus [cited 2025 Feb 6]. Available from: https://www.merckvetmanual.com/digestive-system/diseases-of-the-stomach-and-intestines-in-small-animals/canine-parvovirus

613. Crawford PC, Dubovi EJ, Castleman WL, Stephenson I, Gibbs EPJ, Chen L, et al. Transmission of equine influenza virus to dogs. Science. 2005 Oct 21;310(5747):482–5.

614. Centers for Disease Control and Prevention. Influenza in animals. 2024 [cited 2025 Feb 15]. Available from: https://www.cdc.gov/flu-in-animals/about/canine-flu.html

615. Voorhees IEH, Dalziel BD, Glaser A, Dubovi EJ, Murcia PR, Newbury S, et al. Multiple incursions and recurrent epidemic fade-out of H3N2 canine influenza a virus in the United States. J Virol. 2018 Aug 15;92(16):e00323-18.

616. Holt DE, Mover MR, Brown DC. Serologic prevalence of antibodies against canine influenza virus (H3N8) in dogs in a metropolitan animal shelter. J Am Vet Med Assoc. 2010 Jul 1;237(1):71–3.

617. Kovalenko G, Galat M, Ishchenko L, Halka I. Serological evidence for influenza a viruses among domestic dogs and cats in Kyiv, Ukraine. Vector-Borne Zoonotic Dis. 2021 Jul;21(7):483–9.

618. Ramírez-Martínez LA, Contreras-Luna M, De la Luz J, Manjarrez ME, Rosete DP, Rivera-Benitez JF, et al. Evidence of transmission and risk factors for influenza A virus in household dogs and their owners. Influenza Other Respir Viruses. 2013;7(6):1292–6.

619. Lee C, Song D, Kang B, Kang D, Yoo J, Jung K, et al. A serological survey of avian origin canine H3N2 influenza virus in dogs in Korea. Vet Microbiol. 2009 Jun;137(3–4):359–62.

620. Kwasnik M, Smreczak M, Rola J, Urbaniak K, Rozek W. Serologic investigation of influenza A virus infection in dogs in Poland. J Vet Diagn Investig Off Publ Am Assoc Vet Lab Diagn Inc. 2020 May;32(3):420–2.

621. Kirkland PD, Finlaison DS, Crispe E, Hurt AC. Influenza virus transmission from horses to dogs, Australia. Emerg Infect Dis. 2010 Apr;16(4):699–702.

622. Lin HT, Wang CH, Chueh LL, Su BL, Wang LC. Influenza A(H6N1) virus in dogs, Taiwan. Emerg Infect Dis. 2015 Dec;21(12):2154–7.

623. Su S, Chen Y, Zhao FR, Chen JD, Xie JX, Chen ZM, et al. Avian-origin H3N2 canine influenza virus circulating in farmed dogs in Guangdong, China. Infect Genet Evol J Mol Epidemiol Evol Genet Infect Dis. 2013 Oct;19:251–6.

624. Spickler AR. The Center for Food Security and Public Health. Canine Influenza. 2022 [cited 2023 Feb 21]. Available from: http://www.cfsph.iastate.edu/DiseaseInfo/ factsheets.php.

625. Vahlenkamp Thomas W, Sykes JE. 25 - Influenza Virus Infections. In: Sykes JE, editor. Greene's Infectious Diseases of the Dog and Cat (Fifth Edition). Philadelphia: W.B. Saunders; 2021 [cited 2025 Feb 21]. p. 310–20. Available from: https://www.sciencedirect.com/science/article/pii/B9780323509343000252

626. American Veterinary Medical Association. Canine influenza: Veterinary resources. [cited 2025 Feb 16]. Available from: https://www.avma.org/resources-tools/animal-health-and-welfare/animal-health/canine-influenza-veterinary-resources
