# Supplementary material for: Qualitative Risk Assessment of Infectious Agents Associated with Canine Importation into Canada, 2023–2024
Source: Emerg Infect Dis. 2026 Aug;32(8):1231–40. doi: 10.3201/eid3208.251602 (PMC13426852; doi:10.3201/eid3208.251602)
Supplement: Appendix 3 — Additional information used for magnitude of impact estimates for a qualitative risk assessment of infectious agents associated with canine importation into Canada, 2023–2024 [file 25-1602-Techapp-s3.pdf]

Article DOI: https://doi.org/10.3201/eid3208.251602

EID cannot ensure accessibility for supplementary materials supplied by authors. Readers who have difficulty accessing supplementary content should contact the authors for assistance.

Qualitative Risk Assessment of Infectious Agents Associated with Canine Importation into Canada, 2023–2024

Appendix 3

Appendix 3 Table. Data compiled from a comprehensive literature search and shared with experts before questionnaire completion to inform the estimates for magnitude of impact of exposure for individual canines, individual humans, the canine population, and the human population for a qualitative risk assessment of 53 hazards potentially associated with canine importation into Canada.

HAZARD	CATEGORY	SPREAD SCENARIO CANINE	IMPACT ANIMAL	SPREAD SCENARIO HUMAN	IMPACT HUMAN	OTHER INFORMATION
Bartonella vinsonii* subsp. *berkhoffii **Bartonellosis** *More than 10 *Bartonella* spp. Have been documented to infect canines as incidental hosts (*1*,*2*). UNCERTAINTY = Low	Bacteria	Primary mode of transmission is vector-borne (*3*). *Ctenocephalides felis* (cat flea) is confirmed, *R. sanguineus* (brown dog tick) is suspected (*3*).	Spectrum of clinical disease, ranging from subclinical to severe (*3*). Most common is vegetative endocarditis of the aortic valve leading to congestive heart failure (*3*,*4*). Other signs include myocarditis, polyarthritis, anemia, thrombocytopenia, uveitis (*3*).	Documented transmission has occurred via bites and scratches from infected canine (*3*). Humans are incidental hosts with no further transmission to other humans (*5*).	Immunocompromised are at highest risk (*3*). Leads to fever, lymphadenopathy, and endocarditis (*6*,*7*).	
**Canine infectious respiratory disease complex agents** Canine infectious respiratory disease complex (e.g., kennel cough) UNCERTAINTY = Low	Bacteria + viruses	Direct transmission from an infected dog via respiratory secretions (*8*). Indirect transmission can occur via contact with contaminated environments but depends on pathogen survival (*8*). Highly contagious, especially in high density housing of dogs (*9*).	Vast majority of cases are mild, self-limiting infections associated with mild fever, a dry hacking cough, nasal discharge and conjunctivitis (*8*,*9*). Most severe cases occur in young or senior immunosuppressed dogs, or when there are co-infections (*8*).	Not zoonotic	N/A	Vaccines are not fully effective (*8*).
Brucella canis *Canine brucellosis* UNCERTAINTY = High	Bacteria	Transmission occurs via breeding or direct contact with bodily fluids. Vertical transmission also occurs (*10*,*11*). Intact animals are the greatest risk as the highest concentrations of *B. canis* are found in reproductive tissues and fluids (*12*). Lower concentrations have been found in saliva, feces, nasal and ocular secretions, blood, and	For intact females, reproductive failure is the most common sign (e.g., abortions, stillbirths) (*10*). Intact males present with epididymitis, scrotal edema and orchitis, and can have reduced fertility (*13*). Some dogs may experience systemic signs including lymphadenopathy, lethargy, exercise intolerance, anorexia, and weight loss (*10*). Sterilized dogs can present with ocular disease and discospondylitis leading to spinal pain, paresis, and paralysis (*10*).	Humans are susceptible, although few cases have been reported so the true burden is unknown (*10*,*13*). Given the close contact with dogs, it is considered a significant public health concern (*14*). Transmission generally occurs through contact with reproductive tissues and fluids. Cases have been reported from owners with	Clinical signs of *B. canis* infection in humans resemble mild, influenza-like illness, and symptoms are often non-specific, including intermittent fever, chills, night sweats, loss of appetite, weight loss, fatigue, headaches, muscle aches or joint pain (*13*,*14*). Less common symptoms include endocarditis, liver granulomas, enlarged liver and spleen,	

HAZARD	CATEGORY	SPREAD SCENARIO CANINE	IMPACT ANIMAL	SPREAD SCENARIO HUMAN	IMPACT HUMAN	OTHER INFORMATION
		urine, and thus transmission can occur from sterilized dogs (*11*).	No treatment exists to effectively clear infection. Sterilization reduces the potential for transmission but does not eliminate it. Dogs infected with *B. canis* are frequently euthanized to prevent spread, regardless of clinical signs (*13*).	dogs who have *B. canis*-associated discospondylitis (*13*).	osteomyelitis, and pulmonary disease (*13*). Highest risk groups include those who are young, immunocompromised, and pregnant (*13*). Prolonged antibiotic treatment is required (*13*,*14*).	
Burkholderia pseudomallei (Melioidosis) UNCERTAINTY = High	Bacteria	Dogs most commonly become infected from contaminated environment, although dog to dog transmission has been documented in rare cases (*15*,*16*). Bacteria are shed from infected wounds and other secretions, depending on the site of infection (e.g., urine, feces, nasal secretion, if those sites are infected) (*16*).	In healthy, immunocompetent individuals, disease (melioidosis) is rare (*15*). However, in dogs with compromised immunity or comorbidities, disease can be severe and life threatening. Infection leads to nodules and abscesses in various organs, with most common acute clinical signs being fever, diarrhea, pneumonia and blood infection (*16*). Treatment consists of a combination of antimicrobial therapies. It is rarely curative, and relapses are common (*16*).	Humans most commonly become infected from contaminated environment. Dog to human transmission has been documented, although it is believed to be exceptionally rare (*15*,*16*). Bacteria are shed from infected wounds and other secretions, depending on the site of infection (e.g., urine, feces, nasal secretion, if those sites are infected) (*16*).	Similar to dogs, human disease (melioidosis) is almost always immunocompromised individuals or those with comorbidities (kidney disease, diabetes, cancer), with a case fatality rate of 10 to 40% (*15*). It is nicknamed the "Great Mimicker" due to a variety of non-specific clinical signs. Disease may present as skin ulcers, chronic abscesses (particularly in the spleen and liver), chronic pneumonia, osteomyelitis, arthritis, acute, fulminant bacteremia leading to pneumonia and encephalitis (*15*). Similar to dogs, treatment is rarely curative. An aggressive combination of antimicrobials is required, and relapses are common (*15*).	
Campylobacter jejuni & C. upsaliensis (Campylo-bacteriosis) UNCERTAINTY = Low	Bacteria	Fecal-oral transmission via direct contact with feces of infected dog or indirect contact through consumption of contaminated food, water, and the environment (*17*).	Most common is mild and self-limiting diarrhea. Severe enteritis can occur in a subset of cases that is accompanied with dehydration, fever, abdominal pain, and lethargy (*17*). Subclinical infection is common (*17*).	Fecal-oral transmission via direct contact with feces of infected dog or indirect contact through consumption of contaminated food, water, and the environment (*18*). Shed in human feces for weeks after recovery (*19*).	Most significant cause of bacterial gastroenteritis (*17*). Children, elderly, and immunocompromised are most severely affected (*17*). Extraintestinal signs may occur, including hepatic disease, meningitis, and Guillain-Barre syndrome (*17*).	
Capnocytophaga canimorsus & C. cynodegmi (Capnocytophaga) UNCERTAINTY = Low	Bacteria	Direct contact between saliva containing *Capnocytophaga* spp. and broken skin (*20*).	Localized skin infection can occur, but very rarely (*21*,*22*).	Among *Capnocytophaga* infections reported in humans, 54% of cases are associated with dog and cat bites, 8.5% with scratching, and 27% with close contact with the animal (i.e., licking a wound) (*20*).	Although C*apnocytophaga* cases are infrequently reported, cases that do occur involve multiple systems and are associated with mortality in 30% of cases (*20*). The most common manifestations of *Capnocytophaga* spp. infections are fever/chills, abdominal cramping/diarrhea, vomiting, headaches, confusion, and muscle aches and pain (*20*). Other symptoms that have been reported range from localized swelling and abscesses around the wound site to sepsis, endocarditis, and meningitis (*21*,*23*).	

HAZARD	CATEGORY	SPREAD SCENARIO CANINE	IMPACT ANIMAL	SPREAD SCENARIO HUMAN	IMPACT HUMAN	OTHER INFORMATION
					Human disease is most common in at-risk groups, including individuals with a history of alcohol use, those without a functioning spleen or asplenic and immunosuppression (*20*).	
Ehrlichia canis (Canine ehrlichiosis) UNCERTAINTY = Low	Bacteria	Tick-borne transmission (*24*). The brown dog tick (*Rhipicephalus* species complex) is the primary vector. Dogs can be sub-clinically infected and a source of bacteria for feeding ticks (*24*).	Three stages can occur (*24*). The acute stage is characterized by fever, lethargy, inappetence and weight loss. Ocular and nasal discharge are common as well as uveitis. Petechial hemorrhages and other bleeding tendencies, as well as neurologic signs may be present (*24*). The sub-clinical phase can occur if dogs spontaneously recover or recover following inadequate treatment. This phase can last for years and may progress to the chronic phase (*24*). The chronic phase is only seen in a subset of dogs and is generally associated with poor prognosis. Chronically infected dogs may have pancytopenia leading to bleeding tendencies and secondary infections. Ocular abnormalities and neurologic signs are common, along with overall poor body condition (*24*).	Not considered to be zoonotic (*25*).	N/A	Vector is not established in Canada (*26*).
Ehrlichia ewingii (Canine ehrlichiosis) UNCERTAINTY = Low	Bacteria	Tick-borne transmission (*24*). *Amblyomma americanum* is the primary vector. Naturally and experimentally infected dogs can maintain infection for 5 mo to 2 y (*27*).	Subclinical infection common. If signs are present, they are generally mild and include fever, lethargy, anorexia, and arthritis (*24*).	Vector-borne transmission predominates, although blood-borne transmission cannot be ruled out (*24*,*28*). Humans are incidental hosts (*29*).	Human infection with *EE* is rare and typically only in immunosuppressed individuals (*24*,*30*). Presents as a flu-like illness with thrombocytopenia (*24*). Responds well to antibiotics treatment (*24*).	Vector is not established in Canada. Adventitial introductions do occur (*31*).
Leptospira interrogans & Leptospira Kirschneri (Leptospirosis) UNCERTAINTY = Low	Bacteria	Direct transmission via contact with contaminated urine (*32*). OR Indirect transmission via contact with water (stagnant or slow-moving water such as puddles, ponds and lakes), soil, food or bedding contaminated with infected urine (*33*). OR Ingestion of tissues or carcass of infected host (*32*). OR Breeding with an infected dog (rare) (*34*). OR	Clinical signs in dogs range from subclinical to severe with acute kidney injury, liver damage and respiratory disease. In the most severe cases, the infection may result in death (*36*). Clinical signs can include fever, shivering, muscle tenderness, lethargy, polydipsia, change in quantity or frequency of urination, dehydration, vomiting, diarrhea, anorexia, jaundice and inflammation of the eyes (*37*). Kidney failure can occur with or without liver failure. Infection may also cause bleeding disorders, which present as blood in the urine, stool, saliva or vomitus and petechial hemorrhages (*37*).	Direct transmission via contact with contaminated urine or body fluids. OR Indirect transmission via contact with contaminated water via recreational or work activities (*38*).	Leptospirosis can cause a wide range of symptoms (*38*). The first phase of the infection can cause mild influenza-like symptoms such as fever, headaches, chills, muscle aches, vomiting. The patient can recover but will become ill again (*39*). The second phase is more severe and can cause kidney, liver or respiratory failure as well as meningitis (*38*). Though uncommon, infection during pregnancy may result in fetal death, abortion, stillbirth or congenital leptospirosis (*38*).	Underdiagnosed as symptoms can be mistaken for other diseases both in animals and humans and the disease may be mild (*38*,*39*). Leptospirosis can only rarely be transmitted from human to human, either through sexual intercourse, transplacental transmission and through breast feeding (*38*).

HAZARD	CATEGORY	SPREAD SCENARIO CANINE	IMPACT ANIMAL	SPREAD SCENARIO HUMAN	IMPACT HUMAN	OTHER INFORMATION
		Infected bitch transfers bacteria to puppies during pregnancy (*34*).<br>OR<br>Through bite wounds/damaged skin (*35*).	Treatment requires antibiotics and supportive care. Surviving dogs may have permanent kidney or liver damage (*33*).		Treatment involves a course of antibiotics that helps reduce the length and severity of the infection (*26*).<br>Case fatality rates worldwide range from <5% to 70%; however, this range is not reliable as occurrence of disease is not well documented in many areas and mild cases may not be recorded (*38*).	
Mycoplasma hemocanis<br>UNCERTAINTY = Moderate	Bacteria	Natural mode of transmission is uncertain (*40*).<br>Possible modes of transmission include:<br>Infected blood (aggressive interaction, transfusion) (*41*).<br>OR<br>Blood-sucking arthropods (Rhicephalus sanguineus) is a suspected vector (*40*).<br>OR<br>Vertical transmission (possibly) (*40*,*41*).<br>OR<br>Horizontal transmission between dogs (possibly) (*42*).	Causes acute hemolysis in dogs that are splenectomized, but infections are usually asymptomatic in healthy dogs (*43*).<br>Splenectomized dogs may have agglutination, spherocytosis, and a positive Coombs' test. Clinical signs may include lethargy, anorexia, and fever, with splenomegaly and icterus occurring less often (*43*).<br>Infections can be treated with Doxycycline (*41*).<br>May require supportive treatment with IV fluids or blood products to provide oxygen-carrying capacity. Prognosis is generally good if effective treatment is given promptly (*40*).	Not known to affect humans	N/A	
Rickettsia conorii subsp. conorii<br>(Mediterranean Spotted Fever)<br>UNCERTAINTY = Low	Bacteria	Tick-borne transmission via the bite of an infected brown dog tick (*Rhipicephalus* species complex) (*44*).<br>Foci of transmission within households has been documented (*45*).	Subclinical infection is the most common. Fever, anorexia, and lethargy can occur and are generally self-limiting (*46*).	Tick-borne transmission via the bite of an infected brown dog tick (*Rhipicephalus* species complex) (*44*).	Non-specific febrile illness characterized by fever, skin rash and eschar (*47*). Infection is typically mild, although severe and fatal cases have been reported, particularly in those who receive delayed treatment, the elderly, and immunocompromised individuals. Complications associated with *R. conorii* subsp. *conorii* infections may include cardiac, respiratory, neurologic, and renal involvement (*45*).	
Microsporum canis and gypseum<br>***Trichophyton mentagrophytes***<br>(Ringworm)<br>UNCERTAINTY = Moderate	Fungus	Direct contact with arthrospores in the hair or skin of an infected canine (*48*).<br>OR<br>Direct contact with contaminated fomites (*48*).	Most cases are caused by M. canis (70%), followed by M. gypseum (20%) and T. mentagrophytes (10%) (*49*).<br>Ringworms is most commonly reported in young and immunosuppressed hosts, although animals of any age can be affected (*49*).<br>Asymptomatic infections are more common in adult animals (*49*).<br>Symptomatic infections involve single or multiple circular lesions with raised margins commonly associated with regions of	All 3 are zoonotic and these fungal species have been isolated from human infections (*48*).<br>Direct contact with infected human (*51*).<br>OR<br>Direct contact with infected canine (*51*).<br>OR<br>Direct contact with contaminated fomites (*51*).	The most common zoophilic dermatophyte implicated in human disease is M. canis (*48*).<br>Presents as superficial erythematous, scaly lesions on the scalp or body, referred to as tinea capitis and tinea corporis, respectively (*48*).<br>Although inflammation may occur, not all cases elicit an inflammatory response (*52*).<br>Tinea is often self-limiting lasting weeks to months; however, cases of	

HAZARD	CATEGORY	SPREAD SCENARIO CANINE	IMPACT ANIMAL	SPREAD SCENARIO HUMAN	IMPACT HUMAN	OTHER INFORMATION
			alopecia and hyperpigmented, scaly patches (*50*). Can be associated with persistent scratching of the infected area due to pruritus, which can lead to secondary infection (*51*). In healthy animals, ringworm is often self-limiting within weeks to months, with regions of alopecia and hyperpigmentation typically reversible (*48*,*49*). Disseminated infections are rare (*48*). Treatment with a systemic or topical antifungal therapy can speed up recover and reduce spread of fungus into the environment (*50*).		tinea capitis have persisted up to several years without intervention (*52*). Young children (<5 y of age) and individuals with immunocompromised conditions are at increased risk for more severe dermatophyte infections, including extensive skin infections and subcutaneous abscesses (*48*,*52*). Disseminated disease has also been reported, although cases are sporadic (*48*). These dermatophytes' communicability ranges from low to moderate (*53*). Can usually be treated with non-prescription antifungal creams, lotions or powders applied over 2–4 weeks. Prescription antifungal medication can be needed for persistent infections (*54*).	
**Cancer cells of canine transmissible venereal tumor** (Transmissible venereal tumor) UNCERTAINTY = Low	Transmissible neoplasia	Direct skin-to-skin contact with the tumorous growths, which usually occurs during coitus (*55*). OR May also be transmitted through socialization (i.e., licking, sniffing) (*55*). OR Parturition (*55*).	Clinical appearance depends on location of the tumor(s) (*56*). Hemorrhagic discharge common for mucosal, membrane-based tumors on genitals or oral/nasal cavities (*56*). Tumours are usually observed as 'cauliflower-like' nodules that appear red to flesh-colored (*57*). Depending on tumor size, genital protrusions may be observed, mostly in female dogs. Penile tumors typically present as enlargement of the inguinal lymph nodes (*57*). Tumours may cause pain and discomfort associated with the hemorrhaging and serosanguineous discharge of the external genitalia (*57*). CTVT is generally localized to the original site of implantation - usually does not impair overall health unless becomes necrotic, infected or blocks the urethral orifice (*57*). Metastases are rare (roughly 5% of cases) - occurs especially in neonatal and immunocompromised dogs (*56*). Metastasis is usually in the regional lymph nodes but has also been reported in the skin, brain, eye, liver, spleen, testes and muscle (*57*). CTVT may resolve itself on its own, but this rarely occurs (*58*).	There is no evidence to suggest CTVT affects humans	No risk of zoonotic transmission	

HAZARD	CATEGORY	SPREAD SCENARIO CANINE	IMPACT ANIMAL	SPREAD SCENARIO HUMAN	IMPACT HUMAN	OTHER INFORMATION
			Chemotherapy with vincristine sulfate is the most effective therapy - >90% of treated cases recover fully (*55*,*59*).			
Alaria spp. (Intestinal flukes) UNCERTAINTY = Low	Parasite	Ingestion of infected frog (intermediate host) OR Ingestion of infected paratenic host (*60*).	Adult *Alaria* in their carnivore definitive hosts are not usually associated with adverse clinical effects (*61*). *Alaria* spp. adults develop in the small intestine and are not usually associated with intestinal illness. However, migration of immature *Alaria* through the lungs may result in pulmonary hemorrhage and respiratory compromise (*62*). No drugs are labeled for *Alaria,* however praziquantel and epsiprantel are often used (*60*,*62*).	No direct transmission risk between dogs and humans (*60*). Humans can get infected by ingesting raw/undercooked frogs (*60*).	A few human infections with *Alaria* sp. have been documented and at least two human fatalities caused by infection with *A. americana* have been reported (*60*). Human infections have been associated with ocular disease and rarely death resulting from damage to the lungs caused by the migrating immature flukes (*61*).	
Ancylostoma caninum, A. braziliense, A ceylanicum Uncinaria stenocephala (HOOKWORM) UNCERTAINTY = Low	Parasite	Ingestion of free larvae from the environment (*63*,*64*) OR Ingestion of an infected prey paratenic host (*63*,*64*) OR Skin penetration by larvae at the paw level (*63*,*64*) OR Transmammary transmission (only applicable for *A. caninum*) (*63*,*64*).	-Peracute disease is seen in neonatal puppies infected by *A. caninum* through the transmammary route. Pups appear healthy in the first week of life, then rapidly deteriorate and die at 2–3 wks of age (*63*). -Older pups/adults exposed to overwhelming numbers of larvae may have diarrhea with melena/mucus, mucosal pallor, lethargy, inappetence, emaciation, poor haircoat, anemia (*63*,*64*). -Well-nourished and immunocompetent dogs with small numbers of worms may show few, if any signs of disease (*64*). - *U. stenocephala* is most commonly associated with dermatitis, typically on the paw pads, but may also be observed on the ventral thorax, abdomen, and limbs (*63*). -The prognosis for recovery from acute and chronic hookworm disease is good to excellent after effective anthelmintic treatment (*63*).	Skin penetration by larvae (usually through the foot, legs, buttocks, or back) from contaminated environments (*65*).	Can cause cutaneous larva migrans. From the point of entry, the hookworm burrows along a haphazard tract, leaving a winding, threadlike, raised, reddish brown rash. The rash itches intensely. Small bumps and blisters may also occur. Often, scratching of the bumps or blisters results in a bacterial infection of the skin (*66*). -*A. braziliense* is responsible for most cases of cutaneous larva migrans in humans. *A. caninum, A. ceylanicum*, and *U. stenocephala* are involved less frequently (*67*). People are considered aberrant hosts in which the parasite does not generally complete its life cycle. CLM is self-limiting in humans (*68*).	
Angiostrongylus vasorum Angiostrongyliasis / French heartworm UNCERTAINTY = Low	Parasite	Ingestion of infected intermediary hosts	Disease ranges from subclinical to fatal (*69*). May cause cardiopulmonary disease, central nervous system disease or coagulopathies separately or in conjunction (*69*). Clinical signs are usually chronic and seen months after the initial infections (*70*). Clinical signs can include exercise intolerance, labored breathing, coughing, anorexia and weight loss (*70*). There are no licensed anthelmintic treatments for *A. vasorum* in Canada (*71*).	Not considered zoonotic (*71*).	No known impacts on humans	

HAZARD	CATEGORY	SPREAD SCENARIO CANINE	IMPACT ANIMAL	SPREAD SCENARIO HUMAN	IMPACT HUMAN	OTHER INFORMATION
Babesia canis spp. -*B. vogeli, rossi, canis*<br>***B. gibsoni***<br>UNCERTAINTY = Low	Parasite	Infected tick (most common) (*72*,*73*).<br>OR<br>Dog-to-dog transmission via dog bites/dog fighting (main mode of transmission of *B. gibsoni* in North America and Europe where competent tick vectors are not widespread) (*72*,*73*).<br>OR<br>Vertical (transplacental) transmission (especially *B. gibsoni* in North America and Europe where competent tick vectors are not widespread) (*72*,*73*).<br>OR<br>Blood transfusion (*72*,*73*).	May present with a wide variety of clinical signs ranging in severity from sudden collapse with systemic shock, to a hemolytic crisis, to a subtle and slowly progressing infection with no apparent clinical signs (*74*).<br>Often present with the acute and severe form of babesiosis, characterized by abnormal dark urine, fever, weakness, pale mucous membranes, depression, swollen lymph nodes, splenomegaly (*74*).<br>Blood results may show anemia, thrombocytopenia, hypoalbuminemia, bilirubinemia, bilirubinuria (*73*,*74*).<br>Prognosis depends on which body systems are affected at the time of diagnosis. Dogs who survive babesiosis often remain sub-clinically infected and may suffer a disease relapse in the future or serve as a source for further spreading disease (*74*).<br>Treatment:<br>A combination of azithromycin (antibiotic) and atovaquone (quinone antimicrobial medication) for *B. gibsoni* (*73–75*).<br>Imidocarb dipropionate injections for *B. canis* spp (*73*,*76*).<br>Blood transfusions and IV fluids depending on severity (*77*).	Not zoonotic (*73*).	N/A	
Crenosoma vulpis, Eucoleus (Capillaria) aerophilus<br>(Canine lungworm)<br>UNCERTAINTY = Low	Parasite	<u>*C. vulpis*</u><br>Ingestion of infective third-stage larvae in the tissues of the terrestrial gastropod intermediate hosts (slugs, land snails) (*78*).<br>OR<br>Ingestion of infective third-stage larvae released into the environment (*78*).<br><u>*E. aerophilus*</u><br>Ingestion of infective larvated egg from environment (fecal oral) (*78*)<br>OR<br>Ingestion of an infected earthworm (IH) (*78*).	<u>*C. vulpis*</u><br>-In dogs, clinical disease is seldom more than a persistent cough. Third stage larvae can cause pneumonia and adults in the lung can cause bronchitis and bronchiolitis (*79*).<br><u>*E. aerophilus*</u><br>-Light infestations with *E. aerophilus* are usually asymptomatic. Moderate to severe infections can result in bronchitis, nasal discharge, wheezing cough, sneezing, dyspnea, bronchopneumonia, abscesses in the lungs, emphysema, or secondary bacterial infections which can be fatal in younger animals (*80*).<br>Treatment:<br>No products are labeled for this parasite within Canada, but fenbendazole and macrocyclic lactones (moxidectin, milbemycin, eprinomectin), should be effective (*80*).	<u>*C. vulpis*</u><br>-Does not infect humans<br><u>*E. aerophilus*</u><br>-Ingestion of infective larvated eggs from contaminated environment<br>-Human infection is very rare. <20 cases exist in the literature (*78*).	<u>*C. vulpis*</u><br>N/A<br><u>*E. aerophilus*</u><br>-Clinical symptoms include bronchitis, coughing, mucoid or blood-tinged sputum, fever, dyspnea, and eosinophilia (Lalosevic et al., 2008).	

HAZARD	CATEGORY	SPREAD SCENARIO CANINE	IMPACT ANIMAL	SPREAD SCENARIO HUMAN	IMPACT HUMAN	OTHER INFORMATION
Cryptosporidium canis UNCERTAINTY = Low	Parasite	Ingestion of sporulated oocyst from coprophagia, grooming, contaminated food, or water (*81*).	Many infected dogs remain asymptomatic even though they might continue to shed oocysts for months. Typically resolves without treatment in healthy dogs (*82*,*83*). Puppies and immunocompromised dogs may need medications and IV fluids to correct dehydration caused by diarrhea. Medications such as azithromycin or paromomycin may be prescribed in persistent cases (*83*). When symptoms are present, they may include diarrhea, lack of appetite, fever, lethargy (*83*). Prognosis is good. Puppies and immunocompromised dogs also have a good outcome if they receive appropriate treatment (*83*).	Cases are RARE in humans. Most human infections have been in immunosuppressed individuals (*82*,*84*,*85*). Possibly from ingestion of oocysts from canine feces (*85*). *C. canis* is of low zoonotic risk to humans (*86*).	A case study examining *C. canis* infection in two children from Peru reported transient diarrhea in both children (*87*).	
Dioctophyma renale UNCERTAINTY = Moderate	Parasite	Eating raw fish, frogs, or earthworms infected with *D. renale* (*88*,*89*).	*D. renale* almost always resides in the right kidney but can also be found free in the abdomen (*90*). Dogs are usually asymptomatic because often one kidney can serve the entire body. *D. renale* is usually an incidental finding (*90*). Sometimes, the worm can destroy the kidney. Can cause obstruction, hydronephrosis, and destruction of the renal parenchyma. Kidney failure can result if both kidneys are parasitized. Clinical signs are hematuria, pollakiuria, weight loss, and renal or abdominal pain (*88*). Treatment: surgical removal of the worm. Sometimes the kidney has been devastated by the worm and needs to be removed (*91*,*92*).	Human infections are very rare (*89*). Humans can become incidental hosts after eating undercooked paratenic hosts (e.g., fish, frog) (*89*). Approximately 20 cases of human infection have been reported worldwide (*93*).	Eggs or adult worms expelled in urine, usually accompanied by hematuria, and sometimes abdominal pain, fever, and eosinophilia. Adult worms have been found in the right kidney, left kidney, both kidneys, retroperitoneal space, and liver. Can lead to the destruction of the kidney if left untreated (*89*). Treatment: surgical removal of worm and affected organ or tissue (*94*).	
Diphylidium caninum (Flea tapeworm) UNCERTAINTY = Low	Parasite	Ingestion of infected flea during grooming or from consumption of prey carrying infected fleas (*95*).	Infection with *D. caninum* is not usually harmful. Most infected dogs are asymptomatic (*96*). Occasionally dogs may experience anal pruritis, mild gastrointestinal disturbances or other non-specific clinical signs (*96*,*97*).	Risk of human infection is very low as ingestion of an infected flea is required. For this reason, most cases are in children between the age of 1–5 y old (*96*,*98*).	Human infection is rare; however, cases have been reported on every continent (*96*). Human infections are usually asymptomatic. Some children may experience mild clinical signs such as diarrhea and abdominal pain (*99*,*100*). Treatment with praziquantel is administered orally to dissolve the tapeworm (*96*).	Intermediate host (flea) prevalence and endemicity may be influenced by climate change, urbanization and the increasing number of pets (*97*).
Dirofilaria immitis (Canine heartworm) UNCERTAINTY = Low	Parasite	Mosquito takes a blood meal from infected host and ingests microfilaria à after process of maturation, the mosquito	The pulmonary artery becomes enlarged and tortuous from inflammation (*102*). Lungs become inflamed and, if chronic, can lead to fibrosis (*102*). Dead worms may also	Mosquito takes a blood meal from infected host and ingests microfilaria after process of maturation the mosquito	Humans are not suitable hosts for the parasite. Most larvae that migrate to the heart will die (*105*). When the parasite dies, it can obstruct	Risk factors for canine heartworm include size of the dog population in an area, prevalence of *D.*

HAZARD	CATEGORY	SPREAD SCENARIO CANINE	IMPACT ANIMAL	SPREAD SCENARIO HUMAN	IMPACT HUMAN	OTHER INFORMATION
		transmits microfilaria to domestic dog<br>*D. immitis* cannot be transmitted directly dog to dog (*101*).	obstruct blood flow should they become lodged in pulmonary vasculature (*102*).<br>Most infected dogs are asymptomatic; however, clinical signs generally develop as disease progresses (*103*).<br>Clinical symptoms of heartworm may include a persistent cough, hemoptysis, exercise intolerance, dyspnea, congestive heart failure, epistaxis, ascites, and anorexia (*104*). Sudden death is possible (*102*).	transmits microfilaria to a human (*105*).<br>Infections cannot be transmitted directly from dog to human (*105*).	pulmonary vessels leading to infarction (*103*).<br>Most patients do not show symptoms (*103*). Often it produces pulmonary disease and infected persons present with one or multiple pulmonary nodules (*103*).<br>Some individuals may display specific or non-specific signs such as coughing, hemoptysis, chest pain and wheezing (*103*).<br>Peripheral eosinophilia is only present in 6.5%–15% of cases (*103*).	*immitis* infection in the dog population, and density of mosquito population (*103*). Ownership does not appear to be a risk factor (*103*).
Echinococcosis vogeli<br>(Polycystic neotropical echinococcosis)<br>UNCERTAINTY = Low	Parasite	Ingestion of larval cysts from raw paca meat (hunting dogs are sometimes rewarded with the raw viscera of pacas) (*106*,*107*).	Dogs rarely experience clinical signs (*108*).	Ingestion of *E. vogeli* eggs. Domestic dogs, in expelling eggs of *E. vogeli*, appear to be the sole source of risk to people (*109*).<br>Humans are dead-end hosts (*110*).	Affects mainly the liver, where it acts as a slow growing tumor; secondary cystic development is common in abdominal or thoracic structures (*111*).<br>Symptoms depend on the location of cysts. For example, cysts in liver may cause jaundice, abdominal tenderness and pain, fever, and/or anaphylactic shock. Cysts in lungs may cause shortness of breath, coughing, and/or chest pain (*108*).<br>Mortality high without treatment (*112*).<br>Treatment: Albendazole (chemotherapy) followed by surgery to remove cysts has shown success (*106*,*108*,*113*).	Patients infected with *E. vogeli* are typically born or have lived for prolonged periods, in rural tropical areas of continental south America, particularly in regions with abundant wildlife (*113*).
Echinococcus granulosus<br>(Granulomatous / cystic echinococcosis)<br>UNCERTAINTY = Low	Parasite	Dogs typically acquire it if they are fed offal or can scavenge infected sheep, horse or camel carcasses containing EG cysts (*114*).	Adult tapeworms can live for up to 3 y in dogs and usually stop laying eggs after 6–10 mo (*108*).<br>Large numbers of parasites may cause enteritis and diarrhea in DHs but this is rare (*108*).<br>DHs can be treated with various anthelminthic drugs (*108*).	Fecal oral route by ingestion of food or water contaminated w/ eggs or via hands contaminated w/ egg-containing soil, sand or hairs of infected dogs (*115*).<br>Copraphagic flies, arthropods or other animals may function as mechanical vectors for egg transmission (*115*).	Infected individuals are often asymptomatic for years (months to years) until cysts are large enough to cause symptoms (*111*).<br>The rate at which symptoms appears varies with cyst location (*111*).<br>The liver (>65%) and lungs (25%) are most common locations for cysts. Other sites include the spleen, kidneys, heart, bone and central nervous system (*111*,*115*).<br>Cyst rupture can result in the host going into anaphylactic shock (*111*).<br>Most human infections remain asymptomatic (*115*).<br>Most infections can be eliminated by benzimidazole compounds such as albendazole; however, some strains are resistant and require praziquantel and avermectins (*115*).	Eggs are resistant to phenol, aldehydes and ethanol disinfectants (*115*). They can survive weeks/months in the environment in the right conditions but are easily destroyed by direct sunlight (*108*,*115*).<br>As of 2020, *E. granulosus* s.s., *E. canadensis* and *E. ortleppi* have been detected in people (*108*).<br>No human-human transmission has been reported (*115*).

HAZARD	CATEGORY	SPREAD SCENARIO CANINE	IMPACT ANIMAL	SPREAD SCENARIO HUMAN	IMPACT HUMAN	OTHER INFORMATION
					Diagnosis may be achieved through imaging (radiographs, ultrasound, CT scan). If a cyst ruptures, protoscoleces and brood capsules may be found in vomitus, feces or urine. Serology is 80%–100% sensitive and 88%–96% specific for liver disease (*116*).<br>Treatment includes surgical removal of intact cysts, if possible. Chemotherapy is recommended 4 weeks before and 1 mo post-surgery (*115*).<br>In patients with inoperable cysts, percutaneous puncture using U/S and injection of a protoscolicidal agent for at least 15 min followed by re-aspiration is performed (*115*).	
Echinococcus multilocularis<br>(Alveolar Echinococcosis)<br>UNCERTAINTY = Low	Parasite	Ingestion of cyst-containing organs from intermediate host (small mammal) infected with EM (*111*).	Intestinal infections in dogs are subclinical (*117*).<br>Domestic dogs can act as either a DH or an IH (sometimes at the same time, roughly 30%) (*118*).<br>Definitive hosts (foxes, coyotes, jackals, etc.) are asymptomatic (*118*).<br>Dogs that act as the IH often present initially with anorexia, vomiting and abdominal distension. This may progress to signs of hepatic failure due to large cystic structure developing in the liver. Other organs can be impacted as well (*119*).	Ingestion of eggs shed in feces of infected definitive hosts such as foxes, coyotes, dogs, which likely occurs from hand to mouth contact with fecal matter or by ingesting contaminated food, water (*111*).	Greater health risk than *E. granulosis*. Causes parasitic tumors in liver, brain, and other organs (*111*).<br>Humans are dead-end intermediate hosts. When left untreated 70%–100% of cases are fatal (*118*).<br>Incubation period is between 5–15 y in humans with slow development of lesion primarily in the liver.<br>Metastasis to other organs such as the lungs and brain also occurs, making surgical cure rare except in very early stages of disease (*118*).<br>The 10-y survival rate for long-term parasitic drug treatment is ≈80% (*118*).<br>Most cases are diagnosed later in the disease stage, when severe risks or death are increased (*118*).	
Filaroides hirthi, F. osleri<br>(Lung / bronchial worm)<br>UNCERTAINTY = Moderate	Parasite	Ingestion of larvae in fresh fecal material<br>OR<br>Ingestion of larvae in sputum, saliva, or vomitus (*78*).	<u>*F. Hirthi*</u><br>-Usually infection is not associated with clinical signs (*78*).<br>- Clinical signs are usually associated with immunosuppression or comorbid diseases. Through autoinfection, such dogs develop massive hyperinfections with life-threatening granulomatous pneumonia. Most often, affected dogs develop a non-productive cough that progresses to dyspnea, tachypnea, cyanosis, and sometimes death (*78*).<br><u>*F. osleri*</u>	Not zoonotic (*78*).	N/A	

HAZARD	CATEGORY	SPREAD SCENARIO CANINE	IMPACT ANIMAL	SPREAD SCENARIO HUMAN	IMPACT HUMAN	OTHER INFORMATION
			- Major sign is the spasmodic attack of a hard, dry cough started by exercise or exposure to cold air (*78*,*120*).<br>- In some cases, wheezing, dyspnea, and cyanosis occur. Severely affected dogs will show weight loss, emaciation, collapse, and even death (*78*).<br>- Subclinical infections occur in some dogs (*78*).<br>Treatment:<br>-There is no anthelmintic approved for use in the treatment of *Filaroides* spp. infection in dogs. Fenbendazole has been used to successfully treat several dogs with clinical disease signs due to *F. hirthi* infection (*78*).			
Giardia duodenalis **(assembles A-D)**<br>UNCERTAINTY = Low	Parasite	Ingestion of feces (fecal oral route) from contaminated water, soil, food, or objects (*121*).	Patients will often be asymptomatic and still have a normal appetite and energy levels (*122*).<br>Clinical signs of Giardiasis:<br>- Acute or sudden diarrhea<br>- Soft or watery stool with mucus and a foul odor<br>- Abdominal discomfort (*123*)<br>In severe cases, dogs may experience lethargy, decreased appetite or weight loss (*123*).<br>Treatment:<br>-Fenbendazole, metronidazole, prescribed diet to resolve diarrhea (*122*,*123*).	Transmission from dogs to humans appears to be rare (*124*).<br>Assemblages A and B can infect humans via the fecal-oral route; however, dogs are most commonly infected with assemblages C and D (*121*).	Some people remain asymptomatic. Symptoms may include diarrhea, gas, stomach pain, nausea, vomiting (*125*). Signs and symptoms may last two to six weeks, but in some people, they last longer or recur (*125*).	
Hepatozoon americanum<br>(American canine hepatozoonosis)<br>UNCERTAINTY = Low	Parasite	Ingestion of infected *A. maculatum* tick<br>OR<br>Ingestion of infected paratenic prey host (*126*).	-Most dogs exhibit moderate to severe clinical signs even in the absence of concurrent disease or immunosuppression. Disease is debilitating and often fatal (*126*,*127*).<br>-Clinical signs of infection are associated with the strong inflammatory response that occurs when meronts rupture, leukocytes are recruited, and pyogranulomas form in skeletal muscle (*127*).<br>- Disease is characterized by periodic or persistent fever, weakness, muscle atrophy, generalized pain or hyperesthesia, reluctance to move, mucopurulent ocular discharge, and gradual deterioration of body condition (*127*).<br>-No treatment is effective in eliminating *H. americanum* in infected dogs. Treatment can increase survival time, improve the quality of life, and decrease the	Not zoonotic (*127*).	N/A	

HAZARD	CATEGORY	SPREAD SCENARIO CANINE	IMPACT ANIMAL	SPREAD SCENARIO HUMAN	IMPACT HUMAN	OTHER INFORMATION
			number and severity of clinical relapses (*127*).			
Hepatozoon canis (OLD WORLD HEPTAZOONOSIS) UNCERTAINTY = Low	Parasite	Ingestion of infected *R. sanguineus* tick and possibly other tick species OR Transplacental transmission (*126*).	-Infection varies greatly in severity from inapparent to severe and life-threatening infection, although dogs are most often subclinically or mildly affected (*126*). - A compromised immune status tends to lead to more severe disease. In patients with overt disease, clinical signs including fever, anemia, lethargy, and anorexia may be observed (*126*).	Not known to infect humans	N/A	
Heterobilharzia americana UNCERTAINTY = Low	Parasite	Direct skin penetration of cercariae from a freshwater aquatic environment (*128*,*129*).	May be asymptomatic or have systemic illness due to granulomatous inflammation (caused by migration of eggs) within the gastrointestinal tract, lungs, liver, lymph nodes, pancreas, and spleen (*60*,*128*,*129*). Clinical signs include diarrhea (which may be blood-tinged), vomiting, anorexia, weight loss, lethargy, and polyuria/polydipsia (*128*). Laboratory finding include hypoalbuminemia, hypoglobulinemia, hypercalcemia, azotemia, anemia, and eosinophilia (*128*). Debilitation associated with severe lesions in some chronic infections may result in fatalities (*128*). Treatment is often unrewarding. Severe lesions, including extensive fibrosis, may already be present when clinical signs develop. While treatment is not always effective, a combination of praziquantel and fenbendazole may result in resolution of clinical signs in some infected dogs (*128*).	*H. americana* in dogs does NOT pose a zoonotic risk to humans (*128*). Humans can become infected form free cercariae released in water from snail hosts (*128*).	Can cause a self-limiting dermatitis in humans (“swimmer’s itch”) (*128*).	
Leishmania infantum / L. Braziliensis (Leishmaniasis) UNCERTAINTY = Low	Parasite	Primarily transmitted from one infected dog to another by the bite of a phlebotomine sandfly (*Lutzomyia* and *Phlebotomus* spp.) (*130*). Vertical transmission as well as transmission via breeding and blood transfusion have been documented (*130*). Dog to dog contact (bites, licks) has been suspected (*130*). Dogs remain infected and infective (to varying degrees) for life (*131*).	Disease severity ranges from asymptomatic to fatal (*131*). Visceral and cutaneous involvement is common in dogs showing clinical signs (*130*). Clinical signs include weight loss, lethargy, fever, generalized lymph-adenomegaly, splenomegaly, vomiting, diarrhea, lameness, epistaxis, polyuria and polydipsia, and cutaneous and ocular lesions (*132*,*133*). Advanced stages of disease, in which prognosis is guarded, present with vasculitis, polyarthritis, uveitis, and glomerulo-nephritis leading to kidney failure and death (*130*).	Zoonotic (*130*). Primary mode of transmission is via the bite of an infected phlebotomine sandfly (*134*). Sandflies species that transmit Leishmaniasis are NOT present in Canada (*135*). Cannot rule out blood-borne transmission from an infected dog, but if it occurs, it is rare	Causes visceral leishmaniosis in humans, which is almost always fatal if left untreated (*135*). Characterized by recurrent fever, weight loss, splenomegaly, hepatomegaly, and anemia (*135*).	

HAZARD	CATEGORY	SPREAD SCENARIO CANINE	IMPACT ANIMAL	SPREAD SCENARIO HUMAN	IMPACT HUMAN	OTHER INFORMATION
Nanophyetus salmincola and ***Neorickettsia helminthoeca*** (SALMON POISONING FLUKE***)*** UNCERTAINTY = Low	Parasite	Ingestion of encysted metacercariae within uncooked or undercooked salmonid fish (*136*).	Adult *N. salmincola* in their definitive hosts are essentially non-pathogenic, although there have been reports of clinical GI signs in very heavily infected dogs (*137*). However, *N. salmincola* flukes are the vector for *Neorickettsia helminthoeca*, an intracellular endosymbiotic rickettsial bacteria that is the cause of Salmon Poisoning Disease (SPD) in dogs and other canids. SPD is a severe and commonly fatal hemorrhagic gastro-enteritis (*136*,*137*). The treatment of choice for SPD is doxycycline, tetracycline, or oxytetracycline. Although complete clinical recovery may occur without anthelmintic treatment, the fluke infection should be treated with praziquantel after initial recovery from SPD (*136*).	Ingestion of encysted metacercariae within uncooked or undercooked salmonid fish (*136*). Infected dogs do not pose a direct zoonotic infection risk to people (CAPC, 2012).	Human infection with the fluke *N. Salmincola* has been reported occasionally in people in the U.S (*137*). Most humans are subclinically infected, although self-limiting abdominal discomfort, diarrhea, vomiting, weight loss, nausea, and peripheral eosinophilia may occur (*136*). There are no reports of human infection with *Neorickettsia helminthoeca* (*136*).	
Neospora caninum (Neosporosis) UNCERTAINTY = Low	Parasite	Ingestion of placental tissues, aborted fetuses, uterine fluid or dead tissue from an infected intermediate host (*138*).<br><br>OR<br><br>Transmammary transmission through colostrum or milk. Not all pups will become infected or show clinical signs (*138*).<br><br>OR<br><br>Ingestion of oocysts from the feces of definitive hosts (dogs and coyotes) – not confirmed (*138*).	The majority of infected dogs do not display any symptoms (*138*). Clinical signs are most often seen in dogs less than 1 y of age but can occur in dogs of all ages (*138*). Infections seem to be more serious in dogs that acquire the infection prenatally (*138*). Most common signs affect the nervous and musculoskeletal system. Disease often begins with incoordination/paresis of the hindlimbs which may progress to the forelimbs (*138*). Other signs may include difficulty swallowing, megaesophagus, muscle wasting and incontinence (*138*). In older dogs, disease may manifest as encephalomyelitis, focal cutaneous nodules/ulcers, pneumonia, peritonitis, hepatitis or myocarditis (*139*). Dogs remain infected for life but only pass oocysts for 3 weeks to a few months after initial infection (*138*,*140*). Treatment with long term clindamycin reduces symptoms. No cure is available (*138*).	N/A	No evidence of the parasite or its DNA in humans, despite serologic detection of human exposure (*138*).<br><br>*Neospora caninum* does not cause disease in people (*140*).	
Oncherocerca lupi (Onchocerciasis) UNCERTAINTY = Low	Parasite	Bite from an infected black fly (*Simuliium* spp.) or biting midge (*Culicoides* spp) (*141*).	Most infected dogs are asymptomatic (*141*). Infections usually involve the eyes (*142*). Acute ocular signs include: redness, conjunctivitis, mild to severe periorbital swelling, exophthalmia, blepharitis, photophobia, lacrimation, serous or	Bite from an infected black fly (*Simuliium* spp.) or biting midge (*Culicoides* spp) (*141*).	Zoonotic infections have been reported, though rarely (*143*). Infections have involved the eyes, joints on limbs and upper cervical spine (*141*).	

HAZARD	CATEGORY	SPREAD SCENARIO CANINE	IMPACT ANIMAL	SPREAD SCENARIO HUMAN	IMPACT HUMAN	OTHER INFORMATION
			mucopurulent discharge, protrusion of the 3rd eyelid, diffuse corneal stromal edema and corneal ulcers, uveitis (*141*). Chronic signs may include subconjunctival granulomatous nodules penetrating the retrobulbar space, orbital fascia, eyelid, 3rd eyelid or sclera (*141*). Treatment involves surgical removal of worms, if possible, which may require enucleation (*141*). Granulomatous masses containing gravid females can occur in the skin of the ears, nose intrascapular region, periocular region and umbilical region (*141*). Treatment also involves melarsomine, ivermectin, topical antibiotics and systemic prednisone (*141*).		Most cases outside the USA seem to be specifically subconjunctival and localized ocular disease in adults (*143*). In the USA, cases have mostly been in children, many having neuroinvasion where masses of nematodes compressing the cervical spinal canal (*142*,*143*). Ocular onchocerciasis in adults usually presents as a single conjunctival nodule with mild redness with the vision not affected (*144*).	
Opisthorchis spp. ***- Opisthorchis felineus*** ***- Opisthorchis viverrini*** ***Clonorchis sinensis*** ***(Chinese/oriental liver fluke)*** (LIVER FLUKE) UNCERTAINTY = Low	Parasite	Ingestion of metacercariae from infected intermediate hosts (snails, fish) (*145*).	Opisthorchis infections in dogs are mostly inapparent. Longterm presence causes thickening and fibrosis of bile and pancreatic duct walls. Severe or chronic cases have been associated with cancer in the liver or pancreas (*145*). *O. viverrini and C. sinensis* are classified as Group 1 carcinogens by the International Agency for Research on Cancer (*146*). Treatment of *Opisthorchis* spp. infections in dogs may be attempted with fenbendazole or praziquantel. *C. sinensis* infections in dogs may be attempted with praziquantel. All these treatments are extra-label (*147*).	Ingestion of metacercariae from infected intermediate hosts (snails, fish) (*148*,*149*). No direct transmission from dogs to humans (*147*).	Most infections are asymptomatic. Most pathologic manifestations result from inflammation and intermittent obstruction of the biliary ducts. In mild cases, manifestations include dyspepsia, abdominal pain, diarrhea, or constipation (*148*). With infections of longer duration, the symptoms can be more severe, and hepatomegaly and malnutrition may be present. In rare cases, cholangitis, cholecystitis, and cholangiocarcinoma may develop (*148*). Treatment of choice is either Praziquantel or Albendazole (*149*). Praziquantel has a cure rate of 83%–85% (*150*).	
Paragonimus kellicotti and P. westermani (Lung flukes) UNCERTAINTY = Low	Parasite	Infection by *P. kellicotti* and *P. westermani* occur through the ingestion of raw crayfish or crabs containing encysted cercariae (*151*). OR Consumption of small animals that feed on crayfish (*152*).	Clinical signs of *P. kellicotti* infection range from asymptomatic to severe dyspnea (*152*). *P. kellicotti* and *P. westermani* cysts form primarily in the lungs. In rare cases, they have also been recorded in the brain and other viscera (*151*). Symptoms from *P. kellicotti* and *P. westermani* can include a chronic, deep intermittent cough and lethargy (*151*). Severe infections may result in bronchiectasis, hemoptysis or spontaneous pneumothorax (*152*). *P. kellicotti* and *P. westermani* treatment is achieved through fenbendazole or praziquantel (*151*,*152*).	Infection by *P. kellicotti* and *P. westermani* occur through the ingestion of raw crayfish or crabs containing encysted cercariae (*151*). *P. kellicotti* is not contagious and cannot be spread from human to human (*153*).	Although humans can become infected by *P. kellicotti*, the incidence is very low (*152*). Initial symptoms from *Paragonimus spp.* may present as diarrhea and abdominal pain in the first 2–15 d (*153*). Afterwards the individual may experience fever, chest pain and fatigue (*153*). Chronic *Paragonimus spp.* infections may have a dry cough that develops into a productive cough, with red tinged sputum on exertion (*153*). *Paragonimus spp.* infections can be misdiagnosed as tuberculosis (*153*).	

HAZARD	CATEGORY	SPREAD SCENARIO CANINE	IMPACT ANIMAL	SPREAD SCENARIO HUMAN	IMPACT HUMAN	OTHER INFORMATION
Physaloptera spp.<br>UNCERTAINTY = Moderate	Parasite	Ingestion of an intermediate or paratenic host containing the parasite<br>*OR*<br>Ingestion of fecal matter contaminated with parasite eggs (*154*).	Animals are usually asymptomatic. Gastric ulceration and hemorrhage can occur from parasite attachment sites on the mucosa. Chronic intermittent vomiting may also occur (*155*).<br>When parasitic burden is high, dogs may experience vomiting, anorexia, weight loss, bloody stools and catarrhal gastritis (*155*).<br>No standardized treatment protocol. Infections in dogs can be treated with repeated doses of anthelmintics and reinfection is common (*155*,*156*). Removal of adult nematodes can also be done through endoscopy but anthelmintic treatments should still follow (*156*).	*Physaloptera* species in dogs and cats are NOT zoonotic (*156*).	N/A	
Sarcocystis spp.<br>*S. cruzi*<br>*S. capracanis*<br>*S. hircicanis*<br>*S. meisheriana*<br>*S. fayeri*<br>*S. tenella*<br>(Sarcocytosis)<br>UNCERTAINTY = Low	Parasite	Ingestion of undercooked beef or pork products containing sporocysts (*157*).<br>OR<br>Ingesting feces from another animal that ate food infected with sporocysts (*157*).	Infected dogs rarely display clinical signs (*157*); mild diarrhea may occur if parasites are in very high numbers (*157*,*158*).<br>Clinical signs can develop if sarcocysts develop in particular areas, such as close to the central nervous system (*158*).<br>Occasionally DHs like the dog can also serve as IHs and develop sarcocysts (*158*).	Ingestion of uncooked meat infected with sporocysts (definitive hosts) (*159*).<br>OR<br>Ingestion of infective oocysts from non-human sarcocystis species (intermediate host) (*159*).<br>Person to person transmission does NOT occur (*159*).	Humans are accidental dead-end hosts for non-human *Sarcocystis* species (*159*).<br>Clinical signs may include nausea, abdominal pain and diarrhea lasting up to 48 h. Muscle sarcocystosis, which is rare, can cause inflammation and soreness in (*157*).	
Sarcoptes scabei var. canis<br>UNCERTAINTY = Low	Parasite	Direct contact with an infected host (*160*).<br>OR<br>Direct contact with contaminated environments (e.g., Kennel, grooming facility, dog park) (*160*).	Clinical signs of sarcoptic mange may develop anytime from 10 d to 8 weeks after contact with an infested animal (*161*).<br>Intense pruritus, lesions consisting of papulocrustous eruptions with thick, yellow crusts, excoriation, erythema, alopecia. Secondary bacterial and yeast infections may develop. Asymptomatic carriers may exist (*161*).	Zoonotic cases of *S. scabiei* var. *canis* infestation are only a concern in individuals that have direct contact with infested dogs or wild canids. Once acquired by a person, *S. scabiei* var. *canis* mites do not transfer to other humans (*162*).	Human infestations are generally considered self-limiting (*162*).<br>Intensely pruritic rash usually develops within 1 to 4 d after contact with an infested dog. Lasts several weeks (*162*).<br>May be managed symptomatically with topical hydrocortisone creams or, if severe, treated systemically (*162*).	
Spirocerca lupi<br>***UNCERTAINTY = Low***	Parasite	Ingestion of an infected intermediate host (dung beetle) or a paratenic host (e.g., chickens, reptiles, rabbits, or rodents) (*163*).	Most dogs show no clinical signs. However, when signs are present, they include vomiting, weight loss, coughing. May include difficulty swallowing and vomiting if esophageal lesions are very large (*163*).<br>In severe cases, *S. lupi* can cause aneurysm of the thoracic aorta and esophageal nodules.<br>About 25% of esophageal nodules undergo neoplastic transformation, typically to malignant sarcoma (*163*).<br>Treatment:	Not known to infect humans (*154*).	N/A	

HAZARD	CATEGORY	SPREAD SCENARIO CANINE	IMPACT ANIMAL	SPREAD SCENARIO HUMAN	IMPACT HUMAN	OTHER INFORMATION
			-Doramectin<br>-Monitor efficacy with endoscopy and/or radiology<br>-Excision of nonmetastatic neoplasms (*163*).<br>Diagnosis in the early stages can be challenging. Most animals are only diagnosed in advanced stages of the disease (*164*).			
Strongyloides stercoralis<br>(Strongyloidosis)<br>UNCERTAINTY = Low	Parasite	Rhabditiform larvae penetrates skin (transcutaneous)<br>OR<br>Rhabditiform larvae in soil are ingested (oral mucosa penetration)<br>OR<br>Transmammary transmission (*154*,*165*).	Disease ranges from asymptomatic to severe clinical signs (*166*).<br>Clinical symptoms appear most often in immunocompromised or juvenile dogs (*167*).<br>Clinical symptoms in dogs may include skin lesions, coughing and intestinal symptoms such as bloody diarrhea (*165*,*167*).<br>Emaciation and decreased growth rate may be evident. Appetite is often unchanged in early stages of infection (*165*).<br>Infected puppies infected via transmammary transmission can shed eggs within 7–10 d post infection (*165*).<br>Dogs <1 y have a greater likelihood of infection than dogs >1 y (*168*).<br>No labeled treatment exists for dogs in Canada – macrocytic lactones can have high efficacy (*167*). Treatment usually involves: ivermectin or fenbendazole with supportive care (*165*).<br>The dog specific strain does not appear to cause autoinfection under normal conditions. If infected by the human strain, autoinfection can occur but reverts back to normal infection after a few generations (*169*).	Rhabditiform larvae penetrates skin (transcutaneous).<br>OR<br>Rhabditiform larvae in soil are ingested (oral mucosa penetration) (*147*,*154*).	Acute infection can include red/pruritic rash on skin at entry site, followed by tracheal irritation and a dry cough as larvae migrate up from the lungs.<br>Gastrointestinal symptoms such as diarrhea, constipation, abdominal pain and anorexia can be seen (*170*).<br>Chronic infection is often asymptomatic with occasional gastrointestinal, pulmonary or cutaneous symptoms (*170*).<br>Rarely individuals with chronic infections can develop medical complications such as arthritis, cardiac arrhythmias, chronic malabsorption, central nervous system issues, duodenal obstruction, nephrotic syndrome and recurrent asthma (*170*).<br>Impaired immunity leads to accelerated autoinfection (*170*).<br>Subcutaneous migration from autoinfection can result in a rash along the buttock, perineum and thighs (*170*).<br>Hyperinfection can occur in those taking immunosuppressive drugs or who have impaired cell-mediated immunity (*149*).<br>In hyperinfection syndrome and chronic strongyloidiasis, the parasite is contained to the small intestine and lungs. In disseminated strongyloidiasis, larvae disperse into various organs and can result in severe complications (a) like bacteremia and meningitis (*170*).<br>If untreated, hyperinfection and disseminated strongyloidiasis has a mortality rate of 90% (*170*).<br>Treatment involves with an antiparasitic such as Ivermectin or	Two genetic populations have been identified in dogs – one that only infects dogs and another that can infect both dogs and humans (*170*).<br>The species found most commonly in dogs is the same that infects humans (*171*).<br>Transmission of parasite between dogs and humans is possible (*167*).<br>Can cause significant disease in immunocompromised individuals (*172*).<br>There have been reports of humans infected by dogs and vice versa (*169*).<br>In a systematic review and metanalysis, the prevalence of dog owners infected was 7% (*168*).

HAZARD	CATEGORY	SPREAD SCENARIO CANINE	IMPACT ANIMAL	SPREAD SCENARIO HUMAN	IMPACT HUMAN	OTHER INFORMATION
					Albendazole – treatment length varies depending on severity of infection (*170*).	
Taenia spp. ***T. hydatigena*** ***T. taeniaeformis*** ***T. multiceps*** ***T. crassiceps*** ***T. serialis*** ***T. pisiformis*** (Taeniasis) UNCERTAINTY = Low	Parasite	Ingestion of larval cysts while preying on or scavenging infected vertebrates (*173*).	-Disease in dogs due to infection with adult *Taenia* species is rare (*173*). -Passage of proglottids may be associated with perianal irritation. Can occasionally cause intestinal impactions (*173*). -Tapeworm medications are highly effective at eliminating *Taenia spp*. Praziquantel, Epsiprantel, Febendazole are approved for treatment in dogs and cats (*173*,*174*).	-Adults of *Taenia pisiformis* are known to infect ONLY dogs and wild canids (*173*). -Isolated reports of zoonotic infection with larval *Taenia spp*. of dogs and cats exist. However, the overall risk of human infection with *Taenia spp*. in North America appears EXTREMELY low (*173*). Other *Taenia* species of significant public health concern include *T. saginata* and *T. solium*, but they are NOT parasites of domestic cats and dogs (*173*).	N/A	
Toxocara canis (Toxocariasis) UNCERTAINTY = Low	Parasite	Fecal-oral contamination - ingestion of embryonated eggs from the environment (*175*,*176*). OR Ingestion of infected paratenic host (*175*). OR Vertical transmission from infected pregnant bitch to developing fetus(es) (*177*). OR Transmammary transmission to puppies via nursing (*178*).	*Toxocara* infections worldwide was higher in dogs ≤12 mo (28.7%) compared to older dogs (12.9%) (*176*). Intestinal infections are usually asymptomatic in older dogs (Radman et al., 2018). Young dogs are usually symptomatic (*178*). Clinical signs in puppies include the characteristic 'pot-bellied' appearance, dull coat, lack of growth and loss of condition (*178*,*179*). Diarrhea with mucus, vomiting, constipation, and flatulence may also be present (*178*,*179*). Chronic intestinal infections may result in intestinal wall thickening and intussusception (*179*). Large numbers of intestinal *T. canis* nematodes may uncommonly result in obstruction of the gall bladder, bile duct, or pancreatic duct gastrointestinal perforation, or peritonitis (*179*). In the early stages, larvae migrating through the lungs may lead to inflammation and respiratory signs, such as eosinophilic pneumonia (*178*,*179*). Puppies infected *in utero* can develop pneumonia resulting in death days after birth.	Ingestion of embryonated eggs from the environment OR Ingestion of raw/undercooked paratenic hosts containing infective L3 larvae (*176*).	Humans are accidental hosts of *T. canis* (*176*). Most people with *T. canis* infections do not have symptoms (*176*). In clinical cases, symptoms relate to the site of the migrating larvae, with the most sites being the liver and lungs (visceral larval migrans) and the eyes (ocular larval migrans) (*180*). Most cases of VLM are subclinical, but in more severe cases, signs can include malaise, fever, enlarged liver, and upper abdominal discomfort (*179*). Other symptoms may include nausea, vomiting and respiratory signs such as coughing, wheezing and dyspnea (*176*). VLM is most common in children aged 1–7 (*180*). OLM is most common in children >8 y and adults, resulting in retinal granulomas (*179*,*180*). Other clinical signs from OLM include endophthalmitis, neuritis, cataracts, conjunctivitis, uveitis and optic neuritis. In the worst cases, OLM can result in irreversible blindness (*178*,*179*). High parasite burden has also been associated with *T. canis* larvae migrating to other organs and resulting	

HAZARD	CATEGORY	SPREAD SCENARIO CANINE	IMPACT ANIMAL	SPREAD SCENARIO HUMAN	IMPACT HUMAN	OTHER INFORMATION
			Severe infections in young dogs may also result in verminous pneumonia, ascites, and fatty liver degeneration (*178*). Myocarditis and cortical kidney granulomas containing larvae have also been reported (*178*,*179*). Treatment involves anthelmintics (*178*).		in severe pneumonia, cardiac involvement and central nervous system disease. These cases are rare but often result in death (*179*). Treatment of VLM is accomplished with antiparasitic drugs. OLM is more difficult and involves of measures to prevent progressive eye damage (*176*).	
Trichuris vulpis<br>UNCERTAINTY = Low	Parasite	Ingestion of eggs containing infective larvae from the environment (e.g., from soil).<br>Fecal oral route<br>Eggs can remain in environment for several years (*181*).	No clinical signs are found in light infections. As worm burden increases in severe infections, may see weight loss, diarrhea (fresh blood may be present), and anemia (*182*).<br>Treatment:<br>Multiple anthelmintics are available for treatment (Drontal Plus, Panacur, Advantage Multi, Coraxis, Inceptor...etc) (*181*).<br>Prognosis excellent in most cases, though severely infected animals may require more intensive therapy (*181*).	No sufficient evidence of zoonotic risk to humans (*181*).	N/A	
Trypanosoma cruzi<br>(Chagas disease/American trypanosomiasis)<br>UNCERTAINTY = Low	Parasite	The stercorarian (vector-fecal) route: triatomine bug excretes *T. cruzi* in its feces onto the host. *T. cruzi* enters through the bite wound, a break in the skin, or a mucous membrane (*183*).<br>OR<br>Transplacental and trans mammary from infected mother to offspring (*183*).<br>OR<br>Blood transfusion (*183*)<br>OR<br>Ingestion of an infected animal or insect (*184*).	Severe and rapid, in young dogs (<6 mo). Can include weakness, not eating and sudden death due to heart disease (*185*).<br>Slowly progressive (chronic) in adult dogs. Most infected dogs never develop any symptoms, while others may progress to chronic infection and develop heart disease later in life (*185*).<br>Currently no cure (parasiticide) for infected dogs (*185*).<br>Dogs are usually infected for life. Therapy is only supportive, used to reduce the signs of heart disease and slow progression to heart failure (*185*).	Contact with the feces of an infected triatomine bug (most common) (*186*).<br>OR<br>Mother-to-baby (congenital) (*186*).<br>OR<br>Contaminated blood products (transfusions) (*186*).<br>OR<br>An organ transplanted from an infected donor (*186*).<br>OR<br>Laboratory accident (rare) (*186*).	Most infected individuals (70%) remain asymptomatic for life (*187*).<br>However, over a period of 10–30 y, 20%–35% of patients develop symptomatic chronic Chagas disease, characterized by cardiac and/or gastrointestinal disorders (*186*,*188*).<br>Treatment: benznidazole or nifurtimox. Both medications kill the parasite and are fully effective in curing the disease if given early in the acute phase. Their efficacy diminishes the longer a person has been infected (*189*).<br>There is no vaccine to prevent Chagas disease (*189*).	
Trypanosoma congolese, T brucei brucei<br>(UNCERTAINTY = Low)	Parasite	Bitten by an infected tsetse fly (*190*,*191*).<br>No direct dog-to-dog transmission (*190*,*191*).	Clinical signs include persistent fever, lethargy, anorexia, weight loss, pallor, mucopurulent oculonasal discharge, lymphadenopathy, hepatosplenomegaly, variable peripheral edema, abdominal distention due to ascites, petechial hemorrhages, signs of pancarditis, and ocular signs such as unilateral or bilateral uveitis, corneal edema, and/ or keratitis (*190*).	Neither *T. congolese* nor *T. brucei brucei* infects humans (*190*,*191*).	N/A	

HAZARD	CATEGORY	SPREAD SCENARIO CANINE	IMPACT ANIMAL	SPREAD SCENARIO HUMAN	IMPACT HUMAN	OTHER INFORMATION
			-Hemorrhagic vomiting and diarrhea and neurologic signs (seizures, tremors, opisthotonos, and hyperreflexia) have also been described in dogs infected with *T. congolense* (*190*).<br>Treatment:<br>There has been development of several compounds with efficacy against African canine trypanosomiasis, however none of these products have been produced in a large commercial scale or even available in the market. The unavailability of new trypanocides in the market are a challenge for treatment. Diminazene aceturate has shown efficacy when used to treat *T. congolense and T. brucei brucei* infection (*192*).			
**Canine adenovirus type 1**<br>(Infectious canine hepatitis)<br>UNCERTAINTY = Low	Virus	Transmission occurs via direct contact with saliva, urine, or feces of infected dog (*193*,*194*).	Three types of infection have been documented: uncomplicated, acute, and peracute (*195*).<br>Uncomplicated presents as transient fever, vomiting, diarrhea, lethargy, abdominal pain, tonsillitis, and corneal edema (*190*,*194*).<br>Acute presents similarly as uncomplicated, but can last much longer and result in death (*194*).<br>Peracute is due to chronic infection. Death can occur due to hepatic failure (*194*).<br>Mortality ranges from 10% to 30%. Death is most common in young dogs (*193*,*194*).	Not zoonotic.	N/A	
**Canine distemper virus**<br>(Canine distemper)<br>UNCERTAINTY = Low	Virus	Shed in all bodily secretions from infected dogs, with direct oronasal contact the most likely. Highly contagious (R_o = 1.26) but does not persist in the environment for long (<24 h) (*196*,*197*).<br>Thrives in crowded, closed-air environments (*197*).	Highly dependent on strain as well as age and immune status of the dog. ~50% of cases are associated with rapidly progressive, multi-system fatal disease (up to ~80% in puppies) (*197*).	Not zoonotic.<br>Controversy exists with role of CDV in Paget's disease of bone (*197*).	N/A	
**Canine herpes Virus-1**<br>(Canine herpes)<br>UNCERTAINTY = Low	Virus	Transmission occurs via direct contact with respiratory and /or genital secretions (*198*,*199*).<br>Does not survive well in the environment (*198*,*199*).	Ranges from subclinical to severe and fatal disease depending on age and immune status (*199*).<br>Healthy adult dogs may present with mild and transient upper respiratory disease, including ocular and nasal discharge, sneezing, and/or ocular disease (keratitis, conjunctivitis) (*199*).<br>Intact adult dogs, may have visible, non-painful lesions on genital tract, as well as abortions and stillbirths (*200*).	Not zoonotic.	N/A	

HAZARD	CATEGORY	SPREAD SCENARIO CANINE	IMPACT ANIMAL	SPREAD SCENARIO HUMAN	IMPACT HUMAN	OTHER INFORMATION
			Neonatal puppies experience acute and often fatal systemic disease, especially if dam was naive and did not pass on maternal antibodies (*199*,*201*).			
***Canis familiaris* papilloma virus**<br>Types 1 and 6 (plus others, at least 23 types have been identified) (*202*).<br>(Viral papilloma)<br>UNCERTAINTY = Moderate	Virus	Transmission occurs via direct contact with an infected dog, usually through micro abrasions in the skin or mucous membranes (*203*).	Several forms exist, depending on the virus type, age, and breed of the dog. The most common are oral or cutaneous papillomas or pigmented plaques, which typically regress on their own. In rare cases, viral infection can lead to squamous cell carcinoma (*202*).	Not zoonotic.	N/A	
**Canine parvovirus**<br>(Parvoviral enteritis)<br>UNCERTAINTY = Low	Virus	Direct contact with feces or vomitus from infected animal (*204*).<br>Virus can persist in the environment for months, making indirect transmission likely (*204*).	Presentation can var.y based on age of animal, immune status, concurrent infections or stressors (*204*).<br>Severe enteritis is common, particularly in puppies, which is characterized by fever, lethargy anorexia, vomiting, diarrhea, dehydration, and possibly secondary bacterial infections. Mortality can reach 90% (*204*,*205*).<br>Those infected in utero and up to two weeks of age can develop myocarditis leading to congestive heart failure and death (*206*,*207*).	Not zoonotic.	N/A	
**Influenza A H3n8 and h3n2**<br>(Canine influenza)<br>UNCERTAINTY = Moderate	Virus	H3N8: Transmission (albeit inefficient with R_O~1) via direct contact with respiratory secretions or indirect contact with contaminated fomites (*208*).<br>H3N2: Transmission via direct contact with respiratory secretions or indirect contact with contaminated fomites.<br>R_O estimated between 1 and 1.5 (*208*).<br>Closed, high density environments such as kennels and boarding facilities support more efficient transmission (*209*).	Mild, self-limiting upper respiratory infection most common which is characterized by soft cough, purulent nasal and ocular discharge and a low-grade fever (*210*).<br>Severe lower respiratory tract infection can occur in ~1%–5% of cases, which is characterized by high fever, purulent nasal and ocular discharge, difficulty breathing, loss of appetite, and depression (*211*).<br>Death has been reported in rare instances due to hemorrhagic pneumonia (*212*).<br>Young, old, and brachycephalic breeds are at highest risk (*213*).	Not zoonotic.<br>However, since influenzas are notorious for recombination, potential cannot be discounted (*213*).	N/A	

References

1. Diniz PPVP, Chomel BB, Guptill L, Breitschwerdt EB. Bartonellosis. In: Sykes JE, editor. Greene's infectious diseases of the dog and cat, 5th edition. St. Louis, MO: Elsevier; 2023. p. 853–75.

2. Chomel BB, Boulouis HJ, Maruyama S, Breitschwerdt EB. *Bartonella* spp. in pets and effect on human health. Emerg Infect Dis. 2006;12:389–94. PubMed https://doi.org/10.3201/eid1203.050931

3. Sykes JE, Chomel BB. Bartonellosis. In: Sykes JE, editor. Canine and feline infectious diseases, 1st edition. St. Louis, MO: Elsevier; 2014. p. 498–511.

4. Centers for Disease Control and Prevention. Veterinary guidance for bartonellosis [cited 2025 Mar 8]. https://www.cdc.gov/bartonella/hcp/veterinarians/index.html

5. Álvarez-Fernández A, Breitschwerdt EB, Solano-Gallego L. *Bartonella* infections in cats and dogs including zoonotic aspects. Parasit Vectors. 2018;11:624. PubMed https://doi.org/10.1186/s13071-018-3152-6

6. Breitschwerdt EB, Maggi RG, Lantos PM, Woods CW, Hegarty BC, Bradley JM. *Bartonella vinsonii* subsp. *berkhoffii* and *Bartonella henselae* bacteremia in a father and daughter with neurological disease. Parasit Vectors. 2010;3:29. PubMed https://doi.org/10.1186/1756-3305-3-29

7. Bai Y, Gilbert A, Fox K, Osikowicz L, Kosoy M. *Bartonella rochalimae* and *B. vinsonii* subsp. *berkhoffii* in wild carnivores from Colorado, USA. J Wildl Dis. 2016;52:844–9. PubMed https://doi.org/10.7589/2016-01-015

8. Sykes JE. Bordetellosis. In: Sykes JE, editor. Canine and feline infectious diseases, 1st edition. St. Louis, MO: Elsevier; 2014. p. 372–9.

9. Datz C. *Bordetella* infections in dogs and cats: treatment and prevention [cited 2025 Feb 17]. https://www.vetfolio.com/learn/article/bordetella-infections-in-dogs-and-cats-treatment-and-prevention

10. Spickler AR, The Center for Food Security and Public Health. Brucellosis [cited 2025 Feb 17]. https://www.cfsph.iastate.edu/Factsheets/pdfs/brucellosis.pdf

11. The Center for Food Security & Public Health. Canine brucellosis: brucella canis [cited 2025 Feb 17]. https://www.cfsph.iastate.edu/FastFacts/pdfs/canine_brucellosis_F.pdf

12. Kauffman LK, Petersen CA. Canine brucellosis. Vet Clin North Am Small Anim Pract. 2019;49:763–79. PubMed https://doi.org/10.1016/j.cvsm.2019.02.013

13. Sykes J, Davidson A. Canine brucellosis. In: Sykes JE, editor. Canine and feline infectious diseases, 1st edition. St. Louis, MO: Elsevier; 2014. p. 512–9.

14. Santos RL, Souza TD, Mol JPS, Eckstein C, Paíxão TA. Canine brucellosis: an update. Front Vet Sci. 2021;8:594291. PubMed https://doi.org/10.3389/fvets.2021.594291

15. O'Brien CR, Sykes JE. Miscellaneous bacterial infections. In: Sykes JE, editor. Greene's infectious diseases of the dog and cat, 5th edition. St. Louis, MO: Elsevier; 2023. p. 948–59.

16. Sprague LD. Merck Manual Veterinary Manual. Melioidosis in animals [cited 2025 Feb 20]. https://www.merckvetmanual.com/infectious-diseases/melioidosis/melioidosis-in-animals

17. Acke E. Campylobacteriosis. In: Sykes JE, editor. Greene's infectious diseases of the dog and cat, 5th edition. St. Louis, MO: Elsevier; 2023. p. 774–84.

18. Whiley H, van den Akker B, Giglio S, Bentham R. The role of environmental reservoirs in human campylobacteriosis. Int J Environ Res Public Health. 2013;10:5886–907. PubMed https://doi.org/10.3390/ijerph10115886

19. Janssen R, Krogfelt KA, Cawthraw SA, van Pelt W, Wagenaar JA, Owen RJ. Host-pathogen interactions in *Campylobacter* infections: the host perspective. Clin Microbiol Rev. 2008;21:505–18. PubMed https://doi.org/10.1128/CMR.00055-07

20. Lion C, Escande F, Burdin JC. *Capnocytophaga canimorsus* infections in human: review of the literature and cases report. Eur J Epidemiol. 1996;12:521–33. PubMed https://doi.org/10.1007/BF00144007

21. Gaastra W, Lipman LJA. *Capnocytophaga canimorsus*. Vet Microbiol. 2010;140:339–46. PubMed https://doi.org/10.1016/j.vetmic.2009.01.040

22. Meyers B, Schoeman JP, Goddard A, Picard J. The bacteriology and antimicrobial susceptibility of infected and non-infected dog bite wounds: fifty cases. Vet Microbiol. 2008;127:360–8. PubMed https://doi.org/10.1016/j.vetmic.2007.09.004

23. Centers for Disease Control and Prevention. About Capnocytophaga [cited 2025 Mar 14]. https://www.cdc.gov/capnocytophaga/about/index.html

24. Harrus S, Waner T, Mylonakis ME, Sykes JE, Qurollo B. Ehrlichiosis. In: Sykes JE, editor. Greene's infectious diseases of the dog and cat, 5th edition. St. Louis, MO: Elsevier; 2023. p. 522–41.

25. Higgs V. Ehrlichiosis in dogs [cited 2025 Mar 14]. https://www.petmd.com/dog/conditions/infectious-parasitic/c_dg_ehrlichiosis

26. Public Health Agency of Canada. Ticks in Canada [cited 2025 Mar 14]. https://www.canada.ca/en/public-health/services/diseases/ticks-tick-borne-diseases/ticks.html

27. Starkey LA, Barrett AW, Beall MJ, Chandrashekar R, Thatcher B, Tyrrell P, et al. Persistent *Ehrlichia ewingii* infection in dogs after natural tick infestation. J Vet Intern Med. 2015;29:552–5. PubMed https://doi.org/10.1111/jvim.12567

28. Foley JE. Merck Manual Veterinary Manual. Ehrlichiosis, Anaplasmosis, and related infections in animals [cited 2025 Mar 19]. https://www.merckvetmanual.com/infectious-diseases/rickettsial-diseases/ehrlichiosis-anaplasmosis-and-related-infections-in-animals

29. McQuiston JH, McCall CL, Nicholson WL. Ehrlichiosis and related infections. J Am Vet Med Assoc. 2003;223:1750–6. **PMID 14690204**

30. Buller RS, Arens M, Hmiel SP, Paddock CD, Sumner JW, Rikhisa Y, et al. *Ehrlichia ewingii*, a newly recognized agent of human ehrlichiosis. N Engl J Med. 1999;341:148–55. PubMed https://doi.org/10.1056/NEJM199907153410303

31. Government of Canada. A handbook to the ticks of Canada [cited 2025 Mar 14]. https://profils-profiles.science.gc.ca/en/publication/handbook-ticks-canada

32. Cornell Wildlife Health Lab. Leptospirosis [cited 2025 Mar 19]. https://cwhl.vet.cornell.edu/disease/leptospirosis

33. Cohen A, Cornell University College of Veterinary Medicine. Canine leptospirosis [cited 2025 Mar 19]. https://www.vet.cornell.edu/departments-centers-and-institutes/riney-canine-health-center/canine-health-information/canine-leptospirosis

34. American Veterinary Medical Association. Leptospirosis in dogs [cited 2025 Mar 19]. https://www.avma.org/resources-tools/pet-owners/petcare/leptospirosis

35. Lunn KF. Merck Manual Veterinary Manual. Leptospirosis in dogs. 2018. [cited 2025 Mar 25]. https://www.merckvetmanual.com/dog-owners/disorders-affecting-multiple-body-systems-of-dogs/leptospirosis-in-dogs

36. Lunn KF. Merck Manual Veterinary Manual. 2022. Leptospirosis in dogs [cited 2025 Mar 19]. https://www.merckvetmanual.com/infectious-diseases/leptospirosis/leptospirosis-in-dogs

37. American Veterinary Medical Association. Leptospirosis in dogs & cats [cited 2025 Mar 19]. https://ebusiness.avma.org/files/productdownloads/LR_COM_ClientBroch_Leptospirosis_022616.pdf

38. Centers for Disease Control and Prevention. About leptospirosis [cited 2025 Feb 13]. https://www.cdc.gov/leptospirosis/about/index.html

39. World Health Organization. Leptospirosis: fact sheet [cited 2025 Feb 13]. https://www.who.int/publications/i/item/B4221

40. Barker EN, Tasker S. Hemotropic mycoplasma infections. In: Sykes JE, editor. Greene's infectious diseases of the dog and cat, 5th edition. St. Louis, MO: Elsevier; 2023. p. 690–703.

41. Willi B, Novacco M, Meli M, Wolf-Jäckel G, Boretti F, Wengi N, et al. Haemotropic mycoplasmas of cats and dogs: transmission, diagnosis, prevalence and importance in Europe. Schweiz Arch Tierheilkd. 2010;152:237–44. PubMed https://doi.org/10.1024/0036-7281/a000055

42. Tasker S. Hemotropic mycoplasma. Vet Clin North Am Small Anim Pract. 2022;52:1319–40. PubMed https://doi.org/10.1016/j.cvsm.2022.06.010

43. Foley JE. Merck Manual Veterinary Manual. 2022. Hemotropic mycoplasma infections in animals [cited 2024 Sep 17]. https://www.merckvetmanual.com/circulatory-system/blood-parasites/hemotropic-mycoplasma-infections-in-animals

44. Kidd L, Breitschwerdt EB. Spotted fever rickettsioses, flea-borne rickettsioses, and typhus. In: Sykes JE, editor. Greene's infectious diseases of the dog and cat, 5th edition. St. Louis, MO: Elsevier; 2023. p. 555–70.

45. Spickler AR, The Center for Food Security and Public Health. Spotted fevers including rocky mountain spotted fever and mediterranean spotted fever [cited 2025 Mar 19]. https://www.cfsph.iastate.edu/Factsheets/pdfs/RMSF.pdf

46. Solano-Gallego L, Kidd L, Trotta M, Di Marco M, Caldin M, Furlanello T, et al. Febrile illness associated with *Rickettsia conorii* infection in dogs from Sicily. Emerg Infect Dis. 2006;12:1985–8. PubMed https://doi.org/10.3201/eid1212.060326

47. Colomba C, Saporito L, Polara VF, Rubino R, Titone L. Mediterranean spotted fever: clinical and laboratory characteristics of 415 Sicilian children. BMC Infect Dis. 2006;6:60. PubMed https://doi.org/10.1186/1471-2334-6-60

48. Sykes JE, Outerbridge C. Dermatophytosis. In: Sykes JE, editor. Canine and feline infectious diseases, 1st edition. St. Louis, MO: Elsevier; 2014. p. 558–69.

49. Merchant SR. Merck Manual Veterinary Manual. 2018. Ringworm (dermatophytosis) in dogs [cited 2025 Mar 25]. https://www.merckvetmanual.com/dog-owners/skin-disorders-of-dogs/ringworm-dermatophytosis-in-dogs

50. Moriello KA. Merck Manual Veterinary Manual. 2025. Dermatophytosis in dogs and cats [cited 2025 Mar 20]. https://www.merckvetmanual.com/integumentary-system/dermatophytosis/dermatophytosis-in-dogs-and-cats

51. Centers for Disease Control and Prevention. What causes ringworm [cited 2025 Mar 22]. https://www.cdc.gov/ringworm/causes/index.html

52. Spickler AR, The Center for Food Security & Public Health. Dermatophytosis [cited 2025 Mar 19]. https://www.cfsph.iastate.edu/Factsheets/pdfs/dermatophytosis.pdf

53. Public Health Agency of Canada. Pathogen safety data sheets: infectious substances—*Epidermophyton floccosum*, *Microsporum* spp., *Trichophyton* spp. [cited 2025 Mar 22]. https://www.canada.ca/en/public-health/services/laboratory-biosafety-biosecurity/pathogen-safety-data-sheets-risk-assessment/epidermophyton-floccosum-microsporum-trichophyton.html

54. Centers for Disease Control and Prevention. Treatment of ringworm [cited 2025 Mar 22]. https://www.cdc.gov/ringworm/treatment/index.html

55. University of Cambridge. Canine transmissible venereal tumour (CTVT) [cited 2025 Mar 22]. https://www.tcg.vet.cam.ac.uk/about/ctvt

56. Ganguly B. Merck Manual Veterinary Manual. 2024. Canine transmissible venereal tumor [cited 2025 Mar 22]. https://www.merckvetmanual.com/reproductive-system/canine-transmissible-venereal-tumor/canine-transmissible-venereal-tumor

57. Abeka YT. Review on Canine Transmissible Venereal Tumor (CTVT). Canc Therapy & Oncol Int J. 2019;14:555895.

58. Williams K, Stoewen D, Pinard C. Transmissible venereal tumor [cited 2025 Mar 22]. https://vcacanada.com/know-your-pet/transmissible-venereal-tumor

59. Mello Martins MI, Ferreira de Souza F, Gobello C. The canine transmissible venereal tumor: etiology, pathology, diagnosis and treatment [cited 2025 Mar 19]. IN: Concannon PW, England G, Verstegen III J, Forsberg L, editors. Recent advances in small animal reproduction. https://www.ivis.org/library/recent-advances-small-animal-reproduction/canine-transmissible-venereal-tumor-etiology

60. Snowden KF, Ketzis JK. Trematodes. In: Sykes JE, editor. Greene's infectious diseases of the dog and cat, 5th edition. St. Louis, MO: Elsevier; 2023. p. 1528–49.

61. Western College of Veterinary Medicine. Learn about parasites: *Alaria* species [cited 2024 Jul 2]. https://wcvm.usask.ca/learnaboutparasites/parasites/alaria-species.php

62. Companion Animal Parasite Council. Alaria for dog [cited 2024 Jul 2]. https://capcvet.org/guidelines/alaria

63. Bowman DD, Lucio-Forster A, Lee ACY. Hookworms. In: Sykes JE, editor. Greene's infectious diseases of the dog and cat, 5th edition. St. Louis, MO: Elsevier; 2023. p. 1436–43.

64. Companion Animal Parasite Council. Hookworms for dog [cited 2024 Jul 2]. https://capcvet.org/guidelines/hookworms

65. Centers for Disease Control and Prevention. Hookworm (extraintestinal) [cited 2024 Jul 2]. https://www.cdc.gov/dpdx/zoonotichookworm/index.html

66. Merck Manual Consumer Version. Cutaneous larva migrans [cited 2025 Mar 22]. https://www.merckmanuals.com/en-ca/home/skin-disorders/parasitic-skin-infections/cutaneous-larva-migrans

67. Spickler AR, The Center for Food Security and Public Health. Zoonotic hookworms [cited 2025 Mar 19] https://www.cfsph.iastate.edu/Factsheets/pdfs/hookworms.pdf

68. Western College of Veterinary Medicine. Ancylostoma caninum [cited 2025 Mar 22]. https://wcvm.usask.ca/learnaboutparasites/parasites/ancylostoma-caninum.php

69. Conboy GA. Canine angiostrongylosis: the French heartworm: an emerging threat in North America. Vet Parasitol. 2011;176:382–9. PubMed https://doi.org/10.1016/j.vetpar.2011.01.025

70. Newfoundland and Labrador Department of Natural Resources. French heartworm infection of dogs and foxes in Newfoundland [cited 2025 Mar 22]. https://www.gov.nl.ca/fisheries/files/agrifoods-animals-health-pdf-ds-08-009.pdf

71. Western College of Veterinary Medicine. *Angiostrongylus vasorum* [cited 2025 Mar 22]. https://wcvm.usask.ca/learnaboutparasites/parasites/angiostrongylus-vasorum.php

72. Birkenheuer AJ. Babesiosis. In: Sykes JE, editor. Greene's infectious diseases of the dog and cat, 5th edition. St. Louis, MO: Elsevier; 2023. p. 1203–17.

73. Companion Animal Parasite Council. *Babesia* spp. [cited 2024 Jul 2]. https://capcvet.org/guidelines/babesia/

74. Weir M, Llera R, Ward E. Babesiosis in dogs [cited 2025 Mar 22]. https://vcacanada.com/know-your-pet/babesiosis-in-dogs

75. Pion P, Spadafori G. Babesia infection in dogs. https://www.vin.com/doc/?id=8156779

76. Brooks W. Veterinary partner cited 2025 Mar 19]. 2017. https://www.vin.com/doc/?id=8156779

77. Carter PD, Rolls P. Merck Manual Veterinary Manual. Babesiosis in animals. 2022 [cited 2025 Mar 22]. https://www.merckvetmanual.com/circulatory-system/blood-parasites/babesiosis-in-animals

78. Conboy GA, Sykes JE. Nematode infections of the respiratory tract. In: Sykes JE, editor. Greene's infectious diseases of the dog and cat, 5th edition. St. Louis, MO: Elsevier; 2023. p. 1505–27.

79. Companion Animal Parasite Council. *Crenosoma vulpis* [cited 2025 Mar 22]. https://capcvet.org/guidelines/crenosoma-vulpis/

80. Western College of Veterinary Medicine. *Eucoleus (Capillaria) aerophila* [cited 2024 Jul 2]. https://wcvm.usask.ca/learnaboutparasites/parasites/eucoleus-capillaria-aerophila.php

81. Lappin MR. Cryptosporidiosis. In: Sykes JE, editor. Canine and feline infectious diseases, 1st edition. St. Louis, MO: Elsevier; 2014. p. 785–92.

82. Western College of Veterinary Medicine. *Cryptosporidium* species [cited 2025 Mar 23]. https://wcvm.usask.ca/learnaboutparasites/parasites/cryptosporidium-species.php

83. Alonge J. Cryptosporidium in dogs [cited 2025 Mar 23]. https://www.petmd.com/dog/conditions/infectious-parasitic/cryptosporidium-dogs

84. Companion Animal Parasite Council. Cryptosporidium [cited 2025 Mar 23]. https://capcvet.org/guidelines/cryptosporidium/

85. Lucio-Forster A, Griffiths JK, Cama VA, Xiao L, Bowman DD. Minimal zoonotic risk of cryptosporidiosis from pet dogs and cats. Trends Parasitol. 2010;26:174–9. PubMed https://doi.org/10.1016/j.pt.2010.01.004

86. Ryan U, Fayer R, Xiao L. Cryptosporidium species in humans and animals: current understanding and research needs. Parasitology. 2014;141:1667–85. PubMed https://doi.org/10.1017/S0031182014001085

87. Xiao L, Cama VA, Cabrera L, Ortega Y, Pearson J, Gilman RH. Possible transmission of Cryptosporidium canis among children and a dog in a household. J Clin Microbiol. 2007;45:2014–6. PubMed https://doi.org/10.1128/JCM.00503-07

88. Brown SA. Merck Manual Veterinary Manual. 2015. Giant kidney worm infection in mink and dogs [cited 2025 Mar 24]. https://www.merckvetmanual.com/urinary-system/infectious-diseases-of-the-urinary-system-in-small-animals/giant-kidney-worm-infection-in-mink-and-dogs

89. Centers for Disease Control and Prevention. Dioctophymiasis [cited 2025 Mar 24]. https://www.cdc.gov/dpdx/dioctophymiasis/index.html

90. Companion Animal Parasite Council. Urinary tract nematodes [cited 2025 Mar 24]. https://capcvet.org/guidelines/urinary-tract-nematodes/

91. Western College of Veterinary Medicine. *Dioctophyma renale* [cited 2024 Jul 3]. https://wcvm.usask.ca/learnaboutparasites/parasites/dioctophyma-renale.php

92. Weese S. Giant kidney worm in dogs [cited 2024 Jul 3]. https://www.wormsandgermsblog.com/2022/03/articles/animals/dogs/giant-kidney-worm-in-dogs

93. Park HY, Seo JW, Lee BH, Lee JY, Kim SY, Cha SJ, et al. Simultaneous occurrence of malignant fibrous histiocytoma of the ureter and *dioctophyma renale* infection: a case report. Taehan Yongsang Uihakhoe Chi . 2013;68:411. https://doi.org/10.3348/jksr.2013.68.5.411

94. Russo ZH, Callirgos JC, García-Ayachi A, Wetzel EJ. Review of *Dioctophyme renale*. J Parasitol. 2022;108:180–91. PubMed https://doi.org/10.1645/21-65

95. O'Quin J. *Dipylidium caninum*: a potential zoonosis easily missed on routine fecal diagnostics [cited 2024 Jul 3]. https://www.cliniciansbrief.com/article/dipylidium-caninum-potential-zoonosis-easily-missed-routine-fecal-diagnostics

96. Centers for Disease Control and Prevention. *Dipylidium caninum* [cited 2025 Mar 24]. https://www.cdc.gov/dpdx/dipylidium/index.html

97. Rousseau J, Castro A, Novo T, Maia C. *Dipylidium caninum* in the twenty-first century: epidemiological studies and reported cases in companion animals and humans. Parasit Vectors. 2022;15:131. PubMed https://doi.org/10.1186/s13071-022-05243-5

98. Cabello RR, Ruiz AC, Feregrino RR, Romero LC, Feregrino RR, Zavala JT. *Dipylidium caninum* infection. BMJ Case Rep. 2011;2011:bcr0720114510. PubMed https://doi.org/10.1136/bcr.07.2011.4510

99. Western College of Veterinary Medicine. *Dipylidium caninum* [cited 2024 Jul 3]. https://wcvm.usask.ca/learnaboutparasites/parasites/dipylidium-caninum.php

100. Lyons MA, Malhotra R, Thompson CW. Investigating the free-roaming dog population and gastrointestinal parasite diversity in Tulúm, México. PLoS One. 2022;17:e0276880. PubMed https://doi.org/10.1371/journal.pone.0276880

101. Nelson CT. Heartworm and related nematodes. In: Sykes JE, editor. Greene's infectious diseases of the dog and cat, 5th edition. St. Louis, MO: Elsevier; 2023. p. 1399–417.

102. Rishniw M. Veterinary partner [cited 2025 Mar 19]. https://www.vin.com/doc/?id=8156779

103. Malik D, Amaraneni A, Singh S, Roach R. Man's best friend: how humans can develop *Dirofilaria immitis* infections. IDCases. 2016;4:43–5. PubMed https://doi.org/10.1016/j.idcr.2016.03.003

104. Anvari D, Narouei E, Daryani A, Sarvi S, Moosazadeh M, Ziaei Hezarjaribi H, et al. The global status of *Dirofilaria immitis* in dogs: a systematic review and meta-analysis based on published articles. Res Vet Sci. 2020;131:104–16. PubMed https://doi.org/10.1016/j.rvsc.2020.04.002

105. Centers for Disease Control and Prevention. Dirofilariasis [cited 2025 Feb 19]. https://www.cdc.gov/dpdx/dirofilariasis/index.html

106. Eckert J, Deplazes P. Biological, epidemiological, and clinical aspects of echinococcosis, a zoonosis of increasing concern. Clin Microbiol Rev. 2004;17:107–35. PubMed https://doi.org/10.1128/CMR.17.1.107-135.2004

107. Torgerson PR, Budke CM. Echinococcosis—an international public health challenge. Res Vet Sci. 2003;74:191–202. PubMed https://doi.org/10.1016/S0034-5288(03)00006-7

108. The Center for Food Security and Public Health. Echinococcosis [cited 2025 Mar 19]. https://www.cfsph.iastate.edu/Factsheets/pdfs/echinococcosis.pdf

109. Eckert J, Gemmell MA, Meslin F, Pawłowski ZS, editors. WHO-OIE manual on echinococcosis in human and animals: a public health problem of global concern. Paris: OIE; 2001.

110. Gessese AT. Review on epidemiology and public health significance of Hydatidosis. Vet Med Int. 2020;2020:8859116. PubMed https://doi.org/10.1155/2020/8859116

111. Centers for Disease Control and Prevention. Echinococcosis [cited 2025 Mar 24]. https://www.cdc.gov/dpdx/echinococcosis/index.html

112. Merck Manual Veterinary Manual. Zoonotic diseases: parasitic diseases [cited 2024 Jul 3]. https://www.merckvetmanual.com/multimedia/table/zoonotic-diseases-parasitic-diseases

113. D'Alessandro A, Rausch RL. New aspects of neotropical polycystic (*Echinococcus vogeli*) and unicystic (*Echinococcus oligarthrus*) echinococcosis. Clin Microbiol Rev. 2008;21:380–401. PubMed https://doi.org/10.1128/CMR.00050-07

114. World Organisation for Animal Health. Echinococcosis [cited 2025 Mar 24]. https://www.woah.org/en/disease/echinococcosis/

115. Public Health Agency of Canada. Pathogen safety data sheets: infectious substances– *Echinococcus granulosus* [cited 2025 Feb 19]. https://www.canada.ca/en/public-health/services/laboratory-

biosafety-biosecurity/pathogen-safety-data-sheets-risk-assessment/echinococcus-granulosus-pathogen-safety-data-sheet.html

116. Victoria Department of Health. Hydatid disease (echinococcosis) [cited 2025 Feb 19]. https://www.health.vic.gov.au/infectious-diseases/hydatid-disease-echinococcosis

117. Weese JS, Evason M, editors. Infectious diseases of the dog and cat, 1st edition. Boca Raton, FL: CRC Press; 2019.

118. Ministry of Health and Long-Term Care. Management of *Echinococcus Multilocularis* infections in animals guideline, 2019 [cited 2025 Feb 19]. https://www.rcdhu.com/wp-content/uploads/2019/04/Management-of-EM-Infections-in-Animals-2019.pdf

119. Western College of Veterinary Medicine. *Echinococcus multilocularis* [cited 2025 Mar 24]. https://wcvm.usask.ca/learnaboutparasites/parasites/echinococcus-multilocularis.php

120. Companion Animal Parasite Council. *Filaroides hirthi* [cited 2024 Jul 4]. https://capcvet.org/guidelines/filaroides-hirthi

121. Robertson LJ. Merck Manual Veterinary Manual. Giardiasis in animals [cited 2024 Jul 4]. https://www.merckvetmanual.com/digestive-system/giardiasis-giardia/giardiasis-in-animals

122. Western College of Veterinary Medicine. *Giardia* species [cited 2024 Jul 4]. https://wcvm.usask.ca/learnaboutparasites/parasites/giardia-species.php

123. Cohen A. *Giardia*: infection, treatment and prevention [cited 2025 Mar 24]. https://www.vet.cornell.edu/departments-centers-and-institutes/riney-canine-health-center/canine-health-information/giardia-infection-treatment-and-prevention

124. Companion Animal Parasite Council. *Giardia* [cited 2024 Jul 4]. https://capcvet.org/guidelines/giardia

125. Mayo Clinic. *Giardia* infection (giardiasis) [cited 2024 Jul 4]. https://www.mayoclinic.org/diseases-conditions/giardia-infection/symptoms-causes/syc-20372786

126. Vincent-Johnson N, Baneth G, Allen KE. Hepatozoonosis. In: Sykes JE, editor. Greene’s infectious diseases of the dog and cat, 5th edition. St. Louis, MO: Elsevier; 2023. p. 1230–47.

127. Companion Animal Parasite Council. American canine hepatozoonosis [cited 2024 Jul 4]. https://capcvet.org/guidelines/american-canine-hepatozoonosis

128. Companion Animal Parasite Council. Schistosomiasis [cited 2024 Sep 17]. https://capcvet.org/guidelines/schistosomiasis

129. Baniya A, Goldy CJ, Ardpairin J, Achi P, Chang YW, Adrianza RC, et al. Canine schistosomiasis in the West Coast: *Heterobilharzia americana* in two natural intermediate hosts found in the Colorado River, California. Pathogens. 2024;13:245. PubMed https://doi.org/10.3390/pathogens13030245

130. Baneth G, Petersen C, Solano-Gallego L, Sykes JE. Leishmaniosis. In: Sykes JE, editor. Greene's infectious diseases of the dog and cat, 5th edition. St. Louis, MO: Elsevier; 2023. p. 1179–202.

131. Petersen CA, Barr SC. Canine leishmaniasis in North America: emerging or newly recognized? Vet Clin North Am Small Anim Pract. 2009;39:1065–74, vi. PubMed https://doi.org/10.1016/j.cvsm.2009.06.008

132. Petersen C. MSD Manual Veterinary Manual. 2020. Leishmaniosis in dogs [cited 2025 Mar 24]. https://www.msdvetmanual.com/infectious-diseases/leishmaniosis/leishmaniosis-in-dogs

133. Williams K, Llera R, Ward E. Leishmaniasis in dogs [cited 2025 Feb 20]. https://vcahospitals.com/know-your-pet/leishmaniasis-in-dogs

134. Naucke TJ, Amelung S, Lorentz S. First report of transmission of canine leishmaniosis through bite wounds from a naturally infected dog in Germany. Parasit Vectors. 2016;9:256. PubMed https://doi.org/10.1186/s13071-016-1551-0

135. Public Health Agency of Canada. Leishmaniasis [cited 2025 Mar 24]. https://www.canada.ca/en/public-health/services/diseases/leishmaniasis.html

136. Sykes JE. Neorickettsiosis. In: Sykes JE, editor. Greene's infectious diseases of the dog and cat, 5th edition. St. Louis, MO: Elsevier; 2023. p. 571–81.

137. Western College of Veterinary Medicine. *Nanophyetus salmincola* [cited 2024 Sep 18]. https://wcvm.usask.ca/learnaboutparasites/parasites/nanophyetus-salmincola.php

138. Western College of Veterinary Medicine. *Neospora caninum* [cited 2025 Feb 20]. https://wcvm.usask.ca/learnaboutparasites/parasites/neospora-caninum.php

139. McAllister MM. Merck Manual Veterinary Manual. Neosporosis in animals. 2020 [cited 2025 Feb 20]. https://www.merckvetmanual.com/infectious-diseases/neosporosis/neosporosis-in-animals

140. Animal Health Ireland. *Neospora caninum* [cited 2025 Feb 19]. https://animalhealthireland.ie/assets/uploads/2021/04/AHI-Parasite-Control-Neospora-2021.pdf?dl=1

141. Companion Animal Parasite Council. *Onchocerca lupi* [cited 2024 Sep 18]. https://capcvet.org/guidelines/onchocerca-lupi/

142. Verocai GG, Conboy G, Lejeune M, Marron F, Hanna P, MacDonald E, et al. *Onchocerca lupi* nematodes in dogs exported from the United States into Canada. Emerg Infect Dis. 2016;22:1477–9. PubMed https://doi.org/10.3201/eid2208.151918

143. Bowers Wu D, Ko B, Lopez Hernandez G, Botros J, Spader H, Sapp S, et al. Neuroinvasive *Onchocerca lupi* infection in a ten-year-old girl. Case Rep Infect Dis. 2022;2022:9773058.

144. Rojas A, Morales-Calvo F, Salant H, Otranto D, Baneth G. Zoonotic ocular onchocercosis by *Onchocerca lupi.* Yale J Biol Med. 2021;94:331–41. PubMed

145. Merck Manual Veterinary Manual. Flukes that infect dogs [cited 2025 Mar 25]. https://www.merckvetmanual.com/multimedia/table/flukes-that-infect-dogs

146. Ogorodova LM, Fedorova OS, Sripa B, Mordvinov VA, Katokhin AV, Keiser J, et al.; TOPIC Consortium. Opisthorchiasis: an overlooked danger. PLoS Negl Trop Dis. 2015;9:e0003563. PubMed https://doi.org/10.1371/journal.pntd.0003563

147. Peregrine AS, Merck Manual Veterinary Manual. Flukes in small animals [cited 2025 Mar 25]. https://www.merckvetmanual.com/digestive-system/gastrointestinal-parasites-of-small-animals/flukes-in-small-animals

148. Centers for Disease Control and Prevention. Opisthorchiasis [cited 2025 Mar 25]. https://www.cdc.gov/dpdx/opisthorchiasis/index.html

149. Marie C, Petri WA Jr. Merck Manual Professional Version. 2023. Opisthorchiasis [cited 2025 Mar 25]. https://www.merckmanuals.com/en-ca/professional/infectious-diseases/trematodes-flukes/opisthorchiasis

150. Marcos LA, Terashima A, Gotuzzo E. Update on hepatobiliary flukes: fascioliasis, opisthorchiasis and clonorchiasis. Curr Opin Infect Dis. 2008;21:523–30. PubMed https://doi.org/10.1097/QCO.0b013e32830f9818

151. Tonozzi CC. Merck Manual Veterinary Manual. Lung flukes in dogs and cats. 2022. [cited 2025 Mar 25]. https://www.merckvetmanual.com/respiratory-system/respiratory-diseases-of-small-animals/lung-flukes-in-dogs-and-cats

152. Ontario Animal Health Network. Infosheet: *Paragonimus kellicotti* for veterinarians [cited 2024 Sep 27]. https://www.oahn.ca/resources/paragonimus-kellicotti/

153. Centers for Disease Control and Prevention. About Paragonimiasis [cited 2025 Mar 25]. https://www.cdc.gov/paragonimus/about/index.html

154. Ketzis JK, Little SE, Wulcan JM. Miscellaneous nematode infections. In: Sykes JE, editor. Greene's infectious diseases of the dog and cat, 5th edition. St. Louis, MO: Elsevier; 2023. p. 1485–504.

155. Western College of Veterinary Medicine. *Physaloptera* species [cited 2024 Sep 27]. https://wcvm.usask.ca/learnaboutparasites/parasites/physaloptera-species.php

156. Companion Animal Parasite Council. *Physaloptera* spp. [cited 2024 Sep 27]. https://capcvet.org/guidelines/physaloptera-spp/

157. More GA. Merck Manual Veterinary Manual. 2018. Sarcocystosis in dogs [cited 2024 Sep 29]. https://www.merckvetmanual.com/dog-owners/bone-joint-and-muscle-disorders-of-dogs/sarcocystosis-in-dogs

158. Western College of Veterinary Medicine. *Sarcocystis* species [cited 2025 Mar 25]. https://wcvm.usask.ca/learnaboutparasites/parasites/sarcocystis-species.php

159. Centers for Disease Control and Prevention. Sarcocystosis [cited 2025 Mar 25]. https://www.cdc.gov/dpdx/sarcocystosis/index.html

160. Western College of Veterinary Medicine. *Sarcoptes* species—sarcoptic mange or scabies [cited 2025 Mar 25].. https://wcvm.usask.ca/learnaboutparasites/parasites/sarcoptes-species-sarcoptic-mange-or-scabies.php

161. Dryden M. Merck Manual Veterinary Manual. 2023. Mange in dogs and cats [cited 2024 Sep 29]. https://www.merckvetmanual.com/integumentary-system/mange/mange-in-dogs-and-cats

162. Little SE, Cortinas R. Mites. In: Sykes JE, editor. Greene's infectious diseases of the dog and cat, 5th edition. St. Louis, MO: Elsevier; 2023. p. 1378–98.

163. Peregrine AS. Merck Manual Veterinary Manual. 2023. *Spirocerca lupi* in small animals [cited 2025 Mar 25]. https://www.merckvetmanual.com/digestive-system/gastrointestinal-parasites-of-small-animals/spirocerca-lupi-in-small-animals

164. van der Merwe LL, Kirberger RM, Clift S, Williams M, Keller N, Naidoo V. *Spirocerca lupi* infection in the dog: a review. Vet J. 2008;176:294–309. PubMed https://doi.org/10.1016/j.tvjl.2007.02.032

165. Peregrine AS. Merck Manual Veterinary Manual. 2023. *Strongyloides* sp in small animals [cited 2025 Mar 25]. https://www.merckvetmanual.com/digestive-system/gastrointestinal-parasites-of-small-animals/strongyloides-sp-in-small-animals

166. Paradies P, Iarussi F, Sasanelli M, Capogna A, Lia RP, Zucca D, et al. Occurrence of strongyloidiasis in privately owned and sheltered dogs: clinical presentation and treatment outcome. Parasit Vectors. 2017;10:345. PubMed https://doi.org/10.1186/s13071-017-2275-5

167. Western College of Veterinary Medicine. *Strongyloides stercoralis* [cited 2024 Oct 1]. https://wcvm.usask.ca/learnaboutparasites/parasites/strongyloides-stercoralis.php

168. Gorgani-Firouzjaee T, Kalantari N, Chehrazi M, Ghaffari S, Shahdin S. Global prevalence of *Strongyloides stercoralis* in dogs: a systematic review and meta-analysis. J Helminthol. 2022;96:e11. PubMed https://doi.org/10.1017/S0022149X21000808

169. Thamsborg SM, Ketzis J, Horii Y, Matthews JB. *Strongyloides* spp. infections of veterinary importance. Parasitology. 2017;144:274–84. PubMed https://doi.org/10.1017/S0031182016001116

170. Centers for Disease Control and Prevention. Strongyloidiasis [cited 2025 Mar 25]. https://www.cdc.gov/dpdx/strongyloidiasis/index.html

171. Trasviña-Muñoz E, López-Valencia G, Centeno PÁ, Cueto-González SA, Monge-Navarro FJ, Tinoco-Gracia L, et al. Prevalence and distribution of intestinal parasites in stray dogs in the northwest area of Mexico. Austral J Vet Sci. 2017;49:105–11. https://doi.org/10.4067/S0719-81322017000200105

172. Mora Carpio AL, Meseeha M. Strongyloidiasis. In: StatPearls. Treasure Island (FL): StatPearls Publishing; 2023 [cited 2025 Mar 25]. http://www.ncbi.nlm.nih.gov/books/NBK436024

173. Companion Animal Parasite Council. *Taenia* spp. [cited 2025 Mar 25]. https://capcvet.org/guidelines/taenia/

174. Adolph CB, Peregrine AS. Tapeworms. In: Sykes JE, editor. Greene's infectious diseases of the dog and cat, 5th edition. St. Louis, MO: Elsevier; 2023. p. 1455–84.

175. Public Health Agency of Canada. Pathogen safety data sheets: infectious substances– *Toxocara canis, Toxocara cati* [cited 2025 Mar 25]. https://www.canada.ca/en/public-health/services/laboratory-biosafety-biosecurity/pathogen-safety-data-sheets-risk-assessment/toxocara-canis-toxocara-cati.html

176. Centers for Disease Control and Prevention. Toxocariasis [cited 2025 Mar 25]. https://www.cdc.gov/dpdx/toxocariasis/index.html

177. Acha PN, Syzfres B. Visceral Larva Migrans and Toxocariasis. In: Zoonoses and communicable diseases common to man and animals, 3rd edition. Washington, DC: Pan American Health Organization; 2013. p. 305–11.

178. Peregrine AS. Merck Manual Veterinary Manual. 2023. Roundworms in small animals [cited 2025 Mar 25]. https://www.merckvetmanual.com/digestive-system/gastrointestinal-parasites-of-small-animals/roundworms-in-small-animals

179. Spickler AR, The Center for Food Security and Public Health. Toxocariasis [cited 2025 Feb 19]. https://www.cfsph.iastate.edu/Factsheets/pdfs/toxocariasis.pdf

180. Western College of Veterinary Medicine. *Toxocara canis* [cited 2025 Mar 25]. https://wcvm.usask.ca/learnaboutparasites/parasites/toxocara-canis.php

181. Companion Animal Parasite Council. *Trichuris vulpis* [cited 2025 Mar 25]. https://capcvet.org/guidelines/trichuris-vulpis/

182. Peregrine AS. Merck Manual Veterinary Manual. 2023. Whipworms in small animals [cited 2025 Mar 25]. https://www.merckvetmanual.com/digestive-system/gastrointestinal-parasites-of-small-animals/whipworms-in-small-animals

183. Saunders AB, Hamer SA. Chagas disease in dogs: transmission, diagnosis, treatment, and prevention [cited 2025 Mar 25]. https://todaysveterinarypractice.com/parasitology/chagas-disease-dogs/

184. Senestraro A. Chagas disease in dogs (kissing bug disease) [cited 2025 Mar 25]. https://www.petmd.com/dog/conditions/infectious-parasitic/c_dg_chagas_disease

185. Evason M, Stull J, American Kennel Club Canine Health Foundation. American Trypanosomiasis (Chagas disease) information for dog owners [cited 2025 Mar 25]. https://www.akcchf.org/canine-health/top-health-concerns/current-topics-in-infectious-disease/AKC-CHF-Trypanosoma-Chagas-Disease-Fact-Sheet-1.pdf

186. Centers for Disease Control and Prevention. American Trypanosomiasis [cited 2025 Mar 25]. https://www.cdc.gov/dpdx/trypanosomiasisamerican/index.html

187. Public Health Agency of Canada. Trypanosoma cruzi: Infectious substances pathogen safety data sheet [cited 2025 Mar 25]. https://www.canada.ca/en/public-health/services/laboratory-biosafety-biosecurity/pathogen-safety-data-sheets-risk-assessment/trypanosoma-cruzi.html

188. Gascon J, Bern C, Pinazo MJ. Chagas disease in Spain, the United States and other non-endemic countries. Acta Trop. 2010;115:22–7. PubMed https://doi.org/10.1016/j.actatropica.2009.07.019

189. World Health Organization. Chagas disease [cited 2025 Mar 25]. https://www.who.int/news-room/fact-sheets/detail/chagas-disease-(american-trypanosomiasis)

190. Barr SC, Saunders AB, Sykes JE. Trypanosomiasis. In: Sykes JE, editor. Canine and feline infectious diseases, 1st edition. St. Louis, MO: Elsevier; 2014. p. 760–70.

191. Hamer SA, Saunders AB, Snowden KF, Sykes JE. Trypanosomiasis. In: Sykes JE, editor. Greene's infectious diseases of the dog and cat, 5th edition. St. Louis, MO: Elsevier; 2023. p. 1248–62.

192. Nwoha IO. A review on trypanosomosis in dogs and cats. Afr J Biotechnol. 2013;12:6432–42. https://doi.org/10.5897/AJB2013.12093

193. Creevy KE, Evans JB. Merck Manual Veterinary Manual. 2022. Infectious canine hepatitis [cited 2025 Mar 26]. https://www.merckvetmanual.com/infectious-diseases/infectious-canine-hepatitis/infectious-canine-hepatitis

194. Sykes JE. Infectious canine hepatitis. In: Sykes JE, editor. Canine and feline infectious diseases, 1st edition. St. Louis, MO: Elsevier; 2014. p. 182–6.

195. Barr SC, Bowman DD. Infectious canine hepatitis virus infection. In: Blackwell's five-minute veterinary consult clinical companion: canine and feline infectious diseases and parasitology, 2nd edition. Hoboken, NKJ: Wiley-Blackwell; 2011. p. 616–25.

196. Creevy KE, Evans JB. Merck Manual Veterinary Manual. 2022. Canine distemper [cited 2025 Mar 26]. https://www.merckvetmanual.com/infectious-diseases/canine-distemper/canine-distemper

197. Sykes JE. Canine distemper virus infection. In: Sykes JE, editor. Canine and feline infectious diseases, 1st edition. St. Louis, MO: Elsevier; 2014. p. 152–65.

198. Creevy KE, Evans JB. Merck Manual Veterinary Manual. 2022. Canine herpesvirus infection [cited 2025 Mar 26]. https://www.merckvetmanual.com/infectious-diseases/canine-herpesvirus-infection/canine-herpesvirus-infection

199. Davidson AP. Canine herpesvirus infection. In: Sykes JE, editor. Canine and feline infectious diseases, 1st edition. St. Louis, MO: Elsevier; 2014. p. 166–9.

200. Davidson AP, Sykes JE, Casal ML. Canine herpesvirus infection. In: Sykes JE, editor. Greene's infectious diseases of the dog and cat, 5th edition. St. Louis, MO: Elsevier; 2023. p. 301–9.

201. Evermann JF, Ledbetter EC, Maes RK. Canine reproductive, respiratory, and ocular diseases due to canine herpesvirus. Vet Clin North Am Small Anim Pract. 2011;41:1097–120. PubMed https://doi.org/10.1016/j.cvsm.2011.08.007

202. Luff JA, Munday JS. Papillomavirus infections. In: Sykes JE, editor. Greene's infectious diseases of the dog and cat, 5th edition. St. Louis, MO: Elsevier; 2023. p. 477–88.

203. Sykes JE, Luff JA. Viral papillomatosis. In: Sykes JE, editor. Greene's infectious diseases of the dog and cat, 5th edition. St. Louis, MO: Elsevier; 2023. p. 261–8.

204. Sykes JE. Canine parvovirus infections and other viral enteritides. In: Sykes JE, editor. Canine and feline infectious diseases, 1st edition. St. Louis, MO: Elsevier; 2014. p. 141–51.

205. American Veterinary Medical Association. Canine parvovirus [cited 2025 Mar 26]. https://www.avma.org/resources-tools/pet-owners/petcare/canine-parvovirus

206. Hayes MA, Russell RG, Babiuk LA. Sudden death in young dogs with myocarditis caused by parvovirus. J Am Vet Med Assoc. 1979;174:1197–203. PubMed https://doi.org/10.2460/javma.1979.174.11.1197

207. Lenghaus C, Studdert MJ, Finnie JW. Acute and chronic canine parvovirus myocarditis following intrauterine inoculation. Aust Vet J. 1980;56:465–8. PubMed https://doi.org/10.1111/j.1751-0813.1980.tb02555.x

208. Sykes JE. Canine viral respiratory infections. In: Sykes JE, editor. Canine and feline infectious diseases, 1st edition. St. Louis, MO: Elsevier; 2014. p. 170–81.

209. American Veterinary Medical Association. Canine influenza: Veterinary resources [cited 2025 Feb 16]. https://www.avma.org/resources-tools/animal-health-and-welfare/animal-health/canine-influenza-veterinary-resources

210. Hilling K, Hanel R. Canine influenza [cited 2025 Feb 19]. https://vetfolio-vetstreet.s3.amazonaws.com/mmah/e3/8647a6d28f4b469584562955d5828d/filePV0610_hilling_CE.pdf

211. Parry N. Clinical signs & diagnosis of canine influenza [cited 2025 Mar 26]. https://www.dvm360.com/view/clinical-signs–diagnosis-of-canine-influenza

212. Crawford PC, Dubovi EJ, Castleman WL, Stephenson I, Gibbs EPJ, Chen L, et al. Transmission of equine influenza virus to dogs. Science. 2005;310:482–5. PubMed https://doi.org/10.1126/science.1117950

213. Worms and Germs Blog. H3N2 canine influenza for veterinarians [cited 2025 Mar 26]. https://www.wormsandgermsblog.com/files/2018/01/H3N2-CIV-Infosheet-V1.pdf
